# A retrospective review on minimally invasive technique via endoscopic thoracic sympathectomy (ETS) in the treatment of severe primary hyperhidrosis: Experiences from the National Heart Institute, Malaysia

**DOI:** 10.12688/f1000research.14777.1

**Published:** 2018-05-29

**Authors:** Ahmad Farouk Musa, Vignaa Prashanth Gandhi, Jeswant Dillon, Rusli Bin Nordin

**Affiliations:** 1 Jeffrey Cheah School of Medicine and Health Sciences, Monash University Malaysia, Bandar Sunway, Malaysia; 2 Department of Cardiothoracic Surgery, National Heart Institute, Kuala Lumpur, Malaysia

**Keywords:** Primary hyperhidrosis, Endoscopic thoracic sympathectomy, Compensatory sweating, National Heart Institute

## Abstract

**Background**: Hyperhidrosis is due to the hyperactive autonomic stimulation of the sweat glands in response to stress. Primary hyperhidrosis is a common yet psychologically disabling condition. This study will describe our experience in managing hyperhidrosis via endoscopic thoracic sympathectomy (ETS).

**Methods: **The information was obtained from the patient records from 1 ^st^ January 2011 until 31 ^st^ December 2016. Pertinent information was extracted and keyed into a study proforma.

**Results: ** 150 patients were operated on but only 118 patients were included in this study. The mean age was 22.9±7.3 years. The majority (54.2%) had palmar-plantar hyperhidrosis and 39.8% had associated axillary hyperhidrosis. Excision of the sympathetic nerve chain and ganglia were the main surgical technique with the majority (55.9%) at T2-T3 level. Mean ETS procedure time was 46.6±14.29 minutes with no conversion. Surgical complications were minimal and no Horner’s Syndrome reported. Mean hospital stay was 3.5±1.05 days. The majority of patients (67.8%) had only one follow-up and only half of the study sample (58.5%) complained mild to moderate degree of compensatory sweating, even though the long-term resolution is yet to be determined by another study. Following ETS, 98.3% of patients had instant relief and resolved their palmar hyperhidrosis. Predictors of CS were sympathectomy level and follow-up. The odds of reporting CS was 2.87 times in patients undergoing ETS at the T2-T3 level compared to those undergoing ETS at the T2-T4 level. The odds of reporting CS was 13.56 times in patients having more than one follow-up compared to those having only one follow-up.

**Conclusion: **We conclude that ETS is a safe, effective and aesthetically remarkable procedure for the treatment of primary hyperhidrosis  with only half of the patients developing mild to moderate degree of CS. Significant predictors of CS were sympathectomy level during ETS and frequency of follow-up after ETS.

## Introduction

Hyperhidrosis is a pathological condition of excessive sweating beyond the physiological needs for thermoregulation, which may severely disrupt a person’s quality of life and make their social interaction difficult ^1, 2^. Primary hyperhidrosis results from the overactive sudomotor system which controls sweat output with no apparent cause ^2^. And it is important to differentiate this condition from secondary hyperhidrosis, which can be due to many conditions including endocrine disorders, infection, genetic, malignancy, neurologic and miscellaneous causes ^3^.

Epidemiological studies in the United States ^4, 5^ indicated that the prevalence of hyperhidrosis ranged from 0.6% to 9% in the particular populations studied; indicating this disease is not a rare event. It is interesting to note that the prevalence outside the United States are significantly higher and vary extensively. The observed prevalence of primary hyperhidrosis is 13.9% in Japan, 14.5% in China, 16.3% in Germany, 16.7% in Canada and 20.3% in Sweden ^6– 9^. And over the past several decades, different researchers ^10– 13^ have speculated positive family histories in patients with primary hyperhidrosis, widely ranging from 5% to 50%; however, there was no substantial data being offered until recently. Ro *et al*. ^14^ also reported in 2002 that patients with hyperhidrosis had 65% genetic prevalence of autosomal dominance with variable penetrance and with no evidence of sex-linked transmission. This means that a child of a parent with hyperhidrosis has a 25% chance of developing hyperhidrosis due to the likelihood of phenotypic expression of 0.28. It is also important to note that there is potential genetic linkage to chromosome 14 ^3^.

**Figure 1.**
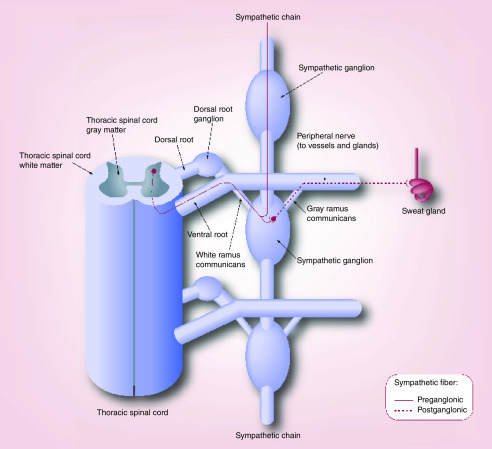
Anatomy of the sympathetic chain in relation to the spinal cord and sweat glands as the target organ ^16^. Reprinted from Thoracic Surgery Clinics, Volume 18, Issue 2, Shargall Y, Spratt E, Zeldin RA. Hyperhidrosis: What is it and why does it occur? Pages 125–132, Copyright (2008) with permission from Elsevier.

There are important anatomic points that need to be understood as they relate to the main intervention discussed in this paper; however, it should be understood that the neuroanatomy of the sweating mechanism is complex beyond the scope of this paper. In relation to hyperhidrosis, the anatomy involves nerve fibers that originate from the hypothalamic preoptic sweat center to the spinal cord where they meet and synapse with the preganglionic fibers. These fibers then leave the spinal cord coursing along the ventral nerve roots and terminate in the sympathetic ganglia ^15, 16^. In the sympathetic ganglion, pre- and postganglionic fibers synapse, which then track peripherally and end at their target organs, in this case, the sweat glands ^16^ ( Figure 1).

The pathophysiology of the underlying hyperhidrosis is intricate and not fully understood, although, it is commonly believed to stem from an exaggerated central response to normal emotional stress or environmental stimuli. In most cases, it occurs intermittently and spontaneously. And it should not be considered as a psychiatric disorder ^17^. In primary palmar hyperhidrosis, there is a hyper-functioning emotional element of the central sudomotor nervous system, substantiated by the observation that excessive sweating does not transpire during sleep and is aggravated by emotional stimuli. The body undergoes a number of physiological changes during mental stress such as increased skin sympathetic nerve activity, excessive sweating, pronounced vasoconstriction and a concomitant increase in evaporation jointly termed as “cold and clammy” hands ^18, 19^.

While the treatment of primary hyperhidrosis could be medical or surgical, this paper will focus on the surgical aspect of minimally invasive surgery known as endoscopic thoracic sympathectomy (ETS). Most ETS procedures are done bilaterally, with denervation of the sympathetic chain on both sides, and hence, the lungs have to be deflated in order to reach the sympathetic chain. Single lung anesthesia is accomplished by the placement of a suitable sized single or double lumen endotracheal tube. Most literatures ^20– 24^ describe the use of a double lumen tube rather than single, which is what our center practiced, as it is easier to achieve a single lung separation. At our center, two incisions are made for thoracoscopic access and ports with lumens. The skin incisions, of about 15mm, are made around the mid-axillary line over roughly the fifth intercostal space. After the underlying lungs are collapsed on one side, the pleural space is entered by blunt dissection. A 5 or 10mm 0-degree thoracoscope is introduced into the cavity. Under endoscopic guidance, a second “stab” incision is made near the sub-mammary fold.

The upper thoracic sympathetic trunk is located underneath the pleura as it traverses over the neck of the ribs. It can be usually confirmed by passing a probe along the neck of the rib and feeling the string-like solid trunk. The cervicothoracic (stellate) ganglion is obscured as it lies beneath a distinctive fat pad on the neck of the first rib. The first and the second rib can be distinguished by the location of the supreme intercostal artery and by the curvature of the rib ^25^. There is some controversy in regards to reaching a consensus as to which level of the ganglion (T2–T4) should be resected to achieve best results. The sympathetic chain is then resected, transected or ablated with a cautery, or divided with a harmonic scalpel or a clip is used, depending on the center and surgeon performing the surgery, though we at the Institute practiced thermo-resection and stripping. It is vital to identify and divide the Nerve of Kuntz. But cauterizing the trunk above the T2 ganglion should be done with caution as there is a risk of causing Horner’s syndrome, characterized by miosis, partial ptosis, anhidrosis, and enophthalmus, which is a disastrous and avoidable complication ^26^. After completing the surgery, an apical chest drain is placed at the anterior wound and the lung is re-inflated. And the procedure is then repeated on the other side.

It is interesting to note, at this stage, based on an analysis of forty-two different ETS techniques by Kopelman and Hashmonai ^27^, there were no distinct differences shown by the different techniques; if the correct level of interruption is achieved, the results are excellent and reproducible. The most imperative notion about nerve disruption is that there is adequate separation between the ends of the chain so that regeneration is halted. Although resection yields better results in terms of lower recurrence rates, most ETS procedures prefer ganglionic thermo-ablation or thermo-transection of the chain due to the relative simplicity of the latter rather than endoscopic resection ^28– 30^.

Although ETS is a minimally invasive procedure aimed at improving the quality of life, it is not without complications. The main side effects of ETS for hyperhidrosis are compensatory sweating (CS), bradycardia, and Horner’s syndrome. Chou *et al.* comments that generally, “the higher the level of interruption on the chain, the higher is the expected regret rate” ^31^. Horner’s syndrome is an undesirable complication, with its occurrence following surgery ranging widely from 0% to 40% ^32– 34^. The rate of this complication occurring is higher in patients who undergo ETS for facial hyperhidrosis, as injury by electrocautery, thermo-ablation, traction, or surrounding inflammation can occur due to improper identification of the second rib. Phantom sweating and gustatory sweating are two side effects of ETS and the etiology of which still remains obscure ^35, 36^. The former is a sensation of sweating in the target area after sympathectomy without any physical evidence of sweating, whereas the latter is mainly sweating on the face triggered by spicy and some other foods. Bradycardia has also been noted in several studies ^37, 38^ after ETS for hyperhidrosis.

CS is by far the most troublesome and disagreeable complication of ETS, producing subjective and objectively quantifiable increased sweating in body segments usually below the area of denervation such as the torso, groin, and lower extremities. However, the wide disparity of the occurrence of CS displayed in the literature clearly highlights the lack of standardization in reporting data. It is mindboggling that the exact same procedure would have such a huge difference of CS rates as it ranges from 3% to 98% ^39, 40^. Due to this unpredictable factor, all patients should be told preemptively of this possible complication and that it is irreversible ^41^. The pathophysiology of CS remains unknown and has not been fully described by the literature. Available data regarding the pathophysiology of CS is not fully verified, hence, the mechanism of CS is said to be multifactorial and, thus, difficult to quantify ^31^.

## Methods

### Study design

This is a single center retrospective review of patients who underwent ETS for primary hyperhidrosis at the National Heart Institute (IJN), Malaysia. Patients’ medical records from 1 ^st^ January 2011 until 31 ^st^ December 2015 were reviewed in a retrospective manner to identify the success rate of the surgery, occurrence rate of CS and also identify factors that might influence the rate of CS post ETS.

### Ethical statement

Ethical approval was obtained through the National Heart Institute Research Ethics Committee (IJNREC 206/2017). No amendment was made throughout the duration of the study. This study was also registered with the National Medical Research Register (NMRR-18-29-39863, Ministry of Health, Malaysia on the 12 ^th^ February 2018.

### Aims

The main aim of the research was to evaluate the efficacy of ETS, provide long-term follow-up data and investigate the occurrence, severity and significant predictors of CS at the National Heart Institute (IJN).

### Study variables

A number of potential pre-operative, intra-operative and post-operative variables were collected after a thorough literature review. The list of variables was reviewed and scrutinized multiple times prior to the start of data collection, taking into account professional advices and availability of certain information in existing medical records in this particular center. The list of variables was then compiled and structured into a proforma ( Supplementary File 1), which was used as our data collection sheet in this study.

### Inclusion and exclusion criteria

Every patient who had ETS in IJN from 1 ^st^ January 2011 to 31 ^st^ December 2015 was included in this study. All patients who had ETS for primary hyperhidrosis only were accounted for in this particular study. No patient underwent two ETS surgeries in IJN during this study period. The exclusion criteria for this study were patients who had primary hyperhidrosis, and having other medical conditions that cause hyperhidrosis (such as hyperhidrosis secondary to hyperthyroidism). All patient medical records had to be complete, and most importantly the follow up reports. Patients who defaulted follow up were not included in this study as it defeats the main aim of this study, which is to determine the rate of CS and success rate of ETS.

### Statistical analysis

Statistical analysis was done using the IBM SPSS Version 24.0. Continuous data were analyzed and depicted as means and standard deviations whereas categorical data were reported as frequencies and percentages. Chi Square and Fisher Exact tests were used to test the association between categorical variables. Differences were considered significant if *p*<0.05. Following a univariate binary logistic regression analysis for predicting CS, independent variables with *p* values of less than 0.25, were selected for entry into the multivariable analysis after checking for possible multicollinearity and interactions. In the multivariate binary logistic regression analyses, significant and independent predictors of CS were modeled using the stepwise method for the preliminary final model after checking for model fitness using three goodness-of-fit assessments: Hosmer-Lemeshow test, Classification Table, and area under the curve (AUC) of the receiver operating characteristics (ROC) curve. Based on these assessments, the final prediction model of CS will be identified. The percentage of variance explained by the final model will be indicated by two pseudo R square measures: Cox & Snell R square and the Nagelkerke R square.

## Results

### Patients’ characteristics

We operated on 150 patients from January 2011 to December 2015. However only 127 patients fit into the inclusion criteria with complete case records. Nine patients were then excluded in accordance to the exclusion criteria for not attending follow-up. The rest of the 118 patients were included for data analysis ( Table 1). The patients in this particular study sample demonstrated an almost equal distribution of male and female gender, with slightly a higher proportion of males (57.6%) having ETS compared to females (42.4%). The mean age of having ETS was 22.91 ± 7.3 years, the youngest being nine years old whilst the eldest was aged 52 years. The majority of the patients who had ETS were aged 21–30 years, representing 44.9% of the sample whereas patients aged 31 years and above represented the lowest proportion (11.9%) of the study sample.

**Table 1.** Characteristics of the study population.

Demographic characteristics		No.	%
Gender	Male	68	57.6
	Female	50	42.4
Age (year)	≤20	51	43.2
	21–30	53	44.9
	>31	14	11.9
	Mean (SD)	22.91 (7.27)	
Ethnic Group	Malay	94	79.7
	Chinese	16	13.6
	Indian	8	6.8
BMI (kg/m ^2^)	Underweight <18.5	11	9.8
	Normal (18.5-<25)	54	48.2
	Overweight (25-<30)	38	33.9
	Obese (≥30) *	9	8.0

* Six missing data for obese category (3 were excluded based on the location of their compensatory sweating and 3 due to loss to follow up), BMI: body mass index

The ethnic distribution of our study corresponds to the racial distribution of the Malaysian population ( Table 1). Malay was the dominant ethnic group (79.7%), followed by Chinese (13.6%) and Indian (6.8%). The pre-operative body mass index (BMI) distribution is as follows: the majority (48.2%) of patients was in the normal category, followed by overweight (33.9%), underweight (9.8%), and obese category (8.0%).

### Location of primary hyperhidrosis

**Figure 2.**
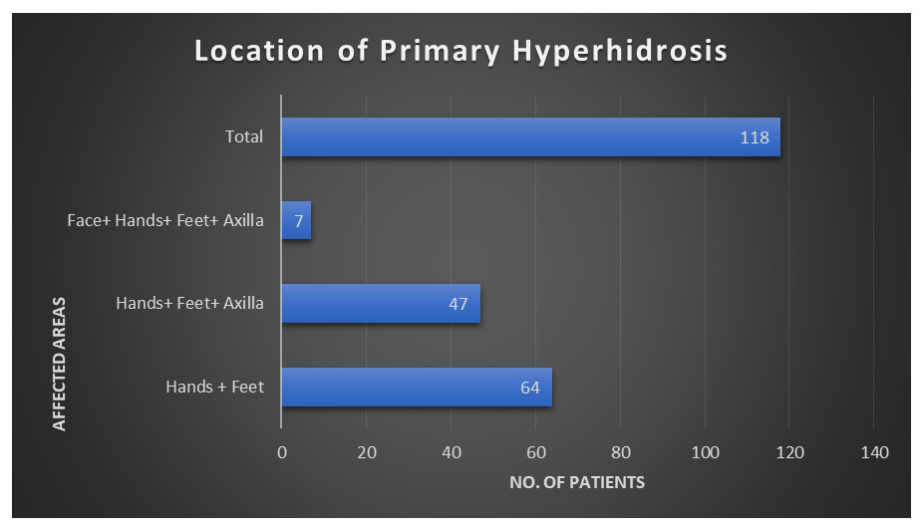
Distribution of primary hyperhidrosis by area affected.

All 118 patients had more than two areas affected by primary hyperhidrosis, hence they are grouped into areas that affected them the most ( Figure 2). Out of the 118 patients, 64 had primary hyperhidrosis affecting their hands and feet, which accounts for the majority of the sample (54.2%). Patients who had the disease affecting their axilla and on top of their hands and feet accounted for 39.8% of the sample. The most severe presentation of primary hyperhidrosis, which affects more than three regions (hands, feet, axilla and the face) only totaled seven patients (5.9%). It is also important to note that one patient had isolated primary hyperhidrosis on the hands, but was lost to follow up, hence not included in this study.

### Level of sympathectomy performed

There were very little variations to the surgery that had been performed, as we were looking at a single center performing a state of the art surgery. The technique used by all surgeons was thermo-resection and stripping. The level of sympathectomy that was predominantly used in this sample was T2-T3 accounting for 55.9% of the total surgeries ( Table 2). This is followed by the T2, T3, and T4 level, accounting for 41.5% of the surgeries. Three patients had sympathectomy at levels T3-T4 that only contributed 2.5% to the total surgeries. All surgeries in IJN were performed in a similar manner with exceptions to the level of sympathectomy. The mean duration of surgery was 46.6 ± 14.29 minutes. Impressively, the mean duration of stay in the hospital after ETS was only 3.5 ± 1.05 days.

**Table 2.** Level of sympathectomy performed.

Level of sympathectomy performed	No.	%
T2–T3	66	55.9
T3–T4	3	2.5
T2, T3 & T4	49	41.5
Total	118	100.0

### Complications of ETS

In IJN, the number of patients experiencing any complications operatively and post- operatively was staggeringly low. The majority of the patients (89.1%) who underwent ETS had no complication during surgery or post operatively ( Table 3). Seven (5.9%) patients developed pneumothorax as a result of the surgery and four (3.4%) patients had severe pain at the operation site. One patient developed bradycardia, a relatively rare complication resulting from sympathectomy of the thoracic ganglia. Interestingly, one patient developed post-sympathetic neuralgia, which is incapacitating due to the constant pain and interferes with the patient’s daily life but none developed Horner’s syndrome.

**Table 3.** Complications of endoscopic thoracic sympathectomy.

Complications	No.	%
Pneumothorax	7	5.9
Pain	4	3.4
Bradycardia	1	0.8
Post Sympathetic Neuralgia	1	0.8
None	105	89.1
Total	118	100.0

### Post-ETS results

All of our patients who had ETS had at least one follow-up post-ETS. In IJN, 67.8%, 24.6%, and 7.6% of the patients had one, two, or three follow-up, respectively. Our primary aim was to look at the resolution of palmar hyperhidrosis after ETS. In IJN, the outcomes were excellent as 98.3% of our patients had almost complete (90–100%) resolution of palmar hyperhidrosis. One patient had partial resolution of symptoms and one more patient had no change in the palmar hyperhidrosis.

We also noted the degree of resolution in other regions, such as the axilla and plantar areas. Only 6 (5.1%) patients had axillary and plantar hyperhidrosis resolution post ETS whereas the majority of the patients had partial resolutions of symptoms only ( Table 4). We noticed that 35.6% of patients with hyperhidrosis involving the axillary region had partial (60–90%) resolution of symptoms and 61.9% of patients from the plantar group had partial resolution of their primary hyperhidrosis. It also important to highlight that 17.8% of the patients from our center who had ETS did not have any changes in the degree of sweating at their plantar region.

**Table 4.** Degree of resolution of primary hyperhidrosis in patients post endoscopic thoracic sympathectomy by area.

Area	Degree of Resolution	No. of Patients	%
Palmar	Complete (90–100%)	116	98.3
	Partial (60–90%)	1	0.8
	No change	1	0.8
Axillary	Complete (90–100%)	6	5.1
	Partial (60–90%)	42	35.8
	No change	6	5.1
Plantar	Complete (90–100%)	6	5.1
	Partial (60–90%)	73	61.9
	No change	21	17.8

### Compensatory sweating

**Figure 3.**
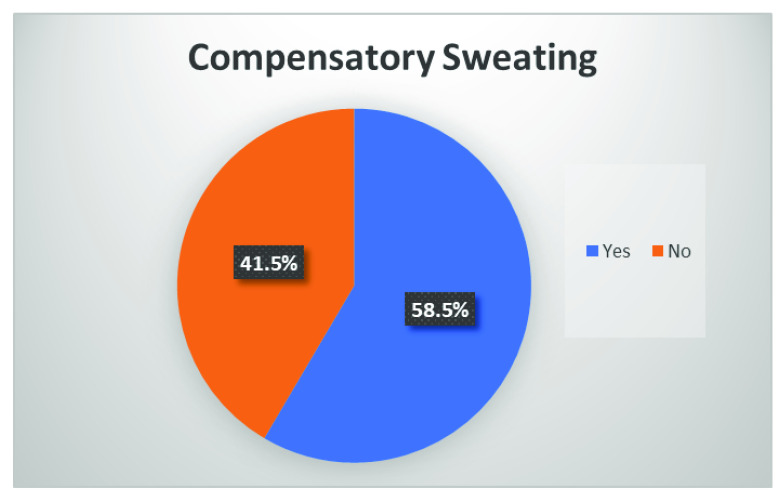
Distribution of patients reporting compensatory sweating.

CS is a pertinent factor whenever ETS is performed for primary hyperhidrosis. It is one of the major factors that have always influenced the decision as to whether ETS should be done or not. In our study, the rate of CS was 58.5% ( Figure 3).

**Figure 4.**
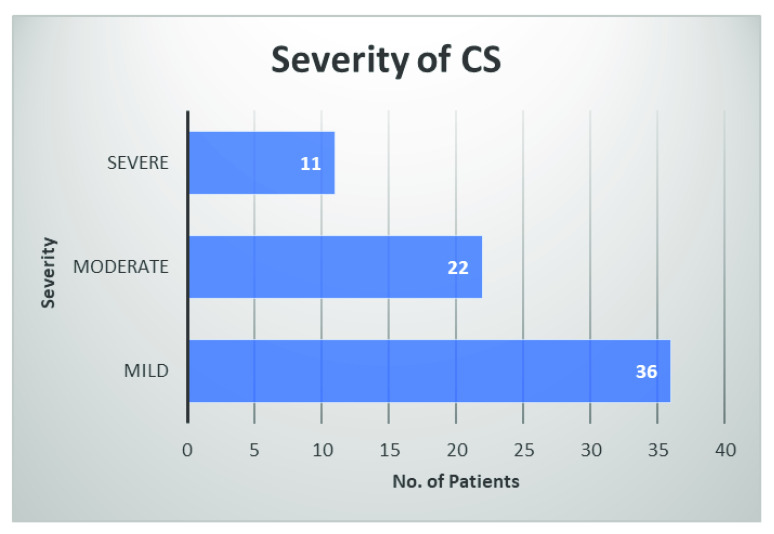
Distribution of patients affected by compensatory sweating (CS) according to severity.

Of those who developed CS, 52.2% reported the mild form of CS ( Figure 4). It means that the majority of patients experienced a much more tolerable form of CS compared to their palmar hyperhidrosis. Only 15.9% of those who developed CS reported the severe form CS and described the severity and location of the sweating as worse than their initial condition. Some also regretted their decision to have the surgery.

There are many areas that may be affected by CS post-ETS. These areas have been grouped according to their frequency. The area that was most affected by CS was the back (29.0%) whilst the least being the chest and face (2.9%). There appears to be a certain predilection to the combination of areas where CS affected the most. CS occurring at the back and back of thighs was as high as 18.8% followed by 14.5% at the trunk and back of thighs. Other areas affected by CS post-ETS are shown in Table 5 below.

**Table 5.** Distribution of areas affected by compensatory sweating (CS) post-endoscopic thoracic sympathectomy.

Areas affected by CS	Number of patients	%
Back	20	29.0
Chest	2	2.9
Abdomen	9	13.0
Back of thighs	4	5.8
Face	2	2.9
Back of thighs + back	13	18.8
Chest + abdomen	9	13.0

Patients who had CS and who had at least two follow-ups were also asked regarding the progression of CS. They were grouped into three categories: reducing, increasing or status quo. Out of the 69 patients who reported CS, the majority (88.4%) reported that their CS remained the same in their later follow-ups post ETS. Four (5.8%) patients claimed that their CS worsened over time and four (5.8%) patients claimed that their CS improved over time.

### Association of patient characteristics and CS

A Chi-Square and Fisher exact test of independence was performed to see if there is an association between age, ethnicity, gender, BMI and CS among 118 IJN patients ( Table 6). No significant association was observed between all four patient characteristics and CS.

**Table 6.** Association between patient characteristics and compensatory sweating (CS) in 118 patients following endoscopic thoracic sympathectomy.

Variables		CS		X ^2^ (df)	*P* ^*^
		Yes	No		
Age (year)	≤20	29 (42.0)	22 (44.9)	1.098 (2)	0.577
	21–30	30 (43.5)	23 (46.9)		
	>30	10 (14.5)	4 (8.2)		
Ethnicity	Malay	54 (78.3)	40 (81.6)	0.914 ^a^	0.690
	Indian	9 (13.0)	7 (14.3)	(2)	
	Chinese	6 (8.7)	2 (4.1)		
Gender	Male	43 (62.3)	25 (51.0)	1.498 (1)	0.221
	Female	26 (37.7)	24 (49.0)		
BMI (kg/m ^2^)	Underweight	6 (12.5)	5 (7.8)	1.025 (3)	0.795
	Normal	21 (43.8)	33 (51.6)		
	Overweight	17 (35.4)	21 (32.8)		
	Obese ^b^	4 (8.3)	5 (7.8)		

χ ^2^= Chi-square value, df=degree of freedom, ^a^Fisher exact test, ^b^Six data missing from obese category, *Significant at *p*<0.05, BMI: body mass index

### Association of Location of Primary Hyperhidrosis, Level of Sympathectomy, and Compensatory Sweating

To determine the association between location of primary hyperhidrosis (PHH), level of sympathectomy and CS, we performed the Chi-Square and Fisher Exact test. There were no significant associations between both variables and CS ( Table 7).

**Table 7.** Association between location of primary hyperhidrosis (PHH), level of sympathectomy and compensatory sweating in 118 patients following endoscopic thoracic sympathectomy.

Variables		CS		χ ^2^ (df)	P *
		Yes	No		
Location of primary hyperhidrosis	Hands + Feet	38 (55.1)	26 (39.1)	0.143 (2) ^a^	0.954
	Hands + Feet + Axilla	27 (39.1)	20 (40.8)		
	Face+ Hands + Feet+ Axilla	4 (5.8)	3 (6.1)		
Level of sympathectomy	T2–T3	43 (62.3)	23(46.4)	3.188 (2) ^a^	0.198
	T3–T4	2 (2.9)	1 (2.0)		
	T2, T3 & T4	24 (34.8)	25 (51.0)		

χ ^2^ = Chi-square value, df=degree of freedom, ^a^Fisher exact test, *Significant at *p*<0.05

### Predictors of compensatory sweating

In univariate logistic regression analysis, potential predictors of CS with *p* values of less than 0.25 were selected for entry into the multivariate analysis after ruling out multicollinearity and interaction. These independent variables were medical issues (Yes/No) ( *p*=0.219), level of sympathectomy (T2-T3, and T2-T4) ( *p*=0.044), and frequency of follow-up (one month and more than one month) ( *p*=0.000). In the multivariate binary logistic regression analyses, a preliminary final model was determined using the stepwise method and contains only two independent variables: level of sympathectomy ( *p*=0.020) and frequency of follow-up ( *p*=0.000). The full model containing both predictors was statistically significant, χ ^2^ (2, N=118) = 31.21, *p*<0.001, indicating that the model was able to distinguish between subjects who reported and did not report CS. The model as a whole explained between 23.8% (Cox and Snell R square) and 32.0% (Nagelkerke R square) of the variance in CS and correctly classified 69.6% of cases. Model fit assessment using the Hosmer-Lemeshow test indicated that the model was a good model fit to the data (p=0.935). The classification table indicated that the model has attained a good fit to the data (70% of subjects were correctly classified by the model). The AUC of the ROC curve was 0.771 (95%CI 0.686-0.855), which is higher than the cut-off value of 0.7, and indicated that the model could adequately discriminate between those subjects reporting or not reporting CS. The final model is presented in Table 8. Subjects who had undergone ETS at the level of T2-T3 were almost three times more likely than those who had ETS at the level of T2-T4 to report CS (adjusted OR=2.869, 95%CI 1.177-6.991, *p*=0.020). Subjects who had more than one follow-up after ETS were more than thirteen times likely than those having only one follow-up to report CS (adjusted OR=13.558, 95%CI 4.174-44.038, *p*<0.001).

**Table 8.** Predictors of compensatory sweating (CS) in 118 subjects after endoscopic thoracic sympathectomy for primary hyperhidrosis at the National Heart Institute, Kuala Lumpur.

Variables in the Equation									
		B	S.E.	Wald	df	P value	OR	95% CI	
								Lower	Upper
Step 1 ^a^	Sympathectomy level	1.054	0.454	5.378	1	0.020	2.869	1.177	6.991
	Follow-up	2.607	0.601	18.813	1	0.000	13.558	4.174	44.038
	Constant	-0.941	0.378	6.198	1	0.013	0.390		

df=degree of freedom, Wald= Wald Chi Squared value, B=Beta value a. Variable(s) entered on step 1: Sympathectomy level, Follow-up Cox & Snell R Square=0.238; Nagelkerke R Square=0.320 Classification Table: Percentage correctly predicting CS=69.6%

Endoscopic thoracic sympathectomy data Click here for additional data file. Copyright: © 2018 Musa AF et al. 2018 Data associated with the article are available under the terms of the Creative Commons Zero "No rights reserved" data waiver (CC0 1.0 Public domain dedication).

Endoscopic thoracic sympathectomy statistical analysis output file Click here for additional data file. Copyright: © 2018 Musa AF et al. 2018 Data associated with the article are available under the terms of the Creative Commons Zero "No rights reserved" data waiver (CC0 1.0 Public domain dedication).

## Discussion

The success rate of ETS for primary hyperhidrosis and the rate of CS are often used to measure how effective ETS is for primary hyperhidrosis in a particular center. Having a high rate of success and also a high rate of CS decreases the overall satisfaction of the patients after surgery. That being said, CS which is severe in nature is the worse outcome we would expect post-ETS. Most patients reported to be happy with having mild CS compared to primary hyperhidrosis on their palm ^42^.

The strength of our study focuses on the relatively large sample size with a good multiracial distribution among the major races in Malaysia compared to other studies internationally. It is also important to highlight that this is the first study of its kind in Malaysia that provides an excellent background of the success rate and occurrence rate of CS that could spearhead many future studies around primary hyperhidrosis and its treatment. But our study had slightly more males seeking ETS as a cure for primary hyperhidrosis compared to females (57% males versus 42% females). This differs from what many authors have reported as the majority of the papers ^1, 2, 5, 43, 44^ found that the incidence of primary hyperhidrosis in men and women are the same. Most of our patients who had ETS were Malays (79.7%) which tallies with our ethic predominance in Malaysia, as Malay race is the majority in Malaysia. Our analysis also shows that age, gender or BMI did not affect the rate of CS in these patients. A well-known study investigating factors affecting the outcome following ETS by Jaffer *et al*. ^45^ supports this notion as their findings also revealed that age, sex, and BMI did not affect the outcome of the surgery or the rate of CS.

In our study, the level of sympathectomy that was mainly used in this sample was T2-T3 that accounted for 55.9% of the surgeries followed by the T2, T3, and T4 level, which accounted for 41.5% of the surgeries. There were very little variations to the technique of sympathectomy between these two groups as both groups had their surgeries in a single center. Earlier in 1994, Herbst *et al*. ^46^ described the CS rate of 67.4% in T2-T4 resection at his center. However, Scognamillo *et al*. ^47^ reported that there were no differences between T2-T4 and T3-T4 sympathectomy in terms of efficacy and CS rates, the sample size for the T3-T4 group in our study was too small to be used as comparison for other two large groups. We had only three patients who underwent sympathectomy at levels T3-T4.

The mean duration of surgery in our center was 46.6 ±14.29 minutes. The duration of surgery varies in many studies but Ong *et al*. ^48^ from our neighboring country, have reported that their mean duration of surgery was 42 minutes. The duration of surgery has dramatically reduced from the time ETS was introduced in 1930s. Impressively, the mean duration of stay in the hospital in our sample was only 3.5 ± 1.05 days. The data was skewed slightly by nine patients who had long duration of hospital stay due to postoperative complications.

In our analysis, 98.3% of patients had complete relief of palmar hyperhidrosis post-ETS. The reported success rates for ETS in curing palmar hyperhidrosis around the world ranges between 94 to 100% with minimal recurrence rates ^37, 49– 51^. The degree of symptom resolution varied significantly according to regions affected by hyperhidrosis. The main indication for ETS in our group of patients was palmar hyperhidrosis. We noted that 42 out of 54 patients who also had axillary hyperhidrosis had partial reduction (60–90%) in their symptoms. Besides that, 74 out of 100 patients who also had plantar hyperhidrosis on top of their palmar hyperhidrosis had partial reduction (60–90%) in their symptoms as well. The success rates in treating axillary and plantar hyperhidrosis were reported to be lower, with higher failure rates compared to having ETS performed to treat palmar hyperhidrosis ^46, 52^. We can conclude that ETS does not only cure palmar hyperhidrosis, but also reduces plantar and axillary hyperhidrosis. Although techniques and methods used to interrupt these chains vary between studies, if the correct level is interrupted, the results can be mimicked and this is indicated from the excellent success rate in our center.

The primary aim of this procedure was to improve the patients’ quality of life; hence, the complications should be minimal and essentially reduced. As expected, the most common post-operative complication in our study was CS. In our center, the CS rate is lower than average reported rate, 58.5%. Out of the 58.5% patient who had CS, the majority of them (30.5%) had mild form of CS. Most of our patients considered CS as a minor drawback, which is much more tolerable compared to their former condition. Only eleven patients (9.3%) who had the severe form CS regretted having the surgery. Twenty-two patients (18.6%) developed moderate CS in our study; but we were unable to assess how it affects them compared to their initial condition. It would be interesting to have longer follow-up to find out the progression of CS and also quality of life. Most of our patient had single follow-up, hence, we were unable to comment on the follow-up progression of CS. It is a common assumption that CS vanishes over time, but based on recent literature, this assertion is probably wrong. Herbst *et al*. ^46^ discovered that after 14 years of follow up, 67% of their patients still had CS. A 16-year follow-up in 2013 by Askari and colleagues ^53^ revealed that 97.6% of the patients from the study still had CS.

We would also like to believe our CS rates would be lower if not confounded by our tropical climate in Malaysia, where the temperature is high throughout the year. This would lead to greater sweating, exaggerating the incidence of CS in patients. A paper published by Li *et al.* ^54^ concluded that climate plays a significant role in incidences of CS. They looked at patients who are from a hotter climate such as Taiwan and the reported CS rates are as high as 98%. Gassot *et al*. ^23^ also quoted this phenomenon in their paper. Among the areas affected by CS, the back was affected the most in our sample size. CS occurring at the back totaled to 29% out of 58.5% patient who had CS in our center (29%, n= 20). Back of thighs and back involvement was the second highest reported site for CS in our patients (18.8%, n=13) followed by the abdomen (13%, n=9). In our study, we can conclude that the back and trunk were the most common regions being affected by CS. A few authors ^34, 52^ have described that the most common affected region by CS was the posterior aspect of the trunk.

Apart from CS, 10.1% of patients from our center developed other post-operative complications. Seven patients or 5.9% of patients developed pneumothorax postoperatively in our study. Its incidences have been reported to be around 1% to 6% in most reports from the literature ^1^. Four patients (3.4%) had severe pain requiring additional analgesia post operatively. One patient developed post sympathetic neuralgia and is still on follow up regularly. One case had permanent bradycardia post ETS which is said to be a rare complication of ETS. Its occurrence has been reported in the literature ^46, 55^ and certain patients may also need pacemakers if their resting heartbeat goes below 50 beats per minute.

Having said this, it has to be stressed again that it was still CS that has the greatest impact on the quality of life after ETS. Although the pathophysiology of CS remains unknown, surgeons have been trying to reduce the incidence by lowering the level of sympathectomy ^31, 56^. Other surgeons have suggested that lowering the level of sympathectomy could reduce severe CS ^57, 58^. A recent meta-analysis conducted by Cai SW *et al*. ^59^, who claimed to be the first group that conducted a meta-analysis of randomized controlled trials (RCT) on ETS to assess the effect of whether lowering the level of sympathectomy could really reduce CS, confirmed the fact that lowering the level of sympathectomy could reduce CS and severe CS after ETS.

Our study has also showed a similar finding that basically lowering the level of sympathectomy, T2-T4, reduces the incidence of CS significantly. This has been the main predictor in our study. We also noticed that those who came for follow-up for more than once were the ones with CS. It is likely that the persistence of CS troubled the patients leading to the further follow-ups, though further study to look at the impact of ETS on the quality of life of the patients is required.

### Limitations

This present study has two limitations. First, it was a retrospective cohort study based on a proforma ( Supplementary File 1) rather than a tele-interview or medical evaluation. Being an observational study, temporal relationship was unable to be established for a few variables, especially the follow-up data. There is also lack of clarity in some of the follow-up notes, which could have aided the study in providing more information regarding the nature of the CS. Nevertheless, this type of data collection has the advantage that it permits patients to provide self-reported data in a setting that is free from medical influence. Certain data regarding the period that the patient noticed the cessation of palmar hyperhidrosis, onset of CS, progression of CS; and triggers to CS were not available or poorly documented.

Secondly, the direct involvement of the investigator in gathering data could entail the possibility of observer bias. Measures to reduce bias were taken beforehand in order to negate the influence of this bias, specifically the project leader was not involved in data collection. Another limitation would be the difficulty to objectively measure the outcome. Responses gathered from the patient during follow-up are subjective in nature and solely depended on the description of the patient and the surgeon’s perception of these descriptions.

### Future directions

Moving forward, to have a more substantiated and quality data regarding the success rate and incidence of CS and their locations, a larger prospective cohort study with a long-term follow-up in multiple centers would be ideal. A thorough criterion of describing CS or resolution of symptoms should be set universally among all centers to allow for more unified and harmonious data comparison. This would eliminate misreporting and decreasing the wide gap in documenting the incidences of CS. Patients should be followed up at least for five years to gather data on the recurrence of hyperhidrosis after CS in our climate.

There should be an increase in awareness among healthcare providers regarding primary hyperhidrosis and ETS as the most important curative method for it. The high success rate and low incidence of CS should be taken into account and be offered to patients who are suffering from this dreadful condition. The local health ministry should modify the management guidelines and protocols of primary hyperhidrosis to reflect the findings of this study as this the first ever study to report the success rate of ETS for primary hyperhidrosis and incidence of CS in Malaysia.

## Conclusion

We conclude that ETS is a safe, effective and aesthetically remarkable procedure for the treatment of primary hyperhidrosis considering the high success rate with only half of the patients developing CS. Our results compare favourably with or exceed those other studies in terms of success rates and incidences of CS. The clinical implication of this study provides solid evidence to patients or healthcare practitioners by showing excellent safety profile, high success rate and low rates of CS occurrence in patients who had ETS done for primary hyperhidrosis. Taking into account the multiracial population and our climate, we provided answers to patients who were hesitant to undergo ETS at our local setting. We also hope that we managed to inspire researchers to look into this subject of primary hyperhidrosis and ETS as this condition has often been neglected in our society despite the profound impact it has on the daily lives of patients. This study, we believe, has provided the groundwork for higher quality studies in this field especially in this country.

## Data availability

The data referenced by this article are under copyright with the following copyright statement: Copyright: ï¿½ 2018 Musa AF et al.

Data associated with the article are available under the terms of the Creative Commons Zero "No rights reserved" data waiver (CC0 1.0 Public domain dedication). 

Dataset 1: Endoscopic thoracic sympathectomy data 10.5256/f1000research.14777.d203598 ^60^

Dataset 2: Endoscopic thoracic sympathectomy statistical analysis output file 10.5256/f1000research.14777.d203599 ^61^

## Supplementary material

Supplementary File 1 – Proforma used for data collection.
