# Supplementary material for: A retrospective review on minimally invasive technique via endoscopic thoracic sympathectomy (ETS) in the treatment of severe primary hyperhidrosis: Experiences from the National Heart Institute, Malaysia
Source: F1000Res. 2018 May 29;7:670. [Version 1] doi: 10.12688/f1000research.14777.1 (PMC7338916; doi:10.12688/f1000research.14777.1)
Supplement: Endoscopic thoracic sympathectomy statistical analysis output file [file f1000research-7-16079-s0001.tgz › 1f17b5e1-fac2-4d40-89ba-ac3c08600de2_ETS_Output.pdf]

```
 GET
  FILE='C:\Users\rnordin.ADMIN\Downloads\ETS.Data(Complete).sav'.
DATASET NAME DataSet1 WINDOW=FRONT.
SORT CASES BY Compensatory.sweating(A).
SORT CASES BY Compensatory.sweating(D).
DATASET ACTIVATE DataSet1.

SAVE OUTFILE='C:\Users\rnordin.ADMIN\Downloads\ETS.Data(Complete).sav'
  /COMPRESSED.
DATASET ACTIVATE DataSet1.

SAVE OUTFILE='C:\Users\rnordin.ADMIN\Downloads\ETS.Data(Complete).sav'
  /COMPRESSED.

SAVE OUTFILE='C:\Users\rnordin.ADMIN\Desktop\2018\PUBLICATION 2018 ETS\ETS.
Data(Complete).sav '+
    '18APRIL2018.sav'
  /COMPRESSED.
DATASET ACTIVATE DataSet1.

SAVE OUTFILE='C:\Users\rnordin.ADMIN\Desktop\2018\PUBLICATION 2018 ETS\ETS.
Data(Complete).sav '+
    '18APRIL2018.sav'
  /COMPRESSED.
SORT CASES BY Occupation (A).
SORT CASES BY ICU.Stay (A).
SORT CASES BY ICU.Stay (D).
SORT CASES BY Compensatory.sweating(A).
SORT CASES BY Compensatory.sweating(D).
SORT CASES BY Location.of.CS (A).
SORT CASES BY Location.of.CS (D).
SORT CASES BY Duration.surgery(A).
SORT CASES BY Age (A).
SORT CASES BY Age (D).
SORT CASES BY Sex (A).
SORT CASES BY Sex (D).
SORT CASES BY Race (A).
SORT CASES BY Race (D).
DATASET ACTIVATE DataSet1.

SAVE OUTFILE='C:\Users\rnordin.ADMIN\Desktop\2018\PUBLICATION 2018 ETS\ETS.
Data(Complete).sav '+
    '18APRIL2018.sav'
  /COMPRESSED.
SORT CASES BY Marital.Status (A).
SORT CASES BY Marital.Status (D).
DATASET ACTIVATE DataSet1.

SAVE OUTFILE='C:\Users\rnordin.ADMIN\Desktop\2018\PUBLICATION 2018 ETS\ETS.
Data(Complete).sav '+
    '18APRIL2018.sav'
```

```
  /COMPRESSED.
SORT CASES BY Occupation (A).
SORT CASES BY Occupation (D).
DATASET ACTIVATE DataSet1.

SAVE OUTFILE='C:\Users\rnordin.ADMIN\Desktop\2018\PUBLICATION 2018 ETS\ETS.
Data(Complete).sav '+
    '18APRIL2018.sav'
  /COMPRESSED.
DATASET ACTIVATE DataSet1.

SAVE OUTFILE='C:\Users\rnordin.ADMIN\Desktop\2018\PUBLICATION 2018 ETS\ETS.
Data(Complete).sav '+
    '18APRIL2018.sav'
  /COMPRESSED.
SORT CASES BY Occupation2 (A).
DATASET ACTIVATE DataSet1.

SAVE OUTFILE='C:\Users\rnordin.ADMIN\Desktop\2018\PUBLICATION 2018 ETS\ETS.
Data(Complete).sav '+
    '18APRIL2018.sav'
  /COMPRESSED.
DATASET ACTIVATE DataSet1.

SAVE OUTFILE='C:\Users\rnordin.ADMIN\Desktop\2018\PUBLICATION 2018 ETS\ETS.
Data(Complete).sav '+
    '18APRIL2018.sav'
  /COMPRESSED.
COMPUTE BMI=(Weight) / (Height) * (Height).
EXECUTE.
COMPUTE BMI=(Weight) / (Height / 100) * (Height / 100).
EXECUTE.
DATASET ACTIVATE DataSet1.

SAVE OUTFILE='C:\Users\rnordin.ADMIN\Desktop\2018\PUBLICATION 2018 ETS\ETS.
Data(Complete).sav '+
    '18APRIL2018.sav'
  /COMPRESSED.
COMPUTE BMI=(Weight) / (Height /100) * (Height /100).
EXECUTE.
COMPUTE Heightmetre=Height / 100.
EXECUTE.
COMPUTE BMI=(Weight) / (Heightmetre) * (Heightmetre).
EXECUTE.
COMPUTE BMI=(Weight) / (Heightmetre) / (Heightmetre).
EXECUTE.
DATASET ACTIVATE DataSet1.

SAVE OUTFILE='C:\Users\rnordin.ADMIN\Desktop\2018\PUBLICATION 2018 ETS\ETS.
Data(Complete).sav '+
    '18APRIL2018.sav'
  /COMPRESSED.
DATASET ACTIVATE DataSet1.
```

```
SAVE OUTFILE='C:\Users\rnordin.ADMIN\Desktop\2018\PUBLICATION 2018 ETS\ETS.
Data(Complete).sav '+
    '18APRIL2018.sav'
  /COMPRESSED.
SORT CASES BY BMI (A).
DATASET ACTIVATE DataSet1.

SAVE OUTFILE='C:\Users\rnordin.ADMIN\Desktop\2018\PUBLICATION 2018 ETS\ETS.
Data(Complete).sav '+
    '18APRIL2018.sav'
  /COMPRESSED.
SORT CASES BY BMI (A).
SORT CASES BY BMI (D).
DATASET ACTIVATE DataSet1.

SAVE OUTFILE='C:\Users\rnordin.ADMIN\Desktop\2018\PUBLICATION 2018 ETS\ETS.
Data(Complete).sav '+
    '18APRIL2018.sav'
  /COMPRESSED.
DATASET ACTIVATE DataSet1.

SAVE OUTFILE='C:\Users\rnordin.ADMIN\Desktop\2018\PUBLICATION 2018 ETS\ETS.
Data(Complete).sav '+
    '18APRIL2018.sav'
  /COMPRESSED.
DATASET ACTIVATE DataSet1.

SAVE OUTFILE='C:\Users\rnordin.ADMIN\Desktop\2018\PUBLICATION 2018 ETS\ETS.
Data(Complete).sav '+
    '18APRIL2018.sav'
  /COMPRESSED.
SORT CASES BY Weight (A).
SORT CASES BY BMI (A).
SORT CASES BY BMI (D).
SORT CASES BY Thyroid.Function (A).
SORT CASES BY Thyroid.Function (D).
SORT CASES BY Diabetes (A).
SORT CASES BY Location.of.PHH (A).
SORT CASES BY Location.of.PHH (D).
SORT CASES BY Medical.issues (A).
SORT CASES BY Medical.issues (D).
SORT CASES BY Medical.issues (D).
SORT CASES BY Medical.issues (A).
SORT CASES BY Operative.procedure(A).
SORT CASES BY Operative.procedure(D).
SORT CASES BY Patient.position (A).
SORT CASES BY Patient.position (D).
SORT CASES BY Port.size (A).
SORT CASES BY CO2.usage (A).
SORT CASES BY CO2.usage (D).
SORT CASES BY CO2.usage (A).
SORT CASES BY CO2.usage (D).
```

```
SORT CASES BY Level.of.Sympathectomy(A).
SORT CASES BY Level.of.Sympathectomy(D).
SORT CASES BY Level.of.Sympathectomy(A).
DATASET ACTIVATE DataSet1.

SAVE OUTFILE='C:\Users\rnordin.ADMIN\Desktop\2018\PUBLICATION 2018 ETS\ETS.
Data(Complete).sav '+
    '18APRIL2018.sav'
  /COMPRESSED.
DATASET ACTIVATE DataSet1.

SAVE OUTFILE='C:\Users\rnordin.ADMIN\Desktop\2018\PUBLICATION 2018 ETS\ETS.
Data(Complete).sav '+
    '18APRIL2018.sav'
  /COMPRESSED.
SORT CASES BY Method.of.excision(A).
SORT CASES BY Method.of.excision(D).
SORT CASES BY Histopathology.sent(A).
SORT CASES BY Histopathology.sent(D).
SORT CASES BY Duration.surgery(A).
SORT CASES BY Duration.surgery(D).
SORT CASES BY Duration.surgery(A).
SORT CASES BY Conversion.to.open.surgery(A).
SORT CASES BY Conversion.to.open.surgery(D).
SORT CASES BY Complications(A).
DATASET ACTIVATE DataSet1.

SAVE OUTFILE='C:\Users\rnordin.ADMIN\Desktop\2018\PUBLICATION 2018 ETS\ETS.
Data(Complete).sav '+
    '18APRIL2018.sav'
  /COMPRESSED.
SORT CASES BY Follow.up(A).
SORT CASES BY Follow.up(D).
SORT CASES BY Number.of.follow.up(A).
SORT CASES BY Number.of.follow.up(D).
SORT CASES BY Number.of.follow.up(A).
SORT CASES BY Number.of.follow.up(D).
DATASET ACTIVATE DataSet1.

SAVE OUTFILE='C:\Users\rnordin.ADMIN\Desktop\2018\PUBLICATION 2018 ETS\ETS.
Data(Complete).sav '+
    '18APRIL2018.sav'
  /COMPRESSED.
DATASET ACTIVATE DataSet1.

SAVE OUTFILE='C:\Users\rnordin.ADMIN\Desktop\2018\PUBLICATION 2018 ETS\ETS.
Data(Complete).sav '+
    '18APRIL2018.sav'
  /COMPRESSED.
DATASET ACTIVATE DataSet1.

SAVE OUTFILE='C:\Users\rnordin.ADMIN\Desktop\2018\PUBLICATION 2018 ETS\ETS.
Data(Complete).sav '+
```

```
    '18APRIL2018.sav'
  /COMPRESSED.
SORT CASES BY Issues (A).
SORT CASES BY Issues (D).
SORT CASES BY Compensatory.sweating(A).
SORT CASES BY Compensatory.sweating(D).
DATASET ACTIVATE DataSet1.

SAVE OUTFILE='C:\Users\rnordin.ADMIN\Desktop\2018\PUBLICATION 2018 ETS\ETS.
Data(Complete).sav '+
    '18APRIL2018.sav'
  /COMPRESSED.
SORT CASES BY Compensatory.sweating(A).
SORT CASES BY Compensatory.sweating(D).
SORT CASES BY When.noticed (A).
SORT CASES BY When.noticed (D).
SORT CASES BY When.noticed (D).
SORT CASES BY When.noticed (A).
DATASET ACTIVATE DataSet1.

SAVE OUTFILE='C:\Users\rnordin.ADMIN\Desktop\2018\PUBLICATION 2018 ETS\ETS.
Data(Complete).sav '+
    '18APRIL2018.sav'
  /COMPRESSED.
SORT CASES BY When.noticed (A).
SORT CASES BY When.noticed (D).
SORT CASES BY Severity (A).
SORT CASES BY Location.of.CS (A).
DATASET ACTIVATE DataSet1.

SAVE OUTFILE='C:\Users\rnordin.ADMIN\Desktop\2018\PUBLICATION 2018 ETS\ETS.
Data(Complete).sav '+
    '18APRIL2018.sav'
  /COMPRESSED.
SORT CASES BY follow.up.progression(A).
DATASET ACTIVATE DataSet1.

SAVE OUTFILE='C:\Users\rnordin.ADMIN\Desktop\2018\PUBLICATION 2018 ETS\ETS.
Data(Complete).sav '+
    '18APRIL2018.sav'
  /COMPRESSED.
SORT CASES BY Reduction.of.PH (A).
SORT CASES BY Reduction.of.PH (D).
DATASET ACTIVATE DataSet1.

SAVE OUTFILE='C:\Users\rnordin.ADMIN\Desktop\2018\PUBLICATION 2018 ETS\ETS.
Data(Complete).sav '+
    '18APRIL2018.sav'
  /COMPRESSED.
SORT CASES BY Age (A).
SORT CASES BY Age (D).
SORT CASES BY Age (A).
DATASET ACTIVATE DataSet1.
```

```
SAVE OUTFILE='C:\Users\rnordin.ADMIN\Desktop\2018\PUBLICATION 2018 ETS\ETS.
Data(Complete).sav '+
    '18APRIL2018.sav'
  /COMPRESSED.
FREQUENCIES VARIABLES=Age Duration.surgery ICU.Stay Hospital.stay
  /FORMAT=NOTABLE
  /PERCENTILES=25.0 75.0
  /STATISTICS=STDDEV MINIMUM MAXIMUM MEAN MEDIAN SKEWNESS SESKEW KURTOSIS S
EKURT
  /ORDER=ANALYSIS.
```

Frequencies

Notes

Output Created		18-APR-2018 17:09:...
Comments		
Input	Data	C:\Users\rnordin.ADMIN\Desktop\2018\PUBLICATION 2018 ETS\ETS.Data(Complete).sav 18APRIL2018.sav
	Active Dataset	DataSet1
	Filter	<none>
	Weight	<none>
	Split File	<none>
	N of Rows in Working Data File	118
Missing Value Handling	Definition of Missing	User-defined missing values are treated as missing.
	Cases Used	Statistics are based on all cases with valid data.
Syntax		FREQUENCIES VARIABLES=Age Duration.surgery ICU.Stay Hospital.stay /FORMAT=NOTABLE /PERCENTILES=25.0 75.0 /STATISTICS=STDDEV MINIMUM MAXIMUM MEAN MEDIAN SKEWNESS SESKEW KURTOSIS SEKURT /ORDER=ANALYSIS.
Resources	Processor Time	00:00:00.02
	Elapsed Time	00:00:00.13

```
[DataSet1] C:\Users\rnordin.ADMIN\Desktop\2018\PUBLICATION 2018 ETS\ETS.Dat
a(Complete).sav 18APRIL2018.sav
```

Statistics

		Age	Duration. surgery	ICU.Stay	Hospital.stay
N	Valid	118	118	118	118
	Missing	0	0	0	0
Mean		22.91	46.6102	1.9661	3.5763
Median		21.00	45.0000	2.0000	3.0000
Std. Deviation		7.262	14.28707	.18174	1.04927
Skewness		1.201	1.083	-5.218	1.827
Std. Error of Skewness		.223	.223	.223	.223
Kurtosis		2.205	2.280	25.660	5.909
Std. Error of Kurtosis		.442	.442	.442	.442
Minimum		9	20.00	1.00	1.00
Maximum		52	105.00	2.00	9.00
Percentiles	25	18.00	35.0000	2.0000	3.0000
	75	26.00	55.0000	2.0000	4.0000

```
 DATASET ACTIVATE DataSet1.

SAVE OUTFILE='C:\Users\rnordin.ADMIN\Desktop\2018\PUBLICATION 2018 ETS\ETS.
Data(Complete).sav '+
    '18APRIL2018.sav'
  /COMPRESSED.
SORT CASES BY Sex (A).
DATASET ACTIVATE DataSet1.

SAVE OUTFILE='C:\Users\rnordin.ADMIN\Desktop\2018\PUBLICATION 2018 ETS\ETS.
Data(Complete).sav '+
    '18APRIL2018.sav'
  /COMPRESSED.
SORT CASES BY Race (A).
DATASET ACTIVATE DataSet1.

SAVE OUTFILE='C:\Users\rnordin.ADMIN\Desktop\2018\PUBLICATION 2018 ETS\ETS.
Data(Complete).sav '+
    '18APRIL2018.sav'
  /COMPRESSED.
SORT CASES BY Marital.Status (A).
SORT CASES BY Marital.Status (D).
SORT CASES BY Marital.Status (A).
DATASET ACTIVATE DataSet1.

SAVE OUTFILE='C:\Users\rnordin.ADMIN\Desktop\2018\PUBLICATION 2018 ETS\ETS.
Data(Complete).sav '+
    '18APRIL2018.sav'
  /COMPRESSED.
DATASET ACTIVATE DataSet1.
```

```
SAVE OUTFILE='C:\Users\rnordin.ADMIN\Desktop\2018\PUBLICATION 2018 ETS\ETS.
Data(Complete).sav '+
    '18APRIL2018.sav'
  /COMPRESSED.
SORT CASES BY Marital.Status (A).
DATASET ACTIVATE DataSet1.

SAVE OUTFILE='C:\Users\rnordin.ADMIN\Desktop\2018\PUBLICATION 2018 ETS\ETS.
Data(Complete).sav '+
    '18APRIL2018.sav'
  /COMPRESSED.
SORT CASES BY Age (A).
DATASET ACTIVATE DataSet1.

SAVE OUTFILE='C:\Users\rnordin.ADMIN\Desktop\2018\PUBLICATION 2018 ETS\ETS.
Data(Complete).sav '+
    '18APRIL2018.sav'
  /COMPRESSED.
DATASET ACTIVATE DataSet1.

SAVE OUTFILE='C:\Users\rnordin.ADMIN\Desktop\2018\PUBLICATION 2018 ETS\ETS.
Data(Complete).sav '+
    '18APRIL2018.sav'
  /COMPRESSED.
SORT CASES BY Sex (A).
SORT CASES BY Sex (D).
SORT CASES BY Sex (A).
DATASET ACTIVATE DataSet1.

SAVE OUTFILE='C:\Users\rnordin.ADMIN\Desktop\2018\PUBLICATION 2018 ETS\ETS.
Data(Complete).sav '+
    '18APRIL2018.sav'
  /COMPRESSED.
DATASET ACTIVATE DataSet1.

SAVE OUTFILE='C:\Users\rnordin.ADMIN\Desktop\2018\PUBLICATION 2018 ETS\ETS.
Data(Complete).sav '+
    '18APRIL2018.sav'
  /COMPRESSED.
SORT CASES BY Race (A).
DATASET ACTIVATE DataSet1.

SAVE OUTFILE='C:\Users\rnordin.ADMIN\Desktop\2018\PUBLICATION 2018 ETS\ETS.
Data(Complete).sav '+
    '18APRIL2018.sav'
  /COMPRESSED.
SORT CASES BY Marital.Status (A).
SORT CASES BY Marital.Status (D).
SORT CASES BY Marital.Status (A).
DATASET ACTIVATE DataSet1.

SAVE OUTFILE='C:\Users\rnordin.ADMIN\Desktop\2018\PUBLICATION 2018 ETS\ETS.
```

```
Data(Complete).sav '+
    '18APRIL2018.sav'
  /COMPRESSED.
SORT CASES BY Occupation2 (A).
DATASET ACTIVATE DataSet1.

SAVE OUTFILE='C:\Users\rnordin.ADMIN\Desktop\2018\PUBLICATION 2018 ETS\ETS.
Data(Complete).sav '+
    '18APRIL2018.sav'
  /COMPRESSED.
DATASET ACTIVATE DataSet1.

SAVE OUTFILE='C:\Users\rnordin.ADMIN\Desktop\2018\PUBLICATION 2018 ETS\ETS.
Data(Complete).sav '+
    '18APRIL2018.sav'
  /COMPRESSED.
SORT CASES BY BMI (A).
DATASET ACTIVATE DataSet1.

SAVE OUTFILE='C:\Users\rnordin.ADMIN\Desktop\2018\PUBLICATION 2018 ETS\ETS.
Data(Complete).sav '+
    '18APRIL2018.sav'
  /COMPRESSED.
DATASET ACTIVATE DataSet1.

SAVE OUTFILE='C:\Users\rnordin.ADMIN\Desktop\2018\PUBLICATION 2018 ETS\ETS.
Data(Complete).sav '+
    '18APRIL2018.sav'
  /COMPRESSED.
SORT CASES BY Thyroid.Function (A).
SORT CASES BY Thyroid.Function (D).
SORT CASES BY Thyroid.Function (A).
SORT CASES BY Thyroid.Function (D).
DATASET ACTIVATE DataSet1.

SAVE OUTFILE='C:\Users\rnordin.ADMIN\Desktop\2018\PUBLICATION 2018 ETS\ETS.
Data(Complete).sav '+
    '18APRIL2018.sav'
  /COMPRESSED.
SORT CASES BY Diabetes (A).
SORT CASES BY Diabetes (D).
SORT CASES BY Diabetes (A).
SORT CASES BY Location.of.PHH (A).
SORT CASES BY Location.of.PHH (D).
SORT CASES BY Location.of.PHH (A).
SORT CASES BY Medical.issues (A).
SORT CASES BY Medical.issues (A).
DATASET ACTIVATE DataSet1.

SAVE OUTFILE='C:\Users\rnordin.ADMIN\Desktop\2018\PUBLICATION 2018 ETS\ETS.
Data(Complete).sav '+
    '18APRIL2018.sav'
  /COMPRESSED.
```

```
SORT CASES BY Operative.procedure(A).
SORT CASES BY Operative.procedure(D).
SORT CASES BY Patient.position(A).
DATASET ACTIVATE DataSet1.

SAVE OUTFILE='C:\Users\rnordin.ADMIN\Desktop\2018\PUBLICATION 2018 ETS\ETS.
Data(Complete).sav '+
    '18APRIL2018.sav'
  /COMPRESSED.
SORT CASES BY Port.size (A).
SORT CASES BY Port.size (D).
SORT CASES BY CO2.usage (A).
SORT CASES BY CO2.usage (D).
SORT CASES BY Level.of.Sympathectomy(A).
SORT CASES BY Level.of.Sympathectomy(D).
SORT CASES BY Sympathectomy.Level(A).
SORT CASES BY Method.of.excision(A).
SORT CASES BY Method.of.excision(D).
SORT CASES BY Histopathology.sent(A).
SORT CASES BY Histopathology.sent(D).
NPAR TESTS
  /K-S(NORMAL)=Age Hospital.stay ICU.Stay Duration.surgery
  /MISSING ANALYSIS.
```

NPar Tests

Notes

Output Created		18-APR-2018 18:10:...
Comments		
Input	Data	C:\Users\rnordin.ADMIN\Desktop\2018\PUBLICATION 2018 ETS\ETS.Data (Complete).sav 18APRIL2018.sav
	Active Dataset	DataSet1
	Filter	<none>
	Weight	<none>
	Split File	<none>
	N of Rows in Working Data File	118
Missing Value Handling	Definition of Missing	User-defined missing values are treated as missing.
	Cases Used	Statistics for each test are based on all cases with valid data for the variable(s) used in that test.

Notes

Syntax		NPAR TESTS<br>/K-S(NORMAL)=Age Hospital.stay ICU.Stay Duration.surgery<br>/MISSING ANALYSIS.
Resources	Processor Time	00:00:00.00
	Elapsed Time	00:00:00.09
	Number of Cases Allowed[a]	449389

a. Based on availability of workspace memory.

One-Sample Kolmogorov-Smirnov Test

		Age	Hospital.stay	ICU.Stay	Duration. surgery
N		118	118	118	118
Normal Parameters[a,b]	Mean	22.91	3.5763	1.9661	46.6102
	Std. Deviation	7.262	1.04927	.18174	14.28707
Most Extreme Differences	Absolute	.112	.319	.540	.135
	Positive	.112	.319	.426	.135
	Negative	-.087	-.258	-.540	-.073
Test Statistic		.112	.319	.540	.135
Asymp. Sig. (2-tailed)		.001[c]	.000[c]	.000[c]	.000[c]

a. Test distribution is Normal.

b. Calculated from data.

c. Lilliefors Significance Correction.

```
EXAMINE VARIABLES=Age Hospital.stay ICU.Stay Duration.surgery
 /PLOT BOXPLOT STEMLEAF NPPLOT
 /COMPARE GROUPS
 /STATISTICS DESCRIPTIVES
 /CINTERVAL 95
 /MISSING LISTWISE
 /NOTOTAL.
```

Explore

Notes

Output Created		18-APR-2018 18:25:...
Comments		
Input	Data	C:\Users\rnordin.<br>ADMIN\Desktop\2018\<br>PUBLICATION 2018<br>ETS\ETS.Data<br>(Complete).sav<br>18APRIL2018.sav
	Active Dataset	DataSet1
	Filter	<none>
	Weight	<none>
	Split File	<none>
	N of Rows in Working Data File	118
Missing Value Handling	Definition of Missing	User-defined missing values for dependent variables are treated as missing.
	Cases Used	Statistics are based on cases with no missing values for any dependent variable or factor used.
Syntax		EXAMINE<br>VARIABLES=Age<br>Hospital.stay ICU.Stay<br>Duration.surgery<br>/PLOT BOXPLOT<br>STEMLEAF NPPLOT<br>/COMPARE GROUPS<br>/STATISTICS<br>DESCRIPTIVES<br>/CINTERVAL 95<br>/MISSING LISTWISE<br>/NOTOTAL.
Resources	Processor Time	00:00:03.23
	Elapsed Time	00:00:06.48

Case Processing Summary

	Cases					
	Valid		Missing		Total	
	N	Percent	N	Percent	N	Percent
Age	118	100.0%	0	0.0%	118	100.0%
Hospital.stay	118	100.0%	0	0.0%	118	100.0%
ICU.Stay	118	100.0%	0	0.0%	118	100.0%
Duration.surgery	118	100.0%	0	0.0%	118	100.0%

Descriptives

			Statistic	Std. Error
Age	Mean		22.91	.669
	95% Confidence Interval for Mean	Lower Bound	21.58	
		Upper Bound	24.23	
	5% Trimmed Mean		22.38	
	Median		21.00	
	Variance		52.735	
	Std. Deviation		7.262	
	Minimum		9	
	Maximum		52	
	Range		43	
	Interquartile Range		8	
	Skewness		1.201	.223
	Kurtosis		2.205	.442
Hospital.stay	Mean		3.5763	.09659
	95% Confidence Interval for Mean	Lower Bound	3.3850	
		Upper Bound	3.7676	
	5% Trimmed Mean		3.4925	
	Median		3.0000	
	Variance		1.101	
	Std. Deviation		1.04927	
	Minimum		1.00	
	Maximum		9.00	
	Range		8.00	
	Interquartile Range		1.00	
	Skewness		1.827	.223
	Kurtosis		5.909	.442
ICU.Stay	Mean		1.9661	.01673
	95% Confidence Interval for Mean	Lower Bound	1.9330	
		Upper Bound	1.9992	
	5% Trimmed Mean		2.0000	
	Median		2.0000	
	Variance		.033	
	Std. Deviation		.18174	
	Minimum		1.00	
	Maximum		2.00	
	Range		1.00	
	Interquartile Range		.00	
	Skewness		-5.218	.223
	Kurtosis		25.660	.442
Duration.surgery	Mean		46.6102	1.31523

Descriptives

			Statistic	Std. Error
	95% Confidence Interval for Mean	Lower Bound	44.0054	
		Upper Bound	49.2149	
	5% Trimmed Mean		45.7392	
	Median		45.0000	
	Variance		204.120	
	Std. Deviation		14.28707	
	Minimum		20.00	
	Maximum		105.00	
	Range		85.00	
	Interquartile Range		20.00	
	Skewness		1.083	.223
	Kurtosis		2.280	.442

Tests of Normality

	Kolmogorov-Smirnov[a]			Shapiro-Wilk		
	Statistic	df	Sig.	Statistic	df	Sig.
Age	.112	118	.001	.922	118	.000
Hospital.stay	.319	118	.000	.746	118	.000
ICU.Stay	.540	118	.000	.174	118	.000
Duration.surgery	.135	118	.000	.934	118	.000

a. Lilliefors Significance Correction

Age

```
Age Stem-and-Leaf Plot

 Frequency    Stem &  Leaf

     1.00        0 .  9
     1.00        1 .  0
     1.00        1 .  3
    12.00        1 .  444555555555
    13.00        1 .  6667777777777
    15.00        1 .  888888888899999
    17.00        2 .  00000000111111111
     9.00        2 .  222223333
    12.00        2 .  445555555555
    15.00        2 .  666666666777777
     7.00        2 .  8888999
     2.00        3 .  01
     3.00        3 .  222
     3.00        3 .  555
     1.00        3 .  7
     6.00 Extremes    (>=39)
```

```
Stem width:        10
Each leaf:        1 case(s)
```

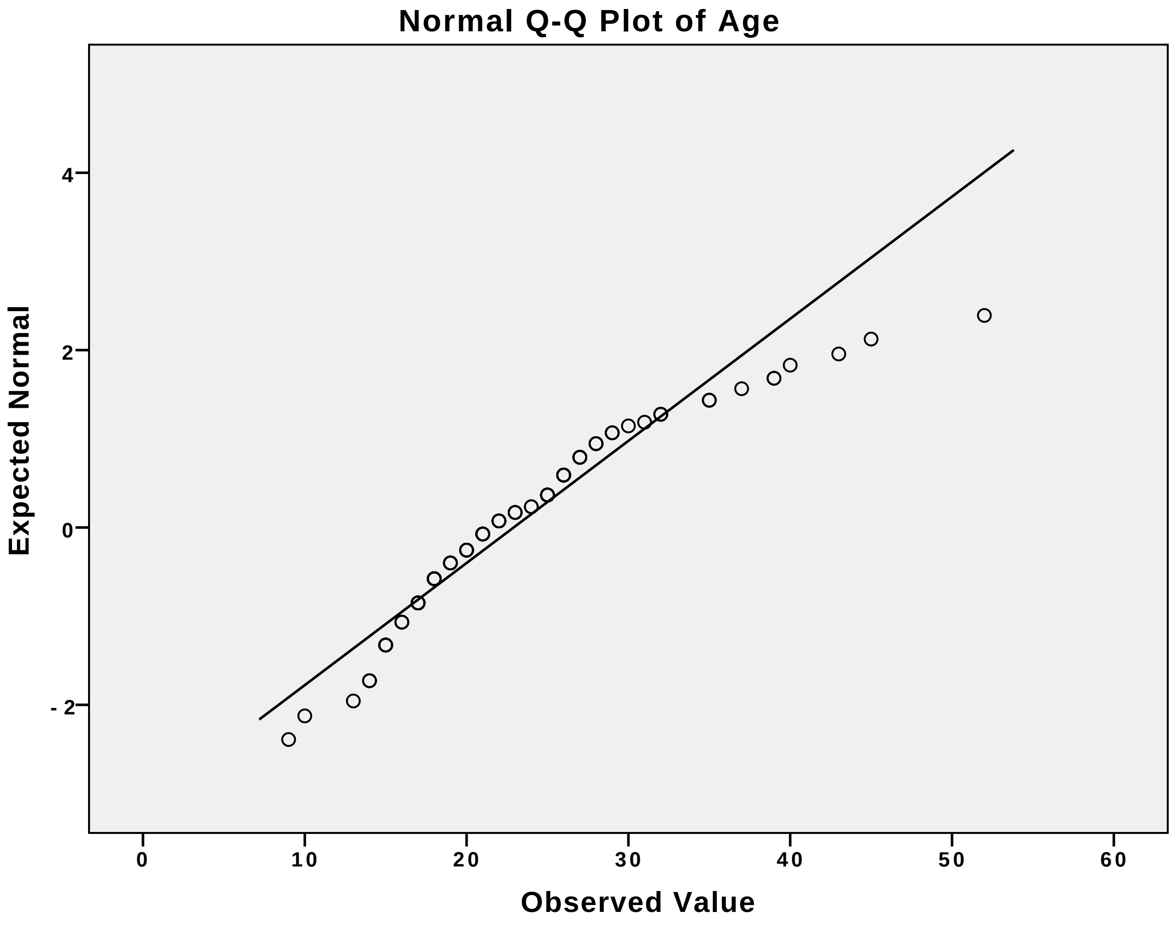

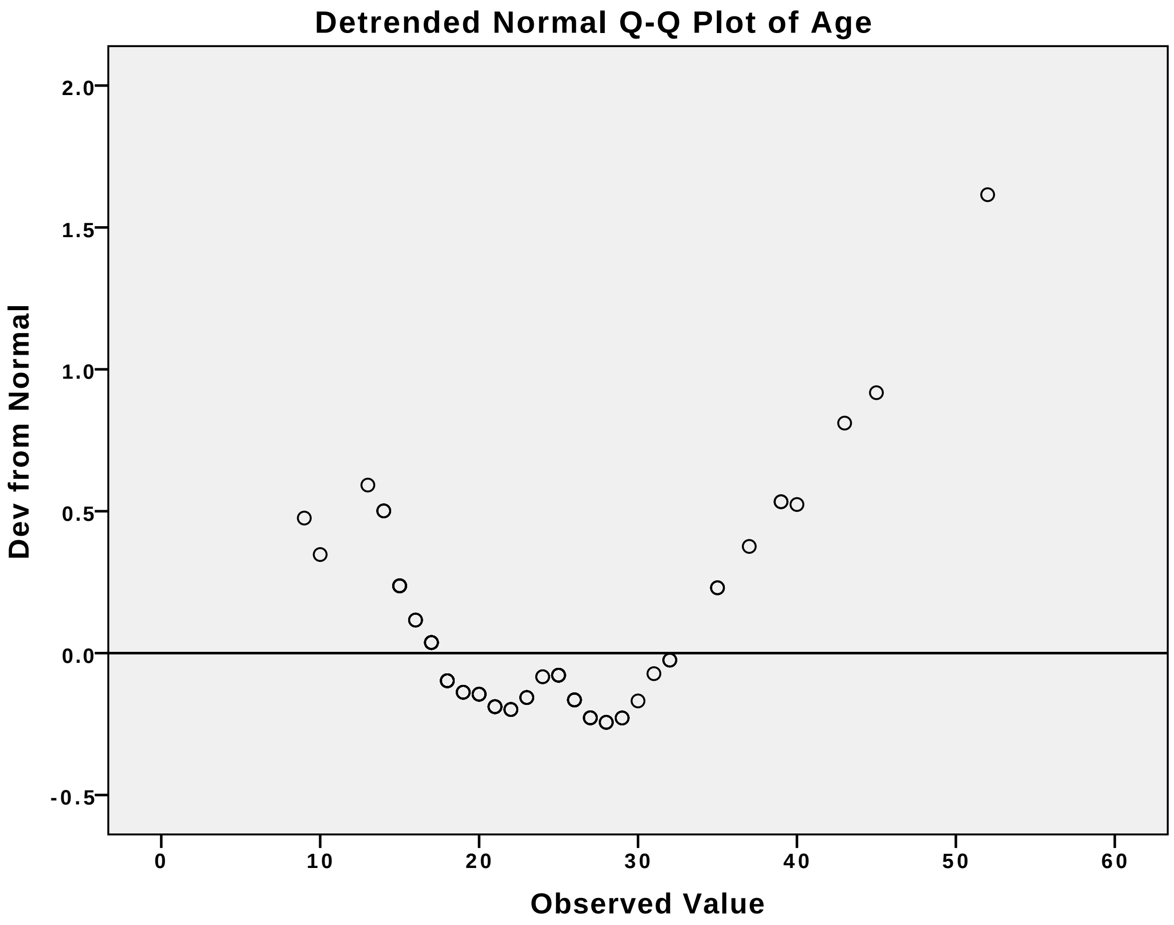

Detrended Normal Q-Q Plot of Age
Dev from Normal
2.0
1.5
1.0
0.5
0.0
-0.5
0
10
20
30
40
50
60
Observed Value

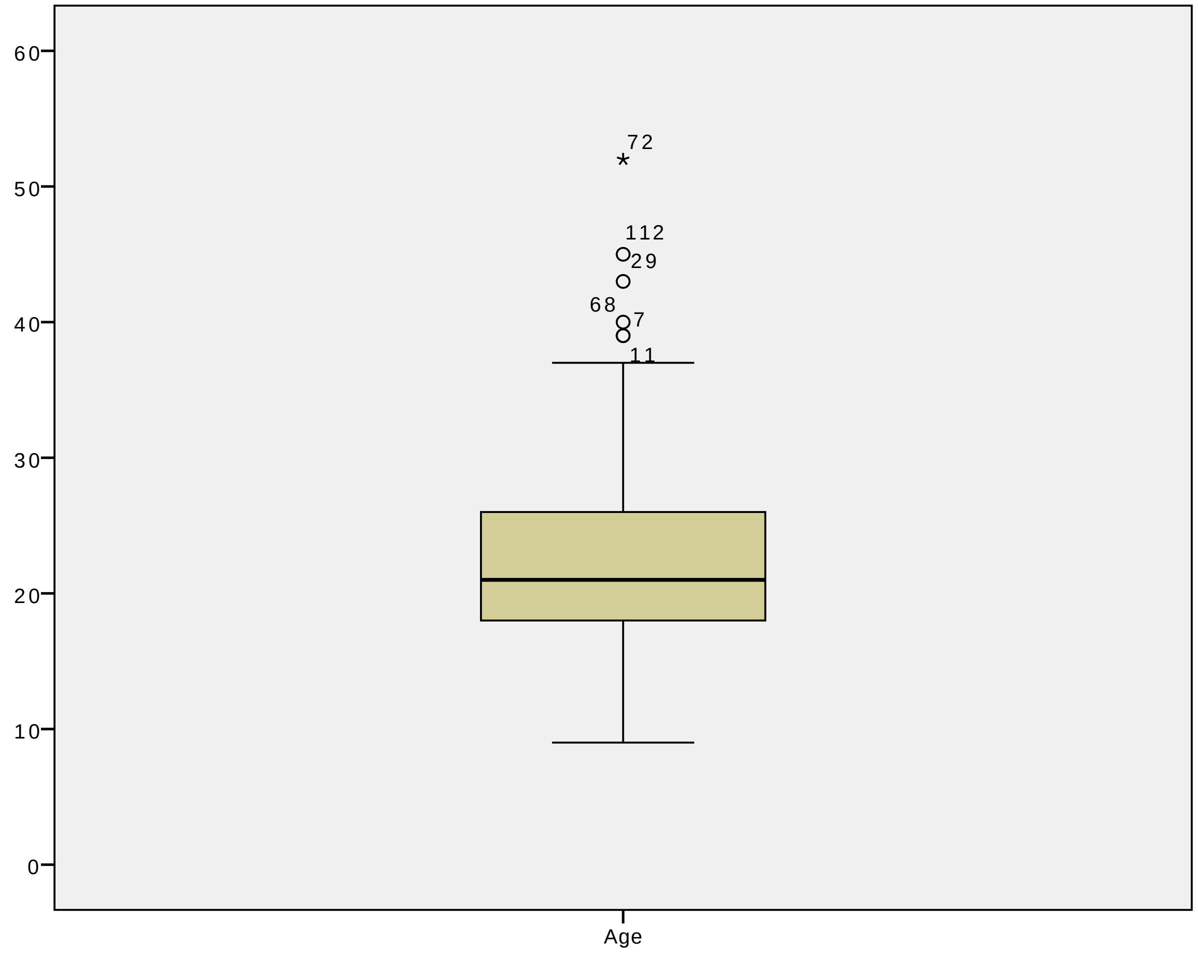

Hospital.stay

```
Hospital.stay Stem-and-Leaf Plot

 Frequency    Stem &  Leaf

     1.00 Extremes    (=<1.0)
     3.00        2 .  000
      .00        2 .
      .00        2 .
      .00        2 .
      .00        2 .
    68.00        3 .  00000000000000000000000000000000000000000000000000000
000000000000000
      .00        3 .
      .00        3 .
      .00        3 .
      .00        3 .
    30.00        4 .  000000000000000000000000000000
      .00        4 .
      .00        4 .
      .00        4 .
      .00        4 .
     8.00        5 .  00000000
```

```
    8.00 Extremes    (>=6.0)

Stem width:      1.00
Each leaf:        1 case(s)
```

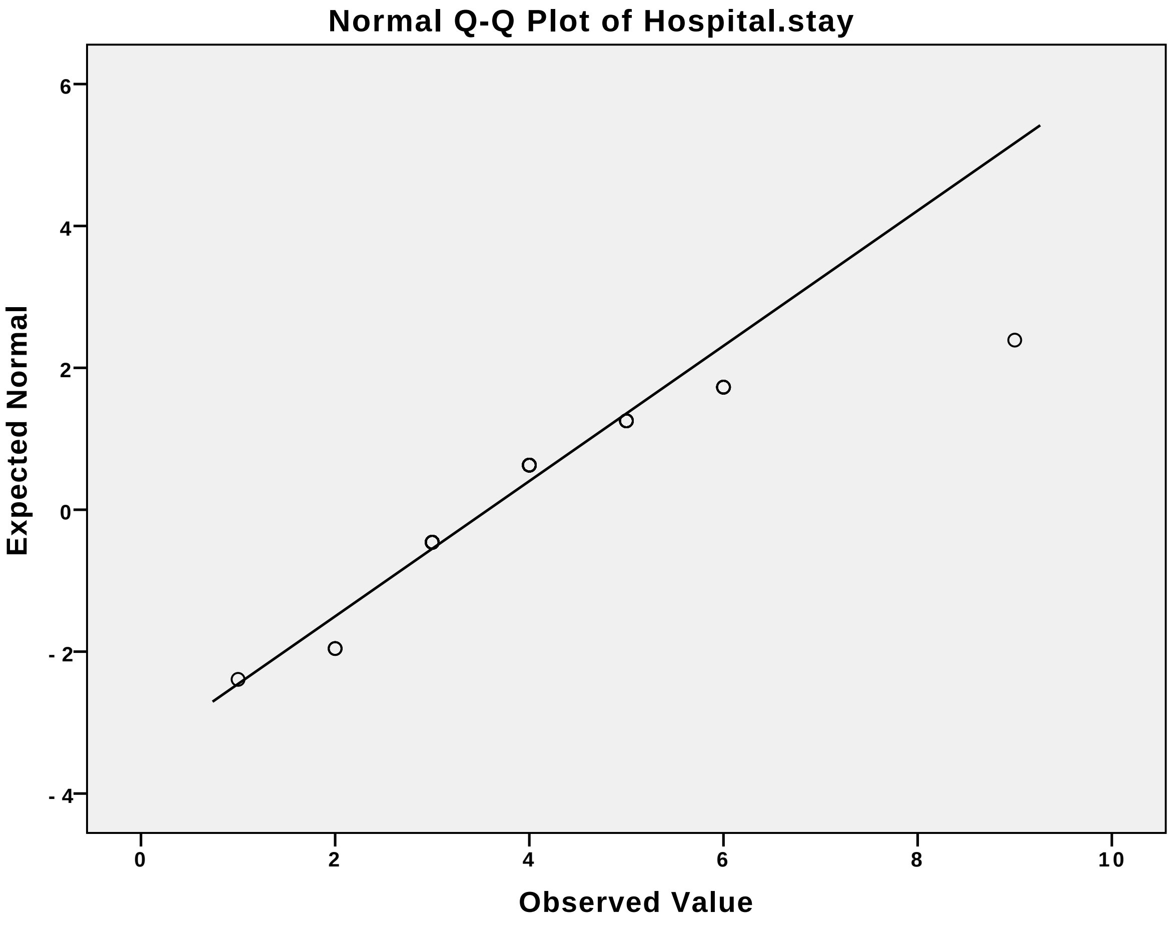

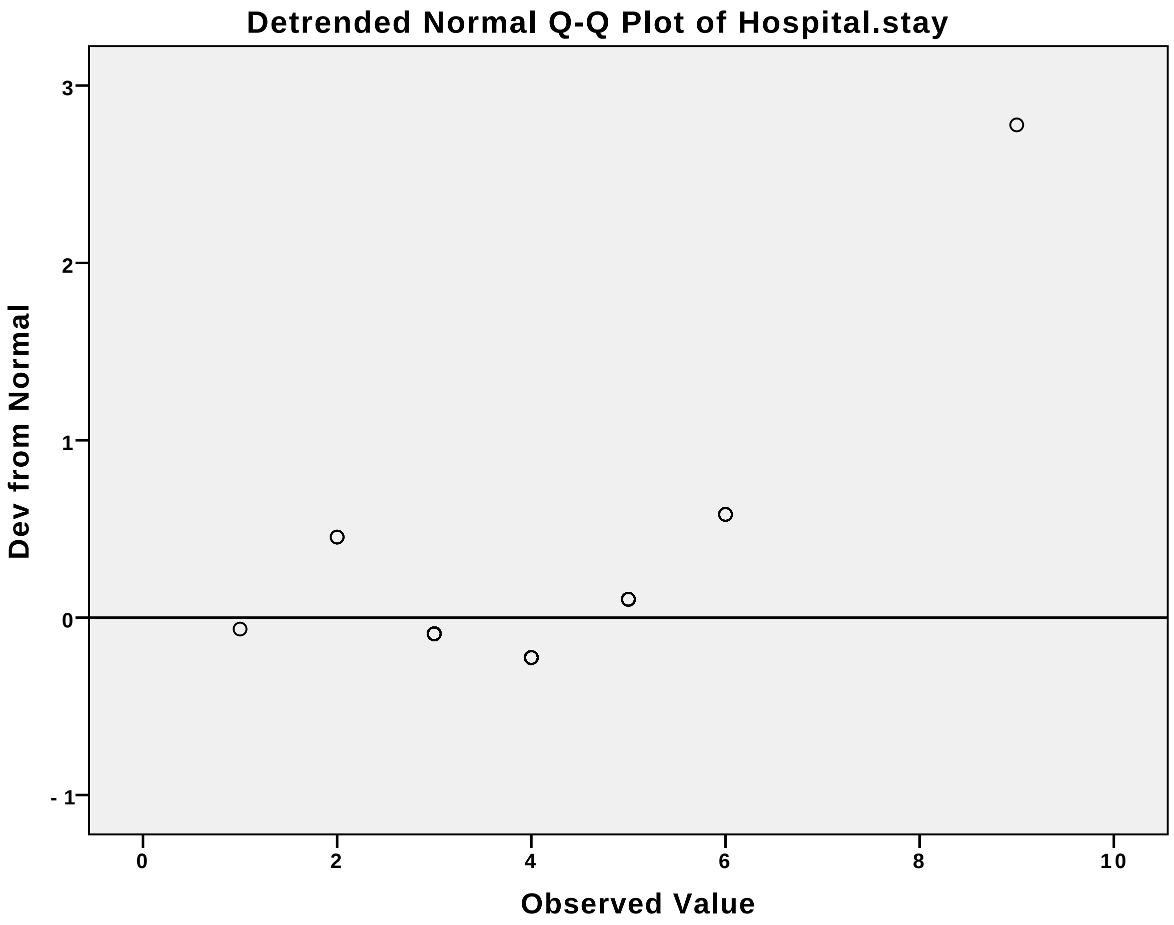

Detrended Normal Q-Q Plot of Hospital.stay
Dev from Normal
Observed Value
3
2
1
0
- 1
0
2
4
6
8
10

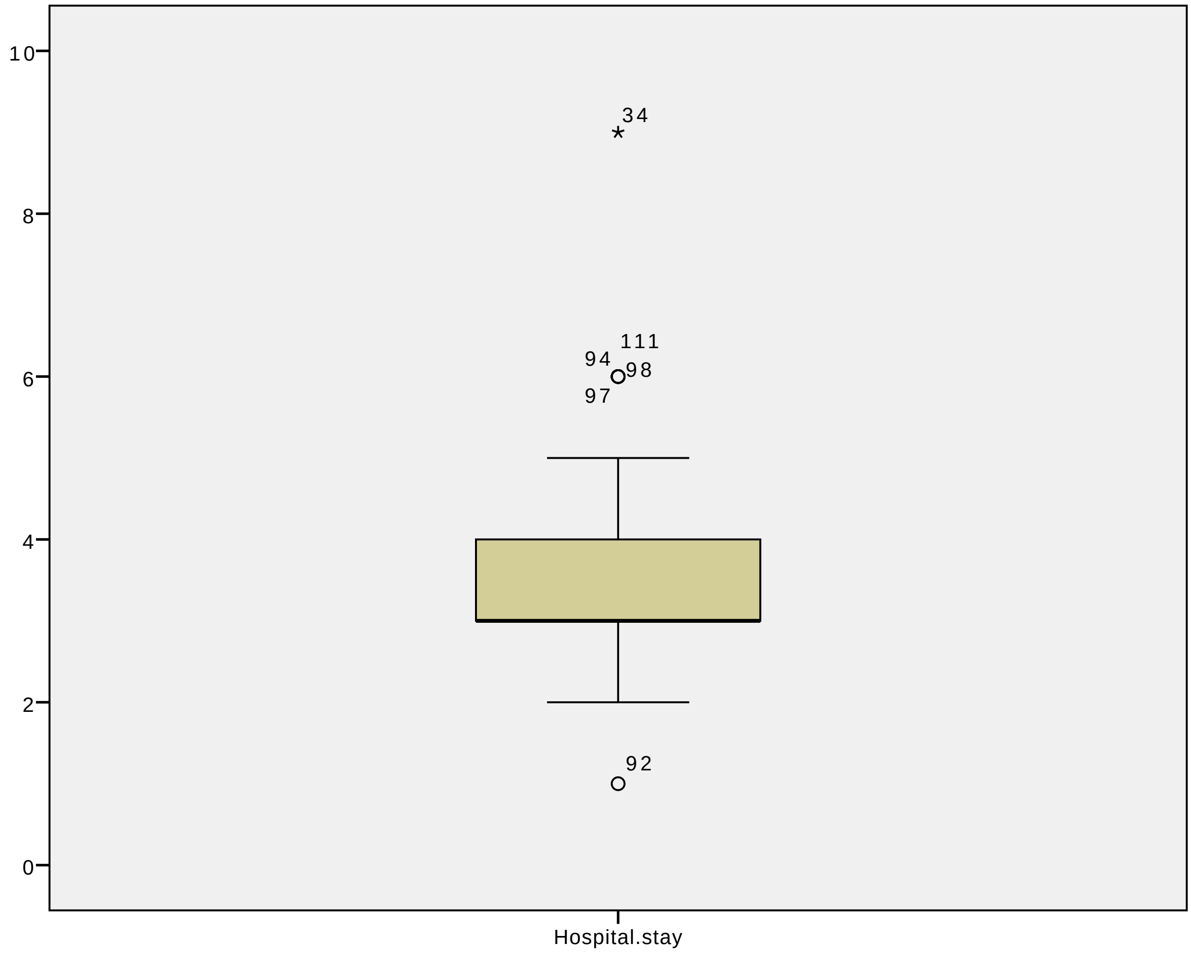

ICU.Stay

```
ICU.Stay Stem-and-Leaf Plot

 Frequency    Stem &  Leaf

     4.00 Extremes    (=<1)
      .00        0 .
   114.00        0 .  22222222222222222222222222222222222222222222222222222
2222

 Stem width:     10.00
 Each leaf:        2 case(s)
```

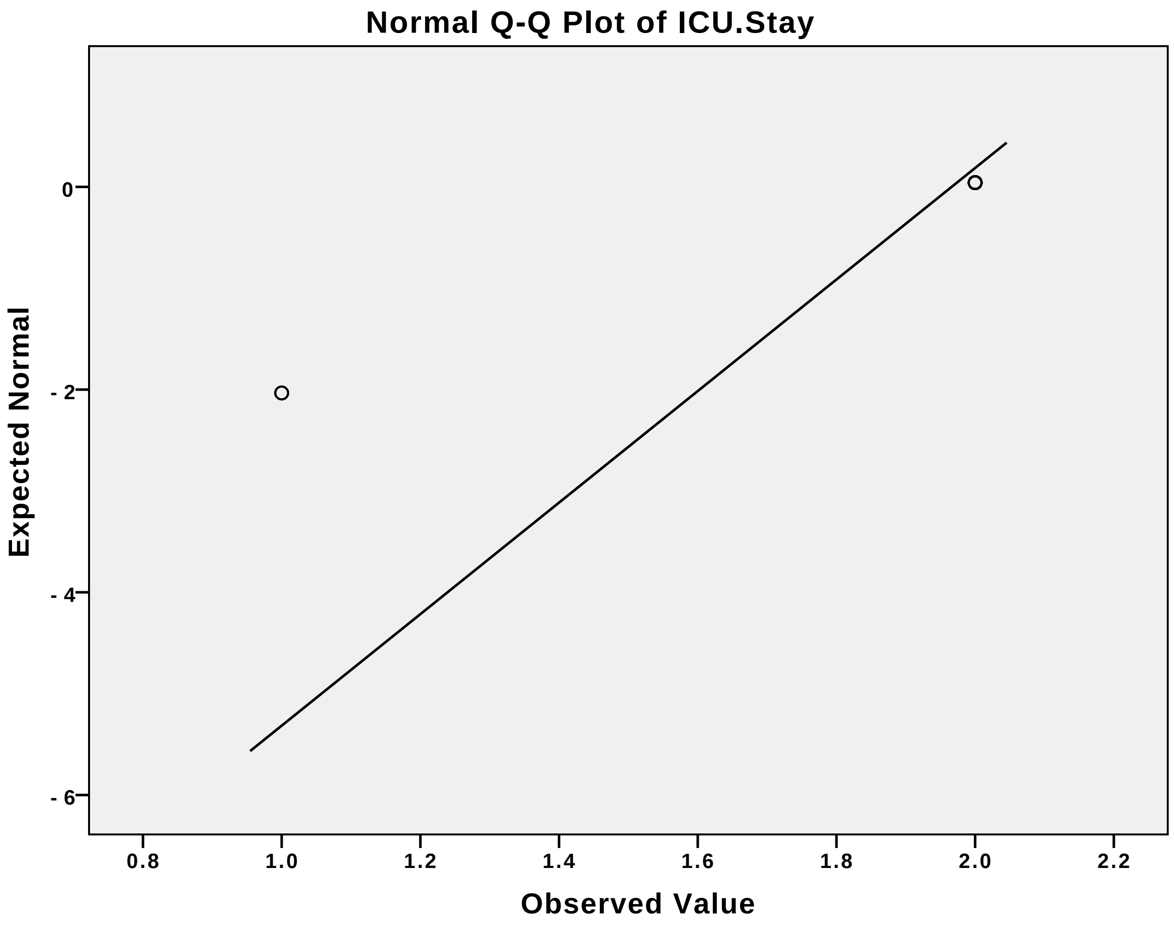
Normal Q-Q Plot of ICU.Stay
Expected Normal
0
-2
-4
-6
0.8
1.0
1.2
1.4
1.6
1.8
2.0
2.2
Observed Value

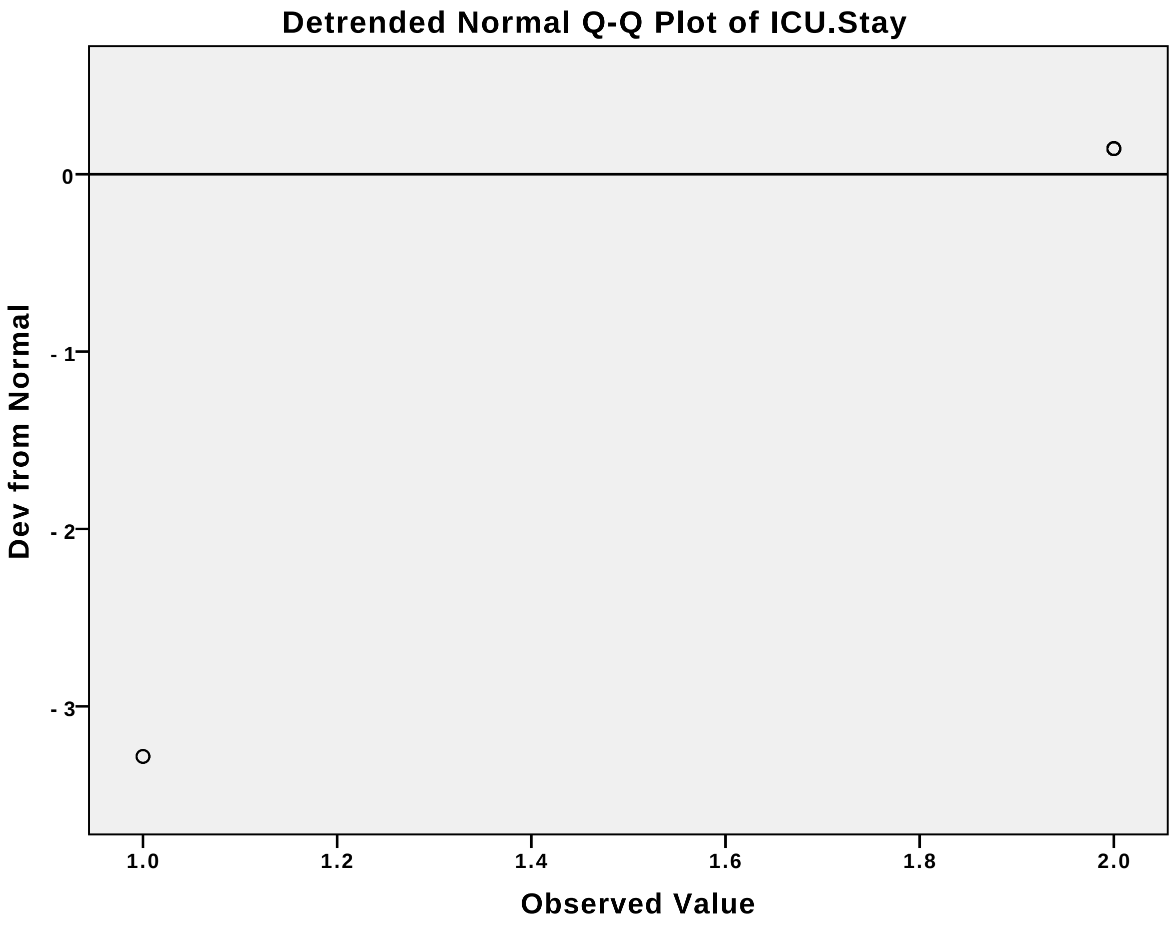
Detrended Normal Q-Q Plot of ICU.Stay
Dev from Normal
0
- 1
- 2
- 3
1.0
1.2
1.4
1.6
1.8
2.0
Observed Value

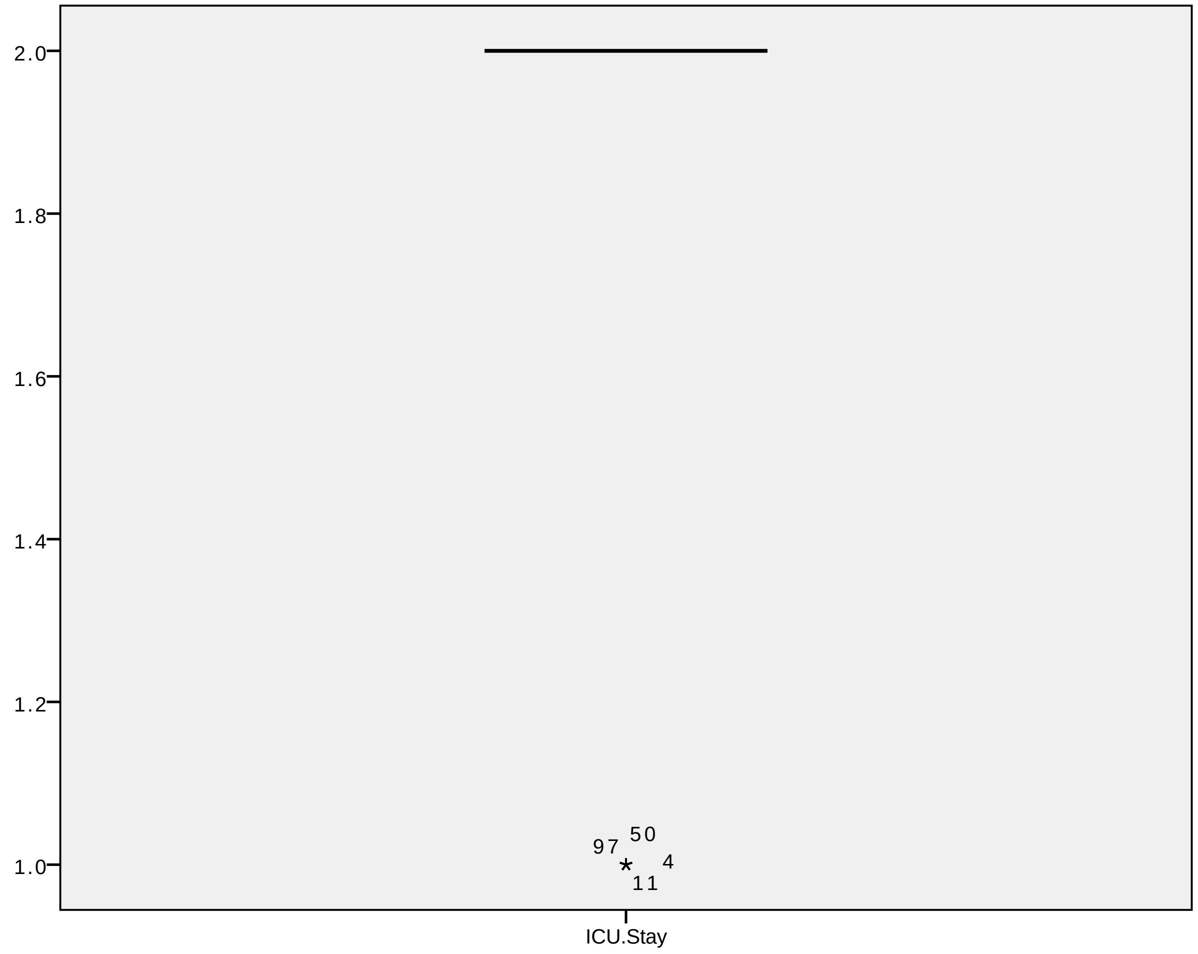

Duration.surgery

```
Duration.surgery Stem-and-Leaf Plot

 Frequency    Stem &  Leaf

     2.00        2 .  02
     4.00        2 .  5555
    10.00        3 .  0000000000
    17.00        3 .  55555555555555555
    16.00        4 .  0000000000000000
    19.00        4 .  5555555555555555558
    18.00        5 .  000000000000000000
    10.00        5 .  5555555555
     9.00        6 .  000000000
     5.00        6 .  55555
     3.00        7 .  000
     1.00        7 .  5
      .00        8 .
     2.00        8 .  55
     2.00 Extremes    (>=90)

 Stem width:     10.00
 Each leaf:        1 case(s)
```

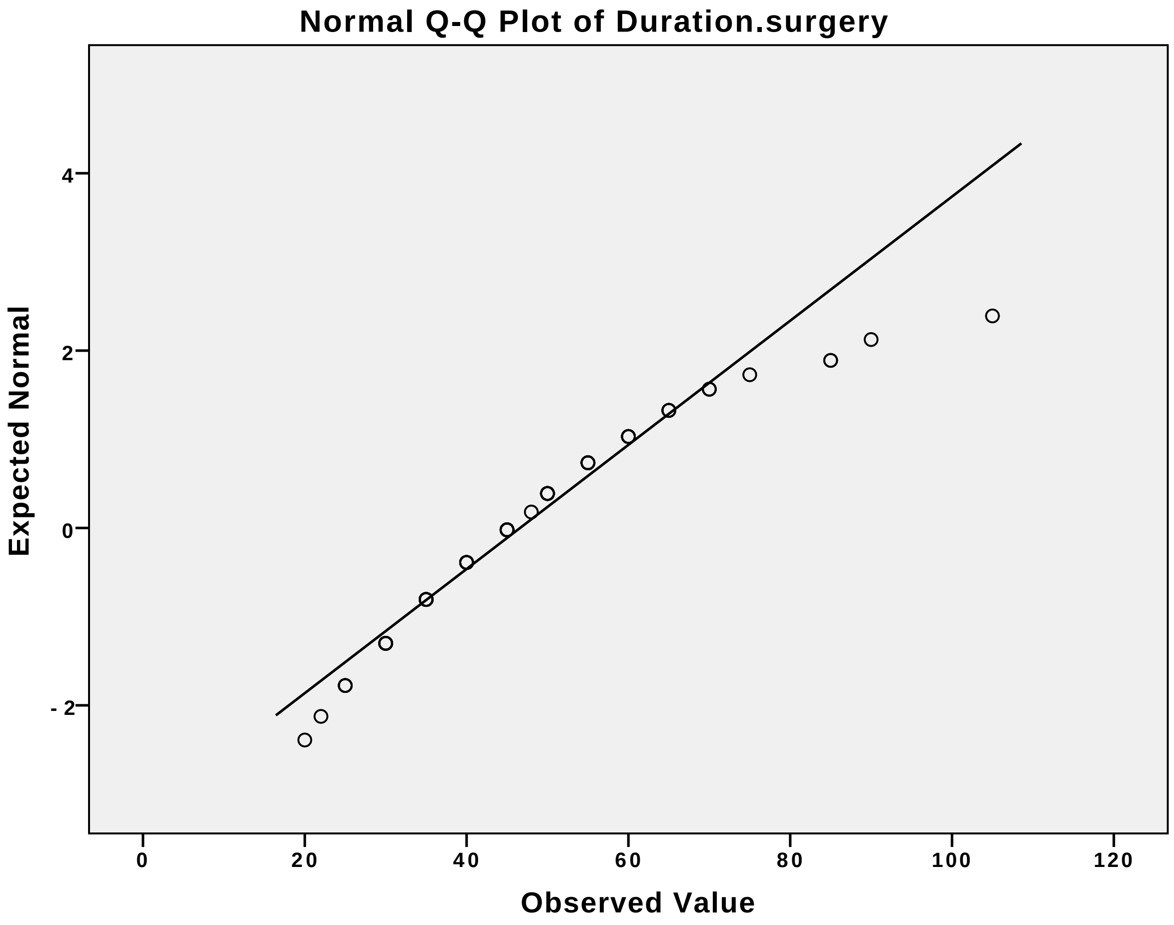

Normal Q-Q Plot of Duration.surgery
Expected Normal
4
2
0
- 2
0
20
40
60
80
100
120
Observed Value

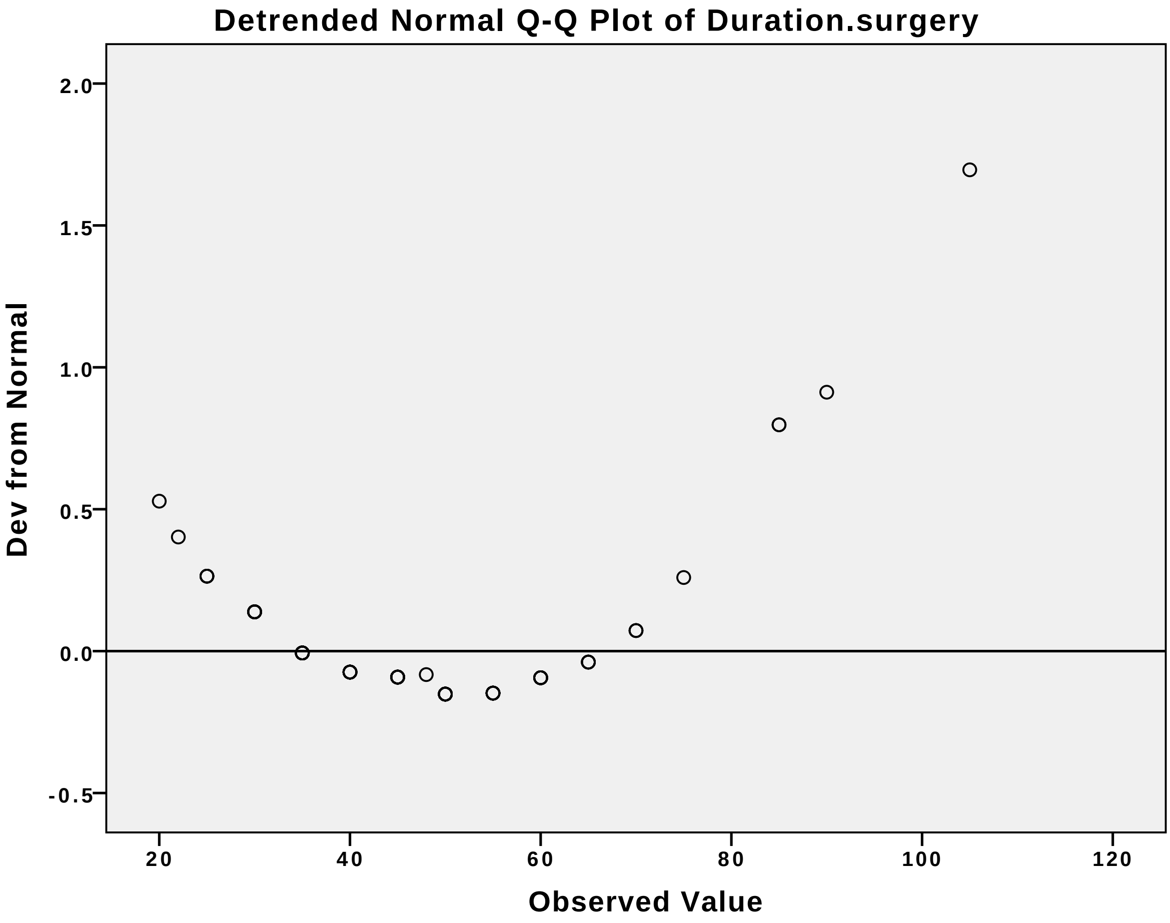
Detrended Normal Q-Q Plot of Duration.surgery
Dev from Normal
2.0
1.5
1.0
0.5
0.0
-0.5
20
40
60
80
100
120
Observed Value

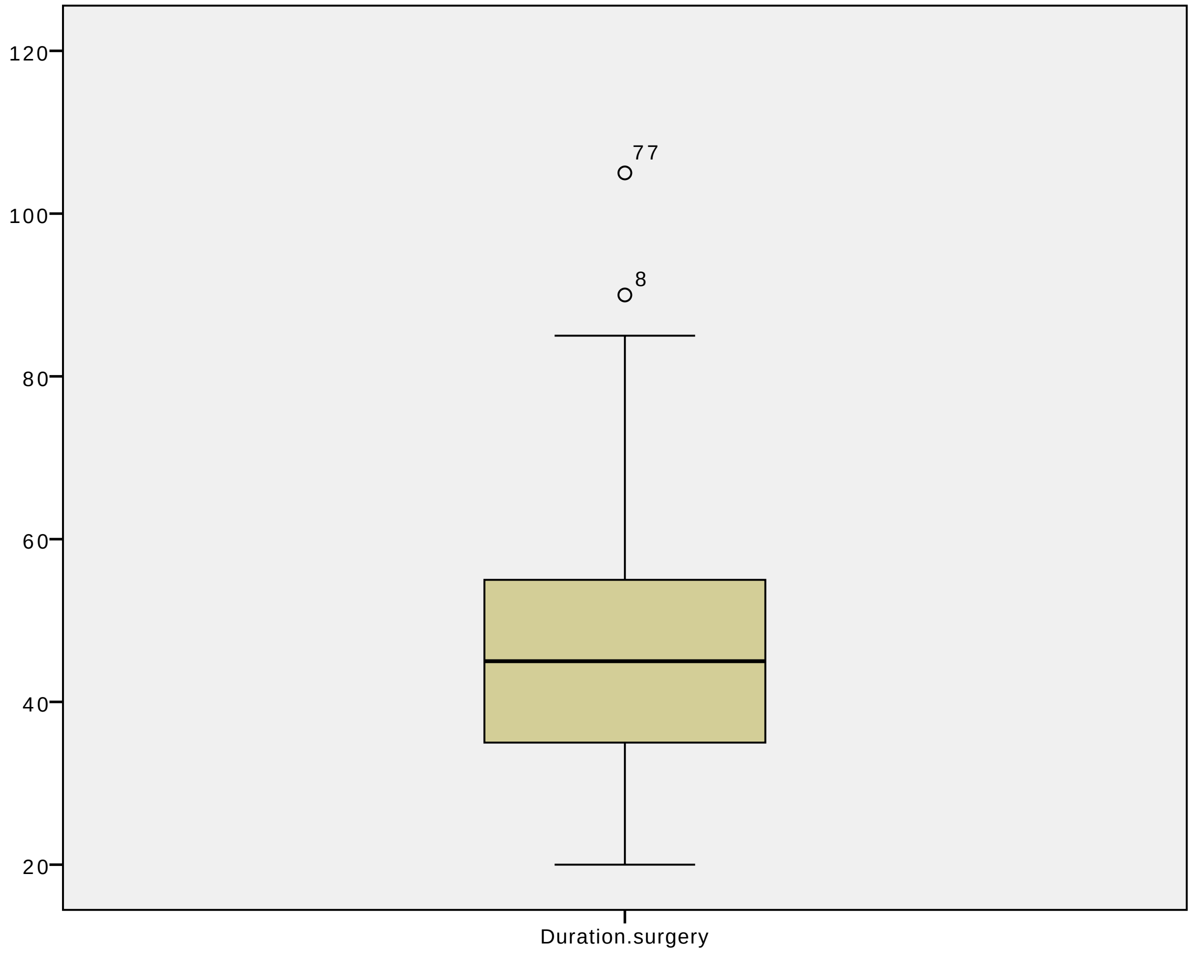


```
EXAMINE VARIABLES=Age Hospital.stay ICU.Stay Duration.surgery BY Compensato
ry.sweating
  /PLOT BOXPLOT STEMLEAF NPPLOT
  /COMPARE GROUPS
  /STATISTICS DESCRIPTIVES
  /CINTERVAL 95
  /MISSING LISTWISE
  /NOTOTAL.
```

Explore

Notes

Output Created		18-APR-2018 18:27:...
Comments		
Input	Data	C:\Users\rnordin. ADMIN\Desktop\2018\ PUBLICATION 2018 ETS\ETS.Data (Complete).sav 18APRIL2018.sav
	Active Dataset	DataSet1
	Filter	<none>
	Weight	<none>
	Split File	<none>
	N of Rows in Working Data File	118
Missing Value Handling	Definition of Missing	User-defined missing values for dependent variables are treated as missing.
	Cases Used	Statistics are based on cases with no missing values for any dependent variable or factor used.
Syntax		EXAMINE VARIABLES=Age Hospital.stay ICU.Stay Duration.surgery BY Compensatory.sweating /PLOT BOXPLOT STEMLEAF NPPLOT /COMPARE GROUPS /STATISTICS DESCRIPTIVES /CINTERVAL 95 /MISSING LISTWISE /NOTOTAL.
Resources	Processor Time	00:00:05.83
	Elapsed Time	00:00:02.35

CS

Case Processing Summary

	CS	Valid N	Valid Percent	Missing N	Missing Percent	Total N	Total Percent
Age	No	50	100.0%	0	0.0%	50	100.0%
	Yes	68	100.0%	0	0.0%	68	100.0%
Hospital.stay	No	50	100.0%	0	0.0%	50	100.0%
	Yes	68	100.0%	0	0.0%	68	100.0%
ICU.Stay	No	50	100.0%	0	0.0%	50	100.0%
	Yes	68	100.0%	0	0.0%	68	100.0%
Duration.surgery	No	50	100.0%	0	0.0%	50	100.0%
	Yes	68	100.0%	0	0.0%	68	100.0%

Descriptives

	CS			Statistic	Std. Error
Age	No	Mean		22.30	.939
		95% Confidence Interval for Mean	Lower Bound	20.41	
			Upper Bound	24.19	
		5% Trimmed Mean		21.92	
		Median		21.50	
		Variance		44.051	
		Std. Deviation		6.637	
		Minimum		9	
		Maximum		45	
		Range		36	
		Interquartile Range		8	
		Skewness		.942	.337
		Kurtosis		1.805	.662
	Yes	Mean		23.35	.935
		95% Confidence Interval for Mean	Lower Bound	21.49	
			Upper Bound	25.22	
		5% Trimmed Mean		22.75	
		Median		21.00	
		Variance		59.396	
		Std. Deviation		7.707	
		Minimum		10	
		Maximum		52	
		Range		42	
		Interquartile Range		9	
		Skewness		1.295	.291
		Kurtosis		2.255	.574
Hospital.stay	No	Mean		3.7400	.17799

Descriptives

	CS			Statistic	Std. Error
		95% Confidence Interval for Mean	Lower Bound	3.3823	
			Upper Bound	4.0977	
		5% Trimmed Mean		3.6333	
		Median		3.0000	
		Variance		1.584	
		Std. Deviation		1.25860	
		Minimum		1.00	
		Maximum		9.00	
		Range		8.00	
		Interquartile Range		1.00	
		Skewness		1.796	.337
		Kurtosis		5.489	.662
	Yes	Mean		3.4559	.10357
		95% Confidence Interval for Mean	Lower Bound	3.2492	
			Upper Bound	3.6626	
		5% Trimmed Mean		3.3954	
		Median		3.0000	
		Variance		.729	
		Std. Deviation		.85403	
		Minimum		2.00	
		Maximum		6.00	
		Range		4.00	
		Interquartile Range		1.00	
		Skewness		1.326	.291
		Kurtosis		1.929	.574
ICU.Stay	No	Mean		1.9800	.02000
		95% Confidence Interval for Mean	Lower Bound	1.9398	
			Upper Bound	2.0202	
		5% Trimmed Mean		2.0000	
		Median		2.0000	
		Variance		.020	
		Std. Deviation		.14142	
		Minimum		1.00	
		Maximum		2.00	
		Range		1.00	
		Interquartile Range		.00	
		Skewness		-7.071	.337
		Kurtosis		50.000	.662
	Yes	Mean		1.9559	.02509

Descriptives

CS				Statistic	Std. Error
		95% Confidence Interval for Mean	Lower Bound	1.9058	
			Upper Bound	2.0060	
		5% Trimmed Mean		2.0000	
		Median		2.0000	
		Variance		.043	
		Std. Deviation		.20688	
		Minimum		1.00	
		Maximum		2.00	
		Range		1.00	
		Interquartile Range		.00	
		Skewness		-4.541	.291
		Kurtosis		19.181	.574
Duration.surgery	No	Mean		47.8400	2.37768
		95% Confidence Interval for Mean	Lower Bound	43.0619	
			Upper Bound	52.6181	
		5% Trimmed Mean		46.7778	
		Median		45.0000	
		Variance		282.668	
		Std. Deviation		16.81273	
		Minimum		20.00	
		Maximum		105.00	
		Range		85.00	
		Interquartile Range		20.00	
		Skewness		1.078	.337
		Kurtosis		1.928	.662
	Yes	Mean		45.7059	1.47410
		95% Confidence Interval for Mean	Lower Bound	42.7636	
			Upper Bound	48.6482	
		5% Trimmed Mean		45.0980	
		Median		45.0000	
		Variance		147.763	
		Std. Deviation		12.15578	
		Minimum		25.00	
		Maximum		90.00	
		Range		65.00	
		Interquartile Range		17.50	
		Skewness		.838	.291
		Kurtosis		1.399	.574

Tests of Normality

	CS	Kolmogorov-Smirnov[a] Statistic	df	Sig.	Shapiro-Wilk Statistic	df	Sig.
Age	No	.090	50	.200*	.951	50	.037
	Yes	.135	68	.004	.906	68	.000
Hospital.stay	No	.282	50	.000	.753	50	.000
	Yes	.350	68	.000	.759	68	.000
ICU.Stay	No	.536	50	.000	.125	50	.000
	Yes	.540	68	.000	.209	68	.000
Duration.surgery	No	.149	50	.007	.929	50	.005
	Yes	.136	68	.003	.946	68	.005

*. This is a lower bound of the true significance.

a. Lilliefors Significance Correction

Age

Stem-and-Leaf Plots

```
Age Stem-and-Leaf Plot for
Compensatory.sweating= No

 Frequency    Stem &  Leaf

     1.00        0 .  9
     3.00        1 .  344
    16.00        1 .  5566777788888999
    12.00        2 .  001112223344
    13.00        2 .  5556666777899
     2.00        3 .  02
     2.00        3 .  57
     1.00 Extremes    (>=45)

 Stem width:        10
 Each leaf:        1 case(s)


Age Stem-and-Leaf Plot for
Compensatory.sweating= Yes

 Frequency    Stem &  Leaf

     2.00        1 .  04
    21.00        1 .  555555567777778888899
    16.00        2 .  0000001111112233
    19.00        2 .  5555555666667778889
     3.00        3 .  122
     4.00        3 .  5599
     3.00 Extremes    (>=40)
```

```
Stem width:        10
Each leaf:        1 case(s)
```

Normal Q-Q Plots

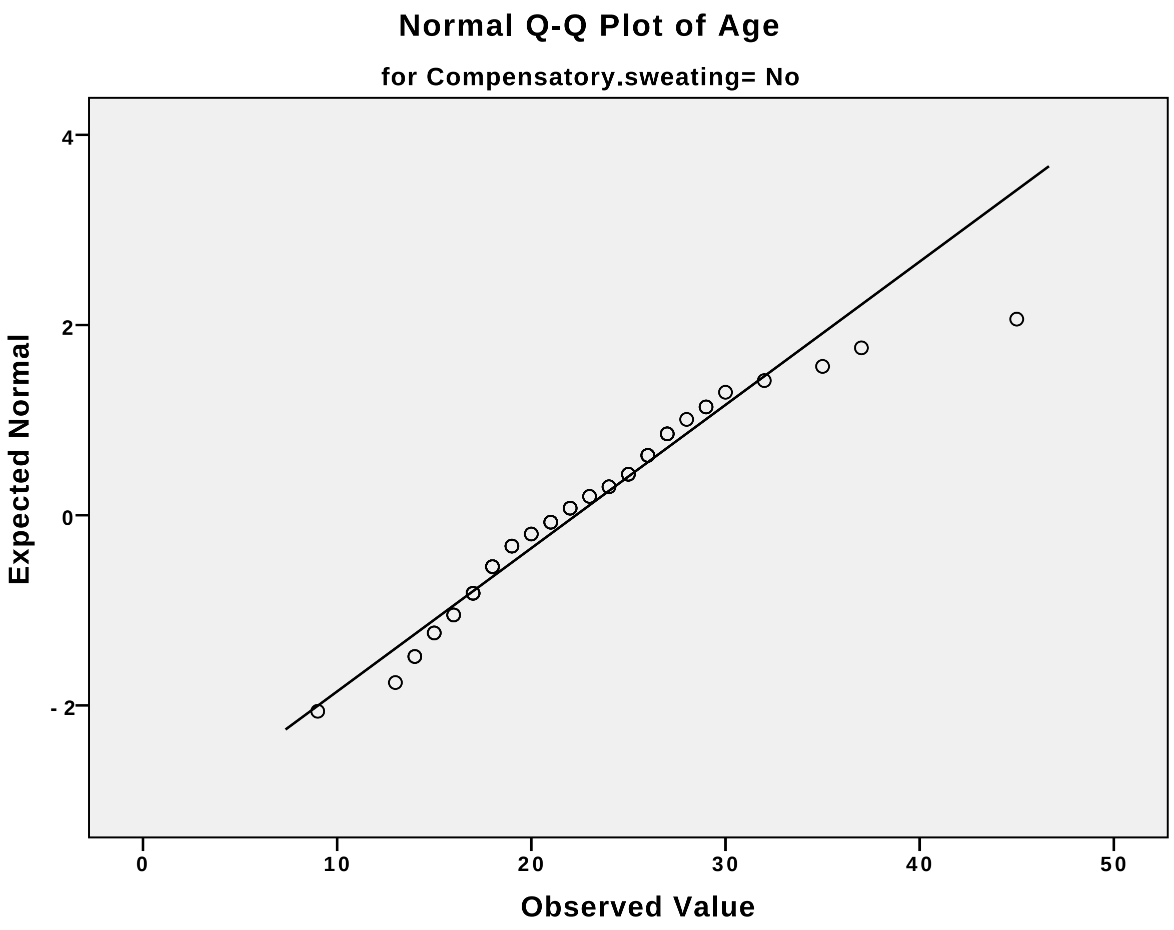

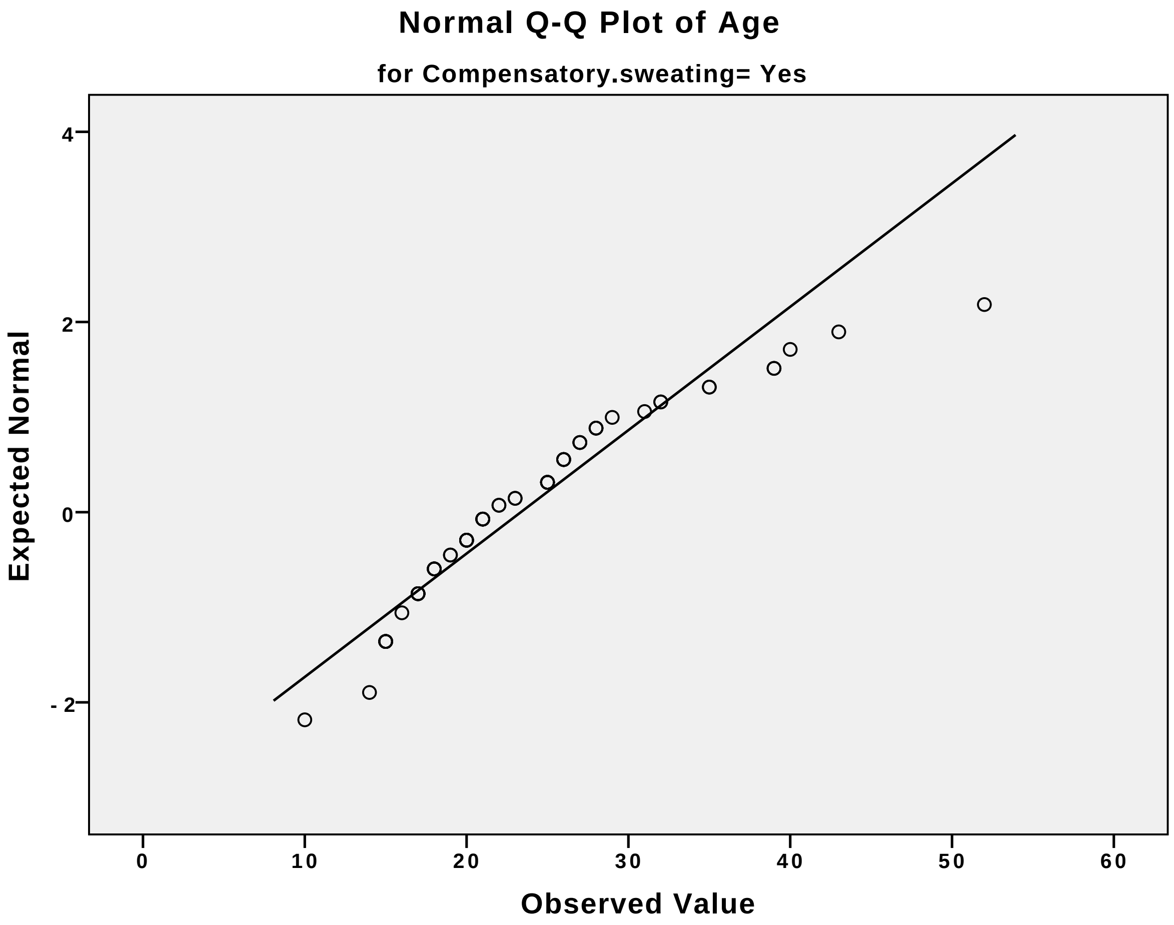

Detrended Normal Q-Q Plots

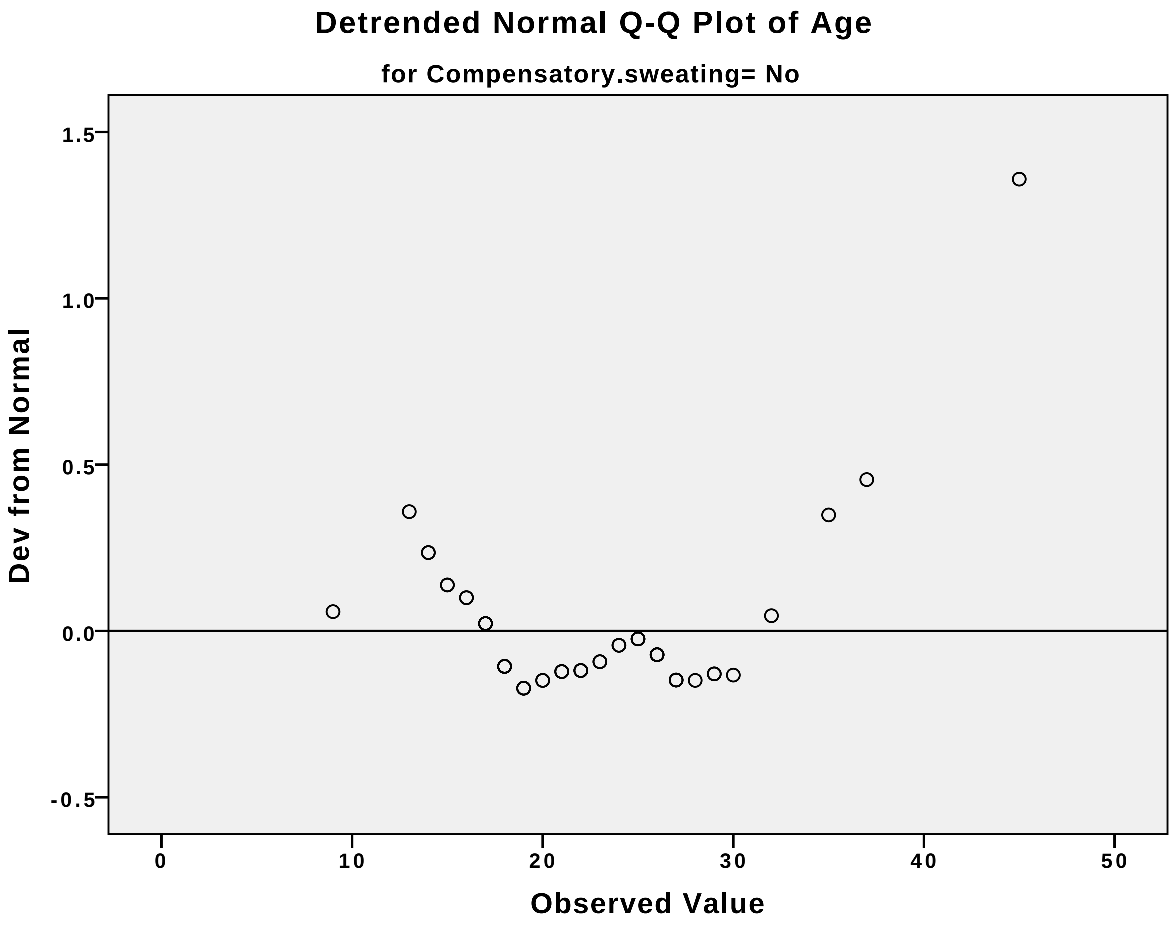
Detrended Normal Q-Q Plot of Age
for Compensatory.sweating= No
Dev from Normal
1.5
1.0
0.5
0.0
-0.5
0
10
20
30
40
50
Observed Value

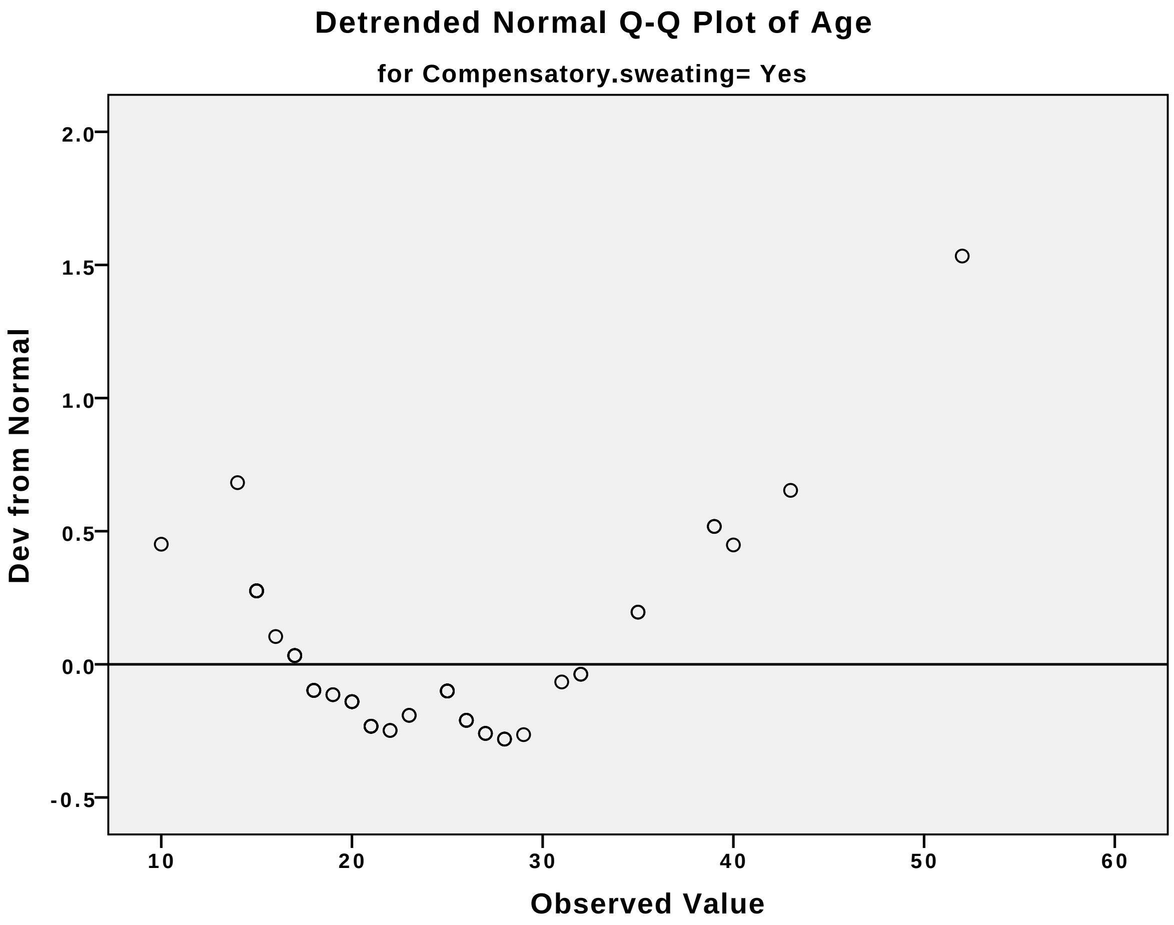
Detrended Normal Q-Q Plot of Age
for Compensatory.sweating= Yes
Dev from Normal
2.0
1.5
1.0
0.5
0.0
-0.5
10
20
30
40
50
60
Observed Value

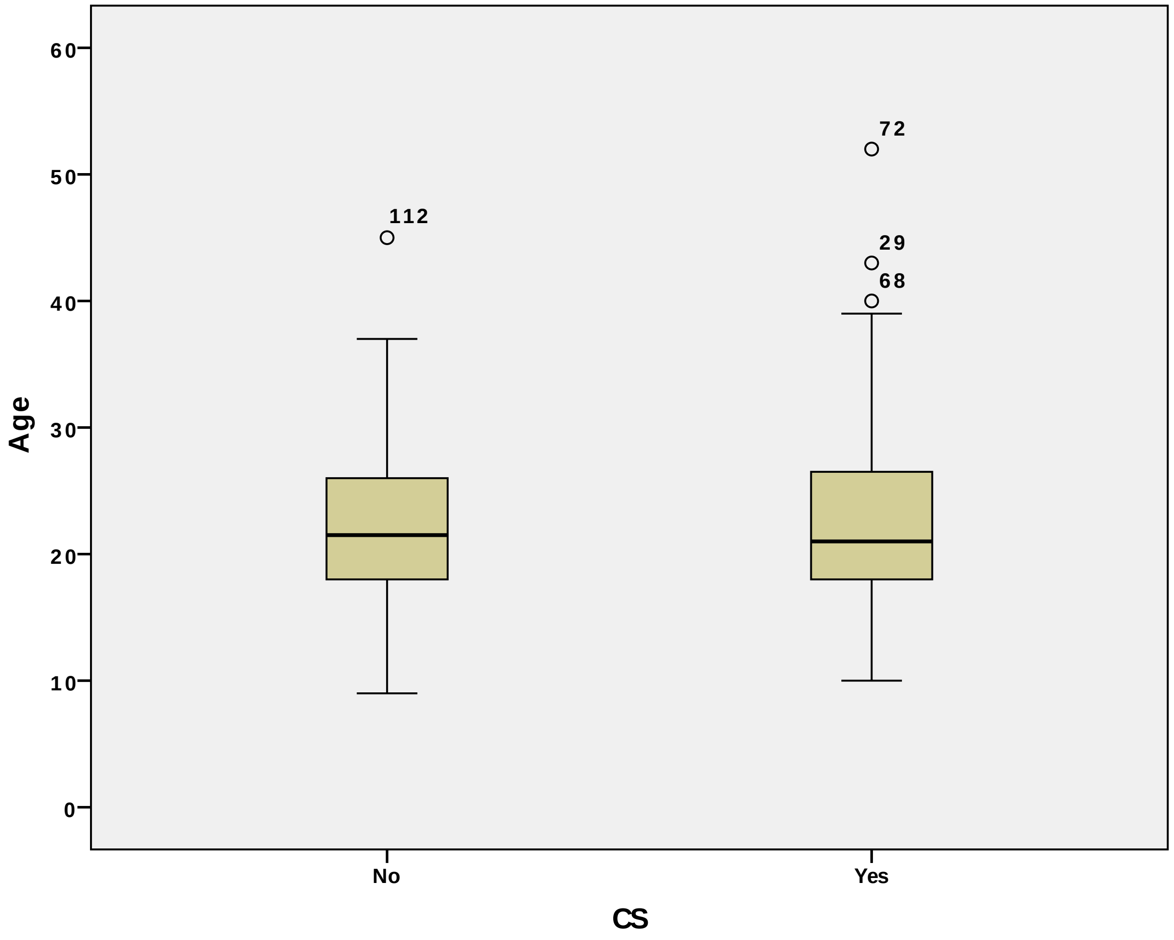

Hospital.stay

Stem-and-Leaf Plots

```
Hospital.stay Stem-and-Leaf Plot for
Compensatory.sweating= No

 Frequency    Stem &  Leaf

     1.00 Extremes    (=<1.0)
    27.00        3 .  000000000000000000000000000
      .00        3 .
    13.00        4 .  0000000000000
      .00        4 .
     4.00        5 .  0000
     5.00 Extremes    (>=6.0)

 Stem width:      1.00
 Each leaf:        1 case(s)


Hospital.stay Stem-and-Leaf Plot for
Compensatory.sweating= Yes
```

```
Frequency    Stem &  Leaf

     3.00        2 .  000
      .00        2 .
    41.00        3 .  00000000000000000000000000000000000000000
      .00        3 .
    17.00        4 .  00000000000000000
      .00        4 .
     4.00        5 .  0000
     3.00 Extremes    (>=6.0)

Stem width:      1.00
Each leaf:        1 case(s)
```

Normal Q-Q Plots

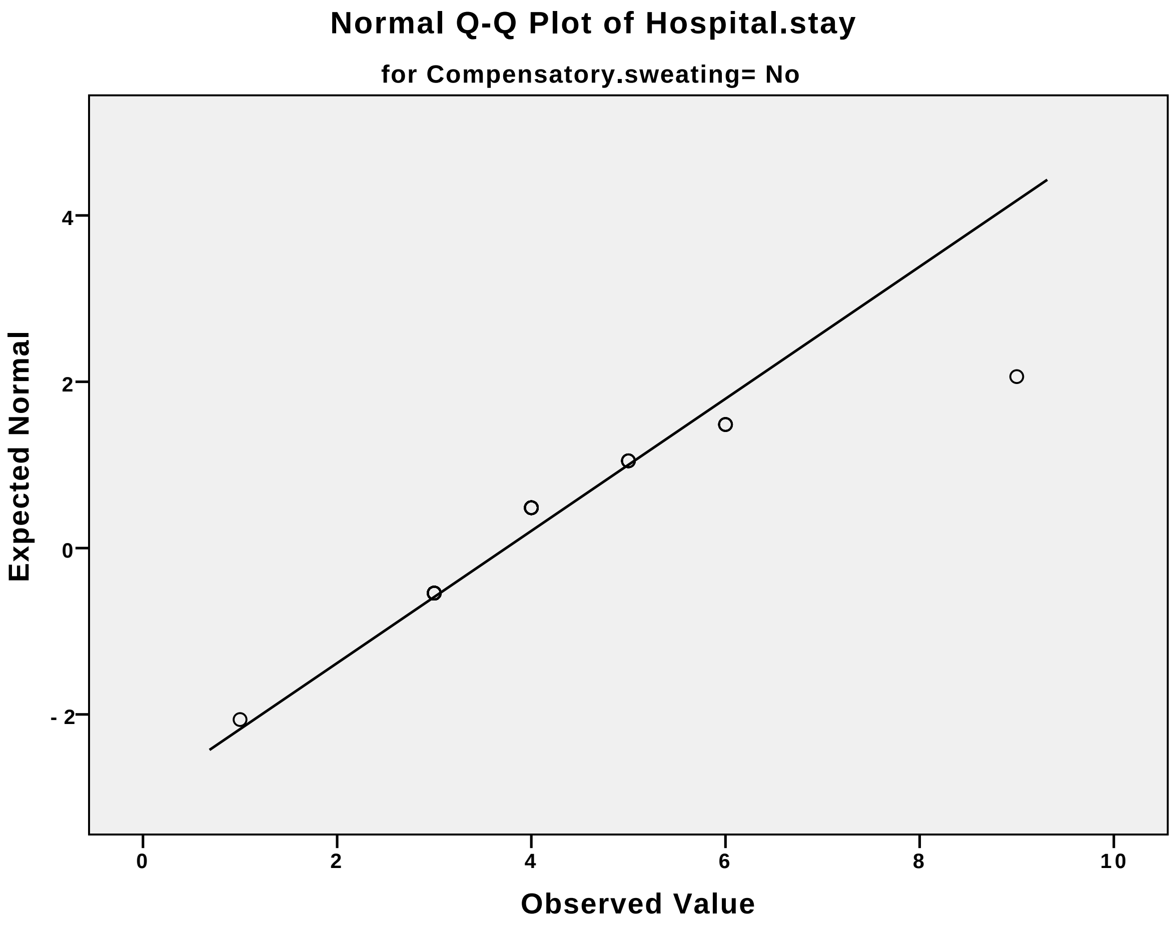

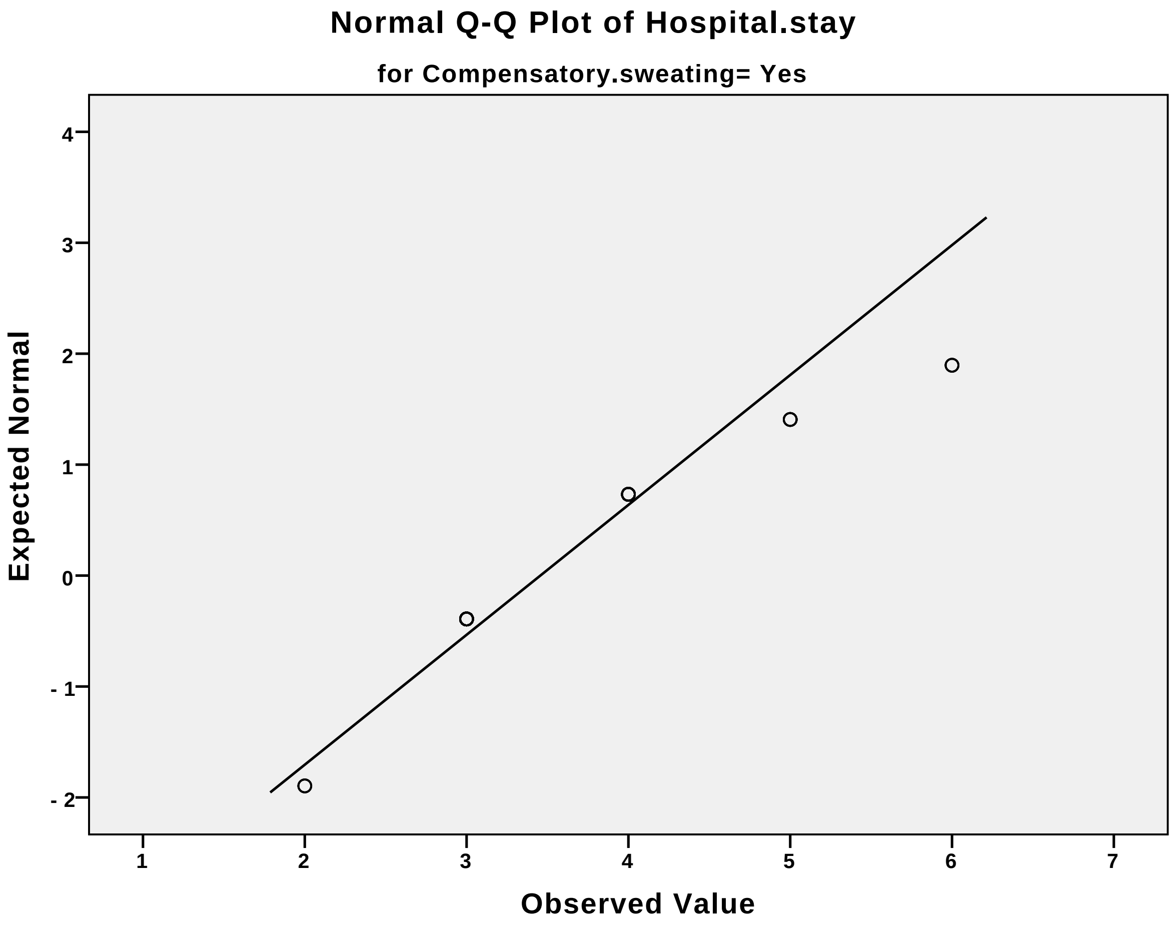

Detrended Normal Q-Q Plots

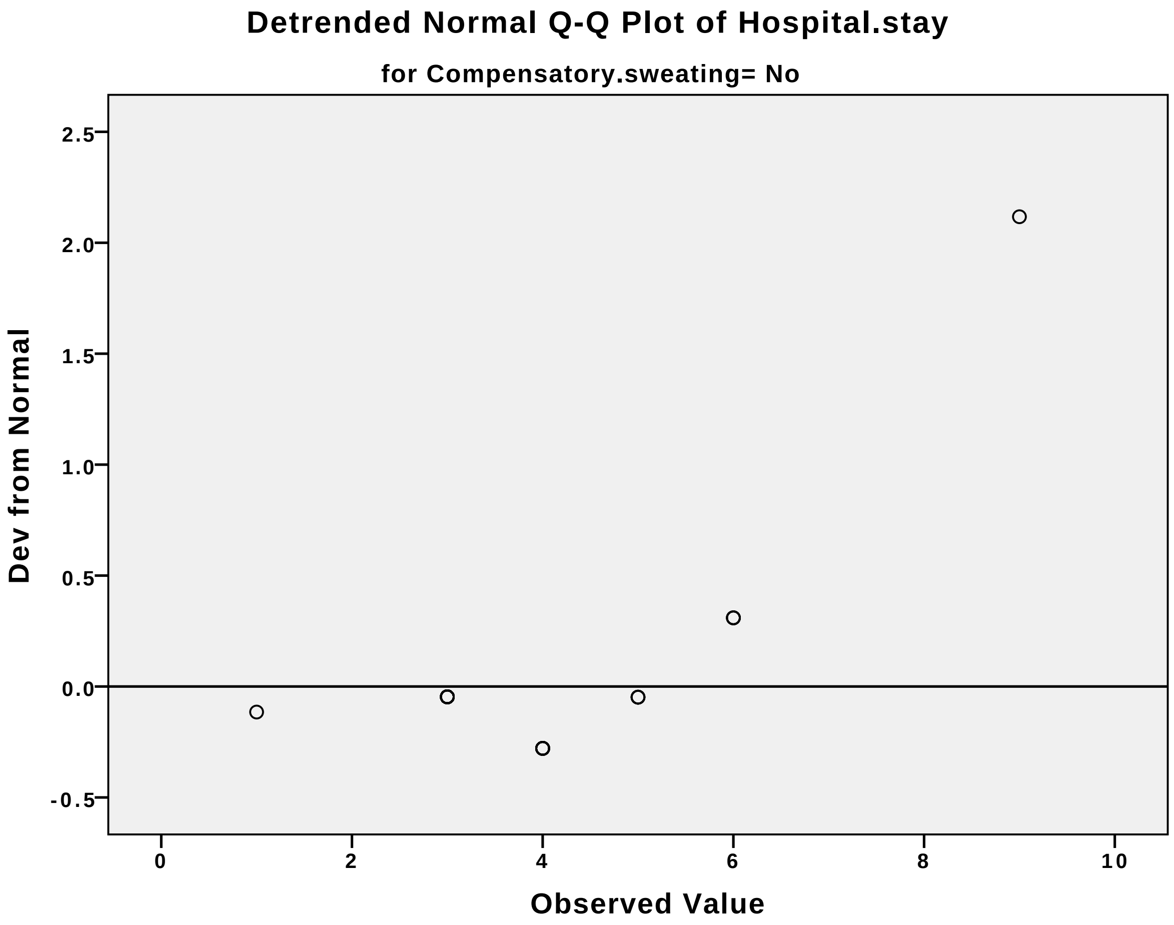
Detrended Normal Q-Q Plot of Hospital.stay
for Compensatory.sweating= No
Dev from Normal
2.5
2.0
1.5
1.0
0.5
0.0
-0.5
0
2
4
6
8
10
Observed Value

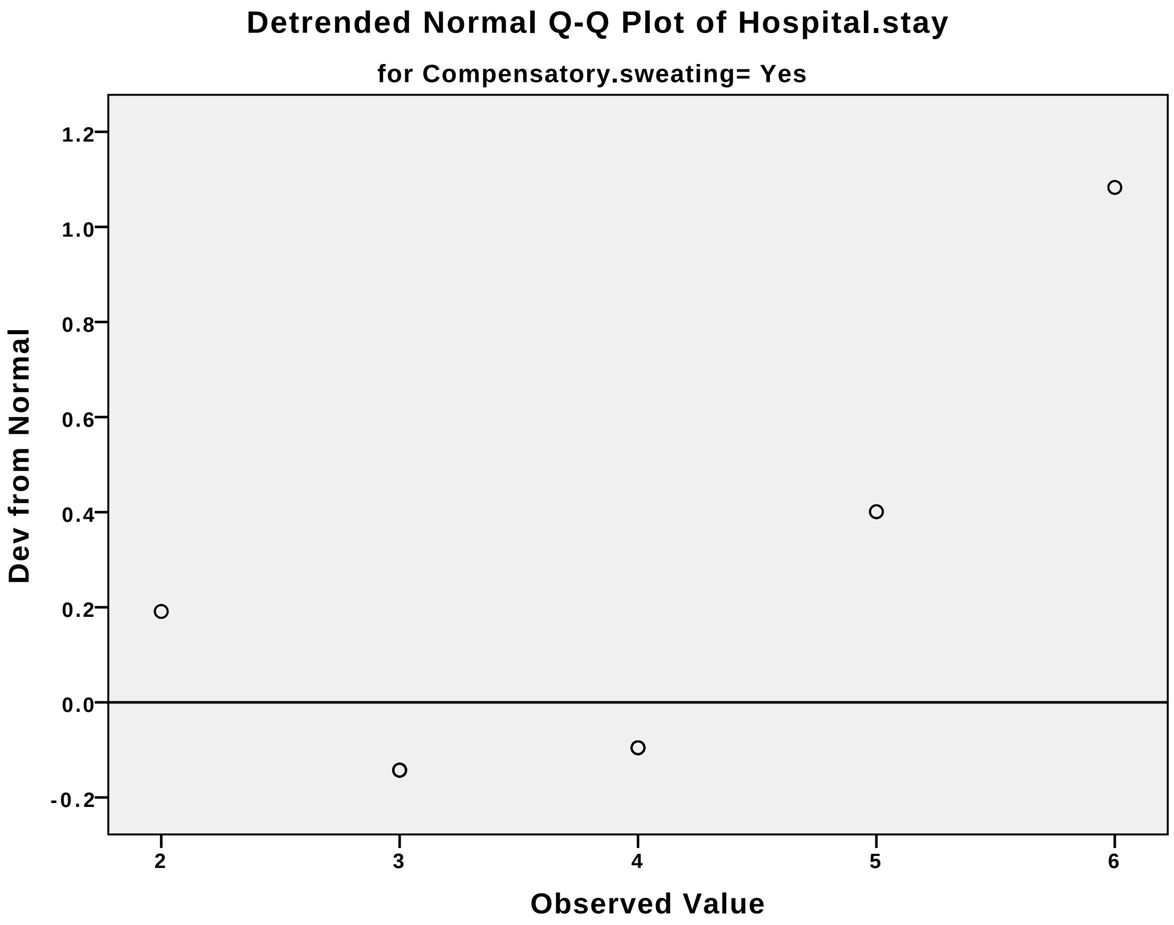
Detrended Normal Q-Q Plot of Hospital.stay
for Compensatory.sweating= Yes
Dev from Normal
1.2
1.0
0.8
0.6
0.4
0.2
0.0
-0.2
2
3
4
5
6
Observed Value

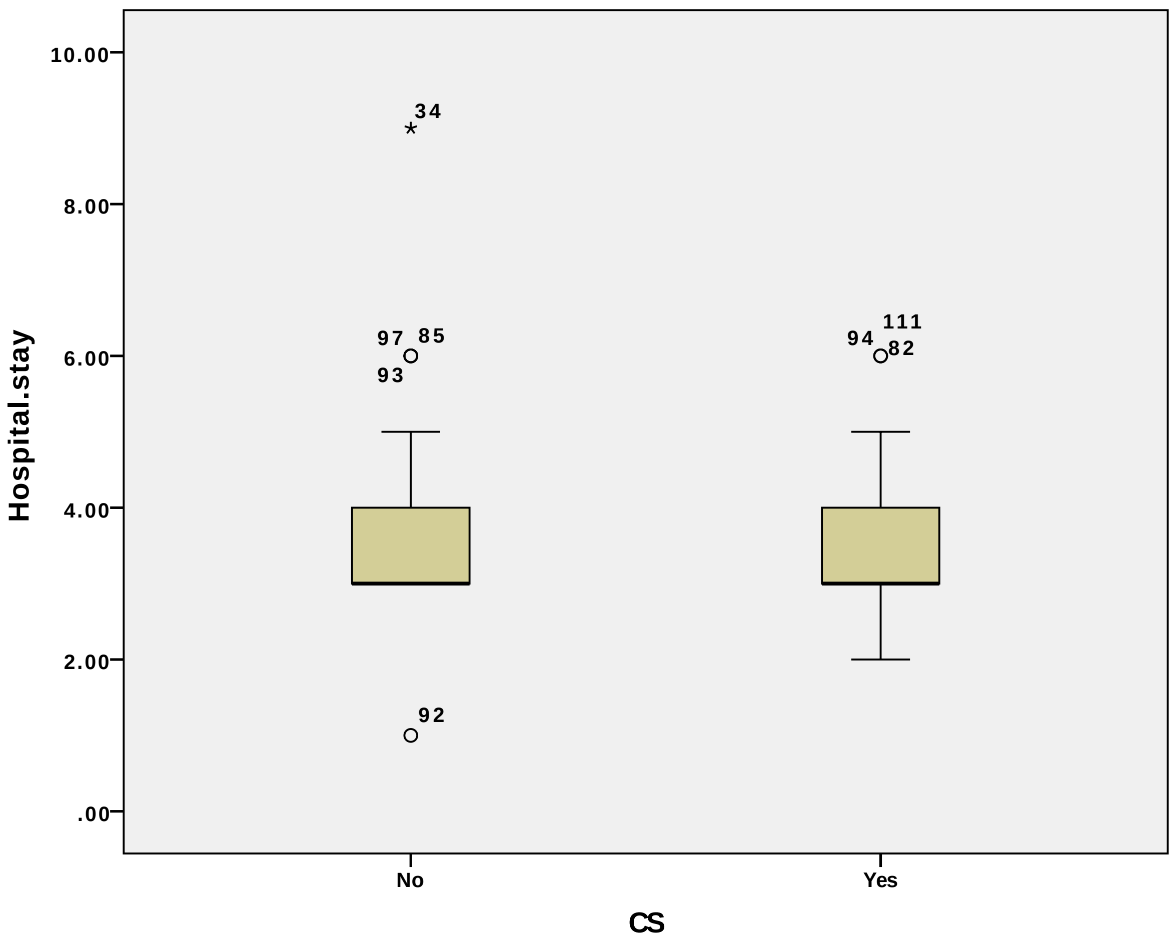

ICU.Stay

Stem-and-Leaf Plots

```
ICU.Stay Stem-and-Leaf Plot for
Compensatory.sweating= No

 Frequency    Stem &  Leaf

     1.00 Extremes    (=<1)
      .00        0 .
    49.00        0 .  2222222222222222222222222222222222222222222222222

 Stem width:     10.00
 Each leaf:        1 case(s)


ICU.Stay Stem-and-Leaf Plot for
Compensatory.sweating= Yes

 Frequency    Stem &  Leaf

     3.00 Extremes    (=<1)
      .00        0 .
```

```
    65.00        0 .  22222222222222222222222222222222222222222222222222222
222222222222

 Stem width:     10.00
 Each leaf:        1 case(s)
```

Normal Q-Q Plots

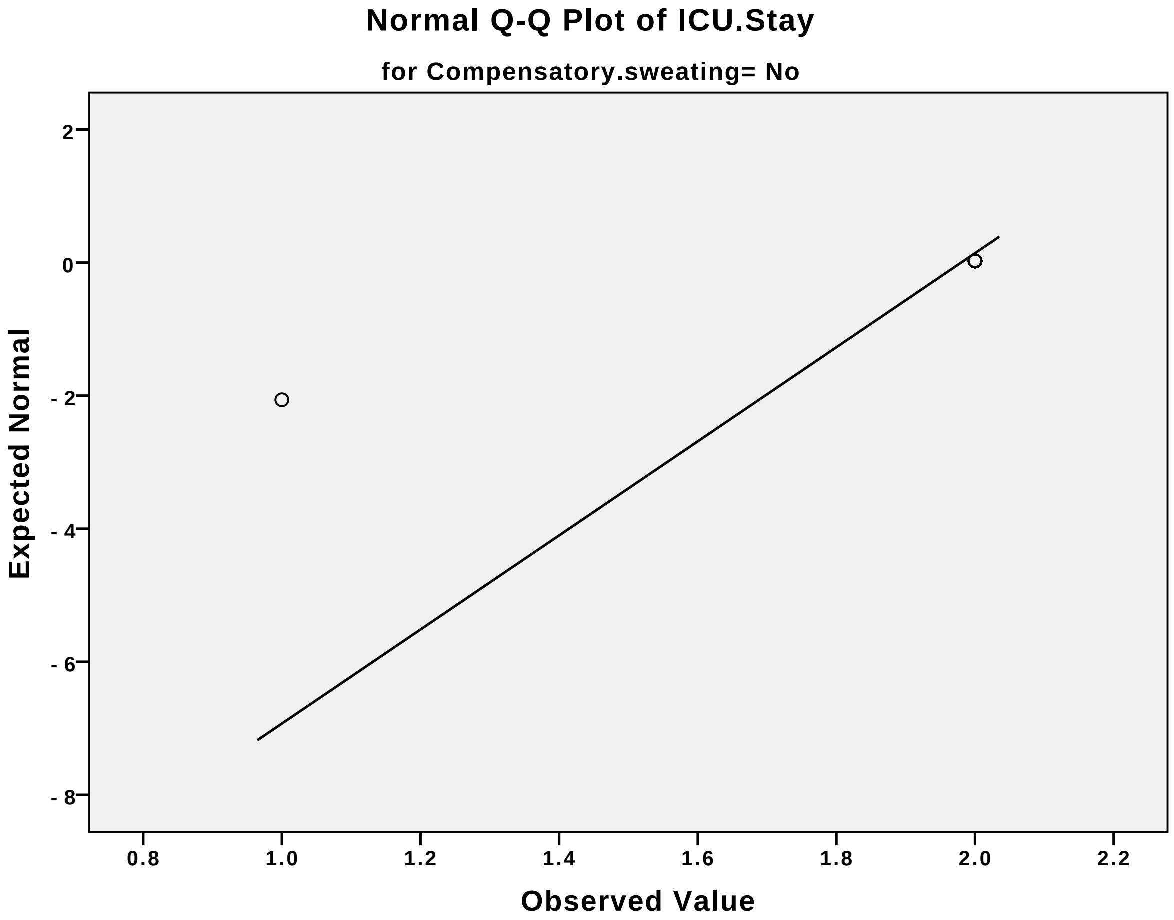

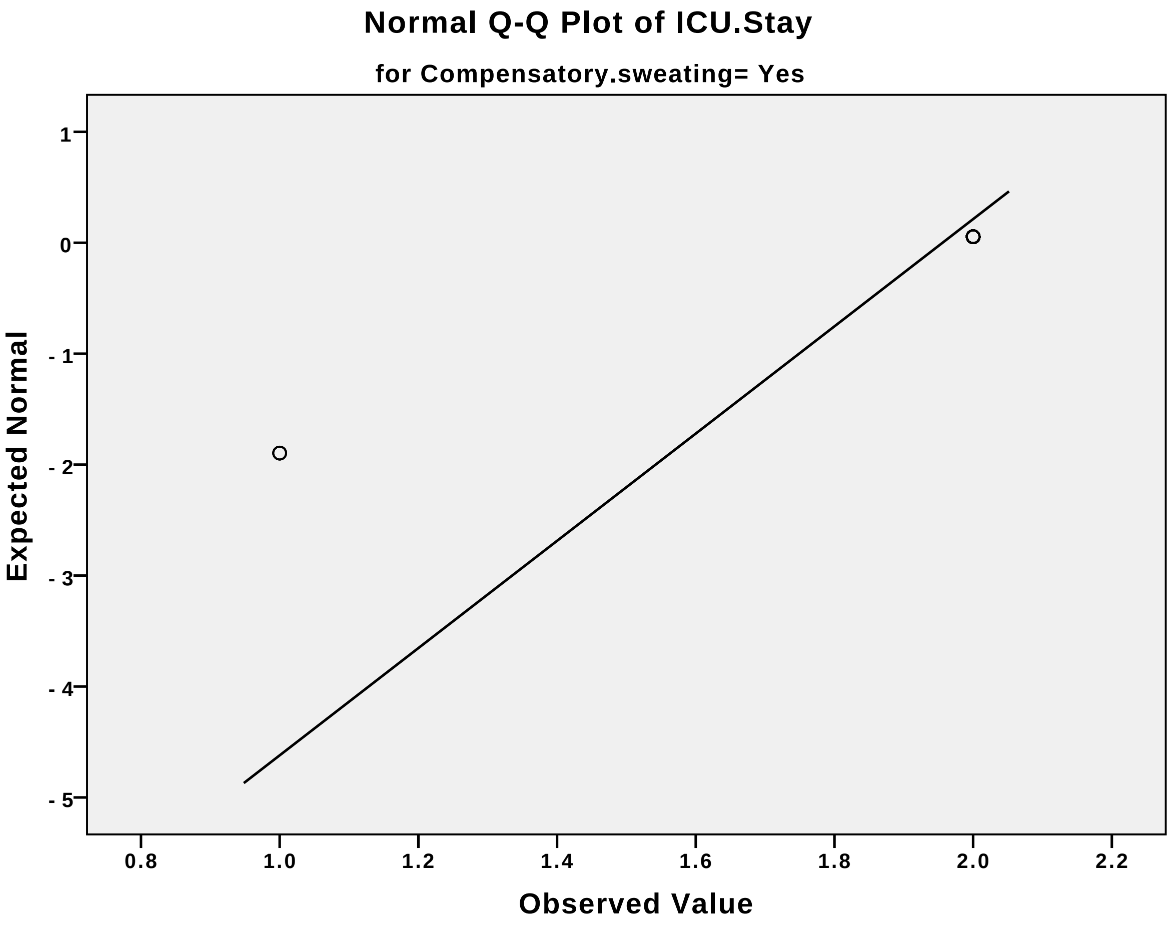

Detrended Normal Q-Q Plots

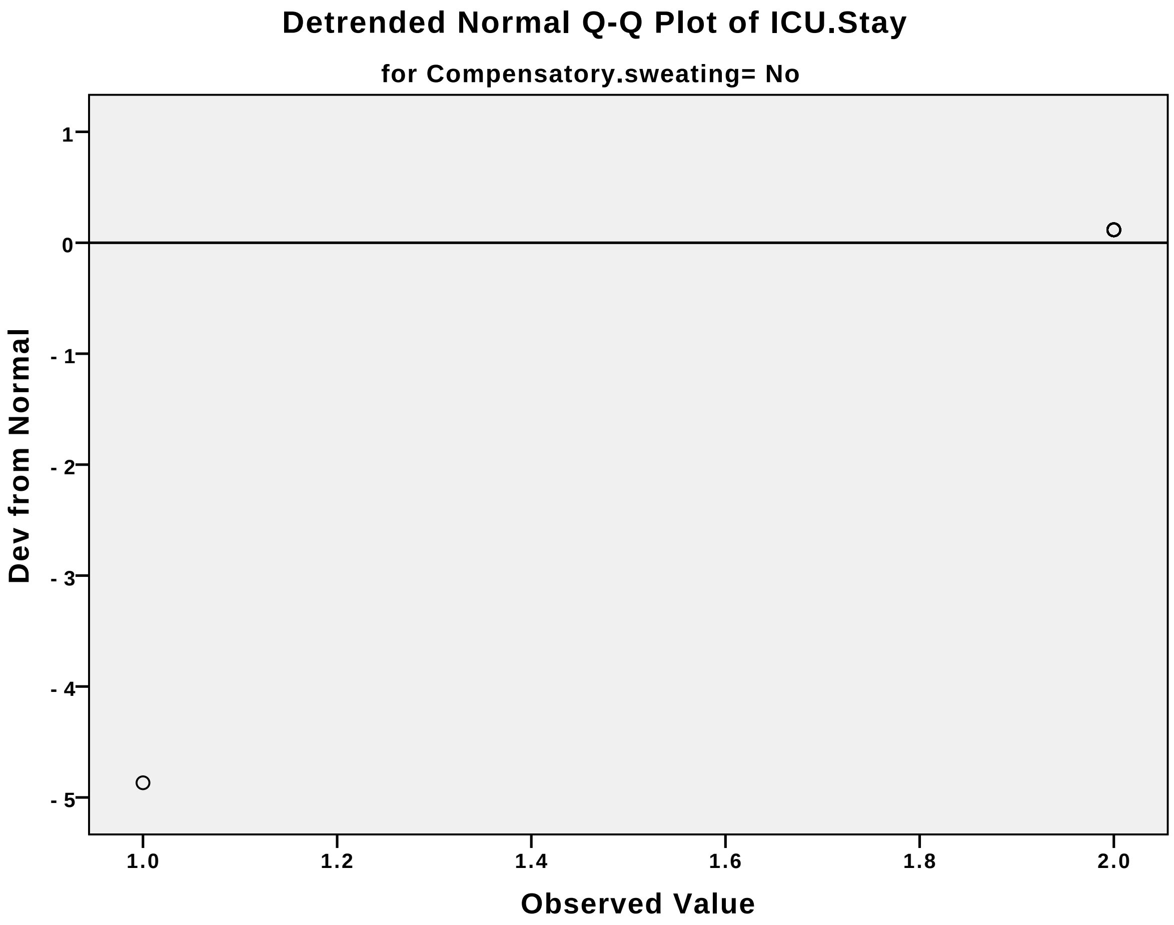

Detrended Normal Q-Q Plot of ICU.Stay
for Compensatory.sweating= No
Dev from Normal
1
0
- 1
- 2
- 3
- 4
- 5
1.0
1.2
1.4
1.6
1.8
2.0
Observed Value

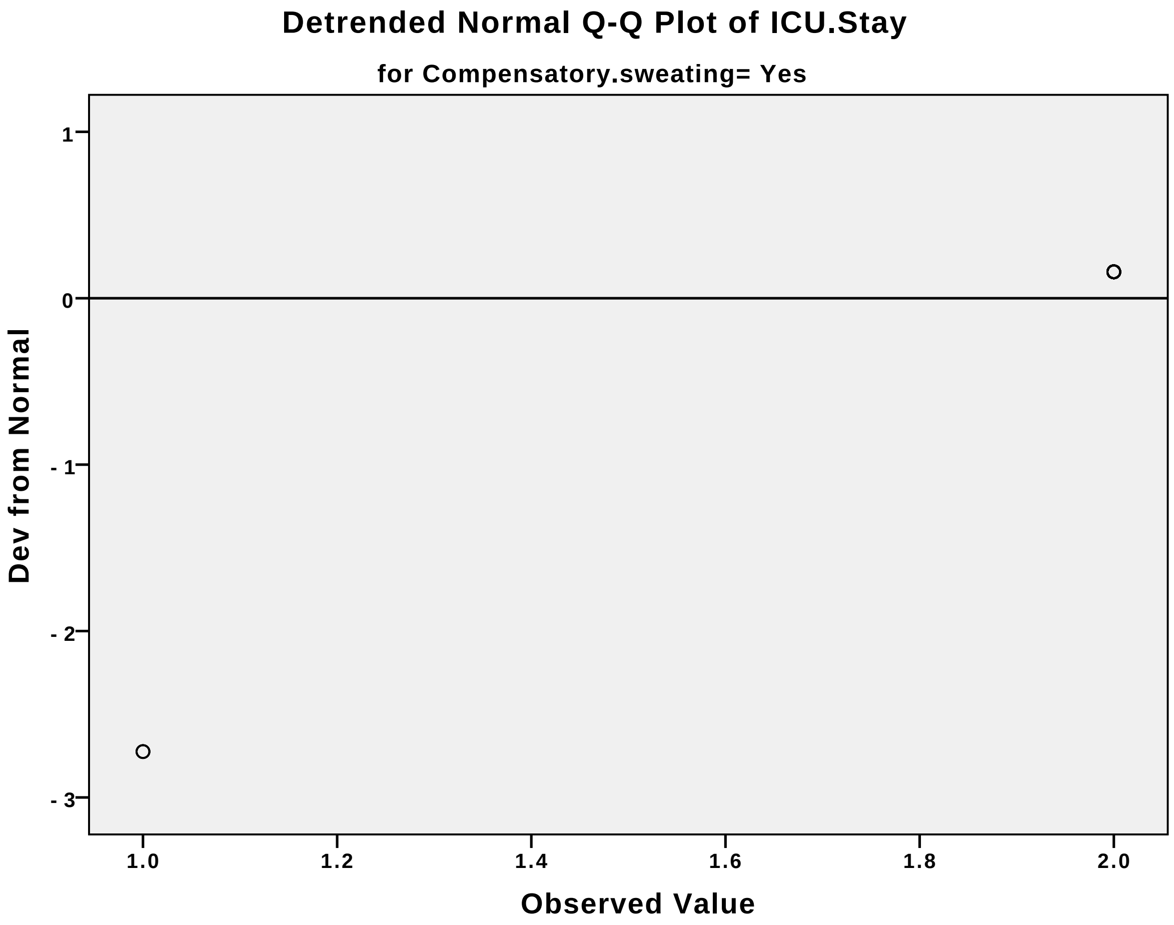
Detrended Normal Q-Q Plot of ICU.Stay
for Compensatory.sweating= Yes
Dev from Normal
Observed Value
1
0
- 1
- 2
- 3
1.0
1.2
1.4
1.6
1.8
2.0

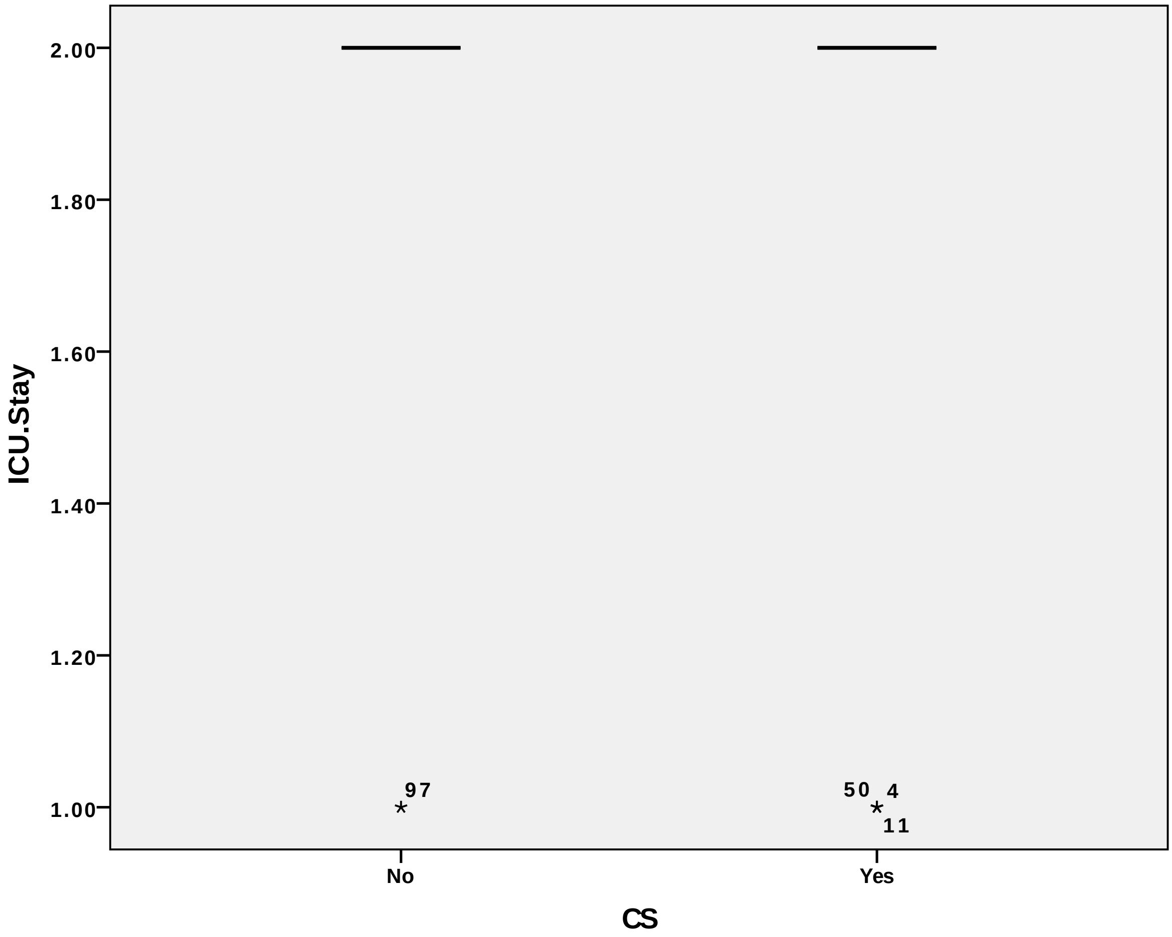

Duration.surgery

Stem-and-Leaf Plots

Duration.surgery Stem-and-Leaf Plot for
Compensatory.sweating= No

```
 Frequency    Stem &  Leaf

     2.00        2 .  02
     2.00        2 .  55
     3.00        3 .  000
     9.00        3 .  555555555
     2.00        4 .  00
     9.00        4 .  555555555
     8.00        5 .  00000000
     5.00        5 .  55555
     2.00        6 .  00
     3.00        6 .  555
     1.00        7 .  0
     1.00        7 .  5
      .00        8 .
     2.00        8 .  55
     1.00 Extremes    (>=105)
```

```
 Stem width:     10.00
 Each leaf:        1 case(s)


Duration.surgery Stem-and-Leaf Plot for
Compensatory.sweating= Yes

 Frequency    Stem &  Leaf

     2.00        2 .  55
    15.00        3 .  000000055555555
    24.00        4 .  000000000000005555555558
    15.00        5 .  000000000055555
     9.00        6 .  000000055
     2.00        7 .  00
     1.00 Extremes    (>=90)

 Stem width:     10.00
 Each leaf:        1 case(s)
```

Normal Q-Q Plots

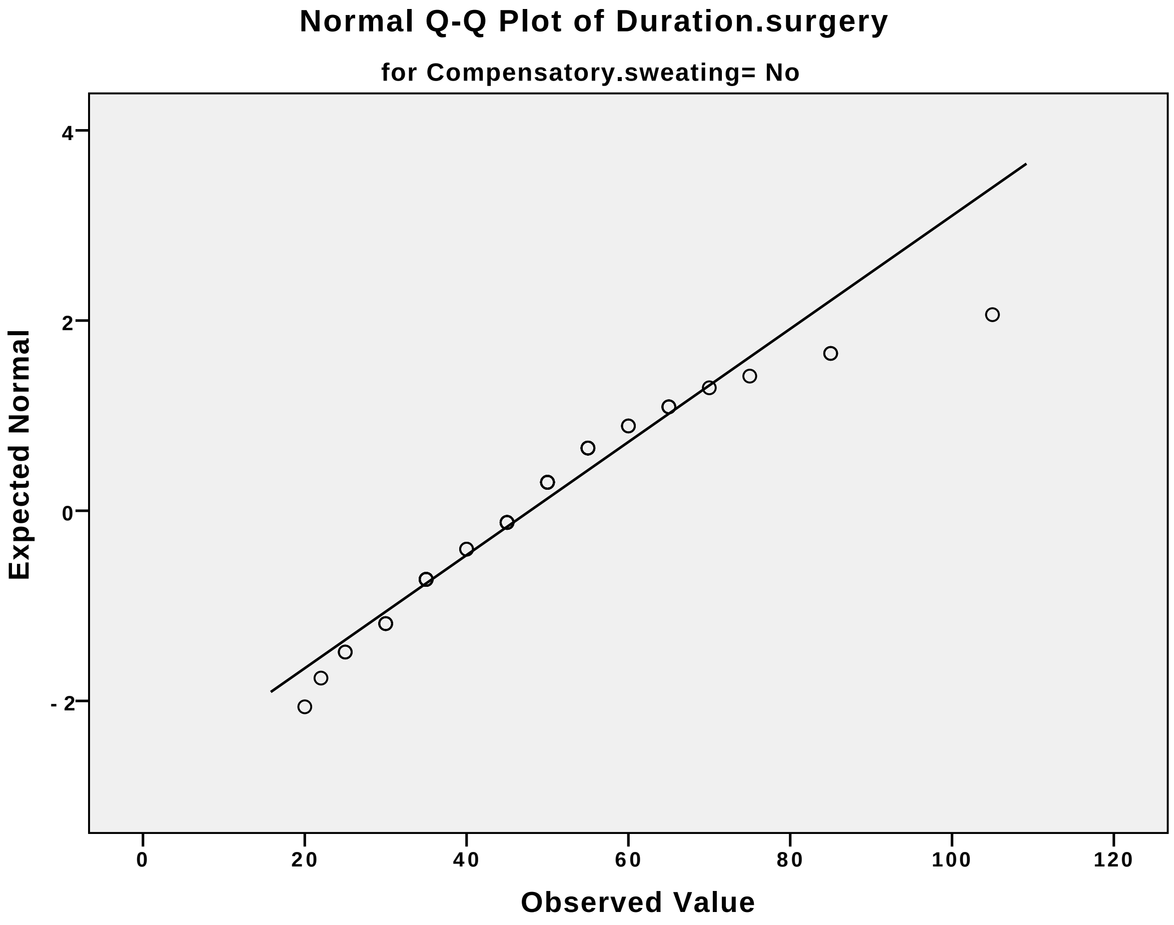

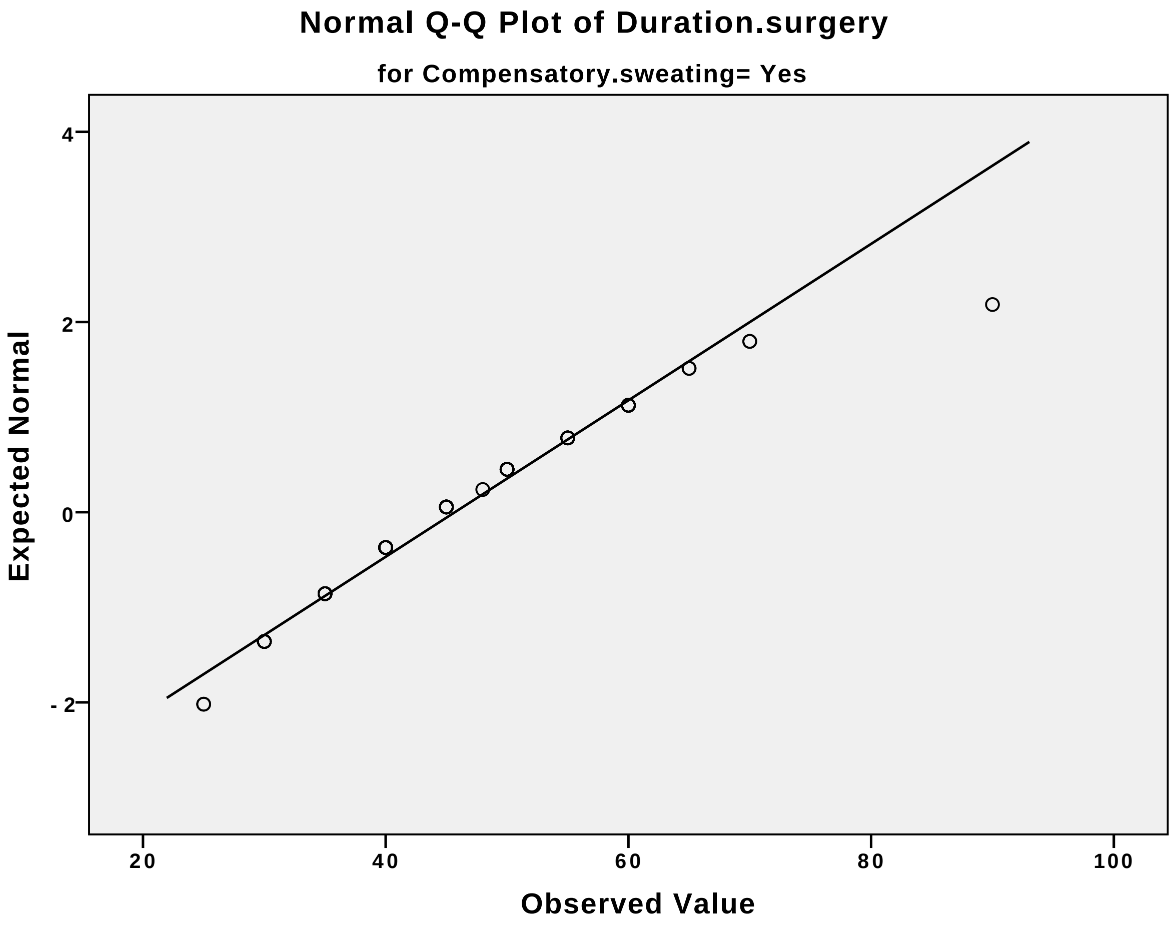

Detrended Normal Q-Q Plots

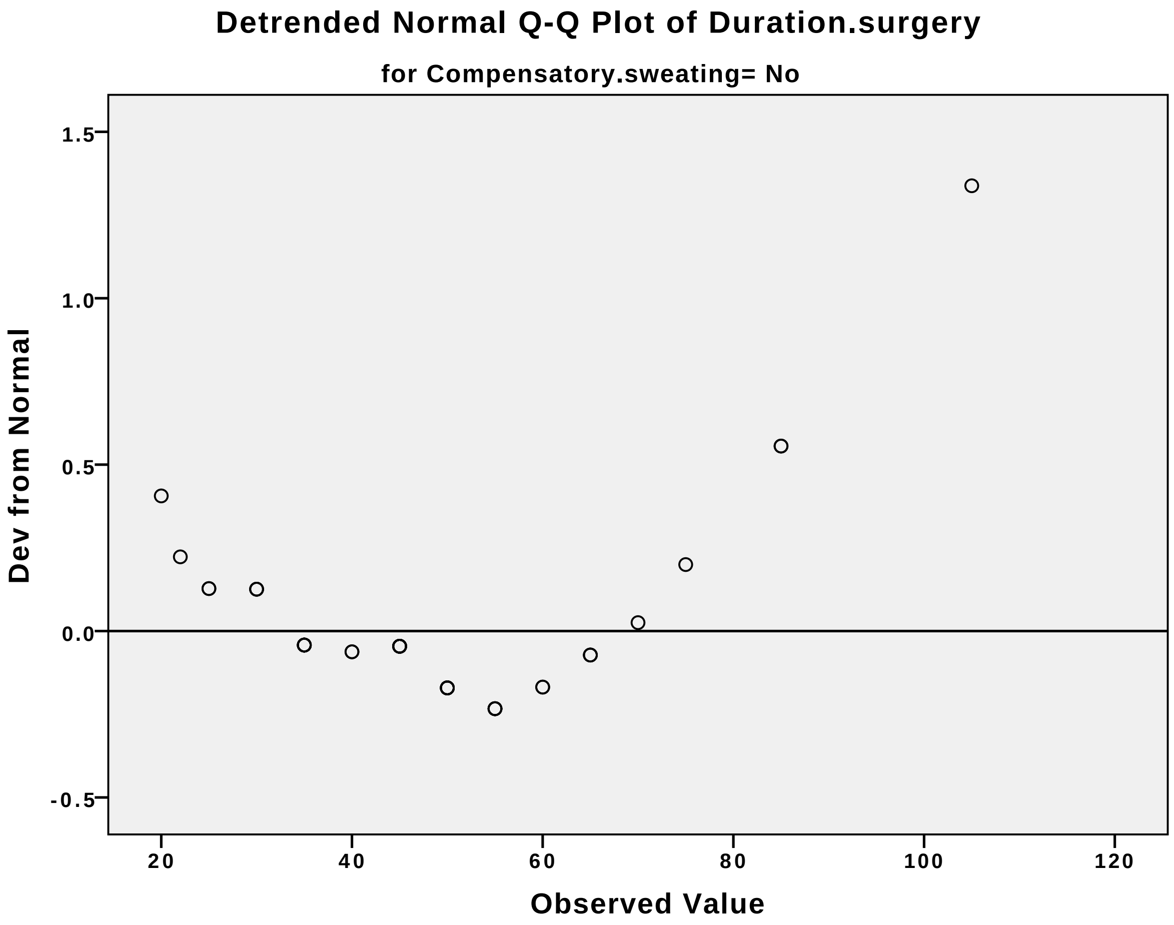

Detrended Normal Q-Q Plot of Duration.surgery
for Compensatory.sweating= No
Dev from Normal
1.5
1.0
0.5
0.0
-0.5
20
40
60
80
100
120
Observed Value

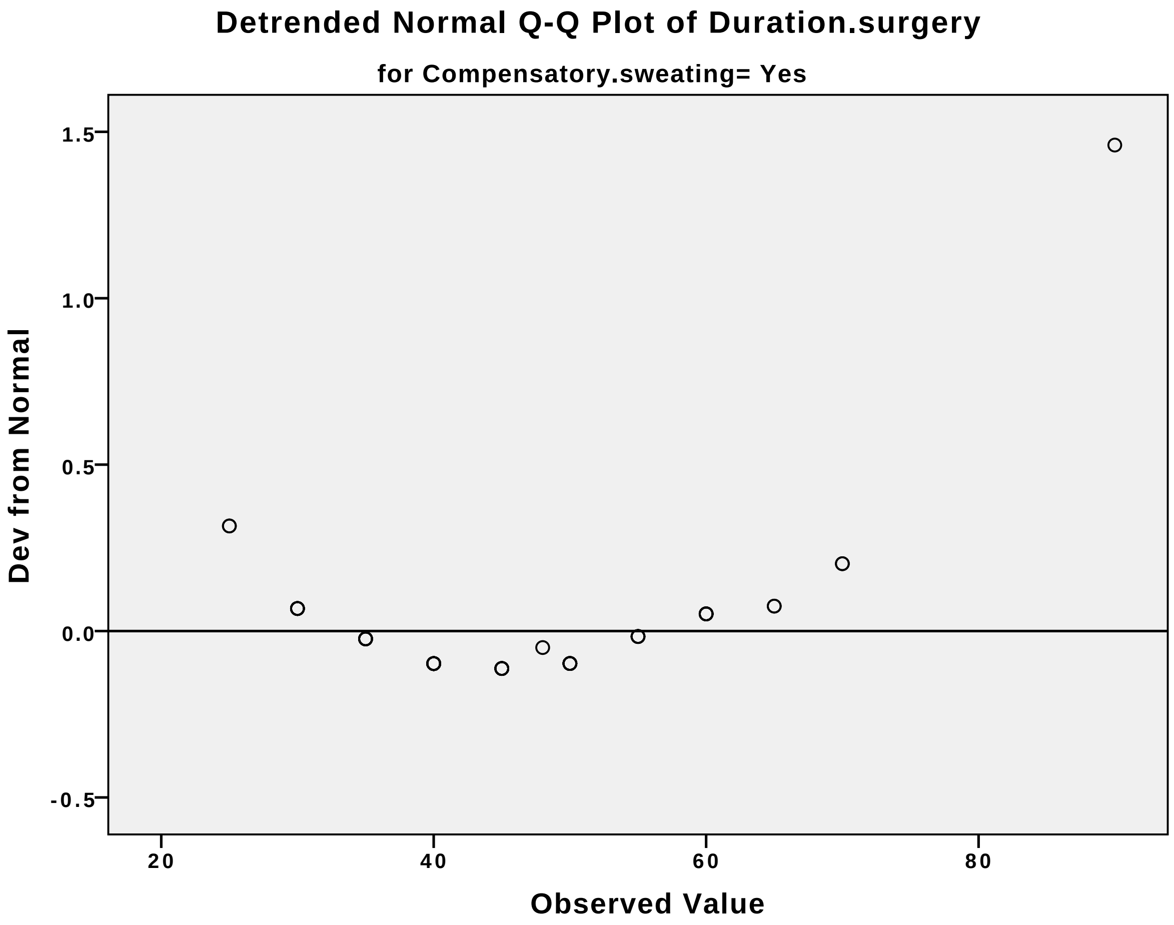
Detrended Normal Q-Q Plot of Duration.surgery
for Compensatory.sweating= Yes
Dev from Normal
1.5
1.0
0.5
0.0
-0.5
20
40
60
80
Observed Value

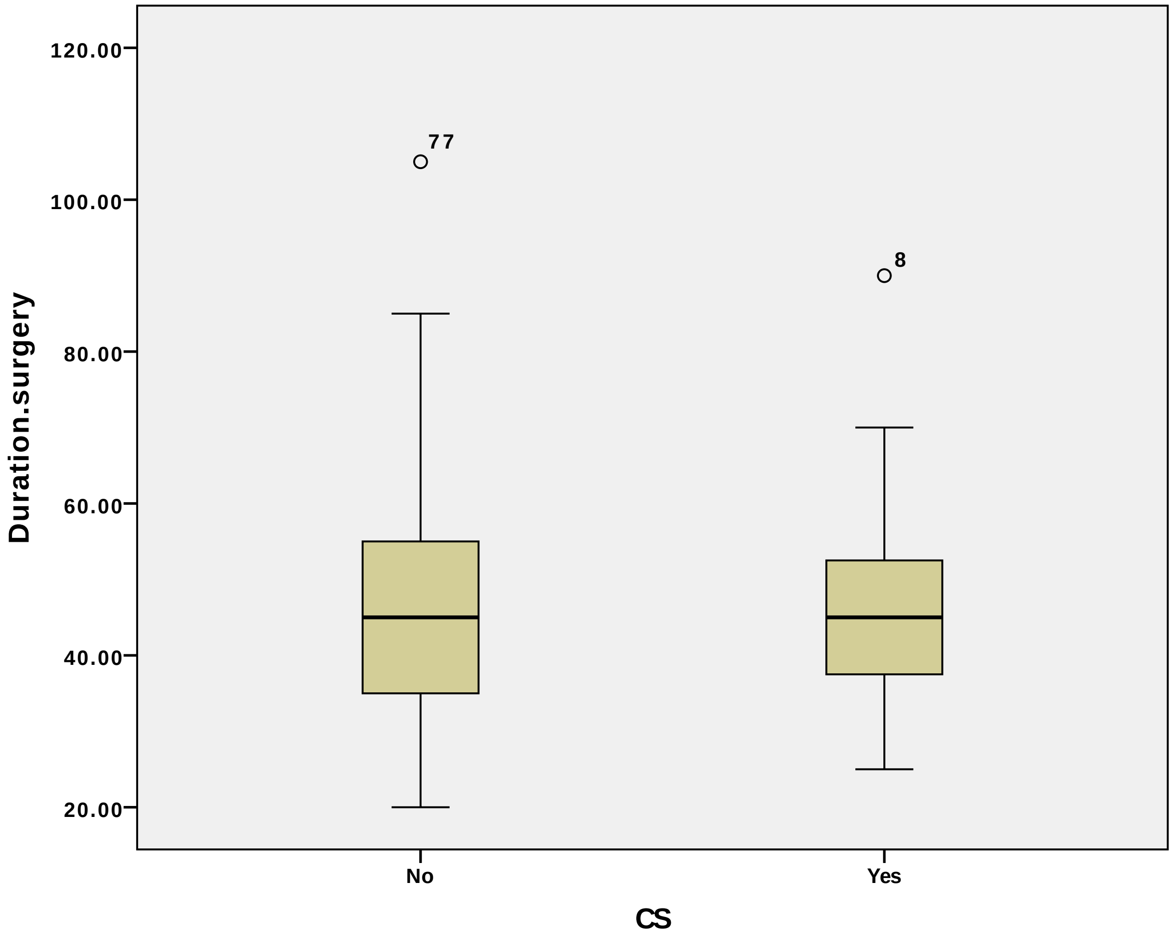


```
 SORT CASES BY DurationOfSurgery(A).
SORT CASES BY DurationOfSurgery(D).
SORT CASES BY Duration.surgery(A).
SORT CASES BY Duration.surgery(A).
SORT CASES BY Duration.surgery(D).
SORT CASES BY Duration.surgery(A).
DATASET ACTIVATE DataSet1.

SAVE OUTFILE='C:\Users\rnordin.ADMIN\Desktop\2018\PUBLICATION 2018 ETS\ETS.
Data(Complete).sav '+
    '18APRIL2018.sav'
  /COMPRESSED.
SORT CASES BY Conversion.to.open.surgery(A).
SORT CASES BY Conversion.to.open.surgery(D).
SORT CASES BY Analgesia (A).
SORT CASES BY Analgesia (D).
SORT CASES BY ICU.Stay (A).
SORT CASES BY ICU.Stay (D).
SORT CASES BY Hospital.stay(A).
SORT CASES BY Hospital.stay(D).
SORT CASES BY ComplicationYN(A).
SORT CASES BY ComplicationYN(D).
```

```
SORT CASES BY Follow.up (A).
SORT CASES BY Follow.up (D).
SORT CASES BY Number.of.follow.up(A).
SORT CASES BY FollowupYN (A).
SORT CASES BY FollowupYN (D).
SORT CASES BY Issues (A).
SORT CASES BY Compensatory.sweating(A).
SORT CASES BY Severity (A).
SORT CASES BY Location.of.CS (A).
SORT CASES BY Location.of.CS (D).
SORT CASES BY follow.up.progression(A).
SORT CASES BY follow.up.progression(D).
DATASET ACTIVATE DataSet1.

SAVE OUTFILE='C:\Users\rnordin.ADMIN\Desktop\2018\PUBLICATION 2018 ETS\ETS.
Data(Complete).sav '+
    '18APRIL2018.sav'
  /COMPRESSED.
LOGISTIC REGRESSION VARIABLES Compensatory.sweating
  /METHOD=ENTER AgeMedian Sex Race Marital.Status Occupation2 BMINOO Medica
l.issues
    Patient.position Sympathectomy.Level DurationOfSurgery ComplicationYN F
ollowupYN
  /CONTRAST (AgeMedian)=Indicator
  /CONTRAST (Sex)=Indicator
  /CONTRAST (Race)=Indicator(1)
  /CONTRAST (Marital.Status)=Indicator(1)
  /CONTRAST (Occupation2)=Indicator(1)
  /CONTRAST (BMINOO)=Indicator(1)
  /CONTRAST (Medical.issues)=Indicator(1)
  /CONTRAST (Patient.position)=Indicator(1)
  /CONTRAST (DurationOfSurgery)=Indicator(1)
  /CONTRAST (ComplicationYN)=Indicator(1)
  /CONTRAST (FollowupYN)=Indicator(1)
  /CONTRAST (Sympathectomy.Level)=Indicator(1)
  /PRINT=GOODFIT CI(95)
  /CRITERIA=PIN(0.05) POUT(0.10) ITERATE(20) CUT(0.5).
```

Logistic Regression

Notes

Output Created		18-APR-2018 18:38:...
Comments		
Input	Data	C:\Users\rnordin.ADMIN\Desktop\2018\PUBLICATION 2018 ETS\ETS.Data (Complete).sav 18APRIL2018.sav
	Active Dataset	DataSet1
	Filter	<none>
	Weight	<none>
	Split File	<none>
	N of Rows in Working Data File	118
Missing Value Handling	Definition of Missing	User-defined missing values are treated as missing

Notes

Syntax		LOGISTIC REGRESSION VARIABLES Compensatory.sweating /METHOD=ENTER AgeMedian Sex Race Marital.Status Occupation2 BMINOO Medical.issues Patient.position Sympathectomy.Level DurationOfSurgery ComplicationYN FollowupYN /CONTRAST (AgeMedian)=Indicator /CONTRAST (Sex)=Indicator /CONTRAST (Race)=Indicator(1) /CONTRAST (Marital.Status)=Indicator(1) /CONTRAST (Occupation2)=Indicator(1) /CONTRAST (BMINOO)=Indicator(1) /CONTRAST (Medical.issues)=Indicator(1) /CONTRAST (Patient.position)=Indicator(1) /CONTRAST (DurationOfSurgery)=Indicator(1) /CONTRAST (ComplicationYN)=Indicator(1) /CONTRAST (FollowupYN)=Indicator(1) /CONTRAST (Sympathectomy.Level)=Indicator(1) /PRINT=GOODFIT CI(95) /CRITERIA=PIN(0.05) POUT(0.10) ITERATE(20) CUT(0.5).
Resources	Processor Time	00:00:00.02
	Elapsed Time	00:00:00.06

Case Processing Summary

Unweighted Cases[a]		N	Percent
Selected Cases	Included in Analysis	115	97.5
	Missing Cases	3	2.5
	Total	118	100.0
Unselected Cases		0	.0
Total		118	100.0

a. If weight is in effect, see classification table for the total number of cases.

Dependent Variable Encoding

Original Value	Internal Value
No	0
Yes	1

Categorical Variables Codings

		Frequency	Parameter coding		
			(1)	(2)	(3)
BMINOO	99	6	.000	.000	.000
	Normal	80	1.000	.000	.000
	Overweight	20	.000	1.000	.000
	Obese	9	.000	.000	1.000
Race	Malay	91	.000	.000	
	Chinese	16	1.000	.000	
	Indian	8	.000	1.000	
Sex	Male	48	1.000		
	Female	67	.000		
MaritalSM	Single	90	.000		
	Married	25	1.000		
Occupation2	Student	59	.000		
	Employee	56	1.000		
FollowupYN	One	77	.000		
	More than one	38	1.000		
MedicalIssue	No	106	.000		
	Yes	9	1.000		
ComplicationYN	No	100	.000		
	Yes	15	1.000		
DurationOfSurgery	Median & below	66	.000		
	Above median	49	1.000		

Categorical Variables Codings

		Frequency	Parameter coding (1)	(2)	(3)
Sympathectomy.Level	T2-T3	67	.000		
	T2-T4	48	1.000		
PatientPosition	Lateral	14	.000		
	Supine?/Semi upright	101	1.000		
AgeMedian	Median & below	59	1.000		
	Above median	56	.000		

Block 0: Beginning Block

Classification Table[a,b]

Observed			Predicted CS No	Predicted CS Yes	Predicted Percentage Correct
Step 0	CS	No	0	48	.0
		Yes	0	67	100.0
	Overall Percentage				58.3

a. Constant is included in the model.

b. The cut value is .500

Variables in the Equation

		B	S.E.	Wald	df	Sig.	Exp(B)
Step 0	Constant	.333	.189	3.110	1	.078	1.396

Variables not in the Equation

			Score	df	Sig.
Step 0	Variables	AgeMedian(1)	.056	1	.813
		Sex(1)	1.293	1	.256
		Race	.995	2	.608
		Race(1)	.031	1	.860
		Race(2)	.991	1	.320
		MaritalSM(1)	.040	1	.842
		Occupation2(1)	.056	1	.813
		BMINOO	.708	3	.871
		BMINOO(1)	.437	1	.509
		BMINOO(2)	.452	1	.501
		BMINOO(3)	.029	1	.864
		MedicalIssue(1)	1.529	1	.216
		PatientPosition(1)	.238	1	.626
		Sympathectomy.Level(1)	3.625	1	.057
		DurationOfSurgery(1)	.350	1	.554
		ComplicationYN(1)	.954	1	.329
		FollowupYN(1)	22.737	1	.000
	Overall Statistics		31.076	15	.009

Block 1: Method = Enter

Omnibus Tests of Model Coefficients

		Chi-square	df	Sig.
Step 1	Step	37.345	15	.001
	Block	37.345	15	.001
	Model	37.345	15	.001

Model Summary

Step	-2 Log likelihood	Cox & Snell R Square	Nagelkerke R Square
1	118.925[a]	.277	.373

a. Estimation terminated at iteration number 5 because parameter estimates changed by less than .001.

Hosmer and Lemeshow Test

Step	Chi-square	df	Sig.
1	7.605	7	.369

Contingency Table for Hosmer and Lemeshow Test

		CS = No		CS = Yes		
		Observed	Expected	Observed	Expected	Total
Step 1	1	9	9.626	3	2.374	12
	2	9	8.705	3	3.295	12
	3	9	8.120	4	4.880	13
	4	7	6.314	5	5.686	12
	5	7	5.608	5	6.392	12
	6	2	4.868	10	7.132	12
	7	2	2.742	10	9.258	12
	8	3	1.307	9	10.693	12
	9	0	.709	18	17.291	18

Classification Table[a]

Observed			Predicted CS No	Predicted CS Yes	Percentage Correct
Step 1	CS	No	32	16	66.7
		Yes	14	53	79.1
	Overall Percentage				73.9

a. The cut value is .500

Variables in the Equation

		B	S.E.	Wald	df	Sig.	Exp(B)	95% C.I.for EXP(B)	
								Lower	Upper
Step 1[a]	AgeMedian(1)	-.380	.827	.211	1	.646	.684	.135	3.461
	Sex(1)	.125	.486	.066	1	.797	1.133	.437	2.935
	Race			.799	2	.671			
	Race(1)	-.046	.695	.004	1	.947	.955	.245	3.727
	Race(2)	.946	1.067	.786	1	.375	2.574	.318	20.830
	MaritalSM(1)	-.325	.713	.208	1	.649	.723	.179	2.924
	Occupation2(1)	-.486	.806	.363	1	.547	.615	.127	2.988
	BMINOO			.852	3	.837			
	BMINOO(1)	-.382	1.278	.089	1	.765	.682	.056	8.360
	BMINOO(2)	.223	1.406	.025	1	.874	1.250	.079	19.663
	BMINOO(3)	-.488	1.514	.104	1	.747	.614	.032	11.928
	MedicalIssue(1)	1.458	.989	2.173	1	.140	4.298	.618	29.869
	PatientPosition(1)	.077	.804	.009	1	.924	1.080	.223	5.219
	Sympathectomy.Level(1)	-1.356	.539	6.318	1	.012	.258	.090	.742
	DurationOfSurgery(1)	.052	.517	.010	1	.920	1.053	.383	2.901
	ComplicationYN(1)	-.944	.744	1.607	1	.205	.389	.090	1.674
	FollowupYN(1)	2.805	.670	17.532	1	.000	16.531	4.447	61.458
	Constant	.822	1.877	.192	1	.661	2.275		

a. Variable(s) entered on step 1: AgeMedian, Sex, Race, MaritalSM, Occupation2, BMINOO, MedicalIssue, PatientPosition, Sympathectomy.Level, DurationOfSurgery, ComplicationYN, FollowupYN.

```
LOGISTIC REGRESSION VARIABLES Compensatory.sweating
  /METHOD=ENTER AgeMedian
  /CONTRAST (AgeMedian)=Indicator
  /PRINT=GOODFIT CI(95)
  /CRITERIA=PIN(0.05) POUT(0.10) ITERATE(20) CUT(0.5).
```

Logistic Regression

Notes

Output Created		18-APR-2018 18:43:...
Comments		
Input	Data	C:\Users\rnordin.ADMIN\Desktop\2018\PUBLICATION 2018 ETS\ETS.Data (Complete).sav 18APRIL2018.sav
	Active Dataset	DataSet1
	Filter	<none>
	Weight	<none>
	Split File	<none>
	N of Rows in Working Data File	118
Missing Value Handling	Definition of Missing	User-defined missing values are treated as missing
Syntax		LOGISTIC REGRESSION VARIABLES Compensatory.sweating /METHOD=ENTER AgeMedian /CONTRAST (AgeMedian)=Indicator /PRINT=GOODFIT CI (95) /CRITERIA=PIN(0.05) POUT(0.10) ITERATE (20) CUT(0.5).
Resources	Processor Time	00:00:00.02
	Elapsed Time	00:00:00.01

Case Processing Summary

Unweighted Cases[a]		N	Percent
Selected Cases	Included in Analysis	118	100.0
	Missing Cases	0	.0
	Total	118	100.0
Unselected Cases		0	.0
Total		118	100.0

a. If weight is in effect, see classification table for the total number of cases.

Dependent Variable Encoding

Original Value	Internal Value
No	0
Yes	1

Categorical Variables Codings

		Frequency	Parameter coding (1)
AgeMedian	Median & below	60	1.000
	Above median	58	.000

Block 0: Beginning Block

Classification Table[a,b]

Observed			Predicted CS No	Predicted CS Yes	Percentage Correct
Step 0	CS	No	0	50	.0
		Yes	0	68	100.0
	Overall Percentage				57.6

a. Constant is included in the model.

b. The cut value is .500

Variables in the Equation

		B	S.E.	Wald	df	Sig.	Exp(B)
Step 0	Constant	.307	.186	2.724	1	.099	1.360

Variables not in the Equation

			Score	df	Sig.
Step 0	Variables	AgeMedian(1)	.025	1	.875
	Overall Statistics		.025	1	.875

Block 1: Method = Enter

Omnibus Tests of Model Coefficients

		Chi-square	df	Sig.
Step 1	Step	.025	1	.875
	Block	.025	1	.875
	Model	.025	1	.875

Model Summary

Step	-2 Log likelihood	Cox & Snell R Square	Nagelkerke R Square
1	160.801[a]	.000	.000

a. Estimation terminated at iteration number 3 because parameter estimates changed by less than .001.

Hosmer and Lemeshow Test

Step	Chi-square	df	Sig.
1	.000	0	.

Contingency Table for Hosmer and Lemeshow Test

		CS = No		CS = Yes		
		Observed	Expected	Observed	Expected	Total
Step 1	1	25	25.000	33	33.000	58
	2	25	25.000	35	35.000	60

Classification Table[a]

Observed			Predicted CS No	Predicted CS Yes	Percentage Correct
Step 1	CS	No	0	50	.0
		Yes	0	68	100.0
	Overall Percentage				57.6

a. The cut value is .500

Variables in the Equation

		B	S.E.	Wald	df	Sig.	Exp(B)	95% C.I.for EXP(B) Lower	Upper
Step 1[a]	AgeMedian(1)	.059	.373	.025	1	.875	1.061	.511	2.202
	Constant	.278	.265	1.096	1	.295	1.320		

a. Variable(s) entered on step 1: AgeMedian.

```
LOGISTIC REGRESSION VARIABLES Compensatory.sweating
 /METHOD=ENTER Sex
 /CONTRAST (Sex)=Indicator
 /PRINT=GOODFIT CI(95)
 /CRITERIA=PIN(0.05) POUT(0.10) ITERATE(20) CUT(0.5).
```

Logistic Regression

Notes

Output Created		18-APR-2018 18:43:...
Comments		
Input	Data	C:\Users\rnordin.ADMIN\Desktop\2018\PUBLICATION 2018 ETS\ETS.Data (Complete).sav 18APRIL2018.sav
	Active Dataset	DataSet1
	Filter	<none>
	Weight	<none>
	Split File	<none>
	N of Rows in Working Data File	118
Missing Value Handling	Definition of Missing	User-defined missing values are treated as missing
Syntax		LOGISTIC REGRESSION VARIABLES Compensatory.sweating /METHOD=ENTER Sex /CONTRAST (Sex)=Indicator /PRINT=GOODFIT CI(95) /CRITERIA=PIN(0.05) POUT(0.10) ITERATE(20) CUT(0.5).
Resources	Processor Time	00:00:00.00
	Elapsed Time	00:00:00.02

Case Processing Summary

Unweighted Cases[a]		N	Percent
Selected Cases	Included in Analysis	118	100.0
	Missing Cases	0	.0
	Total	118	100.0
Unselected Cases		0	.0
Total		118	100.0

a. If weight is in effect, see classification table for the total number of cases.

Dependent Variable Encoding

Original Value	Internal Value
No	0
Yes	1

Categorical Variables Codings

		Frequency	Parameter coding (1)
Sex	Male	50	1.000
	Female	68	.000

Block 0: Beginning Block

Classification Table[a,b]

Observed			Predicted CS No	Predicted CS Yes	Percentage Correct
Step 0	CS	No	0	50	.0
		Yes	0	68	100.0
	Overall Percentage				57.6

a. Constant is included in the model.

b. The cut value is .500

Variables in the Equation

		B	S.E.	Wald	df	Sig.	Exp(B)
Step 0	Constant	.307	.186	2.724	1	.099	1.360

Variables not in the Equation

			Score	df	Sig.
Step 0	Variables	Sex(1)	1.125	1	.289
	Overall Statistics		1.125	1	.289

Block 1: Method = Enter

Omnibus Tests of Model Coefficients

		Chi-square	df	Sig.
Step 1	Step	1.124	1	.289
	Block	1.124	1	.289
	Model	1.124	1	.289

Model Summary

Step	-2 Log likelihood	Cox & Snell R Square	Nagelkerke R Square
1	159.702[a]	.009	.013

a. Estimation terminated at iteration number 3 because parameter estimates changed by less than .001.

Hosmer and Lemeshow Test

Step	Chi-square	df	Sig.
1	.000	0	.

Contingency Table for Hosmer and Lemeshow Test

		CS = No		CS = Yes		
		Observed	Expected	Observed	Expected	Total
Step 1	1	24	24.000	26	26.000	50
	2	26	26.000	42	42.000	68

Classification Table[a]

Observed			Predicted CS No	Predicted CS Yes	Percentage Correct
Step 1	CS	No	0	50	.0
		Yes	0	68	100.0
	Overall Percentage				57.6

a. The cut value is .500

Variables in the Equation

		B	S.E.	Wald	df	Sig.	Exp(B)	95% C.I.for EXP(B) Lower	Upper
Step 1[a]	Sex(1)	-.400	.377	1.121	1	.290	.671	.320	1.405
	Constant	.480	.250	3.693	1	.055	1.615		

a. Variable(s) entered on step 1: Sex.

```
LOGISTIC REGRESSION VARIABLES Compensatory.sweating
 /METHOD=ENTER Race
 /CONTRAST (Race)=Indicator(1)
 /PRINT=GOODFIT CI(95)
```

Logistic Regression

Notes

Output Created		18-APR-2018 18:44:...
Comments		
Input	Data	C:\Users\rnordin.ADMIN\Desktop\2018\PUBLICATION 2018 ETS\ETS.Data (Complete).sav 18APRIL2018.sav
	Active Dataset	DataSet1
	Filter	<none>
	Weight	<none>
	Split File	<none>
	N of Rows in Working Data File	118
Missing Value Handling	Definition of Missing	User-defined missing values are treated as missing
Syntax		LOGISTIC REGRESSION VARIABLES Compensatory.sweating /METHOD=ENTER Race /CONTRAST (Race)=Indicator(1) /PRINT=GOODFIT CI(95) /CRITERIA=PIN(0.05) POUT(0.10) ITERATE(20) CUT(0.5).
Resources	Processor Time	00:00:00.02
	Elapsed Time	00:00:00.02

Case Processing Summary

Unweighted Cases[a]		N	Percent
Selected Cases	Included in Analysis	118	100.0
	Missing Cases	0	.0
	Total	118	100.0
Unselected Cases		0	.0
Total		118	100.0

a. If weight is in effect, see classification table for the total number of cases.

Dependent Variable Encoding

Original Value	Internal Value
No	0
Yes	1

Categorical Variables Codings

		Frequency	Parameter coding (1)	(2)
Race	Malay	94	.000	.000
	Chinese	16	1.000	.000
	Indian	8	.000	1.000

Block 0: Beginning Block

Classification Table[a,b]

Observed			Predicted CS No	Predicted CS Yes	Percentage Correct
Step 0	CS	No	0	50	.0
		Yes	0	68	100.0
	Overall Percentage				57.6

a. Constant is included in the model.

b. The cut value is .500

Variables in the Equation

		B	S.E.	Wald	df	Sig.	Exp(B)
Step 0	Constant	.307	.186	2.724	1	.099	1.360

Variables not in the Equation

			Score	df	Sig.
Step 0	Variables	Race	1.061	2	.588
		Race(1)	.014	1	.905
		Race(2)	1.061	1	.303
	Overall Statistics		1.061	2	.588

Block 1: Method = Enter

Omnibus Tests of Model Coefficients

		Chi-square	df	Sig.
Step 1	Step	1.123	2	.570
	Block	1.123	2	.570
	Model	1.123	2	.570

Model Summary

Step	-2 Log likelihood	Cox & Snell R Square	Nagelkerke R Square
1	159.703[a]	.009	.013

a. Estimation terminated at iteration number 4 because parameter estimates changed by less than .001.

Hosmer and Lemeshow Test

Step	Chi-square	df	Sig.
1	.000	1	1.000

Contingency Table for Hosmer and Lemeshow Test

		CS = No		CS = Yes		
		Observed	Expected	Observed	Expected	Total
Step 1	1	7	7.000	9	9.000	16
	2	41	41.000	53	53.000	94
	3	2	2.000	6	6.000	8

Classification Table[a]

Observed			Predicted		
			CS		Percentage Correct
			No	Yes	
Step 1	CS	No	0	50	.0
		Yes	0	68	100.0
	Overall Percentage				57.6

a. The cut value is .500

Variables in the Equation

		B	S.E.	Wald	df	Sig.	Exp(B)	95% C.I.for EXP(B)	
								Lower	Upper
Step 1[a]	Race			1.009	2	.604			
	Race(1)	-.005	.545	.000	1	.992	.995	.342	2.895
	Race(2)	.842	.843	.998	1	.318	2.321	.445	12.101
	Constant	.257	.208	1.524	1	.217	1.293		

a. Variable(s) entered on step 1: Race.

```
LOGISTIC REGRESSION VARIABLES Compensatory.sweating
 /METHOD=ENTER Marital.Status
 /CONTRAST (Marital.Status)=Indicator(1)
 /PRINT=GOODFIT CI(95)
 /CRITERIA=PIN(0.05) POUT(0.10) ITERATE(20) CUT(0.5).
```

Logistic Regression

Notes

Output Created		18-APR-2018 18:45:...
Comments		
Input	Data	C:\Users\rnordin.ADMIN\Desktop\2018\PUBLICATION 2018 ETS\ETS.Data (Complete).sav 18APRIL2018.sav
	Active Dataset	DataSet1
	Filter	<none>
	Weight	<none>
	Split File	<none>
	N of Rows in Working Data File	118
Missing Value Handling	Definition of Missing	User-defined missing values are treated as missing
Syntax		LOGISTIC REGRESSION VARIABLES Compensatory.sweating /METHOD=ENTER Marital.Status /CONTRAST (Marital.Status)=Indicator(1) /PRINT=GOODFIT CI (95) /CRITERIA=PIN(0.05) POUT(0.10) ITERATE(20) CUT(0.5).
Resources	Processor Time	00:00:00.02
	Elapsed Time	00:00:00.02

Case Processing Summary

Unweighted Cases[a]		N	Percent
Selected Cases	Included in Analysis	118	100.0
	Missing Cases	0	.0
	Total	118	100.0
Unselected Cases		0	.0
Total		118	100.0

a. If weight is in effect, see classification table for the total number of cases.

Dependent Variable Encoding

Original Value	Internal Value
No	0
Yes	1

Categorical Variables Codings

		Frequency	Parameter coding (1)
MaritalSM	Single	93	.000
	Married	25	1.000

Block 0: Beginning Block

Classification Table[a,b]

Observed			Predicted CS No	Predicted CS Yes	Percentage Correct
Step 0	CS	No	0	50	.0
		Yes	0	68	100.0
	Overall Percentage				57.6

a. Constant is included in the model.

b. The cut value is .500

Variables in the Equation

		B	S.E.	Wald	df	Sig.	Exp(B)
Step 0	Constant	.307	.186	2.724	1	.099	1.360

Variables not in the Equation

			Score	df	Sig.
Step 0	Variables	MaritalSM(1)	.073	1	.787
	Overall Statistics		.073	1	.787

Block 1: Method = Enter

Omnibus Tests of Model Coefficients

		Chi-square	df	Sig.
Step 1	Step	.073	1	.786
	Block	.073	1	.786
	Model	.073	1	.786

Model Summary

Step	-2 Log likelihood	Cox & Snell R Square	Nagelkerke R Square
1	160.753[a]	.001	.001

a. Estimation terminated at iteration number 3 because parameter estimates changed by less than .001.

Hosmer and Lemeshow Test

Step	Chi-square	df	Sig.
1	.000	0	.

Contingency Table for Hosmer and Lemeshow Test

		CS = No		CS = Yes		
		Observed	Expected	Observed	Expected	Total
Step 1	1	40	40.000	53	53.000	93
	2	10	10.000	15	15.000	25

Classification Table[a]

			Predicted		
			CS		Percentage Correct
Observed			No	Yes	
Step 1	CS	No	0	50	.0
		Yes	0	68	100.0
	Overall Percentage				57.6

a. The cut value is .500

Variables in the Equation

		B	S.E.	Wald	df	Sig.	Exp(B)	95% C.I.for EXP(B)	
								Lower	Upper
Step 1[a]	MaritalSM(1)	.124	.459	.073	1	.787	1.132	.461	2.783
	Constant	.281	.209	1.805	1	.179	1.325		

a. Variable(s) entered on step 1: MaritalSM.

```
LOGISTIC REGRESSION VARIABLES Compensatory.sweating
 /METHOD=ENTER Occupation2
 /CONTRAST (Occupation2)=Indicator(1)
 /PRINT=GOODFIT CI(95)
 /CRITERIA=PIN(0.05) POUT(0.10) ITERATE(20) CUT(0.5).
```

Logistic Regression

Notes

Output Created		18-APR-2018 18:45:...
Comments		
Input	Data	C:\Users\rnordin.ADMIN\Desktop\2018\PUBLICATION 2018 ETS\ETS.Data (Complete).sav 18APRIL2018.sav
	Active Dataset	DataSet1
	Filter	<none>
	Weight	<none>
	Split File	<none>
	N of Rows in Working Data File	118
Missing Value Handling	Definition of Missing	User-defined missing values are treated as missing
Syntax		LOGISTIC REGRESSION VARIABLES Compensatory.sweating /METHOD=ENTER Occupation2 /CONTRAST (Occupation2)=Indicator(1) /PRINT=GOODFIT CI(95) /CRITERIA=PIN(0.05) POUT(0.10) ITERATE(20) CUT(0.5).
Resources	Processor Time	00:00:00.03
	Elapsed Time	00:00:00.02

Case Processing Summary

Unweighted Cases[a]		N	Percent
Selected Cases	Included in Analysis	118	100.0
	Missing Cases	0	.0
	Total	118	100.0
Unselected Cases		0	.0
Total		118	100.0

a. If weight is in effect, see classification table for the total number of cases.

Dependent Variable Encoding

Original Value	Internal Value
No	0
Yes	1

Categorical Variables Codings

		Frequency	Parameter coding (1)
Occupation2	Student	60	.000
	Employee	58	1.000

Block 0: Beginning Block

Classification Table[a,b]

Observed			Predicted CS No	Predicted CS Yes	Percentage Correct
Step 0	CS	No	0	50	.0
		Yes	0	68	100.0
	Overall Percentage				57.6

a. Constant is included in the model.

b. The cut value is .500

Variables in the Equation

		B	S.E.	Wald	df	Sig.	Exp(B)
Step 0	Constant	.307	.186	2.724	1	.099	1.360

Variables not in the Equation

			Score	df	Sig.
Step 0	Variables	Occupation2(1)	.025	1	.875
	Overall Statistics		.025	1	.875

Block 1: Method = Enter

Omnibus Tests of Model Coefficients

		Chi-square	df	Sig.
Step 1	Step	.025	1	.875
	Block	.025	1	.875
	Model	.025	1	.875

Model Summary

Step	-2 Log likelihood	Cox & Snell R Square	Nagelkerke R Square
1	160.801[a]	.000	.000

a. Estimation terminated at iteration number 3 because parameter estimates changed by less than .001.

Hosmer and Lemeshow Test

Step	Chi-square	df	Sig.
1	.000	0	.

Contingency Table for Hosmer and Lemeshow Test

		CS = No		CS = Yes		
		Observed	Expected	Observed	Expected	Total
Step 1	1	25	25.000	33	33.000	58
	2	25	25.000	35	35.000	60

Classification Table[a]

Observed			Predicted: CS No	Predicted: CS Yes	Percentage Correct
Step 1	CS	No	0	50	.0
		Yes	0	68	100.0
	Overall Percentage				57.6

a. The cut value is .500

Variables in the Equation

		B	S.E.	Wald	df	Sig.	Exp(B)	95% C.I.for EXP(B)	
								Lower	Upper
Step 1[a]	Occupation2(1)	-.059	.373	.025	1	.875	.943	.454	1.957
	Constant	.336	.262	1.651	1	.199	1.400		

a. Variable(s) entered on step 1: Occupation2.

```
LOGISTIC REGRESSION VARIABLES Compensatory.sweating
 /METHOD=ENTER BMINOO
 /CONTRAST (BMINOO)=Indicator(1)
 /PRINT=GOODFIT CI(95)
 /CRITERIA=PIN(0.05) POUT(0.10) ITERATE(20) CUT(0.5).
```

Logistic Regression

Notes

Output Created		18-APR-2018 18:45:...
Comments		
Input	Data	C:\Users\rnordin.ADMIN\Desktop\2018\PUBLICATION 2018 ETS\ETS.Data (Complete).sav 18APRIL2018.sav
	Active Dataset	DataSet1
	Filter	<none>
	Weight	<none>
	Split File	<none>
	N of Rows in Working Data File	118
Missing Value Handling	Definition of Missing	User-defined missing values are treated as missing
Syntax		LOGISTIC REGRESSION VARIABLES Compensatory.sweating /METHOD=ENTER BMINOO /CONTRAST (BMINOO)=Indicator(1) /PRINT=GOODFIT CI(95) /CRITERIA=PIN(0.05) POUT(0.10) ITERATE(20) CUT(0.5).
Resources	Processor Time	00:00:00.02
	Elapsed Time	00:00:00.01

Case Processing Summary

Unweighted Cases[a]		N	Percent
Selected Cases	Included in Analysis	118	100.0
	Missing Cases	0	.0
	Total	118	100.0
Unselected Cases		0	.0
Total		118	100.0

a. If weight is in effect, see classification table for the total number of cases.

Dependent Variable Encoding

Original Value	Internal Value
No	0
Yes	1

Categorical Variables Codings

		Frequency	Parameter coding (1)	(2)	(3)
BMINOO	99	6	.000	.000	.000
	Normal	82	1.000	.000	.000
	Overweight	20	.000	1.000	.000
	Obese	10	.000	.000	1.000

Block 0: Beginning Block

Classification Table[a,b]

Observed			Predicted CS No	Yes	Percentage Correct
Step 0	CS	No	0	50	.0
		Yes	0	68	100.0
	Overall Percentage				57.6

a. Constant is included in the model.

b. The cut value is .500

Variables in the Equation

		B	S.E.	Wald	df	Sig.	Exp(B)
Step 0	Constant	.307	.186	2.724	1	.099	1.360

Variables not in the Equation

			Score	df	Sig.
Step 0	Variables	BMINOO	.963	3	.810
		BMINOO(1)	.258	1	.612
		BMINOO(2)	.536	1	.464
		BMINOO(3)	.260	1	.610
	Overall Statistics		.963	3	.810

Block 1: Method = Enter

Omnibus Tests of Model Coefficients

		Chi-square	df	Sig.
Step 1	Step	.974	3	.808
	Block	.974	3	.808
	Model	.974	3	.808

Model Summary

Step	-2 Log likelihood	Cox & Snell R Square	Nagelkerke R Square
1	159.853[a]	.008	.011

a. Estimation terminated at iteration number 3 because parameter estimates changed by less than .001.

Hosmer and Lemeshow Test

Step	Chi-square	df	Sig.
1	.000	2	1.000

Contingency Table for Hosmer and Lemeshow Test

		CS = No		CS = Yes		
		Observed	Expected	Observed	Expected	Total
Step 1	1	5	5.000	5	5.000	10
	2	36	36.000	46	46.000	82
	3	7	7.000	13	13.000	20
	4	2	2.000	4	4.000	6

Classification Table[a]

Observed			Predicted CS No	Predicted CS Yes	Percentage Correct
Step 1	CS	No	0	50	.0
		Yes	0	68	100.0
	Overall Percentage				57.6

a. The cut value is .500

Variables in the Equation

		B	S.E.	Wald	df	Sig.	Exp(B)	95% C.I.for EXP(B) Lower	95% C.I.for EXP(B) Upper
Step 1[a]	BMINOO			.955	3	.812			
	BMINOO(1)	-.448	.894	.251	1	.616	.639	.111	3.686
	BMINOO(2)	-.074	.985	.006	1	.940	.929	.135	6.398
	BMINOO(3)	-.693	1.072	.418	1	.518	.500	.061	4.091
	Constant	.693	.866	.641	1	.423	2.000		

a. Variable(s) entered on step 1: BMINOO.

```
LOGISTIC REGRESSION VARIABLES Compensatory.sweating
 /METHOD=ENTER Location.of.PHH
 /PRINT=GOODFIT CI(95)
 /CRITERIA=PIN(0.05) POUT(0.10) ITERATE(20) CUT(0.5).
```

Logistic Regression

Notes

Output Created		18-APR-2018 18:46:...
Comments		
Input	Data	C:\Users\rnordin.ADMIN\Desktop\2018\PUBLICATION 2018 ETS\ETS.Data (Complete).sav 18APRIL2018.sav
	Active Dataset	DataSet1
	Filter	<none>
	Weight	<none>
	Split File	<none>
	N of Rows in Working Data File	118
Missing Value Handling	Definition of Missing	User-defined missing values are treated as missing
Syntax		LOGISTIC REGRESSION VARIABLES Compensatory.sweating /METHOD=ENTER Location.of.PHH /PRINT=GOODFIT CI (95) /CRITERIA=PIN(0.05) POUT(0.10) ITERATE (20) CUT(0.5).
Resources	Processor Time	00:00:00.02
	Elapsed Time	00:00:00.02

Case Processing Summary

Unweighted Cases[a]		N	Percent
Selected Cases	Included in Analysis	118	100.0
	Missing Cases	0	.0
	Total	118	100.0
Unselected Cases		0	.0
Total		118	100.0

a. If weight is in effect, see classification table for the total number of cases.

Dependent Variable Encoding

Original Value	Internal Value
No	0
Yes	1

Block 0: Beginning Block

Classification Table[a,b]

Observed			Predicted CS No	Predicted CS Yes	Percentage Correct
Step 0	CS	No	0	50	.0
		Yes	0	68	100.0
	Overall Percentage				57.6

a. Constant is included in the model.

b. The cut value is .500

Variables in the Equation

		B	S.E.	Wald	df	Sig.	Exp(B)
Step 0	Constant	.307	.186	2.724	1	.099	1.360

Variables not in the Equation

			Score	df	Sig.
Step 0	Variables	Location.of.PHH	.383	1	.536
	Overall Statistics		.383	1	.536

Block 1: Method = Enter

Omnibus Tests of Model Coefficients

		Chi-square	df	Sig.
Step 1	Step	.384	1	.536
	Block	.384	1	.536
	Model	.384	1	.536

Model Summary

Step	-2 Log likelihood	Cox & Snell R Square	Nagelkerke R Square
1	160.442[a]	.003	.004

a. Estimation terminated at iteration number 3 because parameter estimates changed by less than .001.

Hosmer and Lemeshow Test

Step	Chi-square	df	Sig.
1	.421	2	.810

Contingency Table for Hosmer and Lemeshow Test

		CS = No		CS = Yes		
		Observed	Expected	Observed	Expected	Total
Step 1	1	5	5.256	6	5.744	11
	2	14	14.174	18	17.826	32
	3	21	19.457	25	26.543	46
	4	10	11.114	19	17.886	29

Classification Table[a]

Observed			Predicted		
			CS		Percentage Correct
			No	Yes	
Step 1	CS	No	0	50	.0
		Yes	1	67	98.5
	Overall Percentage				56.8

a. The cut value is .500

Variables in the Equation

		B	S.E.	Wald	df	Sig.	Exp(B)	95% C.I.for EXP(B)	
								Lower	Upper
Step 1[a]	Location.of.PHH	-.081	.131	.382	1	.536	.922	.713	1.193
	Constant	.798	.817	.954	1	.329	2.221		

a. Variable(s) entered on step 1: Location.of.PHH.

```
LOGISTIC REGRESSION VARIABLES Compensatory.sweating
 /METHOD=ENTER Thyroid.Function
 /PRINT=GOODFIT CI(95)
 /CRITERIA=PIN(0.05) POUT(0.10) ITERATE(20) CUT(0.5).
```

Logistic Regression

Notes

Output Created		18-APR-2018 18:46:...
Comments		
Input	Data	C:\Users\rnordin.ADMIN\Desktop\2018\PUBLICATION 2018 ETS\ETS.Data (Complete).sav 18APRIL2018.sav
	Active Dataset	DataSet1
	Filter	<none>
	Weight	<none>
	Split File	<none>
	N of Rows in Working Data File	118
Missing Value Handling	Definition of Missing	User-defined missing values are treated as missing
Syntax		LOGISTIC REGRESSION VARIABLES Compensatory.sweating /METHOD=ENTER Thyroid.Function /PRINT=GOODFIT CI (95) /CRITERIA=PIN(0.05) POUT(0.10) ITERATE (20) CUT(0.5).
Resources	Processor Time	00:00:00.02
	Elapsed Time	00:00:00.02

Case Processing Summary

Unweighted Cases[a]		N	Percent
Selected Cases	Included in Analysis	118	100.0
	Missing Cases	0	.0
	Total	118	100.0
Unselected Cases		0	.0
Total		118	100.0

a. If weight is in effect, see classification table for the total number of cases.

Dependent Variable Encoding

Original Value	Internal Value
No	0
Yes	1

Block 0: Beginning Block

Classification Table[a,b]

Observed			Predicted CS No	Predicted CS Yes	Percentage Correct
Step 0	CS	No	0	50	.0
		Yes	0	68	100.0
	Overall Percentage				57.6

a. Constant is included in the model.

b. The cut value is .500

Variables in the Equation

		B	S.E.	Wald	df	Sig.	Exp(B)
Step 0	Constant	.307	.186	2.724	1	.099	1.360

Block 1: Method = Enter

Model Summary

Step	-2 Log likelihood	Cox & Snell R Square	Nagelkerke R Square
1	160.826[a]	.000	.000

a. Estimation terminated at iteration number 3 because parameter estimates changed by less than .001.

Hosmer and Lemeshow Test

Step	Chi-square	df	Sig.
1	.000	0	.

Contingency Table for Hosmer and Lemeshow Test

		CS = No		CS = Yes		
		Observed	Expected	Observed	Expected	Total
Step 1	1	50	50.000	68	68.000	118

Classification Table[a]

Observed			Predicted CS No	Predicted CS Yes	Percentage Correct
Step 1	CS	No	0	50	.0
		Yes	0	68	100.0
	Overall Percentage				57.6

a. The cut value is .500

Variables in the Equation

		B	S.E.	Wald	df	Sig.	Exp(B)
Step 1	Constant	.307	.186	2.724	1	.099	1.360

```
LOGISTIC REGRESSION VARIABLES Compensatory.sweating
  /METHOD=ENTER Diabetes
  /PRINT=GOODFIT CI(95)
  /CRITERIA=PIN(0.05) POUT(0.10) ITERATE(20) CUT(0.5).
```

Logistic Regression

Notes

Output Created		18-APR-2018 18:47:...
Comments		
Input	Data	C:\Users\rnordin.ADMIN\Desktop\2018\PUBLICATION 2018 ETS\ETS.Data (Complete).sav 18APRIL2018.sav
	Active Dataset	DataSet1
	Filter	<none>
	Weight	<none>
	Split File	<none>
	N of Rows in Working Data File	118
Missing Value Handling	Definition of Missing	User-defined missing values are treated as missing

Notes

Syntax		LOGISTIC REGRESSION VARIABLES Compensatory.sweating /METHOD=ENTER Diabetes /PRINT=GOODFIT CI (95) /CRITERIA=PIN(0.05) POUT(0.10) ITERATE (20) CUT(0.5).
Resources	Processor Time	00:00:00.00
	Elapsed Time	00:00:00.01

Case Processing Summary

Unweighted Cases[a]		N	Percent
Selected Cases	Included in Analysis	118	100.0
	Missing Cases	0	.0
	Total	118	100.0
Unselected Cases		0	.0
Total		118	100.0

a. If weight is in effect, see classification table for the total number of cases.

Dependent Variable Encoding

Original Value	Internal Value
No	0
Yes	1

Block 0: Beginning Block

Classification Table[a,b]

Observed			Predicted CS No	Predicted CS Yes	Percentage Correct
Step 0	CS	No	0	50	.0
		Yes	0	68	100.0
	Overall Percentage				57.6

a. Constant is included in the model.

b. The cut value is .500

Variables in the Equation

		B	S.E.	Wald	df	Sig.	Exp(B)
Step 0	Constant	.307	.186	2.724	1	.099	1.360

Variables not in the Equation

			Score	df	Sig.
Step 0	Variables	Diabetes	.742	1	.389
	Overall Statistics		.742	1	.389

Block 1: Method = Enter

Omnibus Tests of Model Coefficients

		Chi-square	df	Sig.
Step 1	Step	1.109	1	.292
	Block	1.109	1	.292
	Model	1.109	1	.292

Model Summary

Step	-2 Log likelihood	Cox & Snell R Square	Nagelkerke R Square
1	159.718[a]	.009	.013

a. Estimation terminated at iteration number 20 because maximum iterations has been reached. Final solution cannot be found.

Hosmer and Lemeshow Test

Step	Chi-square	df	Sig.
1	.000	0	.

Contingency Table for Hosmer and Lemeshow Test

		CS = No		CS = Yes		Total
		Observed	Expected	Observed	Expected	
Step 1	1	50	50.000	68	68.000	118

Classification Table[a]

Observed			Predicted CS No	Predicted CS Yes	Percentage Correct
Step 1	CS	No	0	50	.0
		Yes	0	68	100.0
	Overall Percentage				57.6

a. The cut value is .500

Variables in the Equation

		B	S.E.	Wald	df	Sig.	Exp(B)	95% C.I.for EXP(B) Lower	95% C.I.for EXP(B) Upper
Step 1[a]	Diabetes	-20.910	40192.126	.000	1	1.000	.000	.000	.
	Constant	42.113	80384.252	.000	1	1.000	1.948E+18		

a. Variable(s) entered on step 1: Diabetes.

```
LOGISTIC REGRESSION VARIABLES Compensatory.sweating
 /METHOD=ENTER Medical.issues
 /CONTRAST (Medical.issues)=Indicator(1)
 /PRINT=GOODFIT CI(95)
 /CRITERIA=PIN(0.05) POUT(0.10) ITERATE(20) CUT(0.5).
```

Logistic Regression

Notes

Output Created		18-APR-2018 18:47:...
Comments		
Input	Data	C:\Users\rnordin.ADMIN\Desktop\2018\PUBLICATION 2018 ETS\ETS.Data (Complete).sav 18APRIL2018.sav
	Active Dataset	DataSet1
	Filter	<none>
	Weight	<none>
	Split File	<none>
	N of Rows in Working Data File	118
Missing Value Handling	Definition of Missing	User-defined missing values are treated as missing
Syntax		LOGISTIC REGRESSION VARIABLES Compensatory.sweating /METHOD=ENTER Medical.issues /CONTRAST (Medical.issues)=Indicator(1) /PRINT=GOODFIT CI(95) /CRITERIA=PIN(0.05) POUT(0.10) ITERATE(20) CUT(0.5).
Resources	Processor Time	00:00:00.02
	Elapsed Time	00:00:00.02

Case Processing Summary

Unweighted Cases[a]		N	Percent
Selected Cases	Included in Analysis	118	100.0
	Missing Cases	0	.0
	Total	118	100.0
Unselected Cases		0	.0
Total		118	100.0

a. If weight is in effect, see classification table for the total number of cases.

Dependent Variable Encoding

Original Value	Internal Value
No	0
Yes	1

Categorical Variables Codings

		Frequency	Parameter coding (1)
MedicalIssue	No	109	.000
	Yes	9	1.000

Block 0: Beginning Block

Classification Table[a,b]

Observed			Predicted CS No	Predicted CS Yes	Percentage Correct
Step 0	CS	No	0	50	.0
		Yes	0	68	100.0
	Overall Percentage				57.6

a. Constant is included in the model.

b. The cut value is .500

Variables in the Equation

		B	S.E.	Wald	df	Sig.	Exp(B)
Step 0	Constant	.307	.186	2.724	1	.099	1.360

Variables not in the Equation

			Score	df	Sig.
Step 0	Variables	MedicalIssue(1)	1.620	1	.203
	Overall Statistics		1.620	1	.203

Block 1: Method = Enter

Omnibus Tests of Model Coefficients

		Chi-square	df	Sig.
Step 1	Step	1.740	1	.187
	Block	1.740	1	.187
	Model	1.740	1	.187

Model Summary

Step	-2 Log likelihood	Cox & Snell R Square	Nagelkerke R Square
1	159.087[a]	.015	.020

a. Estimation terminated at iteration number 4 because parameter estimates changed by less than .001.

Hosmer and Lemeshow Test

Step	Chi-square	df	Sig.
1	.000	0	.

Contingency Table for Hosmer and Lemeshow Test

		CS = No		CS = Yes		
		Observed	Expected	Observed	Expected	Total
Step 1	1	48	48.000	61	61.000	109
	2	2	2.000	7	7.000	9

Classification Table[a]

Observed			Predicted CS No	Predicted CS Yes	Percentage Correct
Step 1	CS	No	0	50	.0
		Yes	0	68	100.0
	Overall Percentage				57.6

a. The cut value is .500

Variables in the Equation

		B	S.E.	Wald	df	Sig.	Exp(B)	95% C.I.for EXP(B) Lower	95% C.I.for EXP(B) Upper
Step 1[a]	MedicalIssue(1)	1.013	.825	1.509	1	.219	2.754	.547	13.866
	Constant	.240	.193	1.543	1	.214	1.271		

a. Variable(s) entered on step 1: MedicalIssue.

```
LOGISTIC REGRESSION VARIABLES Compensatory.sweating
 /METHOD=ENTER Patient.position
 /CONTRAST (Patient.position)=Indicator(1)
 /PRINT=GOODFIT CI(95)
 /CRITERIA=PIN(0.05) POUT(0.10) ITERATE(20) CUT(0.5).
```

Logistic Regression

Notes

Output Created		18-APR-2018 18:48:...
Comments		
Input	Data	C:\Users\rnordin.ADMIN\Desktop\2018\PUBLICATION 2018 ETS\ETS.Data (Complete).sav 18APRIL2018.sav
	Active Dataset	DataSet1
	Filter	<none>
	Weight	<none>
	Split File	<none>
	N of Rows in Working Data File	118
Missing Value Handling	Definition of Missing	User-defined missing values are treated as missing
Syntax		LOGISTIC REGRESSION VARIABLES Compensatory.sweating /METHOD=ENTER Patient.position /CONTRAST (Patient.position)=Indicator(1) /PRINT=GOODFIT CI (95) /CRITERIA=PIN(0.05) POUT(0.10) ITERATE(20) CUT(0.5).
Resources	Processor Time	00:00:00.00
	Elapsed Time	00:00:00.01

Case Processing Summary

Unweighted Cases[a]		N	Percent
Selected Cases	Included in Analysis	118	100.0
	Missing Cases	0	.0
	Total	118	100.0
Unselected Cases		0	.0
Total		118	100.0

a. If weight is in effect, see classification table for the total number of cases.

Dependent Variable Encoding

Original Value	Internal Value
No	0
Yes	1

Categorical Variables Codings

		Frequency	Parameter coding (1)
PatientPosition	Lateral	15	.000
	Supine?/Semi upright	103	1.000

Block 0: Beginning Block

Classification Table[a,b]

Observed			Predicted CS No	Predicted CS Yes	Percentage Correct
Step 0	CS	No	0	50	.0
		Yes	0	68	100.0
	Overall Percentage				57.6

a. Constant is included in the model.

b. The cut value is .500

Variables in the Equation

		B	S.E.	Wald	df	Sig.	Exp(B)
Step 0	Constant	.307	.186	2.724	1	.099	1.360

Variables not in the Equation

			Score	df	Sig.
Step 0	Variables	PatientPosition(1)	.575	1	.448
	Overall Statistics		.575	1	.448

Block 1: Method = Enter

Omnibus Tests of Model Coefficients

		Chi-square	df	Sig.
Step 1	Step	.588	1	.443
	Block	.588	1	.443
	Model	.588	1	.443

Model Summary

Step	-2 Log likelihood	Cox & Snell R Square	Nagelkerke R Square
1	160.239[a]	.005	.007

a. Estimation terminated at iteration number 3 because parameter estimates changed by less than .001.

Hosmer and Lemeshow Test

Step	Chi-square	df	Sig.
1	.000	0	.

Contingency Table for Hosmer and Lemeshow Test

		CS = No		CS = Yes		
		Observed	Expected	Observed	Expected	Total
Step 1	1	45	45.000	58	58.000	103
	2	5	5.000	10	10.000	15

Classification Table[a]

Observed			Predicted CS No	Predicted CS Yes	Percentage Correct
Step 1	CS	No	0	50	.0
		Yes	0	68	100.0
	Overall Percentage				57.6

a. The cut value is .500

Variables in the Equation

		B	S.E.	Wald	df	Sig.	Exp(B)	95% C.I.for EXP(B) Lower	95% C.I.for EXP(B) Upper
Step 1[a]	PatientPosition(1)	-.439	.583	.569	1	.451	.644	.206	2.019
	Constant	.693	.548	1.602	1	.206	2.000		

a. Variable(s) entered on step 1: PatientPosition.

```
LOGISTIC REGRESSION VARIABLES Compensatory.sweating
  /METHOD=ENTER Port.size
  /PRINT=GOODFIT CI(95)
  /CRITERIA=PIN(0.05) POUT(0.10) ITERATE(20) CUT(0.5).
```

Logistic Regression

Notes

Output Created		18-APR-2018 18:48:...
Comments		
Input	Data	C:\Users\rnordin.ADMIN\Desktop\2018\PUBLICATION 2018 ETS\ETS.Data (Complete).sav 18APRIL2018.sav
	Active Dataset	DataSet1
	Filter	<none>
	Weight	<none>
	Split File	<none>
	N of Rows in Working Data File	118
Missing Value Handling	Definition of Missing	User-defined missing values are treated as missing
Syntax		LOGISTIC REGRESSION VARIABLES Compensatory.sweating /METHOD=ENTER Port.size /PRINT=GOODFIT CI (95) /CRITERIA=PIN(0.05) POUT(0.10) ITERATE(20) CUT(0.5).
Resources	Processor Time	00:00:00.02
	Elapsed Time	00:00:00.02

Case Processing Summary

Unweighted Cases[a]		N	Percent
Selected Cases	Included in Analysis	118	100.0
	Missing Cases	0	.0
	Total	118	100.0
Unselected Cases		0	.0
Total		118	100.0

a. If weight is in effect, see classification table for the total number of cases.

Dependent Variable Encoding

Original Value	Internal Value
No	0
Yes	1

Block 0: Beginning Block

Classification Table[a,b]

Observed			Predicted CS No	Predicted CS Yes	Percentage Correct
Step 0	CS	No	0	50	.0
		Yes	0	68	100.0
	Overall Percentage				57.6

a. Constant is included in the model.

b. The cut value is .500

Variables in the Equation

		B	S.E.	Wald	df	Sig.	Exp(B)
Step 0	Constant	.307	.186	2.724	1	.099	1.360

Variables not in the Equation

			Score	df	Sig.
Step 0	Variables	Port.size	.048	1	.826
	Overall Statistics		.048	1	.826

Block 1: Method = Enter

Omnibus Tests of Model Coefficients

		Chi-square	df	Sig.
Step 1	Step	.048	1	.827
	Block	.048	1	.827
	Model	.048	1	.827

Model Summary

Step	-2 Log likelihood	Cox & Snell R Square	Nagelkerke R Square
1	160.778[a]	.000	.001

a. Estimation terminated at iteration number 3 because parameter estimates changed by less than .001.

Hosmer and Lemeshow Test

Step	Chi-square	df	Sig.
1	.000	0	.

Contingency Table for Hosmer and Lemeshow Test

		CS = No		CS = Yes		
		Observed	Expected	Observed	Expected	Total
Step 1	1	1	1.000	1	1.000	2
	2	49	49.000	67	67.000	116

Classification Table[a]

Observed			Predicted: CS No	Predicted: CS Yes	Percentage Correct
Step 1	CS	No	1	49	2.0
		Yes	1	67	98.5
	Overall Percentage				57.6

a. The cut value is .500

Variables in the Equation

		B	S.E.	Wald	df	Sig.	Exp(B)	95% C.I.for EXP(B) Lower	95% C.I.for EXP(B) Upper
Step 1[a]	Port.size	-.156	.713	.048	1	.826	.855	.211	3.461
	Constant	.469	.761	.380	1	.538	1.599		

a. Variable(s) entered on step 1: Port.size.

```
LOGISTIC REGRESSION VARIABLES Compensatory.sweating
 /METHOD=ENTER CO2.usage
 /METHOD=ENTER Level.of.Sympathectomy
 /PRINT=GOODFIT CI(95)
 /CRITERIA=PIN(0.05) POUT(0.10) ITERATE(20) CUT(0.5).
```

Logistic Regression

Notes

Output Created		18-APR-2018 18:48:...
Comments		
Input	Data	C:\Users\rnordin.ADMIN\Desktop\2018\PUBLICATION 2018 ETS\ETS.Data (Complete).sav 18APRIL2018.sav
	Active Dataset	DataSet1
	Filter	<none>
	Weight	<none>
	Split File	<none>
	N of Rows in Working Data File	118
Missing Value Handling	Definition of Missing	User-defined missing values are treated as missing
Syntax		LOGISTIC REGRESSION VARIABLES Compensatory.sweating /METHOD=ENTER CO2.usage /METHOD=ENTER Level.of.Sympathectomy /PRINT=GOODFIT CI (95) /CRITERIA=PIN(0.05) POUT(0.10) ITERATE (20) CUT(0.5).
Resources	Processor Time	00:00:00.02
	Elapsed Time	00:00:00.02

Case Processing Summary

Unweighted Cases[a]		N	Percent
Selected Cases	Included in Analysis	118	100.0
	Missing Cases	0	.0
	Total	118	100.0
Unselected Cases		0	.0
Total		118	100.0

a. If weight is in effect, see classification table for the total number of cases.

Dependent Variable Encoding

Original Value	Internal Value
No	0
Yes	1

Block 0: Beginning Block

Classification Table[a,b]

Observed			Predicted CS No	Predicted CS Yes	Percentage Correct
Step 0	CS	No	0	50	.0
		Yes	0	68	100.0
	Overall Percentage				57.6

a. Constant is included in the model.

b. The cut value is .500

Variables in the Equation

		B	S.E.	Wald	df	Sig.	Exp(B)
Step 0	Constant	.307	.186	2.724	1	.099	1.360

Block 1: Method = Enter

Model Summary

Step	-2 Log likelihood	Cox & Snell R Square	Nagelkerke R Square
1	160.826[a]	.000	.000

a. Estimation terminated at iteration number 3 because parameter estimates changed by less than .001.

Hosmer and Lemeshow Test

Step	Chi-square	df	Sig.
1	.000	0	.

Contingency Table for Hosmer and Lemeshow Test

		CS = No		CS = Yes		
		Observed	Expected	Observed	Expected	Total
Step 1	1	50	50.000	68	68.000	118

Classification Table[a]

Observed			Predicted CS No	Predicted CS Yes	Percentage Correct
Step 1	CS	No	0	50	.0
		Yes	0	68	100.0
	Overall Percentage				57.6

a. The cut value is .500

Variables in the Equation

		B	S.E.	Wald	df	Sig.	Exp(B)
Step 1	Constant	.307	.186	2.724	1	.099	1.360

Block 2: Method = Enter

Omnibus Tests of Model Coefficients

		Chi-square	df	Sig.
Step 1	Step	4.114	1	.043
	Block	4.114	1	.043
	Model	4.114	1	.043

Model Summary

Step	-2 Log likelihood	Cox & Snell R Square	Nagelkerke R Square
1	156.712[a]	.034	.046

a. Estimation terminated at iteration number 3 because parameter estimates changed by less than .001.

Hosmer and Lemeshow Test

Step	Chi-square	df	Sig.
1	.000	0	.

Contingency Table for Hosmer and Lemeshow Test

		CS = No Observed	CS = No Expected	CS = Yes Observed	CS = Yes Expected	Total
Step 1	1	27	27.000	24	24.000	51
	2	23	23.000	44	44.000	67

Classification Table[a]

Observed			Predicted CS No	Predicted CS Yes	Percentage Correct
Step 1	CS	No	27	23	54.0
		Yes	24	44	64.7
	Overall Percentage				60.2

a. The cut value is .500

Variables in the Equation

		B	S.E.	Wald	df	Sig.	Exp(B)	95% C.I.for EXP(B) Lower	Upper
Step 1[a]	Level.of.Sympathectomy	-.383	.190	4.054	1	.044	.682	.469	.990
	Constant	2.182	.954	5.227	1	.022	8.861		

a. Variable(s) entered on step 1: Level.of.Sympathectomy.

```
LOGISTIC REGRESSION VARIABLES Compensatory.sweating
 /METHOD=ENTER Sympathectomy.Level
 /CONTRAST (Sympathectomy.Level)=Indicator(1)
 /PRINT=GOODFIT CI(95)
 /CRITERIA=PIN(0.05) POUT(0.10) ITERATE(20) CUT(0.5).
```

Logistic Regression

Notes

Output Created		18-APR-2018 18:50:...
Comments		
Input	Data	C:\Users\rnordin.ADMIN\Desktop\2018\PUBLICATION 2018 ETS\ETS.Data (Complete).sav 18APRIL2018.sav
	Active Dataset	DataSet1
	Filter	<none>
	Weight	<none>
	Split File	<none>
	N of Rows in Working Data File	118
Missing Value Handling	Definition of Missing	User-defined missing values are treated as missing
Syntax		LOGISTIC REGRESSION VARIABLES Compensatory.sweating /METHOD=ENTER Sympathectomy.Level /CONTRAST (Sympathectomy.Level) =Indicator(1) /PRINT=GOODFIT CI (95) /CRITERIA=PIN(0.05) POUT(0.10) ITERATE (20) CUT(0.5).
Resources	Processor Time	00:00:00.00
	Elapsed Time	00:00:00.01

Case Processing Summary

Unweighted Cases[a]		N	Percent
Selected Cases	Included in Analysis	118	100.0
	Missing Cases	0	.0
	Total	118	100.0
Unselected Cases		0	.0
Total		118	100.0

a. If weight is in effect, see classification table for the total number of cases.

Dependent Variable Encoding

Original Value	Internal Value
No	0
Yes	1

Categorical Variables Codings

		Frequency	Parameter coding (1)
Sympathectomy.Level	T2-T3	67	.000
	T2-T4	51	1.000

Block 0: Beginning Block

Classification Table[a,b]

Observed			Predicted CS No	Predicted CS Yes	Percentage Correct
Step 0	CS	No	0	50	.0
		Yes	0	68	100.0
	Overall Percentage				57.6

a. Constant is included in the model.

b. The cut value is .500

Variables in the Equation

		B	S.E.	Wald	df	Sig.	Exp(B)
Step 0	Constant	.307	.186	2.724	1	.099	1.360

Variables not in the Equation

			Score	df	Sig.
Step 0	Variables	Sympathectomy.Level(1)	4.108	1	.043
	Overall Statistics		4.108	1	.043

Block 1: Method = Enter

Omnibus Tests of Model Coefficients

		Chi-square	df	Sig.
Step 1	Step	4.114	1	.043
	Block	4.114	1	.043
	Model	4.114	1	.043

Model Summary

Step	-2 Log likelihood	Cox & Snell R Square	Nagelkerke R Square
1	156.712[a]	.034	.046

a. Estimation terminated at iteration number 3 because parameter estimates changed by less than .001.

Hosmer and Lemeshow Test

Step	Chi-square	df	Sig.
1	.000	0	.

Contingency Table for Hosmer and Lemeshow Test

		CS = No		CS = Yes		
		Observed	Expected	Observed	Expected	Total
Step 1	1	27	27.000	24	24.000	51
	2	23	23.000	44	44.000	67

Classification Table[a]

Observed			Predicted: CS No	Predicted: CS Yes	Predicted: Percentage Correct
Step 1	CS	No	27	23	54.0
		Yes	24	44	64.7
	Overall Percentage				60.2

a. The cut value is .500

Variables in the Equation

		B	S.E.	Wald	df	Sig.	Exp(B)	95% C.I.for EXP(B) Lower	95% C.I.for EXP(B) Upper
Step 1[a]	Sympathectomy.Level(1)	-.766	.381	4.054	1	.044	.465	.220	.980
	Constant	.649	.257	6.356	1	.012	1.913		

a. Variable(s) entered on step 1: Sympathectomy.Level.

```
LOGISTIC REGRESSION VARIABLES Compensatory.sweating
 /METHOD=ENTER Method.of.excision
 /METHOD=ENTER Histopathology.sent
 /METHOD=ENTER Duration.surgery
 /PRINT=GOODFIT CI(95)
 /CRITERIA=PIN(0.05) POUT(0.10) ITERATE(20) CUT(0.5).
```

Logistic Regression

Notes

Output Created		18-APR-2018 18:51:...
Comments		
Input	Data	C:\Users\rnordin.ADMIN\Desktop\2018\PUBLICATION 2018 ETS\ETS.Data (Complete).sav 18APRIL2018.sav
	Active Dataset	DataSet1
	Filter	<none>
	Weight	<none>
	Split File	<none>
	N of Rows in Working Data File	118
Missing Value Handling	Definition of Missing	User-defined missing values are treated as missing
Syntax		LOGISTIC REGRESSION VARIABLES Compensatory.sweating /METHOD=ENTER Method.of.excision /METHOD=ENTER Histopathology.sent /METHOD=ENTER Duration.surgery /PRINT=GOODFIT CI (95) /CRITERIA=PIN(0.05) POUT(0.10) ITERATE(20) CUT(0.5).
Resources	Processor Time	00:00:00.03
	Elapsed Time	00:00:00.02

Case Processing Summary

Unweighted Cases[a]		N	Percent
Selected Cases	Included in Analysis	118	100.0
	Missing Cases	0	.0
	Total	118	100.0
Unselected Cases		0	.0
Total		118	100.0

a. If weight is in effect, see classification table for the total number of cases.

Dependent Variable Encoding

Original Value	Internal Value
No	0
Yes	1

Block 0: Beginning Block

Classification Table[a,b]

			Predicted		
			CS		Percentage Correct
Observed			No	Yes	
Step 0	CS	No	0	50	.0
		Yes	0	68	100.0
	Overall Percentage				57.6

a. Constant is included in the model.

b. The cut value is .500

Variables in the Equation

		B	S.E.	Wald	df	Sig.	Exp(B)
Step 0	Constant	.307	.186	2.724	1	.099	1.360

Variables not in the Equation

			Score	df	Sig.
Step 0	Variables	Method.of.excision	1.372	1	.242
	Overall Statistics		1.372	1	.242

Block 1: Method = Enter

Omnibus Tests of Model Coefficients

		Chi-square	df	Sig.
Step 1	Step	1.729	1	.189
	Block	1.729	1	.189
	Model	1.729	1	.189

Model Summary

Step	-2 Log likelihood	Cox & Snell R Square	Nagelkerke R Square
1	159.097[a]	.015	.020

a. Estimation terminated at iteration number 20 because maximum iterations has been reached. Final solution cannot be found.

Hosmer and Lemeshow Test

Step	Chi-square	df	Sig.
1	.000	0	.

Contingency Table for Hosmer and Lemeshow Test

		CS = No		CS = Yes		
		Observed	Expected	Observed	Expected	Total
Step 1	1	1	1.000	0	.000	1
	2	49	49.000	68	68.000	117

Classification Table[a]

Observed			Predicted CS No	Predicted CS Yes	Percentage Correct
Step 1	CS	No	1	49	2.0
		Yes	0	68	100.0
	Overall Percentage				58.5

a. The cut value is .500

Variables in the Equation

		B	S.E.	Wald	df	Sig.	Exp(B)	95% C.I.for EXP(B) Lower	95% C.I.for EXP(B) Upper
Step 1[a]	Method.of.excision	-10.765	20096.496	.000	1	1.000	.000	.000	.
	Constant	11.093	20096.496	.000	1	1.000	65708.190		

a. Variable(s) entered on step 1: Method.of.excision.

Block 2: Method = Enter

Omnibus Tests of Model Coefficients

		Chi-square	df	Sig.
Step 1	Model	1.729	1	.189

Model Summary

Step	-2 Log likelihood	Cox & Snell R Square	Nagelkerke R Square
1	159.097[a]	.015	.020

a. Estimation terminated at iteration number 20 because maximum iterations has been reached. Final solution cannot be found.

Hosmer and Lemeshow Test

Step	Chi-square	df	Sig.
1	.000	0	.

Contingency Table for Hosmer and Lemeshow Test

		CS = No		CS = Yes		
		Observed	Expected	Observed	Expected	Total
Step 1	1	1	1.000	0	.000	1
	2	49	49.000	68	68.000	117

Classification Table[a]

Observed			Predicted: CS No	Predicted: CS Yes	Percentage Correct
Step 1	CS	No	1	49	2.0
		Yes	0	68	100.0
	Overall Percentage				58.5

a. The cut value is .500

Variables in the Equation

		B	S.E.	Wald	df	Sig.	Exp(B)	95% C.I.for EXP(B) Lower	95% C.I.for EXP(B) Upper
Step 1[a]	Method.of.excision	-10.765	20096.496	.000	1	1.000	.000	.000	.
	Constant	11.093	20096.496	.000	1	1.000	65708.190		

a. Variable(s) entered on step 1: Method.of.excision.

Block 3: Method = Enter

Omnibus Tests of Model Coefficients

		Chi-square	df	Sig.
Step 1	Step	.551	1	.458
	Block	.551	1	.458
	Model	2.280	2	.320

Model Summary

Step	-2 Log likelihood	Cox & Snell R Square	Nagelkerke R Square
1	158.546[a]	.019	.026

a. Estimation terminated at iteration number 20 because maximum iterations has been reached. Final solution cannot be found.

Hosmer and Lemeshow Test

Step	Chi-square	df	Sig.
1	9.759	6	.135

Contingency Table for Hosmer and Lemeshow Test

		CS = No		CS = Yes		
		Observed	Expected	Observed	Expected	Total
Step 1	1	9	7.331	5	6.669	14
	2	2	4.056	7	4.944	9
	3	4	3.948	5	5.052	9
	4	8	7.681	10	10.319	18
	5	9	7.889	10	11.111	19
	6	2	6.449	14	9.551	16
	7	9	6.654	8	10.346	17
	8	7	5.992	9	10.008	16

Classification Table[a]

			Predicted		
			CS		Percentage Correct
Observed			No	Yes	
Step 1	CS	No	4	46	8.0
		Yes	1	67	98.5
	Overall Percentage				60.2

a. The cut value is .500

Variables in the Equation

		B	S.E.	Wald	df	Sig.	Exp(B)	95% C.I.for EXP(B)	
								Lower	Upper
Step 1[a]	Method.of.excision	-10.725	20096.438	.000	1	1.000	.000	.000	.
	Duration.surgery	-.010	.013	.549	1	.459	.990	.965	1.016
	Constant	11.507	20096.438	.000	1	1.000	99366.148		

a. Variable(s) entered on step 1: Duration.surgery.

```
LOGISTIC REGRESSION VARIABLES Compensatory.sweating
 /METHOD=ENTER DurationOfSurgery
 /CONTRAST (DurationOfSurgery)=Indicator(1)
 /PRINT=GOODFIT CI(95)
 /CRITERIA=PIN(0.05) POUT(0.10) ITERATE(20) CUT(0.5).
```

Logistic Regression

Notes

Output Created		18-APR-2018 18:51:...
Comments		
Input	Data	C:\Users\rnordin.ADMIN\Desktop\2018\PUBLICATION 2018 ETS\ETS.Data (Complete).sav 18APRIL2018.sav
	Active Dataset	DataSet1
	Filter	<none>
	Weight	<none>
	Split File	<none>
	N of Rows in Working Data File	118
Missing Value Handling	Definition of Missing	User-defined missing values are treated as missing
Syntax		LOGISTIC REGRESSION VARIABLES Compensatory.sweating /METHOD=ENTER DurationOfSurgery /CONTRAST (DurationOfSurgery)=Indicator(1) /PRINT=GOODFIT CI(95) /CRITERIA=PIN(0.05) POUT(0.10) ITERATE(20) CUT(0.5).
Resources	Processor Time	00:00:00.00
	Elapsed Time	00:00:00.02

Case Processing Summary

Unweighted Cases[a]		N	Percent
Selected Cases	Included in Analysis	118	100.0
	Missing Cases	0	.0
	Total	118	100.0
Unselected Cases		0	.0
Total		118	100.0

a. If weight is in effect, see classification table for the total number of cases.

Dependent Variable Encoding

Original Value	Internal Value
No	0
Yes	1

Categorical Variables Codings

		Frequency	Parameter coding (1)
DurationOfSurgery	Median & below	67	.000
	Above median	51	1.000

Block 0: Beginning Block

Classification Table[a,b]

	Observed		Predicted CS No	Predicted CS Yes	Percentage Correct
Step 0	CS	No	0	50	.0
		Yes	0	68	100.0
	Overall Percentage				57.6

a. Constant is included in the model.

b. The cut value is .500

Variables in the Equation

		B	S.E.	Wald	df	Sig.	Exp(B)
Step 0	Constant	.307	.186	2.724	1	.099	1.360

Variables not in the Equation

			Score	df	Sig.
Step 0	Variables	DurationOfSurgery(1)	.273	1	.601
	Overall Statistics		.273	1	.601

Block 1: Method = Enter

Omnibus Tests of Model Coefficients

		Chi-square	df	Sig.
Step 1	Step	.273	1	.601
	Block	.273	1	.601
	Model	.273	1	.601

Model Summary

Step	-2 Log likelihood	Cox & Snell R Square	Nagelkerke R Square
1	160.553[a]	.002	.003

a. Estimation terminated at iteration number 3 because parameter estimates changed by less than .001.

Hosmer and Lemeshow Test

Step	Chi-square	df	Sig.
1	.000	0	.

Contingency Table for Hosmer and Lemeshow Test

		CS = No		CS = Yes		
		Observed	Expected	Observed	Expected	Total
Step 1	1	23	23.000	28	28.000	51
	2	27	27.000	40	40.000	67

Classification Table[a]

Observed			Predicted		
			CS		Percentage Correct
			No	Yes	
Step 1	CS	No	0	50	.0
		Yes	0	68	100.0
	Overall Percentage				57.6

a. The cut value is .500

Variables in the Equation

		B	S.E.	Wald	df	Sig.	Exp(B)	95% C.I.for EXP(B)	
								Lower	Upper
Step 1[a]	DurationOfSurgery(1)	-.196	.376	.273	1	.601	.822	.393	1.716
	Constant	.393	.249	2.490	1	.115	1.481		

a. Variable(s) entered on step 1: DurationOfSurgery.

```
LOGISTIC REGRESSION VARIABLES Compensatory.sweating
 /METHOD=ENTER ICU.Stay
 /PRINT=GOODFIT CI(95)
 /CRITERIA=PIN(0.05) POUT(0.10) ITERATE(20) CUT(0.5).
```

Logistic Regression

Notes

Output Created		18-APR-2018 18:51:...
Comments		
Input	Data	C:\Users\rnordin.ADMIN\Desktop\2018\PUBLICATION 2018 ETS\ETS.Data (Complete).sav 18APRIL2018.sav
	Active Dataset	DataSet1
	Filter	<none>
	Weight	<none>
	Split File	<none>
	N of Rows in Working Data File	118
Missing Value Handling	Definition of Missing	User-defined missing values are treated as missing
Syntax		LOGISTIC REGRESSION VARIABLES Compensatory.sweating /METHOD=ENTER ICU.Stay /PRINT=GOODFIT CI(95) /CRITERIA=PIN(0.05) POUT(0.10) ITERATE(20) CUT(0.5).
Resources	Processor Time	00:00:00.03
	Elapsed Time	00:00:00.02

Case Processing Summary

Unweighted Cases[a]		N	Percent
Selected Cases	Included in Analysis	118	100.0
	Missing Cases	0	.0
	Total	118	100.0
Unselected Cases		0	.0
Total		118	100.0

a. If weight is in effect, see classification table for the total number of cases.

Dependent Variable Encoding

Original Value	Internal Value
No	0
Yes	1

Block 0: Beginning Block

Classification Table[a,b]

Observed			Predicted CS No	Predicted CS Yes	Percentage Correct
Step 0	CS	No	0	50	.0
		Yes	0	68	100.0
	Overall Percentage				57.6

a. Constant is included in the model.

b. The cut value is .500

Variables in the Equation

		B	S.E.	Wald	df	Sig.	Exp(B)
Step 0	Constant	.307	.186	2.724	1	.099	1.360

Variables not in the Equation

			Score	df	Sig.
Step 0	Variables	ICU.Stay	.512	1	.474
	Overall Statistics		.512	1	.474

Block 1: Method = Enter

Omnibus Tests of Model Coefficients

		Chi-square	df	Sig.
Step 1	Step	.543	1	.461
	Block	.543	1	.461
	Model	.543	1	.461

Model Summary

Step	-2 Log likelihood	Cox & Snell R Square	Nagelkerke R Square
1	160.283[a]	.005	.006

a. Estimation terminated at iteration number 4 because parameter estimates changed by less than .001.

Hosmer and Lemeshow Test

Step	Chi-square	df	Sig.
1	.000	0	.

Contingency Table for Hosmer and Lemeshow Test

		CS = No		CS = Yes		
		Observed	Expected	Observed	Expected	Total
Step 1	1	50	50.000	68	68.000	118

Classification Table[a]

Observed			Predicted CS: No	Predicted CS: Yes	Percentage Correct
Step 1	CS	No	0	50	.0
		Yes	0	68	100.0
	Overall Percentage				57.6

a. The cut value is .500

Variables in the Equation

		B	S.E.	Wald	df	Sig.	Exp(B)	95% C.I.for EXP(B) Lower	95% C.I.for EXP(B) Upper
Step 1[a]	ICU.Stay	-.816	1.170	.486	1	.486	.442	.045	4.381
	Constant	1.915	2.317	.683	1	.409	6.785		

a. Variable(s) entered on step 1: ICU.Stay.

```
LOGISTIC REGRESSION VARIABLES Compensatory.sweating
```

```
/METHOD=ENTER Hospital.stay
/PRINT=GOODFIT CI(95)
/CRITERIA=PIN(0.05) POUT(0.10) ITERATE(20) CUT(0.5).
```

Logistic Regression

Notes

Output Created		18-APR-2018 18:52:...
Comments		
Input	Data	C:\Users\rnordin.ADMIN\Desktop\2018\PUBLICATION 2018 ETS\ETS.Data (Complete).sav 18APRIL2018.sav
	Active Dataset	DataSet1
	Filter	<none>
	Weight	<none>
	Split File	<none>
	N of Rows in Working Data File	118
Missing Value Handling	Definition of Missing	User-defined missing values are treated as missing
Syntax		LOGISTIC REGRESSION VARIABLES Compensatory.sweating /METHOD=ENTER Hospital.stay /PRINT=GOODFIT CI (95) /CRITERIA=PIN(0.05) POUT(0.10) ITERATE(20) CUT(0.5).
Resources	Processor Time	00:00:00.02
	Elapsed Time	00:00:00.02

Case Processing Summary

Unweighted Cases[a]		N	Percent
Selected Cases	Included in Analysis	118	100.0
	Missing Cases	0	.0
	Total	118	100.0
Unselected Cases		0	.0
Total		118	100.0

a. If weight is in effect, see classification table for the total number of cases.

Dependent Variable Encoding

Original Value	Internal Value
No	0
Yes	1

Block 0: Beginning Block

Classification Table[a,b]

Observed			Predicted CS No	Predicted CS Yes	Percentage Correct
Step 0	CS	No	0	50	.0
		Yes	0	68	100.0
	Overall Percentage				57.6

a. Constant is included in the model.

b. The cut value is .500

Variables in the Equation

		B	S.E.	Wald	df	Sig.	Exp(B)
Step 0	Constant	.307	.186	2.724	1	.099	1.360

Variables not in the Equation

			Score	df	Sig.
Step 0	Variables	Hospital.stay	2.131	1	.144
	Overall Statistics		2.131	1	.144

Block 1: Method = Enter

Omnibus Tests of Model Coefficients

		Chi-square	df	Sig.
Step 1	Step	2.133	1	.144
	Block	2.133	1	.144
	Model	2.133	1	.144

Model Summary

Step	-2 Log likelihood	Cox & Snell R Square	Nagelkerke R Square
1	158.693[a]	.018	.024

a. Estimation terminated at iteration number 3 because parameter estimates changed by less than .001.

Hosmer and Lemeshow Test

Step	Chi-square	df	Sig.
1	.050	1	.824

Contingency Table for Hosmer and Lemeshow Test

		CS = No		CS = Yes		
		Observed	Expected	Observed	Expected	Total
Step 1	1	9	8.957	7	7.043	16
	2	13	13.517	17	16.483	30
	3	28	27.526	44	44.474	72

Classification Table[a]

Observed			Predicted CS No	Predicted CS Yes	Percentage Correct
Step 1	CS	No	9	41	18.0
		Yes	7	61	89.7
	Overall Percentage				59.3

a. The cut value is .500

Variables in the Equation

		B	S.E.	Wald	df	Sig.	Exp(B)	95% C.I.for EXP(B) Lower	95% C.I.for EXP(B) Upper
Step 1[a]	Hospital.stay	-.264	.185	2.031	1	.154	.768	.534	1.104
	Constant	1.254	.690	3.300	1	.069	3.505		

a. Variable(s) entered on step 1: Hospital.stay.

```
LOGISTIC REGRESSION VARIABLES Compensatory.sweating
 /METHOD=ENTER ComplicationYN
 /CONTRAST (ComplicationYN)=Indicator(1)
 /PRINT=GOODFIT CI(95)
 /CRITERIA=PIN(0.05) POUT(0.10) ITERATE(20) CUT(0.5).
```

Logistic Regression

Notes

Output Created		18-APR-2018 18:52:...
Comments		
Input	Data	C:\Users\rnordin.ADMIN\Desktop\2018\PUBLICATION 2018 ETS\ETS.Data (Complete).sav 18APRIL2018.sav
	Active Dataset	DataSet1
	Filter	<none>
	Weight	<none>
	Split File	<none>
	N of Rows in Working Data File	118
Missing Value Handling	Definition of Missing	User-defined missing values are treated as missing
Syntax		LOGISTIC REGRESSION VARIABLES Compensatory.sweating /METHOD=ENTER ComplicationYN /CONTRAST (ComplicationYN)=Indicator(1) /PRINT=GOODFIT CI(95) /CRITERIA=PIN(0.05) POUT(0.10) ITERATE(20) CUT(0.5).
Resources	Processor Time	00:00:00.02
	Elapsed Time	00:00:00.02

Case Processing Summary

Unweighted Cases[a]		N	Percent
Selected Cases	Included in Analysis	118	100.0
	Missing Cases	0	.0
	Total	118	100.0
Unselected Cases		0	.0
Total		118	100.0

a. If weight is in effect, see classification table for the total number of cases.

Dependent Variable Encoding

Original Value	Internal Value
No	0
Yes	1

Categorical Variables Codings

		Frequency	Parameter coding (1)
ComplicationYN	No	103	.000
	Yes	15	1.000

Block 0: Beginning Block

Classification Table[a,b]

Observed			Predicted CS No	Predicted CS Yes	Percentage Correct
Step 0	CS	No	0	50	.0
		Yes	0	68	100.0
	Overall Percentage				57.6

a. Constant is included in the model.

b. The cut value is .500

Variables in the Equation

		B	S.E.	Wald	df	Sig.	Exp(B)
Step 0	Constant	.307	.186	2.724	1	.099	1.360

Variables not in the Equation

			Score	df	Sig.
Step 0	Variables	ComplicationYN(1)	.845	1	.358
	Overall Statistics		.845	1	.358

Block 1: Method = Enter

Omnibus Tests of Model Coefficients

		Chi-square	df	Sig.
Step 1	Step	.835	1	.361
	Block	.835	1	.361
	Model	.835	1	.361

Model Summary

Step	-2 Log likelihood	Cox & Snell R Square	Nagelkerke R Square
1	159.991[a]	.007	.009

a. Estimation terminated at iteration number 3 because parameter estimates changed by less than .001.

Hosmer and Lemeshow Test

Step	Chi-square	df	Sig.
1	.000	0	.

Contingency Table for Hosmer and Lemeshow Test

		CS = No		CS = Yes		
		Observed	Expected	Observed	Expected	Total
Step 1	1	8	8.000	7	7.000	15
	2	42	42.000	61	61.000	103

Classification Table[a]

Observed			Predicted CS No	Predicted CS Yes	Percentage Correct
Step 1	CS	No	8	42	16.0
		Yes	7	61	89.7
	Overall Percentage				58.5

a. The cut value is .500

Variables in the Equation

		B	S.E.	Wald	df	Sig.	Exp(B)	95% C.I.for EXP(B)	
								Lower	Upper
Step 1[a]	ComplicationYN(1)	-.507	.555	.834	1	.361	.602	.203	1.788
	Constant	.373	.201	3.464	1	.063	1.452		

a. Variable(s) entered on step 1: ComplicationYN.

```
LOGISTIC REGRESSION VARIABLES Compensatory.sweating
 /METHOD=ENTER Follow.up
 /PRINT=GOODFIT CI(95)
 /CRITERIA=PIN(0.05) POUT(0.10) ITERATE(20) CUT(0.5).
```

Logistic Regression

Notes

Output Created		18-APR-2018 18:52:...
Comments		
Input	Data	C:\Users\rnordin.ADMIN\Desktop\2018\PUBLICATION 2018 ETS\ETS.Data (Complete).sav 18APRIL2018.sav
	Active Dataset	DataSet1
	Filter	<none>
	Weight	<none>
	Split File	<none>
	N of Rows in Working Data File	118
Missing Value Handling	Definition of Missing	User-defined missing values are treated as missing
Syntax		LOGISTIC REGRESSION VARIABLES Compensatory.sweating /METHOD=ENTER Follow.up /PRINT=GOODFIT CI (95) /CRITERIA=PIN(0.05) POUT(0.10) ITERATE(20) CUT(0.5).
Resources	Processor Time	00:00:00.02
	Elapsed Time	00:00:00.01

Case Processing Summary

Unweighted Cases[a]		N	Percent
Selected Cases	Included in Analysis	118	100.0
	Missing Cases	0	.0
	Total	118	100.0
Unselected Cases		0	.0
Total		118	100.0

a. If weight is in effect, see classification table for the total number of cases.

Dependent Variable Encoding

Original Value	Internal Value
No	0
Yes	1

Block 0: Beginning Block

Classification Table[a,b]

Observed			Predicted CS No	Predicted CS Yes	Percentage Correct
Step 0	CS	No	0	50	.0
		Yes	0	68	100.0
	Overall Percentage				57.6

a. Constant is included in the model.

b. The cut value is .500

Variables in the Equation

		B	S.E.	Wald	df	Sig.	Exp(B)
Step 0	Constant	.307	.186	2.724	1	.099	1.360

Variables not in the Equation

			Score	df	Sig.
Step 0	Variables	Follow.up	2.767	1	.096
	Overall Statistics		2.767	1	.096

Block 1: Method = Enter

Omnibus Tests of Model Coefficients

		Chi-square	df	Sig.
Step 1	Step	3.482	1	.062
	Block	3.482	1	.062
	Model	3.482	1	.062

Model Summary

Step	-2 Log likelihood	Cox & Snell R Square	Nagelkerke R Square
1	157.345[a]	.029	.039

a. Estimation terminated at iteration number 20 because maximum iterations has been reached. Final solution cannot be found.

Hosmer and Lemeshow Test

Step	Chi-square	df	Sig.
1	.000	0	.

Contingency Table for Hosmer and Lemeshow Test

		CS = No		CS = Yes		
		Observed	Expected	Observed	Expected	Total
Step 1	1	2	2.000	0	.000	2
	2	48	48.000	68	68.000	116

Classification Table[a]

Observed			Predicted CS No	Predicted CS Yes	Percentage Correct
Step 1	CS	No	2	48	4.0
		Yes	0	68	100.0
	Overall Percentage				59.3

a. The cut value is .500

Variables in the Equation

		B	S.E.	Wald	df	Sig.	Exp(B)	95% C.I.for EXP(B) Lower	Upper
Step 1[a]	Follow.up	-21.551	28420.737	.000	1	.999	.000	.000	.
	Constant	21.900	28420.737	.000	1	.999	3.242E+9		

a. Variable(s) entered on step 1: Follow.up.

```
LOGISTIC REGRESSION VARIABLES Compensatory.sweating
 /METHOD=ENTER FollowupYN
 /CONTRAST (FollowupYN)=Indicator(1)
 /PRINT=GOODFIT CI(95)
 /CRITERIA=PIN(0.05) POUT(0.10) ITERATE(20) CUT(0.5).
```

Logistic Regression

Notes

Output Created		18-APR-2018 18:53:...
Comments		
Input	Data	C:\Users\rnordin.ADMIN\Desktop\2018\PUBLICATION 2018 ETS\ETS.Data (Complete).sav 18APRIL2018.sav
	Active Dataset	DataSet1
	Filter	<none>
	Weight	<none>
	Split File	<none>
	N of Rows in Working Data File	118
Missing Value Handling	Definition of Missing	User-defined missing values are treated as missing
Syntax		LOGISTIC REGRESSION VARIABLES Compensatory.sweating /METHOD=ENTER FollowupYN /CONTRAST (FollowupYN)=Indicator (1) /PRINT=GOODFIT CI (95) /CRITERIA=PIN(0.05) POUT(0.10) ITERATE (20) CUT(0.5).
Resources	Processor Time	00:00:00.03
	Elapsed Time	00:00:00.02

Case Processing Summary

Unweighted Cases[a]		N	Percent
Selected Cases	Included in Analysis	115	97.5
	Missing Cases	3	2.5
	Total	118	100.0
Unselected Cases		0	.0
Total		118	100.0

a. If weight is in effect, see classification table for the total number of cases.

Dependent Variable Encoding

Original Value	Internal Value
No	0
Yes	1

Categorical Variables Codings

		Frequency	Parameter coding (1)
FollowupYN	One	77	.000
	More than one	38	1.000

Block 0: Beginning Block

Classification Table[a,b]

Observed			Predicted CS No	Predicted CS Yes	Percentage Correct
Step 0	CS	No	0	48	.0
		Yes	0	67	100.0
	Overall Percentage				58.3

a. Constant is included in the model.

b. The cut value is .500

Variables in the Equation

		B	S.E.	Wald	df	Sig.	Exp(B)
Step 0	Constant	.333	.189	3.110	1	.078	1.396

Variables not in the Equation

			Score	df	Sig.
Step 0	Variables	FollowupYN(1)	22.737	1	.000
	Overall Statistics		22.737	1	.000

Block 1: Method = Enter

Omnibus Tests of Model Coefficients

		Chi-square	df	Sig.
Step 1	Step	25.529	1	.000
	Block	25.529	1	.000
	Model	25.529	1	.000

Model Summary

Step	-2 Log likelihood	Cox & Snell R Square	Nagelkerke R Square
1	130.742[a]	.199	.268

a. Estimation terminated at iteration number 5 because parameter estimates changed by less than .001.

Hosmer and Lemeshow Test

Step	Chi-square	df	Sig.
1	.000	0	.

Contingency Table for Hosmer and Lemeshow Test

		CS = No		CS = Yes		
		Observed	Expected	Observed	Expected	Total
Step 1	1	44	44.000	33	33.000	77
	2	4	4.000	34	34.000	38

Classification Table[a]

Observed			Predicted CS No	Predicted CS Yes	Percentage Correct
Step 1	CS	No	44	4	91.7
		Yes	33	34	50.7
	Overall Percentage				67.8

a. The cut value is .500

Variables in the Equation

		B	S.E.	Wald	df	Sig.	Exp(B)	95% C.I.for EXP(B) Lower	Upper
Step 1[a]	FollowupYN(1)	2.428	.577	17.729	1	.000	11.333	3.661	35.087
	Constant	-.288	.230	1.561	1	.212	.750		

a. Variable(s) entered on step 1: FollowupYN.

```
LOGISTIC REGRESSION VARIABLES Compensatory.sweating
  /METHOD=ENTER Issues
  /PRINT=GOODFIT CI(95)
  /CRITERIA=PIN(0.05) POUT(0.10) ITERATE(20) CUT(0.5).
```

Logistic Regression

Notes

Output Created		18-APR-2018 18:53:...
Comments		
Input	Data	C:\Users\rnordin.ADMIN\Desktop\2018\PUBLICATION 2018 ETS\ETS.Data (Complete).sav 18APRIL2018.sav
	Active Dataset	DataSet1
	Filter	<none>
	Weight	<none>
	Split File	<none>
	N of Rows in Working Data File	118
Missing Value Handling	Definition of Missing	User-defined missing values are treated as missing
Syntax		LOGISTIC REGRESSION VARIABLES Compensatory.sweating /METHOD=ENTER Issues /PRINT=GOODFIT CI (95) /CRITERIA=PIN(0.05) POUT(0.10) ITERATE(20) CUT(0.5).
Resources	Processor Time	00:00:00.02
	Elapsed Time	00:00:00.04

Warnings

Text: Issues Command: LOGISTIC REGRESSION
This procedure cannot use string variables longer than 8 bytes. The values will be truncated.

Case Processing Summary

Unweighted Cases[a]		N	Percent
Selected Cases	Included in Analysis	118	100.0
	Missing Cases	0	.0
	Total	118	100.0
Unselected Cases		0	.0
Total		118	100.0

a. If weight is in effect, see classification table for the total number of cases.

Dependent Variable Encoding

Original Value	Internal Value
No	0
Yes	1

Categorical Variables Codings[a]

			Parameter coding									
		Frequency	(1)	(2)	(3)	(4)	(5)	(6)	(7)	(8)	(9)	(10)
Issues	back pai	1	1.000	.000	.000	.000	.000	.000	.000	.000	.000	.000
	Bradycar	1	.000	1.000	.000	.000	.000	.000	.000	.000	.000	.000
	Chest pa	2	.000	.000	1.000	.000	.000	.000	.000	.000	.000	.000
	N/A	1	.000	.000	.000	1.000	.000	.000	.000	.000	.000	.000
	none	107	.000	.000	.000	.000	1.000	.000	.000	.000	.000	.000
	Pain	1	.000	.000	.000	.000	.000	1.000	.000	.000	.000	.000
	pain on	1	.000	.000	.000	.000	.000	.000	1.000	.000	.000	.000
	Pain, Tr	1	.000	.000	.000	.000	.000	.000	.000	1.000	.000	.000
	Post sym	1	.000	.000	.000	.000	.000	.000	.000	.000	1.000	.000
	Right si	1	.000	.000	.000	.000	.000	.000	.000	.000	.000	1.000
	surgery	1	.000	.000	.000	.000	.000	.000	.000	.000	.000	.000

a. This coding results in indicator coefficients.

Block 0: Beginning Block

Classification Table[a,b]

Observed			Predicted		
			CS		Percentage Correct
			No	Yes	
Step 0	CS	No	0	50	.0
		Yes	0	68	100.0
	Overall Percentage				57.6

a. Constant is included in the model.

b. The cut value is .500

Variables in the Equation

		B	S.E.	Wald	df	Sig.	Exp(B)
Step 0	Constant	.307	.186	2.724	1	.099	1.360

Variables not in the Equation

			Score	df	Sig.
Step 0	Variables	Issues	11.216	10	.341
		Issues(1)	1.372	1	.242
		Issues(2)	.742	1	.389
		Issues(3)	1.496	1	.221
		Issues(4)	1.372	1	.242
		Issues(5)	.047	1	.828
		Issues(6)	.742	1	.389
		Issues(7)	.742	1	.389
		Issues(8)	1.372	1	.242
		Issues(9)	.742	1	.389
		Issues(10)	1.372	1	.242
	Overall Statistics		11.216	10	.341

Block 1: Method = Enter

Omnibus Tests of Model Coefficients

		Chi-square	df	Sig.
Step 1	Step	15.205	10	.125
	Block	15.205	10	.125
	Model	15.205	10	.125

Model Summary

Step	-2 Log likelihood	Cox & Snell R Square	Nagelkerke R Square
1	145.621[a]	.121	.162

a. Estimation terminated at iteration number 20 because maximum iterations has been reached. Final solution cannot be found.

Hosmer and Lemeshow Test

Step	Chi-square	df	Sig.
1	.000	1	1.000

Contingency Table for Hosmer and Lemeshow Test

		CS = No		CS = Yes		
		Observed	Expected	Observed	Expected	Total
Step 1	1	5	5.000	0	.000	5
	2	45	45.000	62	62.000	107
	3	0	.000	6	6.000	6

Classification Table[a]

Observed			Predicted		
			CS		Percentage Correct
			No	Yes	
Step 1	CS	No	5	45	10.0
		Yes	0	68	100.0
	Overall Percentage				61.9

a. The cut value is .500

Variables in the Equation

		B	S.E.	Wald	df	Sig.	Exp(B)	95% C.I.for EXP(B)	
								Lower	Upper
Step 1[a]	Issues			.000	10	1.000			
	Issues(1)	.000	56841.452	.000	1	1.000	1.000	.000	.
	Issues(2)	42.406	56841.452	.000	1	.999	2.610E+18	.000	.
	Issues(3)	42.406	49226.144	.000	1	.999	2.610E+18	.000	.
	Issues(4)	.000	56841.452	.000	1	1.000	1.000	.000	.
	Issues(5)	21.523	40192.983	.000	1	1.000	2.226E+9	.000	.
	Issues(6)	42.406	56841.452	.000	1	.999	2.610E+18	.000	.
	Issues(7)	42.406	56841.452	.000	1	.999	2.610E+18	.000	.
	Issues(8)	.000	56841.452	.000	1	1.000	1.000	.000	.
	Issues(9)	42.406	56841.452	.000	1	.999	2.610E+18	.000	.
	Issues(10)	.000	56841.452	.000	1	1.000	1.000	.000	.
	Constant	-21.203	40192.983	.000	1	1.000	.000		

a. Variable(s) entered on step 1: Issues.

```
LOGISTIC REGRESSION VARIABLES Compensatory.sweating
  /METHOD=ENTER Location.of.CS
  /PRINT=GOODFIT CI(95)
  /CRITERIA=PIN(0.05) POUT(0.10) ITERATE(20) CUT(0.5).
```

Logistic Regression

Notes

Output Created		18-APR-2018 18:54:...
Comments		
Input	Data	C:\Users\rnordin.ADMIN\Desktop\2018\PUBLICATION 2018 ETS\ETS.Data (Complete).sav 18APRIL2018.sav
	Active Dataset	DataSet1
	Filter	<none>
	Weight	<none>
	Split File	<none>
	N of Rows in Working Data File	118
Missing Value Handling	Definition of Missing	User-defined missing values are treated as missing
Syntax		LOGISTIC REGRESSION VARIABLES Compensatory.sweating /METHOD=ENTER Location.of.CS /PRINT=GOODFIT CI (95) /CRITERIA=PIN(0.05) POUT(0.10) ITERATE (20) CUT(0.5).
Resources	Processor Time	00:00:00.02
	Elapsed Time	00:00:00.01

Case Processing Summary

Unweighted Cases[a]		N	Percent
Selected Cases	Included in Analysis	55	46.6
	Missing Cases	63	53.4
	Total	118	100.0
Unselected Cases		0	.0
Total		118	100.0

a. If weight is in effect, see classification table for the total number of cases.

Dependent Variable Encoding

Original Value	Internal Value
No	0
Yes	1

Block 0: Beginning Block

Classification Table[a,b]

Observed			Predicted CS No	Predicted CS Yes	Percentage Correct
Step 0	CS	No	0	4	.0
		Yes	0	51	100.0
	Overall Percentage				92.7

a. Constant is included in the model.

b. The cut value is .500

Variables in the Equation

		B	S.E.	Wald	df	Sig.	Exp(B)
Step 0	Constant	2.546	.519	24.034	1	.000	12.750

Variables not in the Equation

			Score	df	Sig.
Step 0	Variables	Location.of.CS	.803	1	.370
	Overall Statistics		.803	1	.370

Block 1: Method = Enter

Omnibus Tests of Model Coefficients

		Chi-square	df	Sig.
Step 1	Step	.910	1	.340
	Block	.910	1	.340
	Model	.910	1	.340

Model Summary

Step	-2 Log likelihood	Cox & Snell R Square	Nagelkerke R Square
1	27.760[a]	.016	.040

a. Estimation terminated at iteration number 6 because parameter estimates changed by less than .001.

Hosmer and Lemeshow Test

Step	Chi-square	df	Sig.
1	26.247	6	.000

Contingency Table for Hosmer and Lemeshow Test

		CS = No		CS = Yes		Total
		Observed	Expected	Observed	Expected	
Step 1	1	0	1.083	9	7.917	9
	2	0	.106	1	.894	1
	3	0	1.022	11	9.978	11
	4	4	.570	3	6.430	7
	5	0	.498	7	6.502	7
	6	0	.279	5	4.721	5
	7	0	.215	6	5.785	6
	8	0	.227	9	8.773	9

Classification Table[a]

			Predicted		
			CS		Percentage Correct
Observed			No	Yes	
Step 1	CS	No	0	4	.0
		Yes	0	51	100.0
	Overall Percentage				92.7

a. The cut value is .500

Variables in the Equation

		B	S.E.	Wald	df	Sig.	Exp(B)	95% C.I.for EXP(B)	
								Lower	Upper
Step 1[a]	Location.of.CS	-.145	.167	.751	1	.386	.865	.624	1.200
	Constant	3.871	1.736	4.973	1	.026	47.995		

a. Variable(s) entered on step 1: Location.of.CS.

```
LOGISTIC REGRESSION VARIABLES Compensatory.sweating
 /METHOD=ENTER follow.up.progression
 /PRINT=GOODFIT CI(95)
 /CRITERIA=PIN(0.05) POUT(0.10) ITERATE(20) CUT(0.5).
```

Logistic Regression

Notes

Output Created		18-APR-2018 18:54:...
Comments		
Input	Data	C:\Users\rnordin.ADMIN\Desktop\2018\PUBLICATION 2018 ETS\ETS.Data (Complete).sav 18APRIL2018.sav
	Active Dataset	DataSet1
	Filter	<none>
	Weight	<none>
	Split File	<none>
	N of Rows in Working Data File	118
Missing Value Handling	Definition of Missing	User-defined missing values are treated as missing
Syntax		LOGISTIC REGRESSION VARIABLES Compensatory.sweating /METHOD=ENTER follow.up.progression /PRINT=GOODFIT CI (95) /CRITERIA=PIN(0.05) POUT(0.10) ITERATE (20) CUT(0.5).
Resources	Processor Time	00:00:00.03
	Elapsed Time	00:00:00.02

Case Processing Summary

Unweighted Cases[a]		N	Percent
Selected Cases	Included in Analysis	80	67.8
	Missing Cases	38	32.2
	Total	118	100.0
Unselected Cases		0	.0
Total		118	100.0

a. If weight is in effect, see classification table for the total number of cases.

Dependent Variable Encoding

Original Value	Internal Value
No	0
Yes	1

Block 0: Beginning Block

Classification Table[a,b]

Observed			Predicted CS No	Predicted CS Yes	Percentage Correct
Step 0	CS	No	0	14	.0
		Yes	0	66	100.0
	Overall Percentage				82.5

a. Constant is included in the model.

b. The cut value is .500

Variables in the Equation

		B	S.E.	Wald	df	Sig.	Exp(B)
Step 0	Constant	1.551	.294	27.770	1	.000	4.714

Variables not in the Equation

			Score	df	Sig.
Step 0	Variables	follow.up.progression	44.964	1	.000
	Overall Statistics		44.964	1	.000

Block 1: Method = Enter

Omnibus Tests of Model Coefficients

		Chi-square	df	Sig.
Step 1	Step	58.352	1	.000
	Block	58.352	1	.000
	Model	58.352	1	.000

Model Summary

Step	-2 Log likelihood	Cox & Snell R Square	Nagelkerke R Square
1	15.844[a]	.518	.857

a. Estimation terminated at iteration number 20 because maximum iterations has been reached. Final solution cannot be found.

Hosmer and Lemeshow Test

Step	Chi-square	df	Sig.
1	.000	2	1.000

Contingency Table for Hosmer and Lemeshow Test

		CS = No		CS = Yes		
		Observed	Expected	Observed	Expected	Total
Step 1	1	14	14.000	3	3.000	17
	2	0	.000	5	5.000	5
	3	0	.000	51	51.000	51
	4	0	.000	7	7.000	7

Classification Table[a]

Observed			Predicted CS No	Predicted CS Yes	Percentage Correct
Step 1	CS	No	14	0	100.0
		Yes	3	63	95.5
	Overall Percentage				96.3

a. The cut value is .500

Variables in the Equation

		B	S.E.	Wald	df	Sig.	Exp(B)	95% C.I.for EXP(B)	
								Lower	Upper
Step 1[a]	follow.up.progression	-18.956	2705.839	.000	1	.994	.000	.000	.
	Constant	93.241	13529.196	.000	1	.995	3.118E+40		

a. Variable(s) entered on step 1: follow.up.progression.

```
LOGISTIC REGRESSION VARIABLES Compensatory.sweating
 /METHOD=ENTER Reduction.of.PH
 /PRINT=GOODFIT CI(95)
 /CRITERIA=PIN(0.05) POUT(0.10) ITERATE(20) CUT(0.5).
```

Logistic Regression

Notes

Output Created		18-APR-2018 18:54:...
Comments		
Input	Data	C:\Users\rnordin.ADMIN\Desktop\2018\PUBLICATION 2018 ETS\ETS.Data (Complete).sav 18APRIL2018.sav
	Active Dataset	DataSet1
	Filter	<none>
	Weight	<none>
	Split File	<none>
	N of Rows in Working Data File	118
Missing Value Handling	Definition of Missing	User-defined missing values are treated as missing
Syntax		LOGISTIC REGRESSION VARIABLES Compensatory.sweating /METHOD=ENTER Reduction.of.PH /PRINT=GOODFIT CI (95) /CRITERIA=PIN(0.05) POUT(0.10) ITERATE (20) CUT(0.5).
Resources	Processor Time	00:00:00.02
	Elapsed Time	00:00:00.02

Case Processing Summary

Unweighted Cases[a]		N	Percent
Selected Cases	Included in Analysis	118	100.0
	Missing Cases	0	.0
	Total	118	100.0
Unselected Cases		0	.0
Total		118	100.0

a. If weight is in effect, see classification table for the total number of cases.

Dependent Variable Encoding

Original Value	Internal Value
No	0
Yes	1

Block 0: Beginning Block

Classification Table[a,b]

Observed			Predicted CS No	Predicted CS Yes	Percentage Correct
Step 0	CS	No	0	50	.0
		Yes	0	68	100.0
	Overall Percentage				57.6

a. Constant is included in the model.

b. The cut value is .500

Variables in the Equation

		B	S.E.	Wald	df	Sig.	Exp(B)
Step 0	Constant	.307	.186	2.724	1	.099	1.360

Variables not in the Equation

			Score	df	Sig.
Step 0	Variables	Reduction.of.PH	2.551	1	.110
	Overall Statistics		2.551	1	.110

Block 1: Method = Enter

Omnibus Tests of Model Coefficients

		Chi-square	df	Sig.
Step 1	Step	2.714	1	.099
	Block	2.714	1	.099
	Model	2.714	1	.099

Model Summary

Step	-2 Log likelihood	Cox & Snell R Square	Nagelkerke R Square
1	158.112[a]	.023	.031

a. Estimation terminated at iteration number 4 because parameter estimates changed by less than .001.

Hosmer and Lemeshow Test

Step	Chi-square	df	Sig.
1	.000	0	.

Contingency Table for Hosmer and Lemeshow Test

		CS = No		CS = Yes		
		Observed	Expected	Observed	Expected	Total
Step 1	1	3	3.209	1	.791	4
	2	47	46.791	67	67.209	114

Classification Table[a]

Observed			Predicted CS No	Predicted CS Yes	Percentage Correct
Step 1	CS	No	3	47	6.0
		Yes	1	67	98.5
	Overall Percentage				59.3

a. The cut value is .500

Variables in the Equation

		B	S.E.	Wald	df	Sig.	Exp(B)	95% C.I.for EXP(B) Lower	95% C.I.for EXP(B) Upper
Step 1[a]	Reduction.of.PH	-.627	.468	1.794	1	.180	.534	.213	1.337
	Constant	.989	.529	3.496	1	.062	2.689		

a. Variable(s) entered on step 1: Reduction.of.PH.

```
 LOGISTIC REGRESSION VARIABLES Compensatory.sweating
  /METHOD=ENTER Medical.issues Sympathectomy.Level Hospital.stay Follow.up
Reduction.of.PH
  /CONTRAST (Medical.issues)=Indicator(1)
  /CONTRAST (Sympathectomy.Level)=Indicator(1)
  /PRINT=GOODFIT CI(95)
  /CRITERIA=PIN(0.05) POUT(0.10) ITERATE(20) CUT(0.5).
```

Logistic Regression

Notes

Output Created		18-APR-2018 18:55:...
Comments		
Input	Data	C:\Users\rnordin.ADMIN\Desktop\2018\PUBLICATION 2018 ETS\ETS.Data (Complete).sav 18APRIL2018.sav
	Active Dataset	DataSet1
	Filter	<none>
	Weight	<none>
	Split File	<none>
	N of Rows in Working Data File	118
Missing Value Handling	Definition of Missing	User-defined missing values are treated as missing
Syntax		LOGISTIC REGRESSION VARIABLES Compensatory.sweating /METHOD=ENTER Medical.issues Sympathectomy.Level Hospital.stay Follow.up Reduction.of.PH /CONTRAST (Medical.issues)=Indicator(1) /CONTRAST (Sympathectomy.Level)=Indicator(1) /PRINT=GOODFIT CI(95) /CRITERIA=PIN(0.05) POUT(0.10) ITERATE(20) CUT(0.5).
Resources	Processor Time	00:00:00.02
	Elapsed Time	00:00:00.01

Case Processing Summary

Unweighted Cases[a]		N	Percent
Selected Cases	Included in Analysis	118	100.0
	Missing Cases	0	.0
	Total	118	100.0
Unselected Cases		0	.0
Total		118	100.0

a. If weight is in effect, see classification table for the total number of cases.

Dependent Variable Encoding

Original Value	Internal Value
No	0
Yes	1

Categorical Variables Codings

		Frequency	Parameter coding (1)
Sympathectomy.Level	T2-T3	67	.000
	T2-T4	51	1.000
MedicalIssue	No	109	.000
	Yes	9	1.000

Block 0: Beginning Block

Classification Table[a,b]

Observed			Predicted CS No	Predicted CS Yes	Percentage Correct
Step 0	CS	No	0	50	.0
		Yes	0	68	100.0
	Overall Percentage				57.6

a. Constant is included in the model.

b. The cut value is .500

Variables in the Equation

		B	S.E.	Wald	df	Sig.	Exp(B)
Step 0	Constant	.307	.186	2.724	1	.099	1.360

Variables not in the Equation

			Score	df	Sig.
Step 0	Variables	MedicalIssue(1)	1.620	1	.203
		Sympathectomy.Level(1)	4.108	1	.043
		Hospital.stay	2.131	1	.144
		Follow.up	2.767	1	.096
		Reduction.of.PH	2.551	1	.110
	Overall Statistics		9.011	5	.109

Block 1: Method = Enter

Omnibus Tests of Model Coefficients

		Chi-square	df	Sig.
Step 1	Step	9.962	5	.076
	Block	9.962	5	.076
	Model	9.962	5	.076

Model Summary

Step	-2 Log likelihood	Cox & Snell R Square	Nagelkerke R Square
1	150.864[a]	.081	.109

a. Estimation terminated at iteration number 20 because maximum iterations has been reached. Final solution cannot be found.

Hosmer and Lemeshow Test

Step	Chi-square	df	Sig.
1	3.076	5	.688

Contingency Table for Hosmer and Lemeshow Test

		CS = No		CS = Yes		
		Observed	Expected	Observed	Expected	Total
Step 1	1	8	7.852	3	3.148	11
	2	5	6.124	6	4.876	11
	3	13	12.115	12	12.885	25
	4	8	7.546	11	11.454	19
	5	2	1.079	1	1.921	3
	6	13	12.760	25	25.240	38
	7	1	2.524	10	8.476	11

Classification Table[a]

Observed			Predicted		
			CS		Percentage Correct
			No	Yes	
Step 1	CS	No	13	37	26.0
		Yes	9	59	86.8
	Overall Percentage				61.0

a. The cut value is .500

Variables in the Equation

		B	S.E.	Wald	df	Sig.	Exp(B)	95% C.I.for EXP(B)	
								Lower	Upper
Step 1[a]	MedicalIssue(1)	1.072	.842	1.622	1	.203	2.922	.561	15.212
	Sympathectomy.Level(1)	-.620	.396	2.454	1	.117	.538	.247	1.169
	Hospital.stay	-.228	.189	1.463	1	.226	.796	.550	1.152
	Follow.up	-20.866	28379.274	.000	1	.999	.000	.000	.
	Reduction.of.PH	-.072	.741	.009	1	.923	.931	.218	3.976
	Constant	22.305	28379.274	.000	1	.999	4.861E+9		

a. Variable(s) entered on step 1: MedicalIssue, Sympathectomy.Level, Hospital.stay, Follow.up, Reduction.of.PH.

```
LOGISTIC REGRESSION VARIABLES Compensatory.sweating
  /METHOD=FSTEP(COND) Medical.issues Sympathectomy.Level Hospital.stay Follow.up Reduction.of.PH
  /CONTRAST (Medical.issues)=Indicator(1)
  /CONTRAST (Sympathectomy.Level)=Indicator(1)
  /PRINT=GOODFIT CI(95)
  /CRITERIA=PIN(0.05) POUT(0.10) ITERATE(20) CUT(0.5).
```

Logistic Regression

Notes

Output Created		18-APR-2018 18:56:...
Comments		
Input	Data	C:\Users\rnordin.ADMIN\Desktop\2018\PUBLICATION 2018 ETS\ETS.Data (Complete).sav 18APRIL2018.sav
	Active Dataset	DataSet1
	Filter	<none>
	Weight	<none>
	Split File	<none>
	N of Rows in Working Data File	118
Missing Value Handling	Definition of Missing	User-defined missing values are treated as missing

Notes

Syntax		LOGISTIC REGRESSION VARIABLES Compensatory.sweating /METHOD=FSTEP (COND) Medical.issues Sympathectomy.Level Hospital.stay Follow.up Reduction.of.PH /CONTRAST (Medical.issues)=Indicator(1) /CONTRAST (Sympathectomy.Level)=Indicator(1) /PRINT=GOODFIT CI (95) /CRITERIA=PIN(0.05) POUT(0.10) ITERATE (20) CUT(0.5).
Resources	Processor Time	00:00:00.03
	Elapsed Time	00:00:00.02

Case Processing Summary

Unweighted Cases[a]		N	Percent
Selected Cases	Included in Analysis	118	100.0
	Missing Cases	0	.0
	Total	118	100.0
Unselected Cases		0	.0
Total		118	100.0

a. If weight is in effect, see classification table for the total number of cases.

Dependent Variable Encoding

Original Value	Internal Value
No	0
Yes	1

Categorical Variables Codings

		Frequency	Parameter coding (1)
Sympathectomy.Level	T2-T3	67	.000
	T2-T4	51	1.000
MedicalIssue	No	109	.000
	Yes	9	1.000

Block 0: Beginning Block

Classification Table[a,b]

Observed			Predicted CS No	Predicted CS Yes	Predicted Percentage Correct
Step 0	CS	No	0	50	.0
		Yes	0	68	100.0
	Overall Percentage				57.6

a. Constant is included in the model.

b. The cut value is .500

Variables in the Equation

		B	S.E.	Wald	df	Sig.	Exp(B)
Step 0	Constant	.307	.186	2.724	1	.099	1.360

Variables not in the Equation

			Score	df	Sig.
Step 0	Variables	MedicalIssue(1)	1.620	1	.203
		Sympathectomy.Level(1)	4.108	1	.043
		Hospital.stay	2.131	1	.144
		Follow.up	2.767	1	.096
		Reduction.of.PH	2.551	1	.110
	Overall Statistics		9.011	5	.109

Block 1: Method = Forward Stepwise (Conditional)

Omnibus Tests of Model Coefficients

		Chi-square	df	Sig.
Step 1	Step	4.114	1	.043
	Block	4.114	1	.043
	Model	4.114	1	.043

Model Summary

Step	-2 Log likelihood	Cox & Snell R Square	Nagelkerke R Square
1	156.712[a]	.034	.046

a. Estimation terminated at iteration number 3 because parameter estimates changed by less than .001.

Hosmer and Lemeshow Test

Step	Chi-square	df	Sig.
1	.000	0	.

Contingency Table for Hosmer and Lemeshow Test

		CS = No		CS = Yes		
		Observed	Expected	Observed	Expected	Total
Step 1	1	27	27.000	24	24.000	51
	2	23	23.000	44	44.000	67

Classification Table[a]

Observed			Predicted CS No	Predicted CS Yes	Percentage Correct
Step 1	CS	No	27	23	54.0
		Yes	24	44	64.7
	Overall Percentage				60.2

a. The cut value is .500

Variables in the Equation

		B	S.E.	Wald	df	Sig.	Exp(B)	95% C.I.for EXP(B) Lower	95% C.I.for EXP(B) Upper
Step 1[a]	Sympathectomy.Level(1)	-.766	.381	4.054	1	.044	.465	.220	.980
	Constant	.649	.257	6.356	1	.012	1.913		

a. Variable(s) entered on step 1: Sympathectomy.Level.

Model if Term Removed[a]

Variable		Model Log Likelihood	Change in -2 Log Likelihood	df	Sig. of the Change
Step 1	Sympathectomy.Level	-80.414	4.117	1	.042

a. Based on conditional parameter estimates

Variables not in the Equation

			Score	df	Sig.
Step 1	Variables	MedicalIssue(1)	1.715	1	.190
		Hospital.stay	1.228	1	.268
		Follow.up	1.850	1	.174
		Reduction.of.PH	1.724	1	.189
	Overall Statistics		4.966	4	.291

```
LOGISTIC REGRESSION VARIABLES Compensatory.sweating
  /METHOD=FSTEP(LR) Medical.issues Sympathectomy.Level Hospital.stay Follow
.up Reduction.of.PH
  /CONTRAST (Medical.issues)=Indicator(1)
  /CONTRAST (Sympathectomy.Level)=Indicator(1)
  /PRINT=GOODFIT CI(95)
  /CRITERIA=PIN(0.05) POUT(0.10) ITERATE(20) CUT(0.5).
```

Logistic Regression

Notes

Output Created		18-APR-2018 18:56:...
Comments		
Input	Data	C:\Users\rnordin.ADMIN\Desktop\2018\PUBLICATION 2018 ETS\ETS.Data (Complete).sav 18APRIL2018.sav
	Active Dataset	DataSet1
	Filter	<none>
	Weight	<none>
	Split File	<none>
	N of Rows in Working Data File	118
Missing Value Handling	Definition of Missing	User-defined missing values are treated as missing
Syntax		LOGISTIC REGRESSION VARIABLES Compensatory.sweating /METHOD=FSTEP(LR) Medical.issues Sympathectomy.Level Hospital.stay Follow.up Reduction.of.PH /CONTRAST (Medical.issues)=Indicator(1) /CONTRAST (Sympathectomy.Level)=Indicator(1) /PRINT=GOODFIT CI(95) /CRITERIA=PIN(0.05) POUT(0.10) ITERATE(20) CUT(0.5).
Resources	Processor Time	00:00:00.00
	Elapsed Time	00:00:00.02

Case Processing Summary

Unweighted Cases[a]		N	Percent
Selected Cases	Included in Analysis	118	100.0
	Missing Cases	0	.0
	Total	118	100.0
Unselected Cases		0	.0
Total		118	100.0

a. If weight is in effect, see classification table for the total number of cases.

Dependent Variable Encoding

Original Value	Internal Value
No	0
Yes	1

Categorical Variables Codings

		Frequency	Parameter coding (1)
Sympathectomy.Level	T2-T3	67	.000
	T2-T4	51	1.000
MedicalIssue	No	109	.000
	Yes	9	1.000

Block 0: Beginning Block

Classification Table[a,b]

Observed			Predicted CS No	Predicted CS Yes	Percentage Correct
Step 0	CS	No	0	50	.0
		Yes	0	68	100.0
	Overall Percentage				57.6

a. Constant is included in the model.

b. The cut value is .500

Variables in the Equation

		B	S.E.	Wald	df	Sig.	Exp(B)
Step 0	Constant	.307	.186	2.724	1	.099	1.360

Variables not in the Equation

			Score	df	Sig.
Step 0	Variables	MedicalIssue(1)	1.620	1	.203
		Sympathectomy.Level(1)	4.108	1	.043
		Hospital.stay	2.131	1	.144
		Follow.up	2.767	1	.096
		Reduction.of.PH	2.551	1	.110
	Overall Statistics		9.011	5	.109

Block 1: Method = Forward Stepwise (Likelihood Ratio)

Omnibus Tests of Model Coefficients

		Chi-square	df	Sig.
Step 1	Step	4.114	1	.043
	Block	4.114	1	.043
	Model	4.114	1	.043

Model Summary

Step	-2 Log likelihood	Cox & Snell R Square	Nagelkerke R Square
1	156.712[a]	.034	.046

a. Estimation terminated at iteration number 3 because parameter estimates changed by less than .001.

Hosmer and Lemeshow Test

Step	Chi-square	df	Sig.
1	.000	0	.

Contingency Table for Hosmer and Lemeshow Test

		CS = No		CS = Yes		
		Observed	Expected	Observed	Expected	Total
Step 1	1	27	27.000	24	24.000	51
	2	23	23.000	44	44.000	67

Classification Table[a]

Observed			Predicted CS No	Predicted CS Yes	Percentage Correct
Step 1	CS	No	27	23	54.0
		Yes	24	44	64.7
	Overall Percentage				60.2

a. The cut value is .500

Variables in the Equation

		B	S.E.	Wald	df	Sig.	Exp(B)	95% C.I.for EXP(B) Lower	95% C.I.for EXP(B) Upper
Step 1[a]	Sympathectomy.Level(1)	-.766	.381	4.054	1	.044	.465	.220	.980
	Constant	.649	.257	6.356	1	.012	1.913		

a. Variable(s) entered on step 1: Sympathectomy.Level.

Model if Term Removed

Variable		Model Log Likelihood	Change in -2 Log Likelihood	df	Sig. of the Change
Step 1	Sympathectomy.Level	-80.413	4.114	1	.043

Variables not in the Equation

			Score	df	Sig.
Step 1	Variables	MedicalIssue(1)	1.715	1	.190
		Hospital.stay	1.228	1	.268
		Follow.up	1.850	1	.174
		Reduction.of.PH	1.724	1	.189
	Overall Statistics		4.966	4	.291

```
LOGISTIC REGRESSION VARIABLES Compensatory.sweating
  /METHOD=FSTEP(WALD) Medical.issues Sympathectomy.Level Hospital.stay Foll
ow.up Reduction.of.PH
  /CONTRAST (Medical.issues)=Indicator(1)
  /CONTRAST (Sympathectomy.Level)=Indicator(1)
  /PRINT=GOODFIT CI(95)
  /CRITERIA=PIN(0.05) POUT(0.10) ITERATE(20) CUT(0.5).
```

Logistic Regression

Notes

Output Created		18-APR-2018 18:57:...
Comments		
Input	Data	C:\Users\rnordin.ADMIN\Desktop\2018\PUBLICATION 2018 ETS\ETS.Data (Complete).sav 18APRIL2018.sav
	Active Dataset	DataSet1
	Filter	<none>
	Weight	<none>
	Split File	<none>
	N of Rows in Working Data File	118
Missing Value Handling	Definition of Missing	User-defined missing values are treated as missing
Syntax		LOGISTIC REGRESSION VARIABLES Compensatory.sweating /METHOD=FSTEP (WALD) Medical.issues Sympathectomy.Level Hospital.stay Follow.up Reduction.of.PH /CONTRAST (Medical.issues)=Indicator(1) /CONTRAST (Sympathectomy.Level)=Indicator(1) /PRINT=GOODFIT CI (95) /CRITERIA=PIN(0.05) POUT(0.10) ITERATE(20) CUT(0.5).
Resources	Processor Time	00:00:00.00
	Elapsed Time	00:00:00.01

Case Processing Summary

Unweighted Cases[a]		N	Percent
Selected Cases	Included in Analysis	118	100.0
	Missing Cases	0	.0
	Total	118	100.0
Unselected Cases		0	.0
Total		118	100.0

a. If weight is in effect, see classification table for the total number of cases.

Dependent Variable Encoding

Original Value	Internal Value
No	0
Yes	1

Categorical Variables Codings

		Frequency	Parameter coding (1)
Sympathectomy.Level	T2-T3	67	.000
	T2-T4	51	1.000
MedicalIssue	No	109	.000
	Yes	9	1.000

Block 0: Beginning Block

Classification Table[a,b]

Observed			Predicted CS No	Predicted CS Yes	Percentage Correct
Step 0	CS	No	0	50	.0
		Yes	0	68	100.0
	Overall Percentage				57.6

a. Constant is included in the model.

b. The cut value is .500

Variables in the Equation

		B	S.E.	Wald	df	Sig.	Exp(B)
Step 0	Constant	.307	.186	2.724	1	.099	1.360

Variables not in the Equation

			Score	df	Sig.
Step 0	Variables	MedicalIssue(1)	1.620	1	.203
		Sympathectomy.Level(1)	4.108	1	.043
		Hospital.stay	2.131	1	.144
		Follow.up	2.767	1	.096
		Reduction.of.PH	2.551	1	.110
	Overall Statistics		9.011	5	.109

Block 1: Method = Forward Stepwise (Wald)

Omnibus Tests of Model Coefficients

		Chi-square	df	Sig.
Step 1	Step	4.114	1	.043
	Block	4.114	1	.043
	Model	4.114	1	.043

Model Summary

Step	-2 Log likelihood	Cox & Snell R Square	Nagelkerke R Square
1	156.712[a]	.034	.046

a. Estimation terminated at iteration number 3 because parameter estimates changed by less than .001.

Hosmer and Lemeshow Test

Step	Chi-square	df	Sig.
1	.000	0	.

Contingency Table for Hosmer and Lemeshow Test

		CS = No		CS = Yes		
		Observed	Expected	Observed	Expected	Total
Step 1	1	27	27.000	24	24.000	51
	2	23	23.000	44	44.000	67

Classification Table[a]

Observed			Predicted CS No	Predicted CS Yes	Percentage Correct
Step 1	CS	No	27	23	54.0
		Yes	24	44	64.7
	Overall Percentage				60.2

a. The cut value is .500

Variables in the Equation

		B	S.E.	Wald	df	Sig.	Exp(B)	95% C.I.for EXP(B) Lower	95% C.I.for EXP(B) Upper
Step 1[a]	Sympathectomy.Level(1)	-.766	.381	4.054	1	.044	.465	.220	.980
	Constant	.649	.257	6.356	1	.012	1.913		

a. Variable(s) entered on step 1: Sympathectomy.Level.

Variables not in the Equation

			Score	df	Sig.
Step 1	Variables	MedicalIssue(1)	1.715	1	.190
		Hospital.stay	1.228	1	.268
		Follow.up	1.850	1	.174
		Reduction.of.PH	1.724	1	.189
	Overall Statistics		4.966	4	.291

```
LOGISTIC REGRESSION VARIABLES Compensatory.sweating
  /METHOD=BSTEP(COND) Medical.issues Sympathectomy.Level Hospital.stay Foll
ow.up Reduction.of.PH
  /CONTRAST (Medical.issues)=Indicator(1)
  /CONTRAST (Sympathectomy.Level)=Indicator(1)
  /PRINT=GOODFIT CI(95)
  /CRITERIA=PIN(0.05) POUT(0.10) ITERATE(20) CUT(0.5).
```

Logistic Regression

Notes

Output Created		18-APR-2018 18:57:...
Comments		
Input	Data	C:\Users\rnordin.ADMIN\Desktop\2018\PUBLICATION 2018 ETS\ETS.Data (Complete).sav 18APRIL2018.sav
	Active Dataset	DataSet1
	Filter	<none>
	Weight	<none>
	Split File	<none>
	N of Rows in Working Data File	118
Missing Value Handling	Definition of Missing	User-defined missing values are treated as missing

Notes

Syntax		LOGISTIC REGRESSION VARIABLES Compensatory.sweating<br>/METHOD=BSTEP (COND) Medical.issues Sympathectomy.Level Hospital.stay Follow.up Reduction.of.PH<br>/CONTRAST (Medical.issues)=Indicator(1)<br>/CONTRAST (Sympathectomy.Level) =Indicator(1)<br>/PRINT=GOODFIT CI (95)<br>/CRITERIA=PIN(0.05) POUT(0.10) ITERATE (20) CUT(0.5).
Resources	Processor Time	00:00:00.03
	Elapsed Time	00:00:00.04

Case Processing Summary

Unweighted Cases[a]		N	Percent
Selected Cases	Included in Analysis	118	100.0
	Missing Cases	0	.0
	Total	118	100.0
Unselected Cases		0	.0
Total		118	100.0

a. If weight is in effect, see classification table for the total number of cases.

Dependent Variable Encoding

Original Value	Internal Value
No	0
Yes	1

Categorical Variables Codings

		Frequency	Parameter coding (1)
Sympathectomy.Level	T2-T3	67	.000
	T2-T4	51	1.000
MedicalIssue	No	109	.000
	Yes	9	1.000

Block 0: Beginning Block

Classification Table[a,b]

Observed			Predicted CS No	Predicted CS Yes	Percentage Correct
Step 0	CS	No	0	50	.0
		Yes	0	68	100.0
	Overall Percentage				57.6

a. Constant is included in the model.

b. The cut value is .500

Variables in the Equation

		B	S.E.	Wald	df	Sig.	Exp(B)
Step 0	Constant	.307	.186	2.724	1	.099	1.360

Variables not in the Equation

			Score	df	Sig.
Step 0	Variables	MedicalIssue(1)	1.620	1	.203
		Sympathectomy.Level(1)	4.108	1	.043
		Hospital.stay	2.131	1	.144
		Follow.up	2.767	1	.096
		Reduction.of.PH	2.551	1	.110
	Overall Statistics		9.011	5	.109

Block 1: Method = Backward Stepwise (Conditional)

Omnibus Tests of Model Coefficients

		Chi-square	df	Sig.
Step 1	Step	9.962	5	.076
	Block	9.962	5	.076
	Model	9.962	5	.076
Step 2[a]	Step	-.009	1	.923
	Block	9.953	4	.041
	Model	9.953	4	.041
Step 3[a]	Step	-1.533	1	.216
	Block	8.421	3	.038
	Model	8.421	3	.038
Step 4[a]	Step	-1.690	1	.194
	Block	6.731	2	.035
	Model	6.731	2	.035
Step 5[a]	Step	-2.616	1	.106
	Block	4.114	1	.043
	Model	4.114	1	.043

a. A negative Chi-squares value indicates that the Chi-squares value has decreased from the previous step.

Model Summary

Step	-2 Log likelihood	Cox & Snell R Square	Nagelkerke R Square
1	150.864[a]	.081	.109
2	150.873[a]	.081	.109
3	152.406[a]	.069	.093
4	154.095[a]	.055	.075
5	156.712[b]	.034	.046

a. Estimation terminated at iteration number 20 because maximum iterations has been reached. Final solution cannot be found.

b. Estimation terminated at iteration number 3 because parameter estimates changed by less than .001.

Hosmer and Lemeshow Test

Step	Chi-square	df	Sig.
1	3.076	5	.688
2	6.088	5	.298
3	.004	2	.998
4	.000	1	1.000
5	.000	0	.

Contingency Table for Hosmer and Lemeshow Test

		CS = No		CS = Yes		
		Observed	Expected	Observed	Expected	Total
Step 1	1	8	7.852	3	3.148	11
	2	5	6.124	6	4.876	11
	3	13	12.115	12	12.885	25
	4	8	7.546	11	11.454	19
	5	2	1.079	1	1.921	3
	6	13	12.760	25	25.240	38
	7	1	2.524	10	8.476	11
Step 2	1	8	9.625	6	4.375	14
	2	5	4.339	3	3.661	8
	3	13	12.129	12	12.871	25
	4	8	7.560	11	11.440	19
	5	2	.711	0	1.289	2
	6	13	13.113	26	25.887	39
	7	1	2.524	10	8.476	11
Step 3	1	2	2.000	0	.000	2
	2	24	23.840	21	21.160	45
	3	22	22.160	40	39.840	62
	4	2	2.000	7	7.000	9
Step 4	1	2	2.000	0	.000	2
	2	25	25.000	24	24.000	49
	3	23	23.000	44	44.000	67
Step 5	1	27	27.000	24	24.000	51
	2	23	23.000	44	44.000	67

Classification Table[a]

Observed			Predicted CS No	Predicted CS Yes	Percentage Correct
Step 1	CS	No	13	37	26.0
		Yes	9	59	86.8
	Overall Percentage				61.0
Step 2	CS	No	13	37	26.0
		Yes	9	59	86.8
	Overall Percentage				61.0
Step 3	CS	No	26	24	52.0
		Yes	21	47	69.1
	Overall Percentage				61.9
Step 4	CS	No	27	23	54.0
		Yes	24	44	64.7
	Overall Percentage				60.2
Step 5	CS	No	27	23	54.0
		Yes	24	44	64.7
	Overall Percentage				60.2

a. The cut value is .500

Variables in the Equation

		B	S.E.	Wald	df	Sig.	Exp(B)	95% C.I.for EXP(B) Lower	Upper
Step 1[a]	MedicalIssue(1)	1.072	.842	1.622	1	.203	2.922	.561	15.212
	Sympathectomy.Level(1)	-.620	.396	2.454	1	.117	.538	.247	1.169
	Hospital.stay	-.228	.189	1.463	1	.226	.796	.550	1.152
	Follow.up	-20.866	28379.274	.000	1	.999	.000	.000	.
	Reduction.of.PH	-.072	.741	.009	1	.923	.931	.218	3.976
	Constant	22.305	28379.274	.000	1	.999	4.861E+9		
Step 2[a]	MedicalIssue(1)	1.075	.841	1.634	1	.201	2.931	.563	15.242
	Sympathectomy.Level(1)	-.621	.396	2.457	1	.117	.537	.247	1.168
	Hospital.stay	-.229	.188	1.479	1	.224	.795	.550	1.150
	Follow.up	-21.151	28378.949	.000	1	.999	.000	.000	.
	Constant	22.519	28378.949	.000	1	.999	6.022E+9		
Step 3[a]	MedicalIssue(1)	1.014	.835	1.474	1	.225	2.757	.536	14.170
	Sympathectomy.Level(1)	-.706	.388	3.312	1	.069	.494	.231	1.056
	Follow.up	-21.084	28420.696	.000	1	.999	.000	.000	.
	Constant	21.670	28420.696	.000	1	.999	2.578E+9		
Step 4[a]	Sympathectomy.Level(1)	-.690	.385	3.215	1	.073	.502	.236	1.066
	Follow.up	-21.162	28420.655	.000	1	.999	.000	.000	.
	Constant	21.811	28420.655	.000	1	.999	2.967E+9		
Step 5[a]	Sympathectomy.Level(1)	-.766	.381	4.054	1	.044	.465	.220	.980
	Constant	.649	.257	6.356	1	.012	1.913		

a. Variable(s) entered on step 1: MedicalIssue, Sympathectomy.Level, Hospital.stay, Follow.up, Reduction.of.PH.

Model if Term Removed[a]

Variable		Model Log Likelihood	Change in -2 Log Likelihood	df	Sig. of the Change
Step 1	MedicalIssue	-76.368	1.872	1	.171
	Sympathectomy.Level	-76.665	2.466	1	.116
	Hospital.stay	-76.190	1.515	1	.218
	Follow.up	-76.510	2.156	1	.142
	Reduction.of.PH	-75.437	.009	1	.923
Step 2	MedicalIssue	-76.380	1.886	1	.170
	Sympathectomy.Level	-76.671	2.469	1	.116
	Hospital.stay	-76.203	1.533	1	.216
	Follow.up	-76.772	2.670	1	.102
Step 3	MedicalIssue	-77.050	1.694	1	.193
	Sympathectomy.Level	-77.881	3.356	1	.067
	Follow.up	-77.473	2.541	1	.111
Step 4	Sympathectomy.Level	-78.673	3.251	1	.071
	Follow.up	-78.394	2.692	1	.101
Step 5	Sympathectomy.Level	-80.414	4.117	1	.042

a. Based on conditional parameter estimates

Variables not in the Equation

			Score	df	Sig.
Step 2[a]	Variables	Reduction.of.PH	.009	1	.923
	Overall Statistics		.009	1	.923
Step 3[b]	Variables	Hospital.stay	1.545	1	.214
		Reduction.of.PH	.027	1	.869
	Overall Statistics		1.554	2	.460
Step 4[c]	Variables	MedicalIssue(1)	1.575	1	.210
		Hospital.stay	1.358	1	.244
		Reduction.of.PH	.046	1	.830
	Overall Statistics		3.118	3	.374
Step 5[d]	Variables	MedicalIssue(1)	1.715	1	.190
		Hospital.stay	1.228	1	.268
		Follow.up	1.850	1	.174
		Reduction.of.PH	1.724	1	.189
	Overall Statistics		4.966	4	.291

a. Variable(s) removed on step 2: Reduction.of.PH.

b. Variable(s) removed on step 3: Hospital.stay.

c. Variable(s) removed on step 4: MedicalIssue.

d. Variable(s) removed on step 5: Follow.up.

```
LOGISTIC REGRESSION VARIABLES Compensatory.sweating
  /METHOD=BSTEP(LR) Medical.issues Sympathectomy.Level Hospital.stay Follow
.up Reduction.of.PH
  /CONTRAST (Medical.issues)=Indicator(1)
  /CONTRAST (Sympathectomy.Level)=Indicator(1)
  /PRINT=GOODFIT CI(95)
  /CRITERIA=PIN(0.05) POUT(0.10) ITERATE(20) CUT(0.5).
```

Logistic Regression

Notes

Output Created		18-APR-2018 18:57:...
Comments		
Input	Data	C:\Users\rnordin.ADMIN\Desktop\2018\PUBLICATION 2018 ETS\ETS.Data (Complete).sav 18APRIL2018.sav
	Active Dataset	DataSet1
	Filter	<none>
	Weight	<none>
	Split File	<none>
	N of Rows in Working Data File	118
Missing Value Handling	Definition of Missing	User-defined missing values are treated as missing
Syntax		LOGISTIC REGRESSION VARIABLES Compensatory.sweating /METHOD=BSTEP(LR) Medical.issues Sympathectomy.Level Hospital.stay Follow.up Reduction.of.PH /CONTRAST (Medical.issues)=Indicator(1) /CONTRAST (Sympathectomy.Level)=Indicator(1) /PRINT=GOODFIT CI(95) /CRITERIA=PIN(0.05) POUT(0.10) ITERATE(20) CUT(0.5).
Resources	Processor Time	00:00:00.03
	Elapsed Time	00:00:00.04

Case Processing Summary

Unweighted Cases[a]		N	Percent
Selected Cases	Included in Analysis	118	100.0
	Missing Cases	0	.0
	Total	118	100.0
Unselected Cases		0	.0
Total		118	100.0

a. If weight is in effect, see classification table for the total number of cases.

Dependent Variable Encoding

Original Value	Internal Value
No	0
Yes	1

Categorical Variables Codings

		Frequency	Parameter coding (1)
Sympathectomy.Level	T2-T3	67	.000
	T2-T4	51	1.000
MedicalIssue	No	109	.000
	Yes	9	1.000

Block 0: Beginning Block

Classification Table[a,b]

Observed			Predicted CS No	Predicted CS Yes	Percentage Correct
Step 0	CS	No	0	50	.0
		Yes	0	68	100.0
	Overall Percentage				57.6

a. Constant is included in the model.

b. The cut value is .500

Variables in the Equation

		B	S.E.	Wald	df	Sig.	Exp(B)
Step 0	Constant	.307	.186	2.724	1	.099	1.360

Variables not in the Equation

			Score	df	Sig.
Step 0	Variables	MedicalIssue(1)	1.620	1	.203
		Sympathectomy.Level(1)	4.108	1	.043
		Hospital.stay	2.131	1	.144
		Follow.up	2.767	1	.096
		Reduction.of.PH	2.551	1	.110
	Overall Statistics		9.011	5	.109

Block 1: Method = Backward Stepwise (Likelihood Ratio)

Omnibus Tests of Model Coefficients

		Chi-square	df	Sig.
Step 1	Step	9.962	5	.076
	Block	9.962	5	.076
	Model	9.962	5	.076
Step 2[a]	Step	-.009	1	.923
	Block	9.953	4	.041
	Model	9.953	4	.041
Step 3[a]	Step	-1.533	1	.216
	Block	8.421	3	.038
	Model	8.421	3	.038
Step 4[a]	Step	-1.690	1	.194
	Block	6.731	2	.035
	Model	6.731	2	.035
Step 5[a]	Step	-2.616	1	.106
	Block	4.114	1	.043
	Model	4.114	1	.043

a. A negative Chi-squares value indicates that the Chi-squares value has decreased from the previous step.

Model Summary

Step	-2 Log likelihood	Cox & Snell R Square	Nagelkerke R Square
1	150.864[a]	.081	.109
2	150.873[a]	.081	.109
3	152.406[a]	.069	.093
4	154.095[a]	.055	.075
5	156.712[b]	.034	.046

a. Estimation terminated at iteration number 20 because maximum iterations has been reached. Final solution cannot be found.

b. Estimation terminated at iteration number 3 because parameter estimates changed by less than .001.

Hosmer and Lemeshow Test

Step	Chi-square	df	Sig.
1	3.076	5	.688
2	6.088	5	.298
3	.004	2	.998
4	.000	1	1.000
5	.000	0	.

Contingency Table for Hosmer and Lemeshow Test

		CS = No		CS = Yes		
		Observed	Expected	Observed	Expected	Total
Step 1	1	8	7.852	3	3.148	11
	2	5	6.124	6	4.876	11
	3	13	12.115	12	12.885	25
	4	8	7.546	11	11.454	19
	5	2	1.079	1	1.921	3
	6	13	12.760	25	25.240	38
	7	1	2.524	10	8.476	11
Step 2	1	8	9.625	6	4.375	14
	2	5	4.339	3	3.661	8
	3	13	12.129	12	12.871	25
	4	8	7.560	11	11.440	19
	5	2	.711	0	1.289	2
	6	13	13.113	26	25.887	39
	7	1	2.524	10	8.476	11
Step 3	1	2	2.000	0	.000	2
	2	24	23.840	21	21.160	45
	3	22	22.160	40	39.840	62
	4	2	2.000	7	7.000	9
Step 4	1	2	2.000	0	.000	2
	2	25	25.000	24	24.000	49
	3	23	23.000	44	44.000	67
Step 5	1	27	27.000	24	24.000	51
	2	23	23.000	44	44.000	67

Classification Table[a]

Observed			Predicted: CS No	Predicted: CS Yes	Percentage Correct
Step 1	CS	No	13	37	26.0
		Yes	9	59	86.8
	Overall Percentage				61.0
Step 2	CS	No	13	37	26.0
		Yes	9	59	86.8
	Overall Percentage				61.0
Step 3	CS	No	26	24	52.0
		Yes	21	47	69.1
	Overall Percentage				61.9
Step 4	CS	No	27	23	54.0
		Yes	24	44	64.7
	Overall Percentage				60.2
Step 5	CS	No	27	23	54.0
		Yes	24	44	64.7
	Overall Percentage				60.2

a. The cut value is .500

Variables in the Equation

		B	S.E.	Wald	df	Sig.	Exp(B)	95% C.I.for EXP(B)	
								Lower	Upper
Step 1[a]	MedicalIssue(1)	1.072	.842	1.622	1	.203	2.922	.561	15.212
	Sympathectomy.Level(1)	-.620	.396	2.454	1	.117	.538	.247	1.169
	Hospital.stay	-.228	.189	1.463	1	.226	.796	.550	1.152
	Follow.up	-20.866	28379.274	.000	1	.999	.000	.000	.
	Reduction.of.PH	-.072	.741	.009	1	.923	.931	.218	3.976
	Constant	22.305	28379.274	.000	1	.999	4.861E+9		
Step 2[a]	MedicalIssue(1)	1.075	.841	1.634	1	.201	2.931	.563	15.242
	Sympathectomy.Level(1)	-.621	.396	2.457	1	.117	.537	.247	1.168
	Hospital.stay	-.229	.188	1.479	1	.224	.795	.550	1.150
	Follow.up	-21.151	28378.949	.000	1	.999	.000	.000	.
	Constant	22.519	28378.949	.000	1	.999	6.022E+9		
Step 3[a]	MedicalIssue(1)	1.014	.835	1.474	1	.225	2.757	.536	14.170
	Sympathectomy.Level(1)	-.706	.388	3.312	1	.069	.494	.231	1.056
	Follow.up	-21.084	28420.696	.000	1	.999	.000	.000	.
	Constant	21.670	28420.696	.000	1	.999	2.578E+9		
Step 4[a]	Sympathectomy.Level(1)	-.690	.385	3.215	1	.073	.502	.236	1.066
	Follow.up	-21.162	28420.655	.000	1	.999	.000	.000	.
	Constant	21.811	28420.655	.000	1	.999	2.967E+9		
Step 5[a]	Sympathectomy.Level(1)	-.766	.381	4.054	1	.044	.465	.220	.980
	Constant	.649	.257	6.356	1	.012	1.913		

a. Variable(s) entered on step 1: MedicalIssue, Sympathectomy.Level, Hospital.stay, Follow.up, Reduction.of.PH.

Model if Term Removed

Variable		Model Log Likelihood	Change in -2 Log Likelihood	df	Sig. of the Change
Step 1	MedicalIssue	-76.365	1.867	1	.172
	Sympathectomy.Level	-76.663	2.463	1	.117
	Hospital.stay	-76.189	1.515	1	.218
	Follow.up	-75.836	.809	1	.368
	Reduction.of.PH	-75.437	.009	1	.923
Step 2	MedicalIssue	-76.377	1.881	1	.170
	Sympathectomy.Level	-76.670	2.467	1	.116
	Hospital.stay	-76.203	1.533	1	.216
	Follow.up	-76.731	2.588	1	.108
Step 3	MedicalIssue	-77.048	1.690	1	.194
	Sympathectomy.Level	-77.879	3.353	1	.067
	Follow.up	-77.437	2.468	1	.116
Step 4	Sympathectomy.Level	-78.672	3.249	1	.071
	Follow.up	-78.356	2.616	1	.106
Step 5	Sympathectomy.Level	-80.413	4.114	1	.043

Variables not in the Equation

			Score	df	Sig.
Step 2[a]	Variables	Reduction.of.PH	.009	1	.923
	Overall Statistics		.009	1	.923
Step 3[b]	Variables	Hospital.stay	1.545	1	.214
		Reduction.of.PH	.027	1	.869
	Overall Statistics		1.554	2	.460
Step 4[c]	Variables	MedicalIssue(1)	1.575	1	.210
		Hospital.stay	1.358	1	.244
		Reduction.of.PH	.046	1	.830
	Overall Statistics		3.118	3	.374
Step 5[d]	Variables	MedicalIssue(1)	1.715	1	.190
		Hospital.stay	1.228	1	.268
		Follow.up	1.850	1	.174
		Reduction.of.PH	1.724	1	.189
	Overall Statistics		4.966	4	.291

a. Variable(s) removed on step 2: Reduction.of.PH.

b. Variable(s) removed on step 3: Hospital.stay.

c. Variable(s) removed on step 4: MedicalIssue.

d. Variable(s) removed on step 5: Follow.up.

```
LOGISTIC REGRESSION VARIABLES Compensatory.sweating
  /METHOD=BSTEP(WALD) Medical.issues Sympathectomy.Level Hospital.stay Foll
ow.up Reduction.of.PH
  /CONTRAST (Medical.issues)=Indicator(1)
  /CONTRAST (Sympathectomy.Level)=Indicator(1)
  /PRINT=GOODFIT CI(95)
  /CRITERIA=PIN(0.05) POUT(0.10) ITERATE(20) CUT(0.5).
```

Logistic Regression

Notes

Output Created		18-APR-2018 18:58:...
Comments		
Input	Data	C:\Users\rnordin.ADMIN\Desktop\2018\PUBLICATION 2018 ETS\ETS.Data (Complete).sav 18APRIL2018.sav
	Active Dataset	DataSet1
	Filter	<none>
	Weight	<none>
	Split File	<none>
	N of Rows in Working Data File	118
Missing Value Handling	Definition of Missing	User-defined missing values are treated as missing
Syntax		LOGISTIC REGRESSION VARIABLES Compensatory.sweating /METHOD=BSTEP (WALD) Medical.issues Sympathectomy.Level Hospital.stay Follow.up Reduction.of.PH /CONTRAST (Medical.issues)=Indicator(1) /CONTRAST (Sympathectomy.Level)=Indicator(1) /PRINT=GOODFIT CI(95) /CRITERIA=PIN(0.05) POUT(0.10) ITERATE(20) CUT(0.5).
Resources	Processor Time	00:00:00.02
	Elapsed Time	00:00:00.02

Case Processing Summary

Unweighted Cases[a]		N	Percent
Selected Cases	Included in Analysis	118	100.0
	Missing Cases	0	.0
	Total	118	100.0
Unselected Cases		0	.0
Total		118	100.0

a. If weight is in effect, see classification table for the total number of cases.

Dependent Variable Encoding

Original Value	Internal Value
No	0
Yes	1

Categorical Variables Codings

		Frequency	Parameter coding (1)
Sympathectomy.Level	T2-T3	67	.000
	T2-T4	51	1.000
MedicalIssue	No	109	.000
	Yes	9	1.000

Block 0: Beginning Block

Classification Table[a,b]

Observed			Predicted CS No	Predicted CS Yes	Percentage Correct
Step 0	CS	No	0	50	.0
		Yes	0	68	100.0
	Overall Percentage				57.6

a. Constant is included in the model.

b. The cut value is .500

Variables in the Equation

		B	S.E.	Wald	df	Sig.	Exp(B)
Step 0	Constant	.307	.186	2.724	1	.099	1.360

Variables not in the Equation

			Score	df	Sig.
Step 0	Variables	MedicalIssue(1)	1.620	1	.203
		Sympathectomy.Level(1)	4.108	1	.043
		Hospital.stay	2.131	1	.144
		Follow.up	2.767	1	.096
		Reduction.of.PH	2.551	1	.110
	Overall Statistics		9.011	5	.109

Block 1: Method = Backward Stepwise (Wald)

Omnibus Tests of Model Coefficients

		Chi-square	df	Sig.
Step 1	Step	9.962	5	.076
	Block	9.962	5	.076
	Model	9.962	5	.076
Step 2[a]	Step	-.809	1	.368
	Block	9.153	4	.057
	Model	9.153	4	.057
Step 3[a]	Step	-1.788	1	.181
	Block	7.365	3	.061
	Model	7.365	3	.061
Step 4[a]	Step	-1.412	1	.235
	Block	5.953	2	.051
	Model	5.953	2	.051
Step 5[a]	Step	-1.838	1	.175
	Block	4.114	1	.043
	Model	4.114	1	.043

a. A negative Chi-squares value indicates that the Chi-squares value has decreased from the previous step.

Model Summary

Step	-2 Log likelihood	Cox & Snell R Square	Nagelkerke R Square
1	150.864[a]	.081	.109
2	151.673[b]	.075	.100
3	153.461[b]	.061	.081
4	154.874[b]	.049	.066
5	156.712[c]	.034	.046

a. Estimation terminated at iteration number 20 because maximum iterations has been reached. Final solution cannot be found.

b. Estimation terminated at iteration number 4 because parameter estimates changed by less than .001.

c. Estimation terminated at iteration number 3 because parameter estimates changed by less than .001.

Hosmer and Lemeshow Test

Step	Chi-square	df	Sig.
1	3.076	5	.688
2	5.986	5	.308
3	5.007	5	.415
4	.006	1	.940
5	.000	0	.

Contingency Table for Hosmer and Lemeshow Test

		CS = No		CS = Yes		
		Observed	Expected	Observed	Expected	Total
Step 1	1	8	7.852	3	3.148	11
	2	5	6.124	6	4.876	11
	3	13	12.115	12	12.885	25
	4	8	7.546	11	11.454	19
	5	2	1.079	1	1.921	3
	6	13	12.760	25	25.240	38
	7	1	2.524	10	8.476	11
Step 2	1	8	7.796	3	3.204	11
	2	5	6.711	7	5.289	12
	3	13	12.156	12	12.844	25
	4	8	7.451	11	11.549	19
	5	2	.715	0	1.285	2
	6	13	12.651	25	25.349	38
	7	1	2.520	10	8.480	11
Step 3	1	6	7.776	6	4.224	12
	2	6	5.058	3	3.942	9
	3	14	13.194	12	12.806	26
	4	9	7.936	11	12.064	20
	5	1	.343	0	.657	1
	6	13	13.165	26	25.835	39
	7	1	2.528	10	8.472	11
Step 4	1	26	25.811	21	21.189	47
	2	22	22.189	40	39.811	62
	3	2	2.000	7	7.000	9
Step 5	1	27	27.000	24	24.000	51
	2	23	23.000	44	44.000	67

Classification Table[a]

Observed			Predicted CS No	Predicted CS Yes	Percentage Correct
Step 1	CS	No	13	37	26.0
		Yes	9	59	86.8
	Overall Percentage				61.0
Step 2	CS	No	13	37	26.0
		Yes	10	58	85.3
	Overall Percentage				60.2
Step 3	CS	No	26	24	52.0
		Yes	21	47	69.1
	Overall Percentage				61.9
Step 4	CS	No	26	24	52.0
		Yes	21	47	69.1
	Overall Percentage				61.9
Step 5	CS	No	27	23	54.0
		Yes	24	44	64.7
	Overall Percentage				60.2

a. The cut value is .500

Variables in the Equation

		B	S.E.	Wald	df	Sig.	Exp(B)	95% C.I.for EXP(B) Lower	Upper
Step 1[a]	MedicalIssue(1)	1.072	.842	1.622	1	.203	2.922	.561	15.212
	Sympathectomy.Level(1)	-.620	.396	2.454	1	.117	.538	.247	1.169
	Hospital.stay	-.228	.189	1.463	1	.226	.796	.550	1.152
	Follow.up	-20.866	28379.274	.000	1	.999	.000	.000	.
	Reduction.of.PH	-.072	.741	.009	1	.923	.931	.218	3.976
	Constant	22.305	28379.274	.000	1	.999	4.861E+9		
Step 2[a]	MedicalIssue(1)	1.064	.842	1.596	1	.206	2.898	.556	15.097
	Sympathectomy.Level(1)	-.640	.396	2.613	1	.106	.527	.243	1.146
	Hospital.stay	-.221	.188	1.384	1	.239	.802	.554	1.159
	Reduction.of.PH	-.534	.479	1.247	1	.264	.586	.229	1.497
	Constant	1.893	.854	4.915	1	.027	6.639		
Step 3[a]	MedicalIssue(1)	1.119	.843	1.763	1	.184	3.063	.587	15.987
	Sympathectomy.Level(1)	-.704	.391	3.234	1	.072	.495	.230	1.065
	Hospital.stay	-.220	.188	1.367	1	.242	.803	.556	1.160
	Constant	1.333	.693	3.699	1	.054	3.793		
Step 4[a]	MedicalIssue(1)	1.058	.837	1.597	1	.206	2.879	.558	14.845
	Sympathectomy.Level(1)	-.782	.384	4.144	1	.042	.458	.216	.971
	Constant	.585	.262	4.992	1	.025	1.794		
Step 5[a]	Sympathectomy.Level(1)	-.766	.381	4.054	1	.044	.465	.220	.980
	Constant	.649	.257	6.356	1	.012	1.913		

a. Variable(s) entered on step 1: MedicalIssue, Sympathectomy.Level, Hospital.stay, Follow.up, Reduction.of.PH.

Variables not in the Equation

			Score	df	Sig.
Step 2[a]	Variables	Follow.up	.642	1	.423
	Overall Statistics		.642	1	.423
Step 3[b]	Variables	Follow.up	1.824	1	.177
		Reduction.of.PH	1.575	1	.209
	Overall Statistics		1.833	2	.400
Step 4[c]	Variables	Hospital.stay	1.423	1	.233
		Follow.up	1.713	1	.191
		Reduction.of.PH	1.546	1	.214
	Overall Statistics		3.265	3	.353
Step 5[d]	Variables	MedicalIssue(1)	1.715	1	.190
		Hospital.stay	1.228	1	.268
		Follow.up	1.850	1	.174
		Reduction.of.PH	1.724	1	.189
	Overall Statistics		4.966	4	.291

a. Variable(s) removed on step 2: Follow.up.

b. Variable(s) removed on step 3: Reduction.of.PH.

c. Variable(s) removed on step 4: Hospital.stay.

d. Variable(s) removed on step 5: MedicalIssue.

```
 SORT CASES BY Sympathectomy.Level(A).
DATASET ACTIVATE DataSet1.

SAVE OUTFILE='C:\Users\rnordin.ADMIN\Desktop\2018\PUBLICATION 2018 ETS\ETS.
Data(Complete).sav '+
    '18APRIL2018.sav'
  /COMPRESSED.
SORT CASES BY Compensatory.sweating(A).
DATASET ACTIVATE DataSet1.

SAVE OUTFILE='C:\Users\rnordin.ADMIN\Desktop\2018\PUBLICATION 2018 ETS\ETS.
Data(Complete).sav '+
    '18APRIL2018.sav'
  /COMPRESSED.
DATASET ACTIVATE DataSet1.

SAVE OUTFILE='C:\Users\rnordin.ADMIN\Desktop\2018\PUBLICATION 2018 ETS\ETS.
Data(Complete).sav '+
    '18APRIL2018.sav'
  /COMPRESSED.
SORT CASES BY Sympathectomy.Level(A).
DATASET ACTIVATE DataSet1.

SAVE OUTFILE='C:\Users\rnordin.ADMIN\Desktop\2018\PUBLICATION 2018 ETS\ETS.
Data(Complete).sav '+
    '18APRIL2018.sav'
  /COMPRESSED.
LOGISTIC REGRESSION VARIABLES Compensatory.sweating
  /METHOD=ENTER Medical.issues Hospital.stay Follow.up Reduction.of.PH Symp
athectomy.Level
  /CONTRAST (Medical.issues)=Indicator(1)
  /CONTRAST (Sympathectomy.Level)=Indicator(1)
  /CONTRAST (Hospital.stay)=Indicator(1)
  /CONTRAST (Follow.up)=Indicator(1)
  /CONTRAST (Reduction.of.PH)=Indicator(1)
  /PRINT=GOODFIT CI(95)
  /CRITERIA=PIN(0.05) POUT(0.10) ITERATE(20) CUT(0.5).
```

Logistic Regression

Notes

Output Created		18-APR-2018 19:09:...
Comments		
Input	Data	C:\Users\rnordin.ADMIN\Desktop\2018\PUBLICATION 2018 ETS\ETS.Data (Complete).sav 18APRIL2018.sav
	Active Dataset	DataSet1
	Filter	<none>
	Weight	<none>
	Split File	<none>
	N of Rows in Working Data File	118
Missing Value Handling	Definition of Missing	User-defined missing values are treated as missing
Syntax		LOGISTIC REGRESSION VARIABLES Compensatory.sweating /METHOD=ENTER Medical.issues Hospital.stay Follow.up Reduction.of.PH Sympathectomy.Level /CONTRAST (Medical.issues)=Indicator(1) /CONTRAST (Sympathectomy.Level)=Indicator(1) /CONTRAST (Hospital.stay)=Indicator(1) /CONTRAST (Follow.up)=Indicator(1) /CONTRAST (Reduction.of.PH)=Indicator(1) /PRINT=GOODFIT CI(95) /CRITERIA=PIN(0.05) POUT(0.10) ITERATE(20) CUT(0.5).
Resources	Processor Time	00:00:00.02
	Elapsed Time	00:00:00.01

Warnings

Due to redundancies, degrees of freedom have been reduced for one or more variables.

Case Processing Summary

Unweighted Cases[a]		N	Percent
Selected Cases	Included in Analysis	118	100.0
	Missing Cases	0	.0
	Total	118	100.0
Unselected Cases		0	.0
Total		118	100.0

a. If weight is in effect, see classification table for the total number of cases.

Dependent Variable Encoding

Original Value	Internal Value
No	0
Yes	1

Categorical Variables Codings

		Frequency	Parameter coding					
			(1)	(2)	(3)	(4)	(5)	(6)
Hospital.stay	1.00	1	.000	.000	.000	.000	.000	.000
	2.00	3	1.000	.000	.000	.000	.000	.000
	3.00	68	.000	1.000	.000	.000	.000	.000
	4.00	30	.000	.000	1.000	.000	.000	.000
	5.00	8	.000	.000	.000	1.000	.000	.000
	6.00	7	.000	.000	.000	.000	1.000	.000
	9.00	1	.000	.000	.000	.000	.000	1.000
Reduction.of.PH	Complete (95-100%)	114	.000	.000				
	No change	2	1.000	.000				
	N/A	2	.000	1.000				
Sympathectomy.Level	T2-T4	51	.000					
	T2-T3	67	1.000					
Follow.up	Yes	116	.000					
	No	2	1.000					
MedicalIssue	No	109	.000					
	Yes	9	1.000					

Block 0: Beginning Block

Classification Table[a,b]

Observed			Predicted CS No	Predicted CS Yes	Percentage Correct
Step 0	CS	No	0	50	.0
		Yes	0	68	100.0
	Overall Percentage				57.6

a. Constant is included in the model.

b. The cut value is .500

Variables in the Equation

		B	S.E.	Wald	df	Sig.	Exp(B)
Step 0	Constant	.307	.186	2.724	1	.099	1.360

Variables not in the Equation[a]

			Score	df	Sig.
Step 0	Variables	MedicalIssue(1)	1.620	1	.203
		Hospital.stay	5.951	6	.429
		Hospital.stay(1)	2.263	1	.132
		Hospital.stay(2)	.467	1	.494
		Hospital.stay(3)	.015	1	.902
		Hospital.stay(4)	.204	1	.651
		Hospital.stay(5)	.665	1	.415
		Hospital.stay(6)	1.372	1	.242
		Follow.up(1)	2.767	1	.096
		Reduction.of.PH	2.829	2	.243
		Reduction.of.PH(1)	.048	1	.826
		Reduction.of.PH(2)	2.767	1	.096
		Sympathectomy.Level(1)	4.108	1	.043

a. Residual Chi-Squares are not computed because of redundancies.

Block 1: Method = Enter

Omnibus Tests of Model Coefficients

		Chi-square	df	Sig.
Step 1	Step	14.995	10	.132
	Block	14.995	10	.132
	Model	14.995	10	.132

Model Summary

Step	-2 Log likelihood	Cox & Snell R Square	Nagelkerke R Square
1	145.832[a]	.119	.160

a. Estimation terminated at iteration number 20 because maximum iterations has been reached. Final solution cannot be found.

Hosmer and Lemeshow Test

Step	Chi-square	df	Sig.
1	1.553	5	.907

Contingency Table for Hosmer and Lemeshow Test

		CS = No		CS = Yes		
		Observed	Expected	Observed	Expected	Total
Step 1	1	9	10.366	6	4.634	15
	2	5	4.357	3	3.643	8
	3	13	12.096	12	12.904	25
	4	8	7.728	11	11.272	19
	5	0	.380	1	.620	1
	6	13	13.073	25	24.927	38
	7	2	2.000	10	10.000	12

Classification Table[a]

Observed			Predicted		
			CS		Percentage Correct
			No	Yes	
Step 1	CS	No	14	36	28.0
		Yes	9	59	86.8
	Overall Percentage				61.9

a. The cut value is .500

Variables in the Equation

		B	S.E.	Wald	df	Sig.	Exp(B)	95% C.I.for EXP(B) Lower	Upper
Step 1[a]	MedicalIssue(1)	1.088	.861	1.594	1	.207	2.967	.549	16.048
	Hospital.stay			.532	6	.997			
	Hospital.stay(1)	41.825	46410.094	.000	1	.999	1.460E+18	.000	.
	Hospital.stay(2)	21.268	40192.104	.000	1	1.000	1.723E+9	.000	.
	Hospital.stay(3)	21.024	40192.104	.000	1	1.000	1.351E+9	.000	.
	Hospital.stay(4)	20.871	40192.104	.000	1	1.000	1.159E+9	.000	.
	Hospital.stay(5)	20.915	40192.104	.000	1	1.000	1.212E+9	.000	.
	Hospital.stay(6)	-.581	56840.831	.000	1	1.000	.560	.000	.
	Follow.up(1)	-21.150	28378.286	.000	1	.999	.000	.000	.
	Reduction.of.PH			.011	1	.917			
	Reduction.of.PH(1)	-.157	1.511	.011	1	.917	.855	.044	16.519
	Sympathectomy.Level(1)	.581	.417	1.937	1	.164	1.787	.789	4.049
	Constant	-21.203	40192.104	.000	1	1.000	.000		

a. Variable(s) entered on step 1: MedicalIssue, Hospital.stay, Follow.up, Reduction.of.PH, Sympathectomy.Level.

```
 LOGISTIC REGRESSION VARIABLES Compensatory.sweating
  /METHOD=FSTEP(COND) Medical.issues Hospital.stay Follow.up Reduction.of.P
H Sympathectomy.Level
  /CONTRAST (Medical.issues)=Indicator(1)
  /CONTRAST (Sympathectomy.Level)=Indicator(1)
  /CONTRAST (Hospital.stay)=Indicator(1)
  /CONTRAST (Follow.up)=Indicator(1)
  /CONTRAST (Reduction.of.PH)=Indicator(1)
  /PRINT=GOODFIT CI(95)
  /CRITERIA=PIN(0.05) POUT(0.10) ITERATE(20) CUT(0.5).
```

Logistic Regression

Notes

Output Created		18-APR-2018 19:10:...
Comments		
Input	Data	C:\Users\rnordin.ADMIN\Desktop\2018\PUBLICATION 2018 ETS\ETS.Data (Complete).sav 18APRIL2018.sav
	Active Dataset	DataSet1
	Filter	<none>
	Weight	<none>
	Split File	<none>
	N of Rows in Working Data File	118
Missing Value Handling	Definition of Missing	User-defined missing values are treated as missing
Syntax		LOGISTIC REGRESSION VARIABLES Compensatory.sweating /METHOD=FSTEP (COND) Medical.issues Hospital.stay Follow.up Reduction.of.PH Sympathectomy.Level /CONTRAST (Medical.issues)=Indicator(1) /CONTRAST (Sympathectomy.Level)=Indicator(1) /CONTRAST (Hospital.stay)=Indicator(1) /CONTRAST (Follow.up)=Indicator(1) /CONTRAST (Reduction.of.PH)=Indicator(1) /PRINT=GOODFIT CI(95) /CRITERIA=PIN(0.05) POUT(0.10) ITERATE(20) CUT(0.5).
Resources	Processor Time	00:00:00.03
	Elapsed Time	00:00:00.02

Case Processing Summary

Unweighted Cases[a]		N	Percent
Selected Cases	Included in Analysis	118	100.0
	Missing Cases	0	.0
	Total	118	100.0
Unselected Cases		0	.0
Total		118	100.0

a. If weight is in effect, see classification table for the total number of cases.

Dependent Variable Encoding

Original Value	Internal Value
No	0
Yes	1

Categorical Variables Codings

		Frequency	Parameter coding (1)	(2)	(3)	(4)	(5)	(6)
Hospital.stay	1.00	1	.000	.000	.000	.000	.000	.000
	2.00	3	1.000	.000	.000	.000	.000	.000
	3.00	68	.000	1.000	.000	.000	.000	.000
	4.00	30	.000	.000	1.000	.000	.000	.000
	5.00	8	.000	.000	.000	1.000	.000	.000
	6.00	7	.000	.000	.000	.000	1.000	.000
	9.00	1	.000	.000	.000	.000	.000	1.000
Reduction.of.PH	Complete (95-100%)	114	.000	.000				
	No change	2	1.000	.000				
	N/A	2	.000	1.000				
Sympathectomy.Level	T2-T4	51	.000					
	T2-T3	67	1.000					
Follow.up	Yes	116	.000					
	No	2	1.000					
MedicalIssue	No	109	.000					
	Yes	9	1.000					

Block 0: Beginning Block

Classification Table[a,b]

Observed			Predicted: CS No	Predicted: CS Yes	Percentage Correct
Step 0	CS	No	0	50	.0
		Yes	0	68	100.0
	Overall Percentage				57.6

a. Constant is included in the model.

b. The cut value is .500

Variables in the Equation

		B	S.E.	Wald	df	Sig.	Exp(B)
Step 0	Constant	.307	.186	2.724	1	.099	1.360

Variables not in the Equation[a]

			Score	df	Sig.
Step 0	Variables	MedicalIssue(1)	1.620	1	.203
		Hospital.stay	5.951	6	.429
		Hospital.stay(1)	2.263	1	.132
		Hospital.stay(2)	.467	1	.494
		Hospital.stay(3)	.015	1	.902
		Hospital.stay(4)	.204	1	.651
		Hospital.stay(5)	.665	1	.415
		Hospital.stay(6)	1.372	1	.242
		Follow.up(1)	2.767	1	.096
		Reduction.of.PH	2.829	2	.243
		Reduction.of.PH(1)	.048	1	.826
		Reduction.of.PH(2)	2.767	1	.096
		Sympathectomy.Level(1)	4.108	1	.043

a. Residual Chi-Squares are not computed because of redundancies.

Block 1: Method = Forward Stepwise (Conditional)

Omnibus Tests of Model Coefficients

		Chi-square	df	Sig.
Step 1	Step	4.114	1	.043
	Block	4.114	1	.043
	Model	4.114	1	.043

Model Summary

Step	-2 Log likelihood	Cox & Snell R Square	Nagelkerke R Square
1	156.712[a]	.034	.046

a. Estimation terminated at iteration number 3 because parameter estimates changed by less than .001.

Hosmer and Lemeshow Test

Step	Chi-square	df	Sig.
1	.000	0	.

Contingency Table for Hosmer and Lemeshow Test

		CS = No		CS = Yes		
		Observed	Expected	Observed	Expected	Total
Step 1	1	27	27.000	24	24.000	51
	2	23	23.000	44	44.000	67

Classification Table[a]

Observed			Predicted: CS No	Predicted: CS Yes	Percentage Correct
Step 1	CS	No	27	23	54.0
		Yes	24	44	64.7
	Overall Percentage				60.2

a. The cut value is .500

Variables in the Equation

		B	S.E.	Wald	df	Sig.	Exp(B)	95% C.I.for EXP(B) Lower	Upper
Step 1[a]	Sympathectomy.Level(1)	.766	.381	4.054	1	.044	2.152	1.021	4.538
	Constant	-.118	.281	.176	1	.675	.889		

a. Variable(s) entered on step 1: Sympathectomy.Level.

Model if Term Removed[a]

Variable		Model Log Likelihood	Change in -2 Log Likelihood	df	Sig. of the Change
Step 1	Sympathectomy.Level	-80.414	4.117	1	.042

a. Based on conditional parameter estimates

Variables not in the Equation[a]

			Score	df	Sig.
Step 1	Variables	MedicalIssue(1)	1.715	1	.190
		Hospital.stay	4.673	6	.586
		Hospital.stay(1)	1.642	1	.200
		Hospital.stay(2)	.208	1	.648
		Hospital.stay(3)	.042	1	.838
		Hospital.stay(4)	.059	1	.808
		Hospital.stay(5)	.057	1	.811
		Hospital.stay(6)	1.942	1	.163
		Follow.up(1)	1.850	1	.174
		Reduction.of.PH	1.896	2	.387
		Reduction.of.PH(1)	.035	1	.852
		Reduction.of.PH(2)	1.850	1	.174

a. Residual Chi-Squares are not computed because of redundancies.

```
 LOGISTIC REGRESSION VARIABLES Compensatory.sweating
  /METHOD=FSTEP(LR) Medical.issues Hospital.stay Follow.up Reduction.of.PH
Sympathectomy.Level
  /CONTRAST (Medical.issues)=Indicator(1)
  /CONTRAST (Sympathectomy.Level)=Indicator(1)
  /CONTRAST (Hospital.stay)=Indicator(1)
  /CONTRAST (Follow.up)=Indicator(1)
  /CONTRAST (Reduction.of.PH)=Indicator(1)
  /PRINT=GOODFIT CI(95)
  /CRITERIA=PIN(0.05) POUT(0.10) ITERATE(20) CUT(0.5).
```

Logistic Regression

Notes

Output Created		18-APR-2018 19:10:...
Comments		
Input	Data	C:\Users\rnordin.ADMIN\Desktop\2018\PUBLICATION 2018 ETS\ETS.Data (Complete).sav 18APRIL2018.sav
	Active Dataset	DataSet1
	Filter	<none>
	Weight	<none>
	Split File	<none>
	N of Rows in Working Data File	118
Missing Value Handling	Definition of Missing	User-defined missing values are treated as missing
Syntax		LOGISTIC REGRESSION VARIABLES Compensatory.sweating /METHOD=FSTEP(LR) Medical.issues Hospital.stay Follow.up Reduction.of.PH Sympathectomy.Level /CONTRAST (Medical.issues)=Indicator(1) /CONTRAST (Sympathectomy.Level)=Indicator(1) /CONTRAST (Hospital.stay)=Indicator(1) /CONTRAST (Follow.up)=Indicator(1) /CONTRAST (Reduction.of.PH)=Indicator(1) /PRINT=GOODFIT CI(95) /CRITERIA=PIN(0.05) POUT(0.10) ITERATE(20) CUT(0.5).
Resources	Processor Time	00:00:00.02
	Elapsed Time	00:00:00.01

Case Processing Summary

Unweighted Cases[a]		N	Percent
Selected Cases	Included in Analysis	118	100.0
	Missing Cases	0	.0
	Total	118	100.0
Unselected Cases		0	.0
Total		118	100.0

a. If weight is in effect, see classification table for the total number of cases.

Dependent Variable Encoding

Original Value	Internal Value
No	0
Yes	1

Categorical Variables Codings

		Frequency	Parameter coding					
			(1)	(2)	(3)	(4)	(5)	(6)
Hospital.stay	1.00	1	.000	.000	.000	.000	.000	.000
	2.00	3	1.000	.000	.000	.000	.000	.000
	3.00	68	.000	1.000	.000	.000	.000	.000
	4.00	30	.000	.000	1.000	.000	.000	.000
	5.00	8	.000	.000	.000	1.000	.000	.000
	6.00	7	.000	.000	.000	.000	1.000	.000
	9.00	1	.000	.000	.000	.000	.000	1.000
Reduction.of.PH	Complete (95-100%)	114	.000	.000				
	No change	2	1.000	.000				
	N/A	2	.000	1.000				
Sympathectomy.Level	T2-T4	51	.000					
	T2-T3	67	1.000					
Follow.up	Yes	116	.000					
	No	2	1.000					
MedicalIssue	No	109	.000					
	Yes	9	1.000					

Block 0: Beginning Block

Classification Table[a,b]

Observed			Predicted CS No	Predicted CS Yes	Predicted Percentage Correct
Step 0	CS	No	0	50	.0
		Yes	0	68	100.0
	Overall Percentage				57.6

a. Constant is included in the model.

b. The cut value is .500

Variables in the Equation

		B	S.E.	Wald	df	Sig.	Exp(B)
Step 0	Constant	.307	.186	2.724	1	.099	1.360

Variables not in the Equation[a]

			Score	df	Sig.
Step 0	Variables	MedicalIssue(1)	1.620	1	.203
		Hospital.stay	5.951	6	.429
		Hospital.stay(1)	2.263	1	.132
		Hospital.stay(2)	.467	1	.494
		Hospital.stay(3)	.015	1	.902
		Hospital.stay(4)	.204	1	.651
		Hospital.stay(5)	.665	1	.415
		Hospital.stay(6)	1.372	1	.242
		Follow.up(1)	2.767	1	.096
		Reduction.of.PH	2.829	2	.243
		Reduction.of.PH(1)	.048	1	.826
		Reduction.of.PH(2)	2.767	1	.096
		Sympathectomy.Level(1)	4.108	1	.043

a. Residual Chi-Squares are not computed because of redundancies.

Block 1: Method = Forward Stepwise (Likelihood Ratio)

Omnibus Tests of Model Coefficients

		Chi-square	df	Sig.
Step 1	Step	4.114	1	.043
	Block	4.114	1	.043
	Model	4.114	1	.043

Model Summary

Step	-2 Log likelihood	Cox & Snell R Square	Nagelkerke R Square
1	156.712[a]	.034	.046

a. Estimation terminated at iteration number 3 because parameter estimates changed by less than .001.

Hosmer and Lemeshow Test

Step	Chi-square	df	Sig.
1	.000	0	.

Contingency Table for Hosmer and Lemeshow Test

		CS = No		CS = Yes		
		Observed	Expected	Observed	Expected	Total
Step 1	1	27	27.000	24	24.000	51
	2	23	23.000	44	44.000	67

Classification Table[a]

Observed			Predicted CS No	Predicted CS Yes	Percentage Correct
Step 1	CS	No	27	23	54.0
		Yes	24	44	64.7
	Overall Percentage				60.2

a. The cut value is .500

Variables in the Equation

		B	S.E.	Wald	df	Sig.	Exp(B)	95% C.I.for EXP(B) Lower	Upper
Step 1[a]	Sympathectomy.Level(1)	.766	.381	4.054	1	.044	2.152	1.021	4.538
	Constant	-.118	.281	.176	1	.675	.889		

a. Variable(s) entered on step 1: Sympathectomy.Level.

Model if Term Removed

Variable		Model Log Likelihood	Change in -2 Log Likelihood	df	Sig. of the Change
Step 1	Sympathectomy.Level	-80.413	4.114	1	.043

Variables not in the Equation [a]

			Score	df	Sig.
Step 1	Variables	MedicalIssue(1)	1.715	1	.190
		Hospital.stay	4.673	6	.586
		Hospital.stay(1)	1.642	1	.200
		Hospital.stay(2)	.208	1	.648
		Hospital.stay(3)	.042	1	.838
		Hospital.stay(4)	.059	1	.808
		Hospital.stay(5)	.057	1	.811
		Hospital.stay(6)	1.942	1	.163
		Follow.up(1)	1.850	1	.174
		Reduction.of.PH	1.896	2	.387
		Reduction.of.PH(1)	.035	1	.852
		Reduction.of.PH(2)	1.850	1	.174

a. Residual Chi-Squares are not computed because of redundancies.

```
 LOGISTIC REGRESSION VARIABLES Compensatory.sweating
  /METHOD=FSTEP(WALD) Medical.issues Hospital.stay Follow.up Reduction.of.P
H Sympathectomy.Level
  /CONTRAST (Medical.issues)=Indicator(1)
  /CONTRAST (Sympathectomy.Level)=Indicator(1)
  /CONTRAST (Hospital.stay)=Indicator(1)
  /CONTRAST (Follow.up)=Indicator(1)
  /CONTRAST (Reduction.of.PH)=Indicator(1)
  /PRINT=GOODFIT CI(95)
  /CRITERIA=PIN(0.05) POUT(0.10) ITERATE(20) CUT(0.5).
```

Logistic Regression

Notes

Output Created		18-APR-2018 19:10:...
Comments		
Input	Data	C:\Users\rnordin.ADMIN\Desktop\2018\PUBLICATION 2018 ETS\ETS.Data (Complete).sav 18APRIL2018.sav
	Active Dataset	DataSet1
	Filter	<none>
	Weight	<none>
	Split File	<none>
	N of Rows in Working Data File	118
Missing Value Handling	Definition of Missing	User-defined missing values are treated as missing
Syntax		LOGISTIC REGRESSION VARIABLES Compensatory.sweating /METHOD=FSTEP (WALD) Medical.issues Hospital.stay Follow.up Reduction.of.PH Sympathectomy.Level /CONTRAST (Medical.issues)=Indicator(1) /CONTRAST (Sympathectomy.Level)=Indicator(1) /CONTRAST (Hospital.stay)=Indicator(1) /CONTRAST (Follow.up)=Indicator(1) /CONTRAST (Reduction.of.PH)=Indicator(1) /PRINT=GOODFIT CI (95) /CRITERIA=PIN(0.05) POUT(0.10) ITERATE (20) CUT(0.5).
Resources	Processor Time	00:00:00.02
	Elapsed Time	00:00:00.02

Case Processing Summary

Unweighted Cases[a]		N	Percent
Selected Cases	Included in Analysis	118	100.0
	Missing Cases	0	.0
	Total	118	100.0
Unselected Cases		0	.0
Total		118	100.0

a. If weight is in effect, see classification table for the total number of cases.

Dependent Variable Encoding

Original Value	Internal Value
No	0
Yes	1

Categorical Variables Codings

		Frequency	Parameter coding (1)	(2)	(3)	(4)	(5)	(6)
Hospital.stay	1.00	1	.000	.000	.000	.000	.000	.000
	2.00	3	1.000	.000	.000	.000	.000	.000
	3.00	68	.000	1.000	.000	.000	.000	.000
	4.00	30	.000	.000	1.000	.000	.000	.000
	5.00	8	.000	.000	.000	1.000	.000	.000
	6.00	7	.000	.000	.000	.000	1.000	.000
	9.00	1	.000	.000	.000	.000	.000	1.000
Reduction.of.PH	Complete (95-100%)	114	.000	.000				
	No change	2	1.000	.000				
	N/A	2	.000	1.000				
Sympathectomy.Level	T2-T4	51	.000					
	T2-T3	67	1.000					
Follow.up	Yes	116	.000					
	No	2	1.000					
MedicalIssue	No	109	.000					
	Yes	9	1.000					

Block 0: Beginning Block

Classification Table[a,b]

Observed			Predicted CS No	Predicted CS Yes	Percentage Correct
Step 0	CS	No	0	50	.0
		Yes	0	68	100.0
	Overall Percentage				57.6

a. Constant is included in the model.

b. The cut value is .500

Variables in the Equation

		B	S.E.	Wald	df	Sig.	Exp(B)
Step 0	Constant	.307	.186	2.724	1	.099	1.360

Variables not in the Equation[a]

			Score	df	Sig.
Step 0	Variables	MedicalIssue(1)	1.620	1	.203
		Hospital.stay	5.951	6	.429
		Hospital.stay(1)	2.263	1	.132
		Hospital.stay(2)	.467	1	.494
		Hospital.stay(3)	.015	1	.902
		Hospital.stay(4)	.204	1	.651
		Hospital.stay(5)	.665	1	.415
		Hospital.stay(6)	1.372	1	.242
		Follow.up(1)	2.767	1	.096
		Reduction.of.PH	2.829	2	.243
		Reduction.of.PH(1)	.048	1	.826
		Reduction.of.PH(2)	2.767	1	.096
		Sympathectomy.Level(1)	4.108	1	.043

a. Residual Chi-Squares are not computed because of redundancies.

Block 1: Method = Forward Stepwise (Wald)

Omnibus Tests of Model Coefficients

		Chi-square	df	Sig.
Step 1	Step	4.114	1	.043
	Block	4.114	1	.043
	Model	4.114	1	.043

Model Summary

Step	-2 Log likelihood	Cox & Snell R Square	Nagelkerke R Square
1	156.712[a]	.034	.046

a. Estimation terminated at iteration number 3 because parameter estimates changed by less than .001.

Hosmer and Lemeshow Test

Step	Chi-square	df	Sig.
1	.000	0	.

Contingency Table for Hosmer and Lemeshow Test

		CS = No		CS = Yes		
		Observed	Expected	Observed	Expected	Total
Step 1	1	27	27.000	24	24.000	51
	2	23	23.000	44	44.000	67

Classification Table[a]

Observed			Predicted CS No	Predicted CS Yes	Percentage Correct
Step 1	CS	No	27	23	54.0
		Yes	24	44	64.7
	Overall Percentage				60.2

a. The cut value is .500

Variables in the Equation

		B	S.E.	Wald	df	Sig.	Exp(B)	95% C.I.for EXP(B) Lower	95% C.I.for EXP(B) Upper
Step 1[a]	Sympathectomy.Level(1)	.766	.381	4.054	1	.044	2.152	1.021	4.538
	Constant	-.118	.281	.176	1	.675	.889		

a. Variable(s) entered on step 1: Sympathectomy.Level.

Variables not in the Equation[a]

			Score	df	Sig.
Step 1	Variables	MedicalIssue(1)	1.715	1	.190
		Hospital.stay	4.673	6	.586
		Hospital.stay(1)	1.642	1	.200
		Hospital.stay(2)	.208	1	.648
		Hospital.stay(3)	.042	1	.838
		Hospital.stay(4)	.059	1	.808
		Hospital.stay(5)	.057	1	.811
		Hospital.stay(6)	1.942	1	.163
		Follow.up(1)	1.850	1	.174
		Reduction.of.PH	1.896	2	.387
		Reduction.of.PH(1)	.035	1	.852
		Reduction.of.PH(2)	1.850	1	.174

a. Residual Chi-Squares are not computed because of redundancies.

```
 LOGISTIC REGRESSION VARIABLES Compensatory.sweating
  /METHOD=BSTEP(COND) Medical.issues Hospital.stay Follow.up Reduction.of.P
H Sympathectomy.Level
  /CONTRAST (Medical.issues)=Indicator(1)
  /CONTRAST (Sympathectomy.Level)=Indicator(1)
  /CONTRAST (Hospital.stay)=Indicator(1)
  /CONTRAST (Follow.up)=Indicator(1)
  /CONTRAST (Reduction.of.PH)=Indicator(1)
  /PRINT=GOODFIT CI(95)
  /CRITERIA=PIN(0.05) POUT(0.10) ITERATE(20) CUT(0.5).
```

Logistic Regression

Notes

Output Created		18-APR-2018 19:11:...
Comments		
Input	Data	C:\Users\rnordin.ADMIN\Desktop\2018\PUBLICATION 2018 ETS\ETS.Data (Complete).sav 18APRIL2018.sav
	Active Dataset	DataSet1
	Filter	<none>
	Weight	<none>
	Split File	<none>
	N of Rows in Working Data File	118
Missing Value Handling	Definition of Missing	User-defined missing values are treated as missing
Syntax		LOGISTIC REGRESSION VARIABLES Compensatory.sweating /METHOD=BSTEP (COND) Medical.issues Hospital.stay Follow.up Reduction.of.PH Sympathectomy.Level /CONTRAST (Medical.issues)=Indicator(1) /CONTRAST (Sympathectomy.Level)=Indicator(1) /CONTRAST (Hospital.stay)=Indicator(1) /CONTRAST (Follow.up)=Indicator(1) /CONTRAST (Reduction.of.PH)=Indicator(1) /PRINT=GOODFIT CI(95) /CRITERIA=PIN(0.05) POUT(0.10) ITERATE(20) CUT(0.5).
Resources	Processor Time	00:00:00.03
	Elapsed Time	00:00:00.04

Warnings

Due to redundancies, degrees of freedom have been reduced for one or more variables.

Case Processing Summary

Unweighted Cases[a]		N	Percent
Selected Cases	Included in Analysis	118	100.0
	Missing Cases	0	.0
	Total	118	100.0
Unselected Cases		0	.0
Total		118	100.0

a. If weight is in effect, see classification table for the total number of cases.

Dependent Variable Encoding

Original Value	Internal Value
No	0
Yes	1

Categorical Variables Codings

		Frequency	Parameter coding (1)	(2)	(3)	(4)	(5)	(6)
Hospital.stay	1.00	1	.000	.000	.000	.000	.000	.000
	2.00	3	1.000	.000	.000	.000	.000	.000
	3.00	68	.000	1.000	.000	.000	.000	.000
	4.00	30	.000	.000	1.000	.000	.000	.000
	5.00	8	.000	.000	.000	1.000	.000	.000
	6.00	7	.000	.000	.000	.000	1.000	.000
	9.00	1	.000	.000	.000	.000	.000	1.000
Reduction.of.PH	Complete (95-100%)	114	.000	.000				
	No change	2	1.000	.000				
	N/A	2	.000	1.000				
Sympathectomy.Level	T2-T4	51	.000					
	T2-T3	67	1.000					
Follow.up	Yes	116	.000					
	No	2	1.000					
MedicalIssue	No	109	.000					
	Yes	9	1.000					

Block 0: Beginning Block

Classification Table[a,b]

Observed			Predicted CS No	Predicted CS Yes	Percentage Correct
Step 0	CS	No	0	50	.0
		Yes	0	68	100.0
	Overall Percentage				57.6

a. Constant is included in the model.

b. The cut value is .500

Variables in the Equation

		B	S.E.	Wald	df	Sig.	Exp(B)
Step 0	Constant	.307	.186	2.724	1	.099	1.360

Variables not in the Equation[a]

			Score	df	Sig.
Step 0	Variables	MedicalIssue(1)	1.620	1	.203
		Hospital.stay	5.951	6	.429
		Hospital.stay(1)	2.263	1	.132
		Hospital.stay(2)	.467	1	.494
		Hospital.stay(3)	.015	1	.902
		Hospital.stay(4)	.204	1	.651
		Hospital.stay(5)	.665	1	.415
		Hospital.stay(6)	1.372	1	.242
		Follow.up(1)	2.767	1	.096
		Reduction.of.PH	2.829	2	.243
		Reduction.of.PH(1)	.048	1	.826
		Reduction.of.PH(2)	2.767	1	.096
		Sympathectomy.Level(1)	4.108	1	.043

a. Residual Chi-Squares are not computed because of redundancies.

Block 1: Method = Backward Stepwise (Conditional)

Omnibus Tests of Model Coefficients

		Chi-square	df	Sig.
Step 1	Step	14.995	10	.132
	Block	14.995	10	.132
	Model	14.995	10	.132
Step 2[a]	Step	-.011	1	.917
	Block	14.984	9	.091
	Model	14.984	9	.091
Step 3[a]	Step	-6.563	6	.363
	Block	8.421	3	.038
	Model	8.421	3	.038
Step 4[a]	Step	-1.690	1	.194
	Block	6.731	2	.035
	Model	6.731	2	.035
Step 5[a]	Step	-2.616	1	.106
	Block	4.114	1	.043
	Model	4.114	1	.043

a. A negative Chi-squares value indicates that the Chi-squares value has decreased from the previous step.

Model Summary

Step	-2 Log likelihood	Cox & Snell R Square	Nagelkerke R Square
1	145.832[a]	.119	.160
2	145.842[a]	.119	.160
3	152.406[a]	.069	.093
4	154.095[a]	.055	.075
5	156.712[b]	.034	.046

a. Estimation terminated at iteration number 20 because maximum iterations has been reached. Final solution cannot be found.

b. Estimation terminated at iteration number 3 because parameter estimates changed by less than .001.

Hosmer and Lemeshow Test

Step	Chi-square	df	Sig.
1	1.553	5	.907
2	.937	4	.919
3	.004	2	.998
4	.000	1	1.000
5	.000	0	.

Contingency Table for Hosmer and Lemeshow Test

		CS = No		CS = Yes		
		Observed	Expected	Observed	Expected	Total
Step 1	1	9	10.366	6	4.634	15
	2	5	4.357	3	3.643	8
	3	13	12.096	12	12.904	25
	4	8	7.728	11	11.272	19
	5	0	.380	1	.620	1
	6	13	13.073	25	24.927	38
	7	2	2.000	10	10.000	12
Step 2	1	9	10.348	6	4.652	15
	2	5	4.358	3	3.642	8
	3	13	12.111	12	12.889	25
	4	8	7.744	11	11.256	19
	5	13	13.438	26	25.562	39
	6	2	2.000	10	10.000	12
Step 3	1	2	2.000	0	.000	2
	2	24	23.840	21	21.160	45
	3	22	22.160	40	39.840	62
	4	2	2.000	7	7.000	9
Step 4	1	2	2.000	0	.000	2
	2	25	25.000	24	24.000	49
	3	23	23.000	44	44.000	67
Step 5	1	27	27.000	24	24.000	51
	2	23	23.000	44	44.000	67

Classification Table[a]

Observed			Predicted CS No	Predicted CS Yes	Percentage Correct
Step 1	CS	No	14	36	28.0
		Yes	9	59	86.8
	Overall Percentage				61.9
Step 2	CS	No	14	36	28.0
		Yes	9	59	86.8
	Overall Percentage				61.9
Step 3	CS	No	26	24	52.0
		Yes	21	47	69.1
	Overall Percentage				61.9
Step 4	CS	No	27	23	54.0
		Yes	24	44	64.7
	Overall Percentage				60.2
Step 5	CS	No	27	23	54.0
		Yes	24	44	64.7
	Overall Percentage				60.2

a. The cut value is .500

Variables in the Equation

		B	S.E.	Wald	df	Sig.	Exp(B)	95% C.I.for EXP(B)	
								Lower	Upper
Step 1[a]	MedicalIssue(1)	1.088	.861	1.594	1	.207	2.967	.549	16.048
	Hospital.stay			.532	6	.997			
	Hospital.stay(1)	41.825	46410.094	.000	1	.999	1.460E+18	.000	.
	Hospital.stay(2)	21.268	40192.104	.000	1	1.000	1.723E+9	.000	.
	Hospital.stay(3)	21.024	40192.104	.000	1	1.000	1.351E+9	.000	.
	Hospital.stay(4)	20.871	40192.104	.000	1	1.000	1.159E+9	.000	.
	Hospital.stay(5)	20.915	40192.104	.000	1	1.000	1.212E+9	.000	.
	Hospital.stay(6)	-.581	56840.831	.000	1	1.000	.560	.000	.
	Follow.up(1)	-21.150	28378.286	.000	1	.999	.000	.000	.
	Reduction.of.PH			.011	1	.917			
	Reduction.of.PH(1)	-.157	1.511	.011	1	.917	.855	.044	16.519
	Sympathectomy.Level(1)	.581	.417	1.937	1	.164	1.787	.789	4.049
	Constant	-21.203	40192.104	.000	1	1.000	.000		
Step 2[a]	MedicalIssue(1)	1.091	.861	1.608	1	.205	2.978	.551	16.089
	Hospital.stay			.549	6	.997			
	Hospital.stay(1)	41.825	46408.371	.000	1	.999	1.460E+18	.000	.
	Hospital.stay(2)	21.265	40190.115	.000	1	1.000	1.719E+9	.000	.
	Hospital.stay(3)	21.024	40190.115	.000	1	1.000	1.350E+9	.000	.
	Hospital.stay(4)	20.851	40190.115	.000	1	1.000	1.137E+9	.000	.
	Hospital.stay(5)	20.915	40190.115	.000	1	1.000	1.212E+9	.000	.
	Hospital.stay(6)	-.581	56839.425	.000	1	1.000	.559	.000	.
	Follow.up(1)	-21.149	28378.994	.000	1	.999	.000	.000	.
	Sympathectomy.Level(1)	.581	.417	1.937	1	.164	1.787	.789	4.049
	Constant	-21.203	40190.115	.000	1	1.000	.000		
Step 3[a]	MedicalIssue(1)	1.014	.835	1.474	1	.225	2.757	.536	14.170
	Follow.up(1)	-21.084	28420.722	.000	1	.999	.000	.000	.
	Sympathectomy.Level(1)	.706	.388	3.312	1	.069	2.025	.947	4.331
	Constant	-.119	.294	.165	1	.685	.888		
Step 4[a]	Follow.up(1)	-21.162	28420.722	.000	1	.999	.000	.000	.
	Sympathectomy.Level(1)	.690	.385	3.215	1	.073	1.993	.938	4.234
	Constant	-.041	.286	.020	1	.886	.960		
Step 5[a]	Sympathectomy.Level(1)	.766	.381	4.054	1	.044	2.152	1.021	4.538
	Constant	-.118	.281	.176	1	.675	.889		

a. Variable(s) entered on step 1: MedicalIssue, Hospital.stay, Follow.up, Reduction.of.PH, Sympathectomy.Level.

Model if Term Removed[a]

Variable		Model Log Likelihood	Change in -2 Log Likelihood	df	Sig. of the Change
Step 1	MedicalIssue	-73.833	1.835	1	.176
	Hospital.stay	-76.207	6.583	6	.361
	Follow.up	-74.250	2.668	1	.102
	Reduction.of.PH	-72.921	.011	1	.917
	Sympathectomy.Level	-73.890	1.948	1	.163
Step 2	MedicalIssue	-73.847	1.852	1	.174
	Hospital.stay	-76.221	6.599	6	.359
	Follow.up	-74.253	2.664	1	.103
	Sympathectomy.Level	-73.896	1.949	1	.163
Step 3	MedicalIssue	-77.050	1.694	1	.193
	Follow.up	-77.473	2.541	1	.111
	Sympathectomy.Level	-77.881	3.356	1	.067
Step 4	Follow.up	-78.394	2.692	1	.101
	Sympathectomy.Level	-78.673	3.251	1	.071
Step 5	Sympathectomy.Level	-80.414	4.117	1	.042

a. Based on conditional parameter estimates

Variables not in the Equation

			Score	df	Sig.
Step 2[a]	Variables	Reduction.of.PH	.011	1	.917
		Reduction.of.PH(1)	.011	1	.917
	Overall Statistics		.011	1	.917
Step 3[b]	Variables	Hospital.stay	4.940	6	.552
		Hospital.stay(1)	1.751	1	.186
		Hospital.stay(2)	.448	1	.503
		Hospital.stay(3)	.147	1	.701
		Hospital.stay(4)	.155	1	.694
		Hospital.stay(5)	.057	1	.811
		Hospital.stay(6)	1.826	1	.177
		Reduction.of.PH	.027	1	.869
		Reduction.of.PH(1)	.027	1	.869
	Overall Statistics		4.950	7	.666
Step 4[c]	Variables	MedicalIssue(1)	1.575	1	.210
		Hospital.stay	4.843	6	.564
		Hospital.stay(1)	1.642	1	.200
		Hospital.stay(2)	.209	1	.648
		Hospital.stay(3)	.009	1	.926
		Hospital.stay(4)	.099	1	.753

Variables not in the Equation

			Score	df	Sig.
		Hospital.stay(5)	.122	1	.726
		Hospital.stay(6)	1.942	1	.163
		Reduction.of.PH	.046	1	.830
		Reduction.of.PH(1)	.046	1	.830
	Overall Statistics		6.577	8	.583
Step 5[d]	Variables	MedicalIssue(1)	1.715	1	.190
		Hospital.stay	4.673	6	.586
		Hospital.stay(1)	1.642	1	.200
		Hospital.stay(2)	.208	1	.648
		Hospital.stay(3)	.042	1	.838
		Hospital.stay(4)	.059	1	.808
		Hospital.stay(5)	.057	1	.811
		Hospital.stay(6)	1.942	1	.163
		Follow.up(1)	1.850	1	.174
		Reduction.of.PH	.035	1	.852
		Reduction.of.PH(1)	.035	1	.852
	Overall Statistics		8.426	9	.492

a. Variable(s) removed on step 2: Reduction.of.PH.

b. Variable(s) removed on step 3: Hospital.stay.

c. Variable(s) removed on step 4: MedicalIssue.

d. Variable(s) removed on step 5: Follow.up.

```
LOGISTIC REGRESSION VARIABLES Compensatory.sweating
  /METHOD=BSTEP(LR) Medical.issues Hospital.stay Follow.up Reduction.of.PH
Sympathectomy.Level
  /CONTRAST (Medical.issues)=Indicator(1)
  /CONTRAST (Sympathectomy.Level)=Indicator(1)
  /CONTRAST (Hospital.stay)=Indicator(1)
  /CONTRAST (Follow.up)=Indicator(1)
  /CONTRAST (Reduction.of.PH)=Indicator(1)
  /PRINT=GOODFIT CI(95)
  /CRITERIA=PIN(0.05) POUT(0.10) ITERATE(20) CUT(0.5).
```

Logistic Regression

Notes

Output Created		18-APR-2018 19:12:...
Comments		
Input	Data	C:\Users\rnordin.ADMIN\Desktop\2018\PUBLICATION 2018 ETS\ETS.Data (Complete).sav 18APRIL2018.sav
	Active Dataset	DataSet1
	Filter	<none>
	Weight	<none>
	Split File	<none>
	N of Rows in Working Data File	118
Missing Value Handling	Definition of Missing	User-defined missing values are treated as missing
Syntax		LOGISTIC REGRESSION VARIABLES Compensatory.sweating /METHOD=BSTEP(LR) Medical.issues Hospital.stay Follow.up Reduction.of.PH Sympathectomy.Level /CONTRAST (Medical.issues)=Indicator(1) /CONTRAST (Sympathectomy.Level)=Indicator(1) /CONTRAST (Hospital.stay)=Indicator(1) /CONTRAST (Follow.up)=Indicator(1) /CONTRAST (Reduction.of.PH)=Indicator(1) /PRINT=GOODFIT CI(95) /CRITERIA=PIN(0.05) POUT(0.10) ITERATE(20) CUT(0.5).
Resources	Processor Time	00:00:00.03
	Elapsed Time	00:00:00.04

Warnings

Due to redundancies, degrees of freedom have been reduced for one or more variables.

Case Processing Summary

Unweighted Cases[a]		N	Percent
Selected Cases	Included in Analysis	118	100.0
	Missing Cases	0	.0
	Total	118	100.0
Unselected Cases		0	.0
Total		118	100.0

a. If weight is in effect, see classification table for the total number of cases.

Dependent Variable Encoding

Original Value	Internal Value
No	0
Yes	1

Categorical Variables Codings

		Frequency	Parameter coding (1)	(2)	(3)	(4)	(5)	(6)
Hospital.stay	1.00	1	.000	.000	.000	.000	.000	.000
	2.00	3	1.000	.000	.000	.000	.000	.000
	3.00	68	.000	1.000	.000	.000	.000	.000
	4.00	30	.000	.000	1.000	.000	.000	.000
	5.00	8	.000	.000	.000	1.000	.000	.000
	6.00	7	.000	.000	.000	.000	1.000	.000
	9.00	1	.000	.000	.000	.000	.000	1.000
Reduction.of.PH	Complete (95-100%)	114	.000	.000				
	No change	2	1.000	.000				
	N/A	2	.000	1.000				
Sympathectomy.Level	T2-T4	51	.000					
	T2-T3	67	1.000					
Follow.up	Yes	116	.000					
	No	2	1.000					
MedicalIssue	No	109	.000					
	Yes	9	1.000					

Block 0: Beginning Block

Classification Table[a,b]

Observed			Predicted: CS No	Predicted: CS Yes	Predicted: Percentage Correct
Step 0	CS	No	0	50	.0
		Yes	0	68	100.0
	Overall Percentage				57.6

a. Constant is included in the model.

b. The cut value is .500

Variables in the Equation

		B	S.E.	Wald	df	Sig.	Exp(B)
Step 0	Constant	.307	.186	2.724	1	.099	1.360

Variables not in the Equation[a]

			Score	df	Sig.
Step 0	Variables	MedicalIssue(1)	1.620	1	.203
		Hospital.stay	5.951	6	.429
		Hospital.stay(1)	2.263	1	.132
		Hospital.stay(2)	.467	1	.494
		Hospital.stay(3)	.015	1	.902
		Hospital.stay(4)	.204	1	.651
		Hospital.stay(5)	.665	1	.415
		Hospital.stay(6)	1.372	1	.242
		Follow.up(1)	2.767	1	.096
		Reduction.of.PH	2.829	2	.243
		Reduction.of.PH(1)	.048	1	.826
		Reduction.of.PH(2)	2.767	1	.096
		Sympathectomy.Level(1)	4.108	1	.043

a. Residual Chi-Squares are not computed because of redundancies.

Block 1: Method = Backward Stepwise (Likelihood Ratio)

Omnibus Tests of Model Coefficients

		Chi-square	df	Sig.
Step 1	Step	14.995	10	.132
	Block	14.995	10	.132
	Model	14.995	10	.132
Step 2[a]	Step	-.011	1	.917
	Block	14.984	9	.091
	Model	14.984	9	.091
Step 3[a]	Step	-6.563	6	.363
	Block	8.421	3	.038
	Model	8.421	8	.394
Step 4[a]	Step	-1.690	1	.194
	Block	6.731	2	.035
	Model	6.731	2	.035
Step 5[a]	Step	-2.616	1	.106
	Block	4.114	1	.043
	Model	4.114	1	.043

a. A negative Chi-squares value indicates that the Chi-squares value has decreased from the previous step.

Model Summary

Step	-2 Log likelihood	Cox & Snell R Square	Nagelkerke R Square
1	145.832[a]	.119	.160
2	145.842[a]	.119	.160
3	152.406[a]	.069	.093
4	154.095[a]	.055	.075
5	156.712[b]	.034	.046

a. Estimation terminated at iteration number 20 because maximum iterations has been reached. Final solution cannot be found.

b. Estimation terminated at iteration number 3 because parameter estimates changed by less than .001.

Hosmer and Lemeshow Test

Step	Chi-square	df	Sig.
1	1.553	5	.907
2	.937	4	.919
3	.004	2	.998
4	.000	1	1.000
5	.000	0	.

Contingency Table for Hosmer and Lemeshow Test

		CS = No		CS = Yes		
		Observed	Expected	Observed	Expected	Total
Step 1	1	9	10.366	6	4.634	15
	2	5	4.357	3	3.643	8
	3	13	12.096	12	12.904	25
	4	8	7.728	11	11.272	19
	5	0	.380	1	.620	1
	6	13	13.073	25	24.927	38
	7	2	2.000	10	10.000	12
Step 2	1	9	10.348	6	4.652	15
	2	5	4.358	3	3.642	8
	3	13	12.111	12	12.889	25
	4	8	7.744	11	11.256	19
	5	13	13.438	26	25.562	39
	6	2	2.000	10	10.000	12
Step 3	1	2	2.000	0	.000	2
	2	24	23.840	21	21.160	45
	3	22	22.160	40	39.840	62
	4	2	2.000	7	7.000	9
Step 4	1	2	2.000	0	.000	2
	2	25	25.000	24	24.000	49
	3	23	23.000	44	44.000	67
Step 5	1	27	27.000	24	24.000	51
	2	23	23.000	44	44.000	67

Classification Table[a]

Observed			Predicted CS No	Predicted CS Yes	Percentage Correct
Step 1	CS	No	14	36	28.0
		Yes	9	59	86.8
	Overall Percentage				61.9
Step 2	CS	No	14	36	28.0
		Yes	9	59	86.8
	Overall Percentage				61.9
Step 3	CS	No	26	24	52.0
		Yes	21	47	69.1
	Overall Percentage				61.9
Step 4	CS	No	27	23	54.0
		Yes	24	44	64.7
	Overall Percentage				60.2
Step 5	CS	No	27	23	54.0
		Yes	24	44	64.7
	Overall Percentage				60.2

a. The cut value is .500

Variables in the Equation

		B	S.E.	Wald	df	Sig.	Exp(B)	95% C.I.for EXP(B)	
								Lower	Upper
Step 1[a]	MedicalIssue(1)	1.088	.861	1.594	1	.207	2.967	.549	16.048
	Hospital.stay			.532	6	.997			
	Hospital.stay(1)	41.825	46410.094	.000	1	.999	1.460E+18	.000	.
	Hospital.stay(2)	21.268	40192.104	.000	1	1.000	1.723E+9	.000	.
	Hospital.stay(3)	21.024	40192.104	.000	1	1.000	1.351E+9	.000	.
	Hospital.stay(4)	20.871	40192.104	.000	1	1.000	1.159E+9	.000	.
	Hospital.stay(5)	20.915	40192.104	.000	1	1.000	1.212E+9	.000	.
	Hospital.stay(6)	-.581	56840.831	.000	1	1.000	.560	.000	.
	Follow.up(1)	-21.150	28378.286	.000	1	.999	.000	.000	.
	Reduction.of.PH			.011	1	.917			
	Reduction.of.PH(1)	-.157	1.511	.011	1	.917	.855	.044	16.519
	Sympathectomy.Level(1)	.581	.417	1.937	1	.164	1.787	.789	4.049
	Constant	-21.203	40192.104	.000	1	1.000	.000		
Step 2[a]	MedicalIssue(1)	1.091	.861	1.608	1	.205	2.978	.551	16.089
	Hospital.stay			.549	6	.997			
	Hospital.stay(1)	41.825	46408.371	.000	1	.999	1.460E+18	.000	.
	Hospital.stay(2)	21.265	40190.115	.000	1	1.000	1.719E+9	.000	.
	Hospital.stay(3)	21.024	40190.115	.000	1	1.000	1.350E+9	.000	.
	Hospital.stay(4)	20.851	40190.115	.000	1	1.000	1.137E+9	.000	.
	Hospital.stay(5)	20.915	40190.115	.000	1	1.000	1.212E+9	.000	.
	Hospital.stay(6)	-.581	56839.425	.000	1	1.000	.559	.000	.
	Follow.up(1)	-21.149	28378.994	.000	1	.999	.000	.000	.
	Sympathectomy.Level(1)	.581	.417	1.937	1	.164	1.787	.789	4.049
	Constant	-21.203	40190.115	.000	1	1.000	.000		
Step 3[a]	MedicalIssue(1)	1.014	.835	1.474	1	.225	2.757	.536	14.170
	Follow.up(1)	-21.084	28420.722	.000	1	.999	.000	.000	.
	Sympathectomy.Level(1)	.706	.388	3.312	1	.069	2.025	.947	4.331
	Constant	-.119	.294	.165	1	.685	.888		
Step 4[a]	Follow.up(1)	-21.162	28420.722	.000	1	.999	.000	.000	.
	Sympathectomy.Level(1)	.690	.385	3.215	1	.073	1.993	.938	4.234
	Constant	-.041	.286	.020	1	.886	.960		
Step 5[a]	Sympathectomy.Level(1)	.766	.381	4.054	1	.044	2.152	1.021	4.538
	Constant	-.118	.281	.176	1	.675	.889		

a. Variable(s) entered on step 1: MedicalIssue, Hospital.stay, Follow.up, Reduction.of.PH, Sympathectomy.Level.

Model if Term Removed

Variable		Model Log Likelihood	Change in -2 Log Likelihood	df	Sig. of the Change
Step 1	MedicalIssue	-73.830	1.829	1	.176
	Hospital.stay	-76.189	6.547	6	.365
	Follow.up	-74.196	2.560	1	.110
	Reduction.of.PH	-72.921	.011	1	.917
	Sympathectomy.Level	-73.889	1.947	1	.163
Step 2	MedicalIssue	-73.844	1.845	1	.174
	Hospital.stay	-76.203	6.563	6	.363
	Follow.up	-74.200	2.557	1	.110
	Sympathectomy.Level	-73.895	1.947	1	.163
Step 3	MedicalIssue	-77.048	1.690	1	.194
	Follow.up	-77.437	2.468	1	.116
	Sympathectomy.Level	-77.879	3.353	1	.067
Step 4	Follow.up	-78.356	2.616	1	.106
	Sympathectomy.Level	-78.672	3.249	1	.071
Step 5	Sympathectomy.Level	-80.413	4.114	1	.043

Variables not in the Equation

			Score	df	Sig.
Step 2[a]	Variables	Reduction.of.PH	.011	1	.917
		Reduction.of.PH(1)	.011	1	.917
	Overall Statistics		.011	1	.917
Step 3[b]	Variables	Hospital.stay	4.940	6	.552
		Hospital.stay(1)	1.751	1	.186
		Hospital.stay(2)	.448	1	.503
		Hospital.stay(3)	.147	1	.701
		Hospital.stay(4)	.155	1	.694
		Hospital.stay(5)	.057	1	.811
		Hospital.stay(6)	1.826	1	.177
		Reduction.of.PH	.027	1	.869
		Reduction.of.PH(1)	.027	1	.869
	Overall Statistics		4.950	7	.666
Step 4[c]	Variables	MedicalIssue(1)	1.575	1	.210
		Hospital.stay	4.843	6	.564
		Hospital.stay(1)	1.642	1	.200
		Hospital.stay(2)	.209	1	.648
		Hospital.stay(3)	.009	1	.926
		Hospital.stay(4)	.099	1	.753
		Hospital.stay(5)	.122	1	.726
		Hospital.stay(6)	1.942	1	.163

Variables not in the Equation

			Score	df	Sig.
		Reduction.of.PH	.046	1	.830
		Reduction.of.PH(1)	.046	1	.830
	Overall Statistics		6.577	8	.583
Step 5[d]	Variables	MedicalIssue(1)	1.715	1	.190
		Hospital.stay	4.673	6	.586
		Hospital.stay(1)	1.642	1	.200
		Hospital.stay(2)	.208	1	.648
		Hospital.stay(3)	.042	1	.838
		Hospital.stay(4)	.059	1	.808
		Hospital.stay(5)	.057	1	.811
		Hospital.stay(6)	1.942	1	.163
		Follow.up(1)	1.850	1	.174
		Reduction.of.PH	.035	1	.852
		Reduction.of.PH(1)	.035	1	.852
	Overall Statistics		8.426	9	.492

a. Variable(s) removed on step 2: Reduction.of.PH.

b. Variable(s) removed on step 3: Hospital.stay.

c. Variable(s) removed on step 4: MedicalIssue.

d. Variable(s) removed on step 5: Follow.up.

```
 LOGISTIC REGRESSION VARIABLES Compensatory.sweating
  /METHOD=BSTEP(WALD) Medical.issues Hospital.stay Follow.up Reduction.of.P
H Sympathectomy.Level
  /CONTRAST (Medical.issues)=Indicator(1)
  /CONTRAST (Sympathectomy.Level)=Indicator(1)
  /CONTRAST (Hospital.stay)=Indicator(1)
  /CONTRAST (Follow.up)=Indicator(1)
  /CONTRAST (Reduction.of.PH)=Indicator(1)
  /PRINT=GOODFIT CI(95)
  /CRITERIA=PIN(0.05) POUT(0.10) ITERATE(20) CUT(0.5).
```

Logistic Regression

Notes

Output Created		18-APR-2018 19:14:...
Comments		
Input	Data	C:\Users\rnordin.ADMIN\Desktop\2018\PUBLICATION 2018 ETS\ETS.Data (Complete).sav 18APRIL2018.sav
	Active Dataset	DataSet1
	Filter	<none>
	Weight	<none>
	Split File	<none>
	N of Rows in Working Data File	118
Missing Value Handling	Definition of Missing	User-defined missing values are treated as missing
Syntax		LOGISTIC REGRESSION VARIABLES Compensatory.sweating /METHOD=BSTEP (WALD) Medical.issues Hospital.stay Follow.up Reduction.of.PH Sympathectomy.Level /CONTRAST (Medical.issues)=Indicator(1) /CONTRAST (Sympathectomy.Level)=Indicator(1) /CONTRAST (Hospital.stay)=Indicator(1) /CONTRAST (Follow.up)=Indicator(1) /CONTRAST (Reduction.of.PH)=Indicator(1) /PRINT=GOODFIT CI(95) /CRITERIA=PIN(0.05) POUT(0.10) ITERATE(20) CUT(0.5).
Resources	Processor Time	00:00:00.02
	Elapsed Time	00:00:00.02

Warnings

Due to redundancies, degrees of freedom have been reduced for one or more variables.

Case Processing Summary

Unweighted Cases[a]		N	Percent
Selected Cases	Included in Analysis	118	100.0
	Missing Cases	0	.0
	Total	118	100.0
Unselected Cases		0	.0
Total		118	100.0

a. If weight is in effect, see classification table for the total number of cases.

Dependent Variable Encoding

Original Value	Internal Value
No	0
Yes	1

Categorical Variables Codings

		Frequency	Parameter coding					
			(1)	(2)	(3)	(4)	(5)	(6)
Hospital.stay	1.00	1	.000	.000	.000	.000	.000	.000
	2.00	3	1.000	.000	.000	.000	.000	.000
	3.00	68	.000	1.000	.000	.000	.000	.000
	4.00	30	.000	.000	1.000	.000	.000	.000
	5.00	8	.000	.000	.000	1.000	.000	.000
	6.00	7	.000	.000	.000	.000	1.000	.000
	9.00	1	.000	.000	.000	.000	.000	1.000
Reduction.of.PH	Complete (95-100%)	114	.000	.000				
	No change	2	1.000	.000				
	N/A	2	.000	1.000				
Sympathectomy.Level	T2-T4	51	.000					
	T2-T3	67	1.000					
Follow.up	Yes	116	.000					
	No	2	1.000					
MedicalIssue	No	109	.000					
	Yes	9	1.000					

Block 0: Beginning Block

Classification Table[a,b]

Observed			Predicted CS No	Predicted CS Yes	Percentage Correct
Step 0	CS	No	0	50	.0
		Yes	0	68	100.0
	Overall Percentage				57.6

a. Constant is included in the model.

b. The cut value is .500

Variables in the Equation

		B	S.E.	Wald	df	Sig.	Exp(B)
Step 0	Constant	.307	.186	2.724	1	.099	1.360

Variables not in the Equation[a]

			Score	df	Sig.
Step 0	Variables	MedicalIssue(1)	1.620	1	.203
		Hospital.stay	5.951	6	.429
		Hospital.stay(1)	2.263	1	.132
		Hospital.stay(2)	.467	1	.494
		Hospital.stay(3)	.015	1	.902
		Hospital.stay(4)	.204	1	.651
		Hospital.stay(5)	.665	1	.415
		Hospital.stay(6)	1.372	1	.242
		Follow.up(1)	2.767	1	.096
		Reduction.of.PH	2.829	2	.243
		Reduction.of.PH(1)	.048	1	.826
		Reduction.of.PH(2)	2.767	1	.096
		Sympathectomy.Level(1)	4.108	1	.043

a. Residual Chi-Squares are not computed because of redundancies.

Block 1: Method = Backward Stepwise (Wald)

Omnibus Tests of Model Coefficients

		Chi-square	df	Sig.
Step 1	Step	14.995	10	.132
	Block	14.995	10	.132
	Model	14.995	10	.132
Step 2[a]	Step	-2.560	1	.110
	Block	12.435	9	.190
	Model	12.435	9	.190
Step 3[a]	Step	-6.464	6	.373
	Block	5.971	3	.113
	Model	5.971	3	.113
Step 4[a]	Step	-.018	1	.892
	Block	5.953	2	.051
	Model	5.953	2	.051
Step 5[a]	Step	-1.838	1	.175
	Block	4.114	1	.043
	Model	4.114	1	.043

a. A negative Chi-squares value indicates that the Chi-squares value has decreased from the previous step.

Model Summary

Step	-2 Log likelihood	Cox & Snell R Square	Nagelkerke R Square
1	145.832[a]	.119	.160
2	148.391[a]	.100	.134
3	154.855[b]	.049	.066
4	154.874[b]	.049	.066
5	156.712[c]	.034	.046

a. Estimation terminated at iteration number 20 because maximum iterations has been reached. Final solution cannot be found.

b. Estimation terminated at iteration number 4 because parameter estimates changed by less than .001.

c. Estimation terminated at iteration number 3 because parameter estimates changed by less than .001.

Hosmer and Lemeshow Test

Step	Chi-square	df	Sig.
1	1.553	5	.907
2	.812	5	.976
3	1.361	3	.715
4	.006	1	.940
5	.000	0	.

Contingency Table for Hosmer and Lemeshow Test

		CS = No		CS = Yes		
		Observed	Expected	Observed	Expected	Total
Step 1	1	9	10.366	6	4.634	15
	2	5	4.357	3	3.643	8
	3	13	12.096	12	12.904	25
	4	8	7.728	11	11.272	19
	5	0	.380	1	.620	1
	6	13	13.073	25	24.927	38
	7	2	2.000	10	10.000	12
Step 2	1	9	9.602	6	5.398	15
	2	4	4.000	3	3.000	7
	3	14	13.180	12	12.820	26
	4	8	7.822	11	11.178	19
	5	0	.374	1	.626	1
	6	13	13.022	25	24.978	38
	7	2	2.000	10	10.000	12
Step 3	1	1	.596	0	.404	1
	2	25	25.215	21	20.785	46
	3	0	.404	1	.596	1
	4	22	21.785	39	39.215	61
	5	2	2.000	7	7.000	9
Step 4	1	26	25.811	21	21.189	47
	2	22	22.189	40	39.811	62
	3	2	2.000	7	7.000	9
Step 5	1	27	27.000	24	24.000	51
	2	23	23.000	44	44.000	67

Classification Table[a]

Observed			Predicted CS No	Predicted CS Yes	Percentage Correct
Step 1	CS	No	14	36	28.0
		Yes	9	59	86.8
	Overall Percentage				61.9
Step 2	CS	No	27	23	54.0
		Yes	21	47	69.1
	Overall Percentage				62.7
Step 3	CS	No	26	24	52.0
		Yes	21	47	69.1
	Overall Percentage				61.9
Step 4	CS	No	26	24	52.0
		Yes	21	47	69.1
	Overall Percentage				61.9
Step 5	CS	No	27	23	54.0
		Yes	24	44	64.7
	Overall Percentage				60.2

a. The cut value is .500

Variables in the Equation

		B	S.E.	Wald	df	Sig.	Exp(B)	95% C.I.for EXP(B)	
								Lower	Upper
Step 1[a]	MedicalIssue(1)	1.088	.861	1.594	1	.207	2.967	.549	16.048
	Hospital.stay			.532	6	.997			
	Hospital.stay(1)	41.825	46410.094	.000	1	.999	1.460E+18	.000	.
	Hospital.stay(2)	21.268	40192.104	.000	1	1.000	1.723E+9	.000	.
	Hospital.stay(3)	21.024	40192.104	.000	1	1.000	1.351E+9	.000	.
	Hospital.stay(4)	20.871	40192.104	.000	1	1.000	1.159E+9	.000	.
	Hospital.stay(5)	20.915	40192.104	.000	1	1.000	1.212E+9	.000	.
	Hospital.stay(6)	-.581	56840.831	.000	1	1.000	.560	.000	.
	Follow.up(1)	-21.150	28378.286	.000	1	.999	.000	.000	.
	Reduction.of.PH			.011	1	.917			
	Reduction.of.PH(1)	-.157	1.511	.011	1	.917	.855	.044	16.519
	Sympathectomy.Level(1)	.581	.417	1.937	1	.164	1.787	.789	4.049
	Constant	-21.203	40192.104	.000	1	1.000	.000		
Step 2[a]	MedicalIssue(1)	1.154	.863	1.787	1	.181	3.171	.584	17.229
	Hospital.stay			.522	6	.998			
	Hospital.stay(1)	41.727	46411.767	.000	1	.999	1.323E+18	.000	.
	Hospital.stay(2)	21.175	40194.036	.000	1	1.000	1.571E+9	.000	.
	Hospital.stay(3)	20.892	40194.036	.000	1	1.000	1.184E+9	.000	.
	Hospital.stay(4)	20.823	40194.036	.000	1	1.000	1.105E+9	.000	.
	Hospital.stay(5)	20.915	40194.036	.000	1	1.000	1.212E+9	.000	.
	Hospital.stay(6)	-.679	56842.197	.000	1	1.000	.507	.000	.
	Reduction.of.PH			.008	1	.929			
	Reduction.of.PH(1)	-.136	1.516	.008	1	.929	.873	.045	17.032
	Sympathectomy.Level(1)	.679	.411	2.725	1	.099	1.972	.881	4.416
	Constant	-21.203	40194.036	.000	1	1.000	.000		
Step 3[a]	MedicalIssue(1)	1.054	.837	1.584	1	.208	2.869	.556	14.803
	Reduction.of.PH			.018	1	.892			
	Reduction.of.PH(1)	-.197	1.455	.018	1	.892	.821	.047	14.216
	Sympathectomy.Level(1)	.781	.384	4.134	1	.042	2.184	1.029	4.636
	Constant	-.193	.290	.443	1	.506	.824		
Step 4[a]	MedicalIssue(1)	1.058	.837	1.597	1	.206	2.879	.558	14.845
	Sympathectomy.Level(1)	.782	.384	4.144	1	.042	2.186	1.030	4.639
	Constant	-.197	.289	.467	1	.494	.821		
Step 5[a]	Sympathectomy.Level(1)	.766	.381	4.054	1	.044	2.152	1.021	4.538
	Constant	-.118	.281	.176	1	.675	.889		

a. Variable(s) entered on step 1: MedicalIssue, Hospital.stay, Follow.up, Reduction.of.PH, Sympathectomy.Level.

Variables not in the Equation

			Score	df	Sig.
Step 2[a]	Variables	Follow.up(1)	1.802	1	.179
	Overall Statistics		1.802	1	.179
Step 3[b]	Variables	Hospital.stay	310.297	6	.000
		Hospital.stay(1)	.000	1	1.000
		Hospital.stay(2)	.000	1	1.000
		Hospital.stay(3)	.000	1	1.000
		Hospital.stay(4)	.000	1	1.000
		Hospital.stay(5)	.000	1	1.000
		Hospital.stay(6)	.000	1	1.000
		Follow.up(1)	1.720	1	.190
	Overall Statistics		1.811	7	.970
Step 4[c]	Variables	Hospital.stay	4.855	6	.563
		Hospital.stay(1)	1.755	1	.185
		Hospital.stay(2)	.459	1	.498
		Hospital.stay(3)	.253	1	.615
		Hospital.stay(4)	.108	1	.743
		Hospital.stay(5)	.016	1	.898
		Hospital.stay(6)	1.823	1	.177
		Follow.up(1)	1.713	1	.191
		Reduction.of.PH	.018	1	.892
		Reduction.of.PH(1)	.018	1	.892
	Overall Statistics		6.661	8	.574
Step 5[d]	Variables	MedicalIssue(1)	1.715	1	.190
		Hospital.stay	4.673	6	.586
		Hospital.stay(1)	1.642	1	.200
		Hospital.stay(2)	.208	1	.648
		Hospital.stay(3)	.042	1	.838
		Hospital.stay(4)	.059	1	.808
		Hospital.stay(5)	.057	1	.811
		Hospital.stay(6)	1.942	1	.163
		Follow.up(1)	1.850	1	.174
		Reduction.of.PH	.035	1	.852
		Reduction.of.PH(1)	.035	1	.852
	Overall Statistics		8.426	9	.492

a. Variable(s) removed on step 2: Follow.up.

b. Variable(s) removed on step 3: Hospital.stay.

c. Variable(s) removed on step 4: Reduction.of.PH.

d. Variable(s) removed on step 5: MedicalIssue.

```
LOGISTIC REGRESSION VARIABLES Compensatory.sweating
 /METHOD=ENTER Hospital.stay
 /CONTRAST (Hospital.stay)=Indicator(1)
 /PRINT=GOODFIT CI(95)
 /CRITERIA=PIN(0.05) POUT(0.10) ITERATE(20) CUT(0.5).
```

Logistic Regression

Notes

Output Created		18-APR-2018 19:15:...
Comments		
Input	Data	C:\Users\rnordin.ADMIN\Desktop\2018\PUBLICATION 2018 ETS\ETS.Data (Complete).sav 18APRIL2018.sav
	Active Dataset	DataSet1
	Filter	<none>
	Weight	<none>
	Split File	<none>
	N of Rows in Working Data File	118
Missing Value Handling	Definition of Missing	User-defined missing values are treated as missing
Syntax		LOGISTIC REGRESSION VARIABLES Compensatory.sweating /METHOD=ENTER Hospital.stay /CONTRAST (Hospital.stay)=Indicator(1) /PRINT=GOODFIT CI (95) /CRITERIA=PIN(0.05) POUT(0.10) ITERATE(20) CUT(0.5).
Resources	Processor Time	00:00:00.02
	Elapsed Time	00:00:00.02

Case Processing Summary

Unweighted Cases[a]		N	Percent
Selected Cases	Included in Analysis	118	100.0
	Missing Cases	0	.0
	Total	118	100.0
Unselected Cases		0	.0
Total		118	100.0

a. If weight is in effect, see classification table for the total number of cases.

Dependent Variable Encoding

Original Value	Internal Value
No	0
Yes	1

Categorical Variables Codings

			Parameter coding					
		Frequency	(1)	(2)	(3)	(4)	(5)	(6)
Hospital.stay	1.00	1	.000	.000	.000	.000	.000	.000
	2.00	3	1.000	.000	.000	.000	.000	.000
	3.00	68	.000	1.000	.000	.000	.000	.000
	4.00	30	.000	.000	1.000	.000	.000	.000
	5.00	8	.000	.000	.000	1.000	.000	.000
	6.00	7	.000	.000	.000	.000	1.000	.000
	9.00	1	.000	.000	.000	.000	.000	1.000

Block 0: Beginning Block

Classification Table[a,b]

Observed			Predicted		
			CS		Percentage Correct
			No	Yes	
Step 0	CS	No	0	50	.0
		Yes	0	68	100.0
	Overall Percentage				57.6

a. Constant is included in the model.

b. The cut value is .500

Variables in the Equation

		B	S.E.	Wald	df	Sig.	Exp(B)
Step 0	Constant	.307	.186	2.724	1	.099	1.360

Variables not in the Equation

			Score	df	Sig.
Step 0	Variables	Hospital.stay	5.951	6	.429
		Hospital.stay(1)	2.263	1	.132
		Hospital.stay(2)	.467	1	.494
		Hospital.stay(3)	.015	1	.902
		Hospital.stay(4)	.204	1	.651
		Hospital.stay(5)	.665	1	.415
		Hospital.stay(6)	1.372	1	.242
	Overall Statistics		5.951	6	.429

Block 1: Method = Enter

Omnibus Tests of Model Coefficients

		Chi-square	df	Sig.
Step 1	Step	7.756	6	.257
	Block	7.756	6	.257
	Model	7.756	6	.257

Model Summary

Step	-2 Log likelihood	Cox & Snell R Square	Nagelkerke R Square
1	153.070[a]	.064	.085

a. Estimation terminated at iteration number 20 because maximum iterations has been reached. Final solution cannot be found.

Hosmer and Lemeshow Test

Step	Chi-square	df	Sig.
1	.000	2	1.000

Contingency Table for Hosmer and Lemeshow Test

		CS = No		CS = Yes		
		Observed	Expected	Observed	Expected	Total
Step 1	1	6	6.000	3	3.000	9
	2	4	4.000	4	4.000	8
	3	13	13.000	17	17.000	30
	4	27	27.000	44	44.000	71

Classification Table[a]

Observed			Predicted CS No	Predicted CS Yes	Percentage Correct
Step 1	CS	No	6	44	12.0
		Yes	3	65	95.6
	Overall Percentage				60.2

a. The cut value is .500

Variables in the Equation

		B	S.E.	Wald	df	Sig.	Exp(B)	95% C.I.for EXP(B) Lower	95% C.I.for EXP(B) Upper
Step 1[a]	Hospital.stay			1.005	6	.985			
	Hospital.stay(1)	42.406	46414.215	.000	1	.999	2.610E+18	.000	.
	Hospital.stay(2)	21.621	40196.863	.000	1	1.000	2.453E+9	.000	.
	Hospital.stay(3)	21.471	40196.863	.000	1	1.000	2.113E+9	.000	.
	Hospital.stay(4)	21.203	40196.863	.000	1	1.000	1.616E+9	.000	.
	Hospital.stay(5)	20.915	40196.863	.000	1	1.000	1.212E+9	.000	.
	Hospital.stay(6)	.000	56844.196	.000	1	1.000	1.000	.000	.
	Constant	-21.203	40196.863	.000	1	1.000	.000		

a. Variable(s) entered on step 1: Hospital.stay.

```
LOGISTIC REGRESSION VARIABLES Compensatory.sweating
 /METHOD=ENTER ICU.Stay
 /CONTRAST (ICU.Stay)=Indicator(1)
 /PRINT=GOODFIT CI(95)
 /CRITERIA=PIN(0.05) POUT(0.10) ITERATE(20) CUT(0.5).
```

Logistic Regression

Notes

Output Created		18-APR-2018 19:16:...
Comments		
Input	Data	C:\Users\rnordin.ADMIN\Desktop\2018\PUBLICATION 2018 ETS\ETS.Data (Complete).sav 18APRIL2018.sav
	Active Dataset	DataSet1
	Filter	<none>
	Weight	<none>
	Split File	<none>
	N of Rows in Working Data File	118
Missing Value Handling	Definition of Missing	User-defined missing values are treated as missing
Syntax		LOGISTIC REGRESSION VARIABLES Compensatory.sweating /METHOD=ENTER ICU.Stay /CONTRAST (ICU.Stay)=Indicator(1) /PRINT=GOODFIT CI(95) /CRITERIA=PIN(0.05) POUT(0.10) ITERATE(20) CUT(0.5).
Resources	Processor Time	00:00:00.02
	Elapsed Time	00:00:00.02

Case Processing Summary

Unweighted Cases[a]		N	Percent
Selected Cases	Included in Analysis	118	100.0
	Missing Cases	0	.0
	Total	118	100.0
Unselected Cases		0	.0
Total		118	100.0

a. If weight is in effect, see classification table for the total number of cases.

Dependent Variable Encoding

Original Value	Internal Value
No	0
Yes	1

Categorical Variables Codings

		Frequency	Parameter coding (1)
ICU.Stay	Yes	4	.000
	No	114	1.000

Block 0: Beginning Block

Classification Table[a,b]

Observed			Predicted CS No	Predicted CS Yes	Percentage Correct
Step 0	CS	No	0	50	.0
		Yes	0	68	100.0
	Overall Percentage				57.6

a. Constant is included in the model.

b. The cut value is .500

Variables in the Equation

		B	S.E.	Wald	df	Sig.	Exp(B)
Step 0	Constant	.307	.186	2.724	1	.099	1.360

Variables not in the Equation

			Score	df	Sig.
Step 0	Variables	ICU.Stay(1)	.512	1	.474
	Overall Statistics		.512	1	.474

Block 1: Method = Enter

Omnibus Tests of Model Coefficients

		Chi-square	df	Sig.
Step 1	Step	.543	1	.461
	Block	.543	1	.461
	Model	.543	1	.461

Model Summary

Step	-2 Log likelihood	Cox & Snell R Square	Nagelkerke R Square
1	160.283[a]	.005	.006

a. Estimation terminated at iteration number 4 because parameter estimates changed by less than .001.

Hosmer and Lemeshow Test

Step	Chi-square	df	Sig.
1	.000	0	.

Contingency Table for Hosmer and Lemeshow Test

		CS = No		CS = Yes		Total
		Observed	Expected	Observed	Expected	
Step 1	1	50	50.000	68	68.000	118

Classification Table[a]

Observed			Predicted CS No	Predicted CS Yes	Percentage Correct
Step 1	CS	No	0	50	.0
		Yes	0	68	100.0
	Overall Percentage				57.6

a. The cut value is .500

Variables in the Equation

		B	S.E.	Wald	df	Sig.	Exp(B)	95% C.I.for EXP(B) Lower	Upper
Step 1[a]	ICU.Stay(1)	-.816	1.170	.486	1	.486	.442	.045	4.381
	Constant	1.099	1.155	.905	1	.341	3.000		

a. Variable(s) entered on step 1: ICU.Stay.

LOGISTIC REGRESSION VARIABLES Compensatory.sweating

```
/METHOD=ENTER DurationOfSurgery
/CONTRAST (DurationOfSurgery)=Indicator(1)
/PRINT=GOODFIT CI(95)
/CRITERIA=PIN(0.05) POUT(0.10) ITERATE(20) CUT(0.5).
```

Logistic Regression

Notes

Output Created		18-APR-2018 19:16:...
Comments		
Input	Data	C:\Users\rnordin.ADMIN\Desktop\2018\PUBLICATION 2018 ETS\ETS.Data (Complete).sav 18APRIL2018.sav
	Active Dataset	DataSet1
	Filter	<none>
	Weight	<none>
	Split File	<none>
	N of Rows in Working Data File	118
Missing Value Handling	Definition of Missing	User-defined missing values are treated as missing
Syntax		LOGISTIC REGRESSION VARIABLES Compensatory.sweating /METHOD=ENTER DurationOfSurgery /CONTRAST (DurationOfSurgery)=Indicator(1) /PRINT=GOODFIT CI(95) /CRITERIA=PIN(0.05) POUT(0.10) ITERATE(20) CUT(0.5).
Resources	Processor Time	00:00:00.02
	Elapsed Time	00:00:00.01

Case Processing Summary

Unweighted Cases[a]		N	Percent
Selected Cases	Included in Analysis	118	100.0
	Missing Cases	0	.0
	Total	118	100.0
Unselected Cases		0	.0
Total		118	100.0

a. If weight is in effect, see classification table for the total number of cases.

Dependent Variable Encoding

Original Value	Internal Value
No	0
Yes	1

Categorical Variables Codings

		Frequency	Parameter coding (1)
DurationOfSurgery	Median & below	67	.000
	Above median	51	1.000

Block 0: Beginning Block

Classification Table[a,b]

Observed			Predicted CS No	Predicted CS Yes	Percentage Correct
Step 0	CS	No	0	50	.0
		Yes	0	68	100.0
	Overall Percentage				57.6

a. Constant is included in the model.

b. The cut value is .500

Variables in the Equation

		B	S.E.	Wald	df	Sig.	Exp(B)
Step 0	Constant	.307	.186	2.724	1	.099	1.360

Variables not in the Equation

			Score	df	Sig.
Step 0	Variables	DurationOfSurgery(1)	.273	1	.601
	Overall Statistics		.273	1	.601

Block 1: Method = Enter

Omnibus Tests of Model Coefficients

		Chi-square	df	Sig.
Step 1	Step	.273	1	.601
	Block	.273	1	.601
	Model	.273	1	.601

Model Summary

Step	-2 Log likelihood	Cox & Snell R Square	Nagelkerke R Square
1	160.553[a]	.002	.003

a. Estimation terminated at iteration number 3 because parameter estimates changed by less than .001.

Hosmer and Lemeshow Test

Step	Chi-square	df	Sig.
1	.000	0	.

Contingency Table for Hosmer and Lemeshow Test

		CS = No		CS = Yes		
		Observed	Expected	Observed	Expected	Total
Step 1	1	23	23.000	28	28.000	51
	2	27	27.000	40	40.000	67

Classification Table[a]

Observed			Predicted: CS No	Predicted: CS Yes	Predicted: Percentage Correct
Step 1	CS	No	0	50	.0
		Yes	0	68	100.0
	Overall Percentage				57.6

a. The cut value is .500

Variables in the Equation

		B	S.E.	Wald	df	Sig.	Exp(B)	95% C.I.for EXP(B) Lower	95% C.I.for EXP(B) Upper
Step 1[a]	DurationOfSurgery(1)	-.196	.376	.273	1	.601	.822	.393	1.716
	Constant	.393	.249	2.490	1	.115	1.481		

a. Variable(s) entered on step 1: DurationOfSurgery.

```
LOGISTIC REGRESSION VARIABLES Compensatory.sweating
  /METHOD=ENTER Medical.issues
  /CONTRAST (Medical.issues)=Indicator(1)
  /PRINT=GOODFIT CI(95)
  /CRITERIA=PIN(0.05) POUT(0.10) ITERATE(20) CUT(0.5).
```

Logistic Regression

Notes

Output Created		18-APR-2018 19:17:...
Comments		
Input	Data	C:\Users\rnordin.ADMIN\Desktop\2018\PUBLICATION 2018 ETS\ETS.Data (Complete).sav 18APRIL2018.sav
	Active Dataset	DataSet1
	Filter	<none>
	Weight	<none>
	Split File	<none>
	N of Rows in Working Data File	118
Missing Value Handling	Definition of Missing	User-defined missing values are treated as missing
Syntax		LOGISTIC REGRESSION VARIABLES Compensatory.sweating /METHOD=ENTER Medical.issues /CONTRAST (Medical.issues)=Indicator(1) /PRINT=GOODFIT CI (95) /CRITERIA=PIN(0.05) POUT(0.10) ITERATE(20) CUT(0.5).
Resources	Processor Time	00:00:00.02
	Elapsed Time	00:00:00.02

Case Processing Summary

Unweighted Cases[a]		N	Percent
Selected Cases	Included in Analysis	118	100.0
	Missing Cases	0	.0
	Total	118	100.0
Unselected Cases		0	.0
Total		118	100.0

a. If weight is in effect, see classification table for the total number of cases.

Dependent Variable Encoding

Original Value	Internal Value
No	0
Yes	1

Categorical Variables Codings

		Frequency	Parameter coding (1)
MedicalIssue	No	109	.000
	Yes	9	1.000

Block 0: Beginning Block

Classification Table[a,b]

Observed			Predicted CS No	Predicted CS Yes	Percentage Correct
Step 0	CS	No	0	50	.0
		Yes	0	68	100.0
	Overall Percentage				57.6

a. Constant is included in the model.

b. The cut value is .500

Variables in the Equation

		B	S.E.	Wald	df	Sig.	Exp(B)
Step 0	Constant	.307	.186	2.724	1	.099	1.360

Variables not in the Equation

			Score	df	Sig.
Step 0	Variables	MedicalIssue(1)	1.620	1	.203
	Overall Statistics		1.620	1	.203

Block 1: Method = Enter

Omnibus Tests of Model Coefficients

		Chi-square	df	Sig.
Step 1	Step	1.740	1	.187
	Block	1.740	1	.187
	Model	1.740	1	.187

Model Summary

Step	-2 Log likelihood	Cox & Snell R Square	Nagelkerke R Square
1	159.087[a]	.015	.020

a. Estimation terminated at iteration number 4 because parameter estimates changed by less than .001.

Hosmer and Lemeshow Test

Step	Chi-square	df	Sig.
1	.000	0	.

Contingency Table for Hosmer and Lemeshow Test

		CS = No		CS = Yes		
		Observed	Expected	Observed	Expected	Total
Step 1	1	48	48.000	61	61.000	109
	2	2	2.000	7	7.000	9

Classification Table[a]

Observed			Predicted CS No	Predicted CS Yes	Percentage Correct
Step 1	CS	No	0	50	.0
		Yes	0	68	100.0
	Overall Percentage				57.6

a. The cut value is .500

Variables in the Equation

		B	S.E.	Wald	df	Sig.	Exp(B)	95% C.I.for EXP(B) Lower	95% C.I.for EXP(B) Upper
Step 1[a]	MedicalIssue(1)	1.013	.825	1.509	1	.219	2.754	.547	13.866
	Constant	.240	.193	1.543	1	.214	1.271		

a. Variable(s) entered on step 1: MedicalIssue.

```
LOGISTIC REGRESSION VARIABLES Compensatory.sweating
 /METHOD=ENTER FollowupYN
 /CONTRAST (FollowupYN)=Indicator(1)
 /PRINT=GOODFIT CI(95)
 /CRITERIA=PIN(0.05) POUT(0.10) ITERATE(20) CUT(0.5).
```

Logistic Regression

Notes

Output Created		18-APR-2018 19:17:...
Comments		
Input	Data	C:\Users\rnordin.ADMIN\Desktop\2018\PUBLICATION 2018 ETS\ETS.Data (Complete).sav 18APRIL2018.sav
	Active Dataset	DataSet1
	Filter	<none>
	Weight	<none>
	Split File	<none>
	N of Rows in Working Data File	118
Missing Value Handling	Definition of Missing	User-defined missing values are treated as missing
Syntax		LOGISTIC REGRESSION VARIABLES Compensatory.sweating /METHOD=ENTER FollowupYN /CONTRAST (FollowupYN)=Indicator(1) /PRINT=GOODFIT CI(95) /CRITERIA=PIN(0.05) POUT(0.10) ITERATE(20) CUT(0.5).
Resources	Processor Time	00:00:00.02
	Elapsed Time	00:00:00.01

Case Processing Summary

Unweighted Cases[a]		N	Percent
Selected Cases	Included in Analysis	115	97.5
	Missing Cases	3	2.5
	Total	118	100.0
Unselected Cases		0	.0
Total		118	100.0

a. If weight is in effect, see classification table for the total number of cases.

Dependent Variable Encoding

Original Value	Internal Value
No	0
Yes	1

Categorical Variables Codings

		Frequency	Parameter coding (1)
FollowupYN	One	77	.000
	More than one	38	1.000

Block 0: Beginning Block

Classification Table[a,b]

Observed			Predicted CS No	Predicted CS Yes	Percentage Correct
Step 0	CS	No	0	48	.0
		Yes	0	67	100.0
	Overall Percentage				58.3

a. Constant is included in the model.

b. The cut value is .500

Variables in the Equation

		B	S.E.	Wald	df	Sig.	Exp(B)
Step 0	Constant	.333	.189	3.110	1	.078	1.396

Variables not in the Equation

			Score	df	Sig.
Step 0	Variables	FollowupYN(1)	22.737	1	.000
	Overall Statistics		22.737	1	.000

Block 1: Method = Enter

Omnibus Tests of Model Coefficients

		Chi-square	df	Sig.
Step 1	Step	25.529	1	.000
	Block	25.529	1	.000
	Model	25.529	1	.000

Model Summary

Step	-2 Log likelihood	Cox & Snell R Square	Nagelkerke R Square
1	130.742[a]	.199	.268

a. Estimation terminated at iteration number 5 because parameter estimates changed by less than .001.

Hosmer and Lemeshow Test

Step	Chi-square	df	Sig.
1	.000	0	.

Contingency Table for Hosmer and Lemeshow Test

		CS = No		CS = Yes		
		Observed	Expected	Observed	Expected	Total
Step 1	1	44	44.000	33	33.000	77
	2	4	4.000	34	34.000	38

Classification Table[a]

Observed			Predicted CS: No	Predicted CS: Yes	Percentage Correct
Step 1	CS	No	44	4	91.7
		Yes	33	34	50.7
	Overall Percentage				67.8

a. The cut value is .500

Variables in the Equation

		B	S.E.	Wald	df	Sig.	Exp(B)	95% C.I.for EXP(B) Lower	Upper
Step 1[a]	FollowupYN(1)	2.428	.577	17.729	1	.000	11.333	3.661	35.087
	Constant	-.288	.230	1.561	1	.212	.750		

a. Variable(s) entered on step 1: FollowupYN.

```
LOGISTIC REGRESSION VARIABLES Compensatory.sweating
 /METHOD=ENTER Reduction.of.PH
 /CONTRAST (Reduction.of.PH)=Indicator(1)
 /PRINT=GOODFIT CI(95)
 /CRITERIA=PIN(0.05) POUT(0.10) ITERATE(20) CUT(0.5).
```

Logistic Regression

Notes

Output Created		18-APR-2018 19:18:...
Comments		
Input	Data	C:\Users\rnordin.ADMIN\Desktop\2018\PUBLICATION 2018 ETS\ETS.Data (Complete).sav 18APRIL2018.sav
	Active Dataset	DataSet1
	Filter	<none>
	Weight	<none>
	Split File	<none>
	N of Rows in Working Data File	118
Missing Value Handling	Definition of Missing	User-defined missing values are treated as missing
Syntax		LOGISTIC REGRESSION VARIABLES Compensatory.sweating /METHOD=ENTER Reduction.of.PH /CONTRAST (Reduction.of.PH) =Indicator(1) /PRINT=GOODFIT CI (95) /CRITERIA=PIN(0.05) POUT(0.10) ITERATE(20) CUT(0.5).
Resources	Processor Time	00:00:00.02
	Elapsed Time	00:00:00.02

Case Processing Summary

Unweighted Cases[a]		N	Percent
Selected Cases	Included in Analysis	118	100.0
	Missing Cases	0	.0
	Total	118	100.0
Unselected Cases		0	.0
Total		118	100.0

a. If weight is in effect, see classification table for the total number of cases.

Dependent Variable Encoding

Original Value	Internal Value
No	0
Yes	1

Categorical Variables Codings

		Frequency	Parameter coding (1)	(2)
Reduction.of.PH	Complete (95-100%)	114	.000	.000
	No change	2	1.000	.000
	N/A	2	.000	1.000

Block 0: Beginning Block

Classification Table[a,b]

Observed			Predicted CS No	Predicted CS Yes	Percentage Correct
Step 0	CS	No	0	50	.0
		Yes	0	68	100.0
	Overall Percentage				57.6

a. Constant is included in the model.

b. The cut value is .500

Variables in the Equation

		B	S.E.	Wald	df	Sig.	Exp(B)
Step 0	Constant	.307	.186	2.724	1	.099	1.360

Variables not in the Equation

			Score	df	Sig.
Step 0	Variables	Reduction.of.PH	2.829	2	.243
		Reduction.of.PH(1)	.048	1	.826
		Reduction.of.PH(2)	2.767	1	.096
	Overall Statistics		2.829	2	.243

Block 1: Method = Enter

Omnibus Tests of Model Coefficients

		Chi-square	df	Sig.
Step 1	Step	3.543	2	.170
	Block	3.543	2	.170
	Model	3.543	2	.170

Model Summary

Step	-2 Log likelihood	Cox & Snell R Square	Nagelkerke R Square
1	157.283[a]	.030	.040

a. Estimation terminated at iteration number 20 because maximum iterations has been reached. Final solution cannot be found.

Hosmer and Lemeshow Test

Step	Chi-square	df	Sig.
1	.000	0	.

Contingency Table for Hosmer and Lemeshow Test

		CS = No		CS = Yes		
		Observed	Expected	Observed	Expected	Total
Step 1	1	3	3.000	1	1.000	4
	2	47	47.000	67	67.000	114

Classification Table[a]

Observed			Predicted: CS No	Predicted: CS Yes	Predicted: Percentage Correct
Step 1	CS	No	2	48	4.0
		Yes	0	68	100.0
	Overall Percentage				59.3

a. The cut value is .500

Variables in the Equation

		B	S.E.	Wald	df	Sig.	Exp(B)	95% C.I.for EXP(B)	
								Lower	Upper
Step 1[a]	Reduction.of.PH			.062	2	.970			
	Reduction.of.PH(1)	-.355	1.427	.062	1	.804	.701	.043	11.499
	Reduction.of.PH(2)	-21.557	28420.722	.000	1	.999	.000	.000	.
	Constant	.355	.190	3.472	1	.062	1.426		

a. Variable(s) entered on step 1: Reduction.of.PH.

```
 SORT CASES BY Reduction.of.PH(A).
SORT CASES BY Reduction.of.PH(D).
LOGISTIC REGRESSION VARIABLES Compensatory.sweating
  /METHOD=ENTER Medical.issues Sympathectomy.Level FollowupYN
  /CONTRAST (Medical.issues)=Indicator(1)
  /CONTRAST (Sympathectomy.Level)=Indicator(1)
  /CONTRAST (FollowupYN)=Indicator(1)
  /PRINT=GOODFIT CI(95)
  /CRITERIA=PIN(0.05) POUT(0.10) ITERATE(20) CUT(0.5).
```

Logistic Regression

Notes

Output Created		18-APR-2018 19:20:...
Comments		
Input	Data	C:\Users\rnordin.ADMIN\Desktop\2018\PUBLICATION 2018 ETS\ETS.Data (Complete).sav 18APRIL2018.sav
	Active Dataset	DataSet1
	Filter	<none>
	Weight	<none>
	Split File	<none>
	N of Rows in Working Data File	118
Missing Value Handling	Definition of Missing	User-defined missing values are treated as missing

Notes

Syntax		LOGISTIC REGRESSION VARIABLES Compensatory.sweating<br>/METHOD=ENTER Medical.issues Sympathectomy.Level FollowupYN<br>/CONTRAST (Medical.issues)=Indicator(1)<br>/CONTRAST (Sympathectomy.Level)=Indicator(1)<br>/CONTRAST (FollowupYN)=Indicator(1)<br>/PRINT=GOODFIT CI(95)<br>/CRITERIA=PIN(0.05) POUT(0.10) ITERATE(20) CUT(0.5).
Resources	Processor Time	00:00:00.02
	Elapsed Time	00:00:00.01

Case Processing Summary

Unweighted Cases[a]		N	Percent
Selected Cases	Included in Analysis	115	97.5
	Missing Cases	3	2.5
	Total	118	100.0
Unselected Cases		0	.0
Total		118	100.0

a. If weight is in effect, see classification table for the total number of cases.

Dependent Variable Encoding

Original Value	Internal Value
No	0
Yes	1

Categorical Variables Codings

		Frequency	Parameter coding (1)
FollowupYN	One	77	.000
	More than one	38	1.000
Sympathectomy.Level	T2-T4	48	.000
	T2-T3	67	1.000
MedicalIssue	No	106	.000
	Yes	9	1.000

Block 0: Beginning Block

Classification Table[a,b]

Observed			Predicted CS No	Predicted CS Yes	Predicted Percentage Correct
Step 0	CS	No	0	48	.0
		Yes	0	67	100.0
	Overall Percentage				58.3

a. Constant is included in the model.

b. The cut value is .500

Variables in the Equation

		B	S.E.	Wald	df	Sig.	Exp(B)
Step 0	Constant	.333	.189	3.110	1	.078	1.396

Variables not in the Equation

			Score	df	Sig.
Step 0	Variables	MedicalIssue(1)	1.529	1	.216
		Sympathectomy.Level(1)	3.625	1	.057
		FollowupYN(1)	22.737	1	.000
	Overall Statistics		28.780	3	.000

Block 1: Method = Enter

Omnibus Tests of Model Coefficients

		Chi-square	df	Sig.
Step 1	Step	33.615	3	.000
	Block	33.615	3	.000
	Model	33.615	3	.000

Model Summary

Step	-2 Log likelihood	Cox & Snell R Square	Nagelkerke R Square
1	122.655[a]	.253	.341

a. Estimation terminated at iteration number 5 because parameter estimates changed by less than .001.

Hosmer and Lemeshow Test

Step	Chi-square	df	Sig.
1	.109	3	.991

Contingency Table for Hosmer and Lemeshow Test

		CS = No		CS = Yes		
		Observed	Expected	Observed	Expected	Total
Step 1	1	21	20.922	7	7.078	28
	2	21	21.163	22	21.837	43
	3	2	1.915	4	4.085	6
	4	3	2.719	13	13.281	16
	5	1	1.281	21	20.719	22

Classification Table[a]

Observed			Predicted: CS No	Predicted: CS Yes	Percentage Correct
Step 1	CS	No	21	27	43.8
		Yes	7	60	89.6
	Overall Percentage				70.4

a. The cut value is .500

Variables in the Equation

		B	S.E.	Wald	df	Sig.	Exp(B)	95% C.I.for EXP(B) Lower	Upper
Step 1[a]	MedicalIssue(1)	1.341	.915	2.151	1	.143	3.824	.637	22.957
	Sympathectomy.Level(1)	1.115	.466	5.738	1	.017	3.050	1.225	7.595
	FollowupYN(1)	2.670	.610	19.145	1	.000	14.436	4.366	47.731
	Constant	-1.084	.399	7.391	1	.007	.338		

a. Variable(s) entered on step 1: MedicalIssue, Sympathectomy.Level, FollowupYN.

```
LOGISTIC REGRESSION VARIABLES Compensatory.sweating
 /METHOD=FSTEP(COND) Medical.issues Sympathectomy.Level FollowupYN
```

```
/CONTRAST (Medical.issues)=Indicator(1)
/CONTRAST (Sympathectomy.Level)=Indicator(1)
/CONTRAST (FollowupYN)=Indicator(1)
/PRINT=GOODFIT CI(95)
/CRITERIA=PIN(0.05) POUT(0.10) ITERATE(20) CUT(0.5).
```

Logistic Regression

Notes

Output Created		18-APR-2018 19:20:...
Comments		
Input	Data	C:\Users\rnordin.ADMIN\Desktop\2018\PUBLICATION 2018 ETS\ETS.Data (Complete).sav 18APRIL2018.sav
	Active Dataset	DataSet1
	Filter	<none>
	Weight	<none>
	Split File	<none>
	N of Rows in Working Data File	118
Missing Value Handling	Definition of Missing	User-defined missing values are treated as missing
Syntax		LOGISTIC REGRESSION VARIABLES Compensatory.sweating /METHOD=FSTEP (COND) Medical.issues Sympathectomy.Level FollowupYN /CONTRAST (Medical.issues)=Indicator(1) /CONTRAST (Sympathectomy.Level)=Indicator(1) /CONTRAST (FollowupYN)=Indicator(1) /PRINT=GOODFIT CI(95) /CRITERIA=PIN(0.05) POUT(0.10) ITERATE(20) CUT(0.5).
Resources	Processor Time	00:00:00.02
	Elapsed Time	00:00:00.02

Case Processing Summary

Unweighted Cases[a]		N	Percent
Selected Cases	Included in Analysis	115	97.5
	Missing Cases	3	2.5
	Total	118	100.0
Unselected Cases		0	.0
Total		118	100.0

a. If weight is in effect, see classification table for the total number of cases.

Dependent Variable Encoding

Original Value	Internal Value
No	0
Yes	1

Categorical Variables Codings

		Frequency	Parameter coding (1)
FollowupYN	One	77	.000
	More than one	38	1.000
Sympathectomy.Level	T2-T4	48	.000
	T2-T3	67	1.000
MedicalIssue	No	106	.000
	Yes	9	1.000

Block 0: Beginning Block

Classification Table[a,b]

	Observed		Predicted CS No	Predicted CS Yes	Percentage Correct
Step 0	CS	No	0	48	.0
		Yes	0	67	100.0
	Overall Percentage				58.3

a. Constant is included in the model.

b. The cut value is .500

Variables in the Equation

		B	S.E.	Wald	df	Sig.	Exp(B)
Step 0	Constant	.333	.189	3.110	1	.078	1.396

Variables not in the Equation

			Score	df	Sig.
Step 0	Variables	MedicalIssue(1)	1.529	1	.216
		Sympathectomy.Level(1)	3.625	1	.057
		FollowupYN(1)	22.737	1	.000
	Overall Statistics		28.780	3	.000

Block 1: Method = Forward Stepwise (Conditional)

Omnibus Tests of Model Coefficients

		Chi-square	df	Sig.
Step 1	Step	25.529	1	.000
	Block	25.529	1	.000
	Model	25.529	1	.000
Step 2	Step	5.685	1	.017
	Block	31.213	2	.000
	Model	31.213	2	.000

Model Summary

Step	-2 Log likelihood	Cox & Snell R Square	Nagelkerke R Square
1	130.742[a]	.199	.268
2	125.057[a]	.238	.320

a. Estimation terminated at iteration number 5 because parameter estimates changed by less than .001.

Hosmer and Lemeshow Test

Step	Chi-square	df	Sig.
1	.000	0	.
2	.134	2	.935

Contingency Table for Hosmer and Lemeshow Test

		CS = No		CS = Yes		
		Observed	Expected	Observed	Expected	Total
Step 1	1	44	44.000	33	33.000	77
	2	4	4.000	34	34.000	38
Step 2	1	22	22.298	9	8.702	31
	2	22	21.702	24	24.298	46
	3	3	2.702	14	14.298	17
	4	1	1.298	20	19.702	21

Classification Table[a]

			Predicted		
			CS		Percentage Correct
Observed			No	Yes	
Step 1	CS	No	44	4	91.7
		Yes	33	34	50.7
	Overall Percentage				67.8
Step 2	CS	No	22	26	45.8
		Yes	9	58	86.6
	Overall Percentage				69.6

a. The cut value is .500

Variables in the Equation

		B	S.E.	Wald	df	Sig.	Exp(B)	95% C.I.for EXP(B)	
								Lower	Upper
Step 1[a]	FollowupYN(1)	2.428	.577	17.729	1	.000	11.333	3.661	35.087
	Constant	-.288	.230	1.561	1	.212	.750		
Step 2[b]	Sympathectomy.Level(1)	1.054	.454	5.378	1	.020	2.869	1.177	6.991
	FollowupYN(1)	2.607	.601	18.813	1	.000	13.558	4.174	44.038
	Constant	-.941	.378	6.198	1	.013	.390		

a. Variable(s) entered on step 1: FollowupYN.

b. Variable(s) entered on step 2: Sympathectomy.Level.

Model if Term Removed[a]

Variable		Model Log Likelihood	Change in -2 Log Likelihood	df	Sig. of the Change
Step 1	FollowupYN	-78.908	27.075	1	.000
Step 2	Sympathectomy.Level	-65.399	5.741	1	.017
	FollowupYN	-77.114	29.170	1	.000

a. Based on conditional parameter estimates

Variables not in the Equation

			Score	df	Sig.
Step 1	Variables	MedicalIssue(1)	1.884	1	.170
		Sympathectomy.Level(1)	5.573	1	.018
	Overall Statistics		7.711	2	.021
Step 2	Variables	MedicalIssue(1)	2.327	1	.127
	Overall Statistics		2.327	1	.127

```
LOGISTIC REGRESSION VARIABLES Compensatory.sweating
  /METHOD=FSTEP(LR) Medical.issues Sympathectomy.Level FollowupYN
  /CONTRAST (Medical.issues)=Indicator(1)
  /CONTRAST (Sympathectomy.Level)=Indicator(1)
  /CONTRAST (FollowupYN)=Indicator(1)
  /PRINT=GOODFIT CI(95)
  /CRITERIA=PIN(0.05) POUT(0.10) ITERATE(20) CUT(0.5).
```

Logistic Regression

Notes

Output Created		18-APR-2018 19:21:...
Comments		
Input	Data	C:\Users\rnordin.ADMIN\Desktop\2018\PUBLICATION 2018 ETS\ETS.Data (Complete).sav 18APRIL2018.sav
	Active Dataset	DataSet1
	Filter	<none>
	Weight	<none>
	Split File	<none>
	N of Rows in Working Data File	118
Missing Value Handling	Definition of Missing	User-defined missing values are treated as missing

Notes

Syntax		LOGISTIC REGRESSION VARIABLES Compensatory.sweating<br>/METHOD=FSTEP(LR) Medical.issues Sympathectomy.Level FollowupYN<br>/CONTRAST (Medical.issues)=Indicator(1)<br>/CONTRAST (Sympathectomy.Level)=Indicator(1)<br>/CONTRAST (FollowupYN)=Indicator(1)<br>/PRINT=GOODFIT CI(95)<br>/CRITERIA=PIN(0.05) POUT(0.10) ITERATE(20) CUT(0.5).
Resources	Processor Time	00:00:00.02
	Elapsed Time	00:00:00.02

Case Processing Summary

Unweighted Cases[a]		N	Percent
Selected Cases	Included in Analysis	115	97.5
	Missing Cases	3	2.5
	Total	118	100.0
Unselected Cases		0	.0
Total		118	100.0

a. If weight is in effect, see classification table for the total number of cases.

Dependent Variable Encoding

Original Value	Internal Value
No	0
Yes	1

Categorical Variables Codings

		Frequency	Parameter coding (1)
FollowupYN	One	77	.000
	More than one	38	1.000
Sympathectomy.Level	T2-T4	48	.000
	T2-T3	67	1.000
MedicalIssue	No	106	.000
	Yes	9	1.000

Block 0: Beginning Block

Classification Table[a,b]

Observed			Predicted CS No	Predicted CS Yes	Percentage Correct
Step 0	CS	No	0	48	.0
		Yes	0	67	100.0
	Overall Percentage				58.3

a. Constant is included in the model.

b. The cut value is .500

Variables in the Equation

		B	S.E.	Wald	df	Sig.	Exp(B)
Step 0	Constant	.333	.189	3.110	1	.078	1.396

Variables not in the Equation

			Score	df	Sig.
Step 0	Variables	MedicalIssue(1)	1.529	1	.216
		Sympathectomy.Level(1)	3.625	1	.057
		FollowupYN(1)	22.737	1	.000
	Overall Statistics		28.780	3	.000

Block 1: Method = Forward Stepwise (Likelihood Ratio)

Omnibus Tests of Model Coefficients

		Chi-square	df	Sig.
Step 1	Step	25.529	1	.000
	Block	25.529	1	.000
	Model	25.529	1	.000
Step 2	Step	5.685	1	.017
	Block	31.213	2	.000
	Model	31.213	2	.000

Model Summary

Step	-2 Log likelihood	Cox & Snell R Square	Nagelkerke R Square
1	130.742[a]	.199	.268
2	125.057[a]	.238	.320

a. Estimation terminated at iteration number 5 because parameter estimates changed by less than .001.

Hosmer and Lemeshow Test

Step	Chi-square	df	Sig.
1	.000	0	.
2	.134	2	.935

Contingency Table for Hosmer and Lemeshow Test

		CS = No		CS = Yes		
		Observed	Expected	Observed	Expected	Total
Step 1	1	44	44.000	33	33.000	77
	2	4	4.000	34	34.000	38
Step 2	1	22	22.298	9	8.702	31
	2	22	21.702	24	24.298	46
	3	3	2.702	14	14.298	17
	4	1	1.298	20	19.702	21

Classification Table[a]

Observed			Predicted CS No	Predicted CS Yes	Percentage Correct
Step 1	CS	No	44	4	91.7
		Yes	33	34	50.7
	Overall Percentage				67.8
Step 2	CS	No	22	26	45.8
		Yes	9	58	86.6
	Overall Percentage				69.6

a. The cut value is .500

Variables in the Equation

		B	S.E.	Wald	df	Sig.	Exp(B)	95% C.I.for EXP(B) Lower	Upper
Step 1[a]	FollowupYN(1)	2.428	.577	17.729	1	.000	11.333	3.661	35.087
	Constant	-.288	.230	1.561	1	.212	.750		
Step 2[b]	Sympathectomy.Level(1)	1.054	.454	5.378	1	.020	2.869	1.177	6.991
	FollowupYN(1)	2.607	.601	18.813	1	.000	13.558	4.174	44.038
	Constant	-.941	.378	6.198	1	.013	.390		

a. Variable(s) entered on step 1: FollowupYN.

b. Variable(s) entered on step 2: Sympathectomy.Level.

Model if Term Removed

Variable		Model Log Likelihood	Change in -2 Log Likelihood	df	Sig. of the Change
Step 1	FollowupYN	-78.135	25.529	1	.000
Step 2	Sympathectomy.Level	-65.371	5.685	1	.017
	FollowupYN	-76.323	27.589	1	.000

Variables not in the Equation

			Score	df	Sig.
Step 1	Variables	MedicalIssue(1)	1.884	1	.170
		Sympathectomy.Level(1)	5.573	1	.018
	Overall Statistics		7.711	2	.021
Step 2	Variables	MedicalIssue(1)	2.327	1	.127
	Overall Statistics		2.327	1	.127

```
LOGISTIC REGRESSION VARIABLES Compensatory.sweating
 /METHOD=FSTEP(WALD) Medical.issues Sympathectomy.Level FollowupYN
 /CONTRAST (Medical.issues)=Indicator(1)
 /CONTRAST (Sympathectomy.Level)=Indicator(1)
```

```
/CONTRAST (FollowupYN)=Indicator(1)
/PRINT=GOODFIT CI(95)
/CRITERIA=PIN(0.05) POUT(0.10) ITERATE(20) CUT(0.5).
```

Logistic Regression

Notes

Output Created		18-APR-2018 19:21:...
Comments		
Input	Data	C:\Users\rnordin.ADMIN\Desktop\2018\PUBLICATION 2018 ETS\ETS.Data (Complete).sav 18APRIL2018.sav
	Active Dataset	DataSet1
	Filter	<none>
	Weight	<none>
	Split File	<none>
	N of Rows in Working Data File	118
Missing Value Handling	Definition of Missing	User-defined missing values are treated as missing
Syntax		LOGISTIC REGRESSION VARIABLES Compensatory.sweating /METHOD=FSTEP (WALD) Medical.issues Sympathectomy.Level FollowupYN /CONTRAST (Medical.issues)=Indicator(1) /CONTRAST (Sympathectomy.Level)=Indicator(1) /CONTRAST (FollowupYN)=Indicator(1) /PRINT=GOODFIT CI(95) /CRITERIA=PIN(0.05) POUT(0.10) ITERATE(20) CUT(0.5).
Resources	Processor Time	00:00:00.02
	Elapsed Time	00:00:00.02

Case Processing Summary

Unweighted Cases[a]		N	Percent
Selected Cases	Included in Analysis	115	97.5
	Missing Cases	3	2.5
	Total	118	100.0
Unselected Cases		0	.0
Total		118	100.0

a. If weight is in effect, see classification table for the total number of cases.

Dependent Variable Encoding

Original Value	Internal Value
No	0
Yes	1

Categorical Variables Codings

		Frequency	Parameter coding (1)
FollowupYN	One	77	.000
	More than one	38	1.000
Sympathectomy.Level	T2-T4	48	.000
	T2-T3	67	1.000
MedicalIssue	No	106	.000
	Yes	9	1.000

Block 0: Beginning Block

Classification Table[a,b]

	Observed		Predicted CS No	Predicted CS Yes	Percentage Correct
Step 0	CS	No	0	48	.0
		Yes	0	67	100.0
	Overall Percentage				58.3

a. Constant is included in the model.

b. The cut value is .500

Variables in the Equation

		B	S.E.	Wald	df	Sig.	Exp(B)
Step 0	Constant	.333	.189	3.110	1	.078	1.396

Variables not in the Equation

			Score	df	Sig.
Step 0	Variables	MedicalIssue(1)	1.529	1	.216
		Sympathectomy.Level(1)	3.625	1	.057
		FollowupYN(1)	22.737	1	.000
	Overall Statistics		28.780	3	.000

Block 1: Method = Forward Stepwise (Wald)

Omnibus Tests of Model Coefficients

		Chi-square	df	Sig.
Step 1	Step	25.529	1	.000
	Block	25.529	1	.000
	Model	25.529	1	.000
Step 2	Step	5.685	1	.017
	Block	31.213	2	.000
	Model	31.213	2	.000

Model Summary

Step	-2 Log likelihood	Cox & Snell R Square	Nagelkerke R Square
1	130.742[a]	.199	.268
2	125.057[a]	.238	.320

a. Estimation terminated at iteration number 5 because parameter estimates changed by less than .001.

Hosmer and Lemeshow Test

Step	Chi-square	df	Sig.
1	.000	0	.
2	.134	2	.935

Contingency Table for Hosmer and Lemeshow Test

		CS = No		CS = Yes		
		Observed	Expected	Observed	Expected	Total
Step 1	1	44	44.000	33	33.000	77
	2	4	4.000	34	34.000	38
Step 2	1	22	22.298	9	8.702	31
	2	22	21.702	24	24.298	46
	3	3	2.702	14	14.298	17
	4	1	1.298	20	19.702	21

Classification Table[a]

Observed			Predicted: CS No	CS Yes	Percentage Correct
Step 1	CS	No	44	4	91.7
		Yes	33	34	50.7
	Overall Percentage				67.8
Step 2	CS	No	22	26	45.8
		Yes	9	58	86.6
	Overall Percentage				69.6

a. The cut value is .500

Variables in the Equation

		B	S.E.	Wald	df	Sig.	Exp(B)	95% C.I.for EXP(B) Lower	Upper
Step 1[a]	FollowupYN(1)	2.428	.577	17.729	1	.000	11.333	3.661	35.087
	Constant	-.288	.230	1.561	1	.212	.750		
Step 2[b]	Sympathectomy.Level(1)	1.054	.454	5.378	1	.020	2.869	1.177	6.991
	FollowupYN(1)	2.607	.601	18.813	1	.000	13.558	4.174	44.038
	Constant	-.941	.378	6.198	1	.013	.390		

a. Variable(s) entered on step 1: FollowupYN.

b. Variable(s) entered on step 2: Sympathectomy.Level.

Variables not in the Equation

			Score	df	Sig.
Step 1	Variables	MedicalIssue(1)	1.884	1	.170
		Sympathectomy.Level(1)	5.573	1	.018
	Overall Statistics		7.711	2	.021
Step 2	Variables	MedicalIssue(1)	2.327	1	.127
	Overall Statistics		2.327	1	.127

```
LOGISTIC REGRESSION VARIABLES Compensatory.sweating
 /METHOD=FSTEP(WALD) Medical.issues Sympathectomy.Level FollowupYN
 /CONTRAST (Medical.issues)=Indicator(1)
 /CONTRAST (Sympathectomy.Level)=Indicator(1)
 /CONTRAST (FollowupYN)=Indicator(1)
 /SAVE=PRED
 /CLASSPLOT
 /CASEWISE OUTLIER(2)
 /PRINT=GOODFIT ITER(1) CI(95)
 /CRITERIA=PIN(0.05) POUT(0.10) ITERATE(20) CUT(0.5).
```

Logistic Regression

Notes

Output Created		18-APR-2018 20:50:...
Comments		
Input	Data	C:\Users\rnordin.ADMIN\Desktop\2018\PUBLICATION 2018 ETS\ETS.Data (Complete).sav 18APRIL2018.sav
	Active Dataset	DataSet1
	Filter	<none>
	Weight	<none>
	Split File	<none>
	N of Rows in Working Data File	118
Missing Value Handling	Definition of Missing	User-defined missing values are treated as missing
Syntax		LOGISTIC REGRESSION VARIABLES Compensatory.sweating /METHOD=FSTEP (WALD) Medical.issues Sympathectomy.Level FollowupYN /CONTRAST (Medical.issues)=Indicator(1) /CONTRAST (Sympathectomy.Level) =Indicator(1) /CONTRAST (FollowupYN)=Indicator (1) /SAVE=PRED /CLASSPLOT /CASEWISE OUTLIER(2) /PRINT=GOODFIT ITER (1) CI(95) /CRITERIA=PIN(0.05) POUT(0.10) ITERATE(20) CUT(0.5).

Notes

Resources	Processor Time	00:00:00.00
	Elapsed Time	00:00:00.08
Variables Created or Modified	PRE_1	Predicted probability

Case Processing Summary

Unweighted Cases[a]		N	Percent
Selected Cases	Included in Analysis	115	97.5
	Missing Cases	3	2.5
	Total	118	100.0
Unselected Cases		0	.0
Total		118	100.0

a. If weight is in effect, see classification table for the total number of cases.

Dependent Variable Encoding

Original Value	Internal Value
No	0
Yes	1

Categorical Variables Codings

		Frequency	Parameter coding (1)
FollowupYN	One	77	.000
	More than one	38	1.000
Sympathectomy.Level	T2-T4	48	.000
	T2-T3	67	1.000
MedicalIssue	No	106	.000
	Yes	9	1.000

Block 0: Beginning Block

Iteration History[a,b,c]

Iteration		-2 Log likelihood	Coefficients Constant
Step 0	1	156.271	.330
	2	156.270	.333
	3	156.270	.333

a. Constant is included in the model.

b. Initial -2 Log Likelihood: 156.270

c. Estimation terminated at iteration number 3 because parameter estimates changed by less than .001.

Classification Table[a,b]

Observed			Predicted CS No	Predicted CS Yes	Percentage Correct
Step 0	CS	No	0	48	.0
		Yes	0	67	100.0
	Overall Percentage				58.3

a. Constant is included in the model.

b. The cut value is .500

Variables in the Equation

		B	S.E.	Wald	df	Sig.	Exp(B)
Step 0	Constant	.333	.189	3.110	1	.078	1.396

Variables not in the Equation

			Score	df	Sig.
Step 0	Variables	MedicalIssue(1)	1.529	1	.216
		Sympathectomy.Level(1)	3.625	1	.057
		FollowupYN(1)	22.737	1	.000
	Overall Statistics		28.780	3	.000

Block 1: Method = Forward Stepwise (Wald)

Iteration History[a,b,c,d]

Iteration		-2 Log likelihood	Coefficients		
			Constant	FollowupYN(1)	Sympathectomy.Level(1)
Step 1	1	132.047	-.286	1.865	
	2	130.777	-.288	2.330	
	3	130.742	-.288	2.424	
	4	130.742	-.288	2.428	
	5	130.742	-.288	2.428	
Step 2	1	127.007	-.756	1.900	.788
	2	125.119	-.916	2.470	1.018
	3	125.057	-.940	2.601	1.053
	4	125.057	-.941	2.607	1.054
	5	125.057	-.941	2.607	1.054

a. Method: Forward Stepwise (Wald)

b. Constant is included in the model.

c. Initial -2 Log Likelihood: 156.270

d. Estimation terminated at iteration number 5 because parameter estimates changed by less than .001.

Omnibus Tests of Model Coefficients

		Chi-square	df	Sig.
Step 1	Step	25.529	1	.000
	Block	25.529	1	.000
	Model	25.529	1	.000
Step 2	Step	5.685	1	.017
	Block	31.213	2	.000
	Model	31.213	2	.000

Model Summary

Step	-2 Log likelihood	Cox & Snell R Square	Nagelkerke R Square
1	130.742[a]	.199	.268
2	125.057[a]	.238	.320

a. Estimation terminated at iteration number 5 because parameter estimates changed by less than .001.

Hosmer and Lemeshow Test

Step	Chi-square	df	Sig.
1	.000	0	.
2	.134	2	.935

Contingency Table for Hosmer and Lemeshow Test

		CS = No		CS = Yes		
		Observed	Expected	Observed	Expected	Total
Step 1	1	44	44.000	33	33.000	77
	2	4	4.000	34	34.000	38
Step 2	1	22	22.298	9	8.702	31
	2	22	21.702	24	24.298	46
	3	3	2.702	14	14.298	17
	4	1	1.298	20	19.702	21

Classification Table[a]

Observed			Predicted CS No	Predicted CS Yes	Percentage Correct
Step 1	CS	No	44	4	91.7
		Yes	33	34	50.7
	Overall Percentage				67.8
Step 2	CS	No	22	26	45.8
		Yes	9	58	86.6
	Overall Percentage				69.6

a. The cut value is .500

Variables in the Equation

		B	S.E.	Wald	df	Sig.	Exp(B)	95% C.I.for EXP(B) Lower	95% C.I.for EXP(B) Upper
Step 1[a]	FollowupYN(1)	2.428	.577	17.729	1	.000	11.333	3.661	35.087
	Constant	-.288	.230	1.561	1	.212	.750		
Step 2[b]	Sympathectomy.Level(1)	1.054	.454	5.378	1	.020	2.869	1.177	6.991
	FollowupYN(1)	2.607	.601	18.813	1	.000	13.558	4.174	44.038
	Constant	-.941	.378	6.198	1	.013	.390		

a. Variable(s) entered on step 1: FollowupYN.

b. Variable(s) entered on step 2: Sympathectomy.Level.

Variables not in the Equation

			Score	df	Sig.
Step 1	Variables	MedicalIssue(1)	1.884	1	.170
		Sympathectomy.Level(1)	5.573	1	.018
	Overall Statistics		7.711	2	.021
Step 2	Variables	MedicalIssue(1)	2.327	1	.127
	Overall Statistics		2.327	1	.127

```
              Step number: 1

              Observed Groups and Predicted Probabilities

          80 +
                                                +
             I                                                 Y
                                                I
             I                                                 Y
                                                I
F            I                                                 Y
                                                I
R         60 +                                                 Y
                                                +
E            I                                                 Y
                                                I
Q            I                                                 Y
                                                I
U            I                                                 N
                                                I
E         40 +                                                 N
                             Y                  +
N            I                                                 N
                             Y                  I
C            I                                                 N
                             Y                  I
Y            I                                                 N
                             Y                  I
          20 +                                                 N
                             Y                  +
             I                                                 N
                             Y                  I
             I                                                 N
                             Y                  I
             I                                                 N
                             N                  I
Predicted ---------+---------+---------+---------+---------+---------+-----
----+---------+---------+----------
  Prob:   0       .1        .2        .3        .4        .5        .6
   .7        .8        .9         1
```

```
  Group:  NNNNNNNNNNNNNNNNNNNNNNNNNNNNNNNNNNNNNNNNNNNNNNNNNNNNNNNNNNYYYYYYYYYYYYYYY
YYYYYYYYYYYYYYYYYYYYYYYYYYYYYYYYYYY

          Predicted Probability is of Membership for Yes
          The Cut Value is .50
          Symbols: N - No
                   Y - Yes
          Each Symbol Represents 5 Cases.




             Step number: 2

             Observed Groups and Predicted Probabilities

      80 +
                                                      +
         I
                                                      I
         I
                                                      I
F        I
                                                      I
R     60 +
                                                      +
E        I
                                                      I
Q        I
                                                      I
U        I                                                              Y
                                                      I
E     40 +                                                              Y
                                                      +
N        I                                                              Y
                                                      I
C        I                                              Y               Y
                                                      I
Y        I                                              Y               Y
                                                      I
      20 +                                              N               N
                                     Y                +
         I                                              N               N
                            Y        Y                I
         I                                              N               N
                            Y        Y                I
         I                                              N               N
                            N        Y                I
Predicted ---------+---------+---------+---------+---------+---------+-----
----+---------+---------+----------
  Prob:   0       .1        .2        .3        .4        .5        .6
   .7        .8        .9         1
  Group:  NNNNNNNNNNNNNNNNNNNNNNNNNNNNNNNNNNNNNNNNNNNNNNNNNNNNNNNNNNYYYYYYYYYYYYYYY
YYYYYYYYYYYYYYYYYYYYYYYYYYYYYYYYYYY
```

```
Predicted Probability is of Membership for Yes
The Cut Value is .50
Symbols: N - No
         Y - Yes
Each Symbol Represents 5 Cases.
```

Casewise List[b]

Case	Selected Status[a]	Observed	Predicted	Predicted Group	Temporary Variable	
		CS			Resid	ZResid
73	S	N**	.938	Y	-.938	-3.896

a. S = Selected, U = Unselected cases, and ** = Misclassified cases.

b. Cases with studentized residuals greater than 2.000 are listed.

```
ROC PRE_1 BY Compensatory.sweating(1)
 /PLOT=CURVE(REFERENCE)
 /PRINT=SE COORDINATES
 /CRITERIA=CUTOFF(INCLUDE) TESTPOS(LARGE) DISTRIBUTION(FREE) CI(95)
 /MISSING=EXCLUDE.
```

ROC Curve

Notes

Output Created		18-APR-2018 20:53:...
Comments		
Input	Data	C:\Users\rnordin.ADMIN\Desktop\2018\PUBLICATION 2018 ETS\ETS.Data (Complete).sav 18APRIL2018.sav
	Active Dataset	DataSet1
	Filter	<none>
	Weight	<none>
	Split File	<none>
	N of Rows in Working Data File	118
Missing Value Handling	Definition of Missing	User-defined missing values are treated as missing.
	Cases Used	Statistics are based on all cases with valid data for all variables in the analysis.

Notes

Syntax		ROC PRE_1 BY Compensatory.sweating (1)<br>/PLOT=CURVE (REFERENCE)<br>/PRINT=SE COORDINATES<br>/CRITERIA=CUTOFF (INCLUDE) TESTPOS (LARGE) DISTRIBUTION (FREE) CI(95)<br>/MISSING=EXCLUDE.
Resources	Processor Time	00:00:00.27
	Elapsed Time	00:00:00.48

Case Processing Summary

CS	Valid N (listwise)
Positive[a]	67
Negative	48
Missing	3

Larger values of the test result variable(s) indicate stronger evidence for a positive actual state.

a. The positive actual state is Yes.

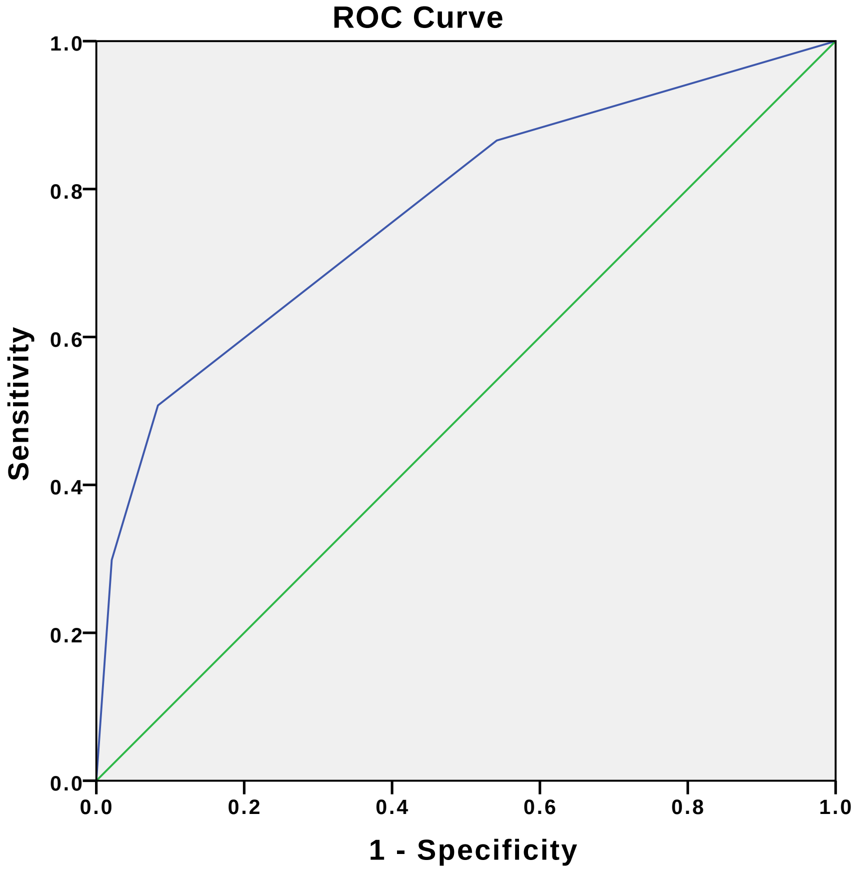

Diagonal segments are produced by ties.

Area Under the Curve

Test Result Variable(s): Predicted probability

Area	Std. Error[a]	Asymptotic Sig.[b]	Asymptotic 95% Confidence Interval	
			Lower Bound	Upper Bound
.771	.043	.000	.686	.855

The test result variable(s): Predicted probability has at least one tie between the positive actual state group and the negative actual state group. Statistics may be biased.

a. Under the nonparametric assumption

b. Null hypothesis: true area = 0.5

Coordinates of the Curve

Test Result Variable(s): Predicted probabilit

Positive if Greater Than or Equal To[a]	Sensitivity	1 - Specificity
.0000000	1.000	1.000
.4044641	.866	.542
.6846337	.507	.083
.8896240	.299	.021
1.0000000	.000	.000

The test result variable(s): Predicted probability has at least one tie between the positive actual state group and the negative actual state group.

a. The smallest cutoff value is the minimum observed test value minus 1, and the largest cutoff value is the maximum observed test value plus 1. All the other cutoff values are the averages of two consecutive ordered observed test values.

```
LOGISTIC REGRESSION VARIABLES Compensatory.sweating
 /METHOD=ENTER Sympathectomy.LevelFollowupYN
 /CONTRAST (Sympathectomy.Level)=Indicator(1)
 /CONTRAST (FollowupYN)=Indicator(1)
 /SAVE=PRED
 /CLASSPLOT
 /CASEWISE OUTLIER(2)
 /PRINT=GOODFIT ITER(1) CI(95)
 /CRITERIA=PIN(0.05) POUT(0.10) ITERATE(20) CUT(0.5).
```

Logistic Regression

Notes

Output Created		18-APR-2018 20:56:...
Comments		
Input	Data	C:\Users\rnordin.ADMIN\Desktop\2018\PUBLICATION 2018 ETS\ETS.Data (Complete).sav 18APRIL2018.sav
	Active Dataset	DataSet1
	Filter	<none>
	Weight	<none>
	Split File	<none>
	N of Rows in Working Data File	118
Missing Value Handling	Definition of Missing	User-defined missing values are treated as missing
Syntax		LOGISTIC REGRESSION VARIABLES Compensatory.sweating /METHOD=ENTER Sympathectomy.Level FollowupYN /CONTRAST (Sympathectomy.Level)=Indicator(1) /CONTRAST (FollowupYN)=Indicator(1) /SAVE=PRED /CLASSPLOT /CASEWISE OUTLIER(2) /PRINT=GOODFIT ITER(1) CI(95) /CRITERIA=PIN(0.05) POUT(0.10) ITERATE(20) CUT(0.5).
Resources	Processor Time	00:00:00.02
	Elapsed Time	00:00:00.02
Variables Created or Modified	PRE_2	Predicted probability

Case Processing Summary

Unweighted Cases[a]		N	Percent
Selected Cases	Included in Analysis	115	97.5
	Missing Cases	3	2.5
	Total	118	100.0
Unselected Cases		0	.0
Total		118	100.0

a. If weight is in effect, see classification table for the total number of cases.

Dependent Variable Encoding

Original Value	Internal Value
No	0
Yes	1

Categorical Variables Codings

		Frequency	Parameter coding (1)
FollowupYN	One	77	.000
	More than one	38	1.000
Sympathectomy.Level	T2-T4	48	.000
	T2-T3	67	1.000

Block 0: Beginning Block

Iteration History[a,b,c]

Iteration		-2 Log likelihood	Coefficients Constant
Step 0	1	156.271	.330
	2	156.270	.333
	3	156.270	.333

a. Constant is included in the model.

b. Initial -2 Log Likelihood: 156.270

c. Estimation terminated at iteration number 3 because parameter estimates changed by less than .001.

Classification Table[a,b]

Observed			Predicted: CS No	Predicted: CS Yes	Predicted: Percentage Correct
Step 0	CS	No	0	48	.0
		Yes	0	67	100.0
	Overall Percentage				58.3

a. Constant is included in the model.

b. The cut value is .500

Variables in the Equation

		B	S.E.	Wald	df	Sig.	Exp(B)
Step 0	Constant	.333	.189	3.110	1	.078	1.396

Variables not in the Equation

			Score	df	Sig.
Step 0	Variables	Sympathectomy.Level(1)	3.625	1	.057
		FollowupYN(1)	22.737	1	.000
	Overall Statistics		27.187	2	.000

Block 1: Method = Enter

Iteration History[a,b,c,d]

Iteration		-2 Log likelihood	Coefficients: Constant	Coefficients: Sympathectomy.Level(1)	Coefficients: FollowupYN(1)
Step 1	1	127.007	-.756	.788	1.900
	2	125.119	-.916	1.018	2.470
	3	125.057	-.940	1.053	2.601
	4	125.057	-.941	1.054	2.607
	5	125.057	-.941	1.054	2.607

a. Method: Enter

b. Constant is included in the model.

c. Initial -2 Log Likelihood: 156.270

d. Estimation terminated at iteration number 5 because parameter estimates changed by less than .001.

Omnibus Tests of Model Coefficients

		Chi-square	df	Sig.
Step 1	Step	31.213	2	.000
	Block	31.213	2	.000
	Model	31.213	2	.000

Model Summary

Step	-2 Log likelihood	Cox & Snell R Square	Nagelkerke R Square
1	125.057[a]	.238	.320

a. Estimation terminated at iteration number 5 because parameter estimates changed by less than .001.

Hosmer and Lemeshow Test

Step	Chi-square	df	Sig.
1	.134	2	.935

Contingency Table for Hosmer and Lemeshow Test

		CS = No		CS = Yes		
		Observed	Expected	Observed	Expected	Total
Step 1	1	22	22.298	9	8.702	31
	2	22	21.702	24	24.298	46
	3	3	2.702	14	14.298	17
	4	1	1.298	20	19.702	21

Classification Table[a]

Observed			Predicted CS No	Predicted CS Yes	Percentage Correct
Step 1	CS	No	22	26	45.8
		Yes	9	58	86.6
	Overall Percentage				69.6

a. The cut value is .500

Variables in the Equation

		B	S.E.	Wald	df	Sig.	Exp(B)	95% C.I.for EXP(B)	
								Lower	Upper
Step 1[a]	Sympathectomy.Level(1)	1.054	.454	5.378	1	.020	2.869	1.177	6.991
	FollowupYN(1)	2.607	.601	18.813	1	.000	13.558	4.174	44.038
	Constant	-.941	.378	6.198	1	.013	.390		

a. Variable(s) entered on step 1: Sympathectomy.Level, FollowupYN.

```
             Step number: 1

             Observed Groups and Predicted Probabilities

      80 +
                                   +
         I
                                   I
         I
                                   I
F        I
                                   I
R     60 +
                                   +
E        I
                                   I
Q        I
                                   I
U        I                                                Y
                                   I
E     40 +                                                Y
                                   +
N        I                                                Y
                                   I
C        I                            Y                   Y
                                   I
Y        I                            Y                   Y
                                   I
      20 +                            N                   N
                            Y      +
         I                            N                   N
                   Y        Y      I
         I                            N                   N
                   Y        Y      I
         I                            N                   N
                   N        Y      I
Predicted ---------+---------+---------+---------+---------+---------+-----
----+---------+---------+----------
  Prob:   0        .1         .2         .3         .4         .5         .6
   .7         .8         .9          1
```

```
  Group:  NNNNNNNNNNNNNNNNNNNNNNNNNNNNNNNNNNNNNNNNNNNNNNNNNNNNYYYYYYYYYYYYYY
YYYYYYYYYYYYYYYYYYYYYYYYYYYYYYYYYYY

          Predicted Probability is of Membership for Yes
          The Cut Value is .50
          Symbols: N - No
                   Y - Yes
          Each Symbol Represents 5 Cases.
```

Casewise List[b]

Case	Selected Status[a]	Observed CS	Predicted	Predicted Group	Temporary Variable Resid	Temporary Variable ZResid
73	S	N**	.938	Y	-.938	-3.896

a. S = Selected, U = Unselected cases, and ** = Misclassified cases.

b. Cases with studentized residuals greater than 2.000 are listed.

```
 SORT CASES BY Follow.up (A).
SORT CASES BY Follow.up (D).
SORT CASES BY Number.of.follow.up(A).
SORT CASES BY Number.of.follow.up(D).
LOGISTIC REGRESSION VARIABLES Compensatory.sweating
  /METHOD=ENTER Sympathectomy.Level FollowupYN Medical.issues Age Sex Race
  /CONTRAST (Sympathectomy.Level)=Indicator(1)
  /CONTRAST (FollowupYN)=Indicator(1)
  /CONTRAST (Medical.issues)=Indicator(1)
  /CONTRAST (Sex)=Indicator(1)
  /CONTRAST (Race)=Indicator(1)
  /SAVE=PRED
  /CLASSPLOT
  /CASEWISE OUTLIER(2)
  /PRINT=GOODFIT ITER(1) CI(95)
  /CRITERIA=PIN(0.05) POUT(0.10) ITERATE(20) CUT(0.5).
```

Logistic Regression

Notes

Output Created		18-APR-2018 21:54:...
Comments		
Input	Data	C:\Users\rnordin.ADMIN\Desktop\2018\PUBLICATION 2018 ETS\ETS.Data (Complete).sav 18APRIL2018.sav
	Active Dataset	DataSet1
	Filter	<none>
	Weight	<none>
	Split File	<none>
	N of Rows in Working Data File	118
Missing Value Handling	Definition of Missing	User-defined missing values are treated as missing
Syntax		LOGISTIC REGRESSION VARIABLES Compensatory.sweating<br>/METHOD=ENTER Sympathectomy.Level FollowupYN Medical.issues Age Sex Race<br>/CONTRAST (Sympathectomy.Level)=Indicator(1)<br>/CONTRAST (FollowupYN)=Indicator(1)<br>/CONTRAST (Medical.issues)=Indicator(1)<br>/CONTRAST (Sex)=Indicator(1)<br>/CONTRAST (Race)=Indicator(1)<br>/SAVE=PRED<br>/CLASSPLOT<br>/CASEWISE OUTLIER(2)<br>/PRINT=GOODFIT ITER(1) CI(95)<br>/CRITERIA=PIN(0.05) POUT(0.10) ITERATE(20) CUT(0.5).
Resources	Processor Time	00:00:00.02
	Elapsed Time	00:00:00.02
Variables Created or Modified	PRE_3	Predicted probability

Case Processing Summary

Unweighted Cases[a]		N	Percent
Selected Cases	Included in Analysis	115	97.5
	Missing Cases	3	2.5
	Total	118	100.0
Unselected Cases		0	.0
Total		118	100.0

a. If weight is in effect, see classification table for the total number of cases.

Dependent Variable Encoding

Original Value	Internal Value
No	0
Yes	1

Categorical Variables Codings

		Frequency	Parameter coding (1)	Parameter coding (2)
Race	Malay	91	.000	.000
	Chinese	16	1.000	.000
	Indian	8	.000	1.000
FollowupYN	One	77	.000	
	More than one	38	1.000	
MedicalIssue	No	106	.000	
	Yes	9	1.000	
Sex	Male	48	.000	
	Female	67	1.000	
Sympathectomy.Level	T2-T4	48	.000	
	T2-T3	67	1.000	

Block 0: Beginning Block

Iteration History[a,b,c]

Iteration		-2 Log likelihood	Coefficients Constant
Step 0	1	156.271	.330
	2	156.270	.333
	3	156.270	.333

a. Constant is included in the model.

b. Initial -2 Log Likelihood: 156.270

c. Estimation terminated at iteration number 3 because parameter estimates changed by less than .001.

Classification Table[a,b]

Observed			Predicted CS No	Predicted CS Yes	Percentage Correct
Step 0	CS	No	0	48	.0
		Yes	0	67	100.0
	Overall Percentage				58.3

a. Constant is included in the model.

b. The cut value is .500

Variables in the Equation

		B	S.E.	Wald	df	Sig.	Exp(B)
Step 0	Constant	.333	.189	3.110	1	.078	1.396

Variables not in the Equation

			Score	df	Sig.
Step 0	Variables	Sympathectomy.Level(1)	3.625	1	.057
		FollowupYN(1)	22.737	1	.000
		MedicalIssue(1)	1.529	1	.216
		Age	.420	1	.517
		Sex(1)	1.293	1	.256
		Race	.995	2	.608
		Race(1)	.031	1	.860
		Race(2)	.991	1	.320
	Overall Statistics		29.239	7	.000

Block 1: Method = Enter

Iteration History[a,b,c,d]

Iteration		-2 Log likelihood	Coefficients							
			Constant	Sympathectomy.Level(1)	FollowupYN(1)	MedicalIssue(1)	Age	Sex(1)	Race(1)	Race(2)
Step 1	1	124.511	-1.055	.799	1.874	.846	.010	-.035	-.047	.441
	2	121.989	-1.346	1.065	2.483	1.272	.012	-.012	-.041	.702
	3	121.885	-1.399	1.124	2.644	1.360	.012	-.007	-.036	.776
	4	121.884	-1.402	1.126	2.654	1.364	.012	-.007	-.035	.780
	5	121.884	-1.402	1.126	2.654	1.364	.012	-.007	-.035	.780

a. Method: Enter

b. Constant is included in the model.

c. Initial -2 Log Likelihood: 156.270

d. Estimation terminated at iteration number 5 because parameter estimates changed by less than .001.

Omnibus Tests of Model Coefficients

		Chi-square	df	Sig.
Step 1	Step	34.386	7	.000
	Block	34.386	7	.000
	Model	34.386	7	.000

Model Summary

Step	-2 Log likelihood	Cox & Snell R Square	Nagelkerke R Square
1	121.884[a]	.258	.348

a. Estimation terminated at iteration number 5 because parameter estimates changed by less than .001.

Hosmer and Lemeshow Test

Step	Chi-square	df	Sig.
1	4.506	8	.809

Contingency Table for Hosmer and Lemeshow Test

		CS = No		CS = Yes		
		Observed	Expected	Observed	Expected	Total
Step 1	1	10	9.220	2	2.780	12
	2	7	9.030	5	2.970	12
	3	8	6.331	3	4.669	11
	4	5	6.175	7	5.825	12
	5	7	5.986	5	6.014	12
	6	6	6.021	7	6.979	13
	7	2	2.552	10	9.448	12
	8	2	1.655	10	10.345	12
	9	1	.794	11	11.206	12
	10	0	.237	7	6.763	7

Classification Table[a]

Observed			Predicted		
			CS		Percentage Correct
			No	Yes	
Step 1	CS	No	33	15	68.8
		Yes	20	47	70.1
	Overall Percentage				69.6

a. The cut value is .500

Variables in the Equation

		B	S.E.	Wald	df	Sig.	Exp(B)	95% C.I.for EXP(B)	
								Lower	Upper
Step 1[a]	Sympathectomy.Level(1)	1.126	.475	5.612	1	.018	3.084	1.215	7.831
	FollowupYN(1)	2.654	.624	18.110	1	.000	14.208	4.185	48.232
	MedicalIssue(1)	1.364	.945	2.083	1	.149	3.911	.614	24.919
	Age	.012	.035	.120	1	.729	1.012	.945	1.084
	Sex(1)	-.007	.466	.000	1	.989	.993	.399	2.474
	Race			.633	2	.729			
	Race(1)	-.035	.692	.003	1	.959	.965	.249	3.749
	Race(2)	.780	.993	.617	1	.432	2.182	.311	15.281
	Constant	-1.402	.862	2.645	1	.104	.246		

a. Variable(s) entered on step 1: Sympathectomy.Level, FollowupYN, MedicalIssue, Age, Sex, Race.

```
Step number: 1

Observed Groups and Predicted Probabilities
```

```
      16 +
                                   +
         I
                                   I
         I
                                   I
F        I
                                   I
R     12 +
                                   +
E        I                                              Y
                                   I
Q        I                       Y                      Y
                            Y      I
U        I                       Y                      Y
                            Y      I
E      8 +                       N                      Y
                            Y      +
N        I                       N                      YY
                            Y      I
C        I                       NYY                   YYYY
                            Y      I
Y        I                       NYY                   YYYY
                YY          Y      I
       4 +                       NYN                   YNNN Y
                YY         YY      +
         I                      NNNN                   NNNNYN
               YYY         YY   Y  I
         I                      NNNN                   NNNNYN Y  Y
              YYYYY        YYY Y Y I
         I                      NNNN  N          N  N  N NNNNNNNYNY N
 Y   Y        NYNNN   Y   YYNYYY Y I
Predicted ---------+---------+---------+---------+---------+---------+-----
----+---------+---------+----------
  Prob:   0        .1        .2        .3        .4        .5        .6
   .7        .8        .9         1
  Group:  NNNNNNNNNNNNNNNNNNNNNNNNNNNNNNNNNNNNNNNNNNNNNNNNNNNNNNNNNNNYYYYYYYYYYYYYY
YYYYYYYYYYYYYYYYYYYYYYYYYYYYYYYYYYY

          Predicted Probability is of Membership for Yes
          The Cut Value is .50
          Symbols: N - No
                   Y - Yes
          Each Symbol Represents 1 Case.
```

Casewise List[b]

Case	Selected Status[a]	Observed	Predicted	Predicted Group	Temporary Variable	
		CS			Resid	ZResid
24	S	N**	.932	Y	-.932	-3.689

a. S = Selected, U = Unselected cases, and ** = Misclassified cases.

b. Cases with studentized residuals greater than 2.000 are listed.

```
LOGISTIC REGRESSION VARIABLES Compensatory.sweating
 /METHOD=ENTER Sympathectomy.Level
 /CONTRAST (Sympathectomy.Level)=Indicator(1)
 /SAVE=PRED
 /CLASSPLOT
 /CASEWISE OUTLIER(2)
 /PRINT=GOODFIT ITER(1) CI(95)
 /CRITERIA=PIN(0.05) POUT(0.10) ITERATE(20) CUT(0.5).
```

Logistic Regression

Notes

Output Created		18-APR-2018 21:59:...
Comments		
Input	Data	C:\Users\rnordin.ADMIN\Desktop\2018\PUBLICATION 2018 ETS\ETS.Data (Complete).sav 18APRIL2018.sav
	Active Dataset	DataSet1
	Filter	<none>
	Weight	<none>
	Split File	<none>
	N of Rows in Working Data File	118
Missing Value Handling	Definition of Missing	User-defined missing values are treated as missing
Syntax		LOGISTIC REGRESSION VARIABLES Compensatory.sweating /METHOD=ENTER Sympathectomy.Level /CONTRAST (Sympathectomy.Level)=Indicator(1) /SAVE=PRED /CLASSPLOT /CASEWISE OUTLIER(2) /PRINT=GOODFIT ITER(1) CI(95) /CRITERIA=PIN(0.05) POUT(0.10) ITERATE(20) CUT(0.5).
Resources	Processor Time	00:00:00.00
	Elapsed Time	00:00:00.02
Variables Created or Modified	PRE_4	Predicted probability

Case Processing Summary

Unweighted Cases[a]		N	Percent
Selected Cases	Included in Analysis	118	100.0
	Missing Cases	0	.0
	Total	118	100.0
Unselected Cases		0	.0
Total		118	100.0

a. If weight is in effect, see classification table for the total number of cases.

Dependent Variable Encoding

Original Value	Internal Value
No	0
Yes	1

Categorical Variables Codings

		Frequency	Parameter coding (1)
Sympathectomy.Level	T2-T4	51	.000
	T2-T3	67	1.000

Block 0: Beginning Block

Iteration History[a,b,c]

Iteration		-2 Log likelihood	Coefficients Constant
Step 0	1	160.826	.305
	2	160.826	.307
	3	160.826	.307

a. Constant is included in the model.

b. Initial -2 Log Likelihood: 160.826

c. Estimation terminated at iteration number 3 because parameter estimates changed by less than .001.

Classification Table[a,b]

Observed			Predicted CS No	Predicted CS Yes	Percentage Correct
Step 0	CS	No	0	50	.0
		Yes	0	68	100.0
	Overall Percentage				57.6

a. Constant is included in the model.

b. The cut value is .500

Variables in the Equation

		B	S.E.	Wald	df	Sig.	Exp(B)
Step 0	Constant	.307	.186	2.724	1	.099	1.360

Variables not in the Equation

			Score	df	Sig.
Step 0	Variables	Sympathectomy.Level(1)	4.108	1	.043
	Overall Statistics		4.108	1	.043

Block 1: Method = Enter

Iteration History[a,b,c,d]

Iteration		-2 Log likelihood	Coefficients Constant	Coefficients Sympathectomy.Level(1)
Step 1	1	156.719	-.118	.745
	2	156.712	-.118	.766
	3	156.712	-.118	.766

a. Method: Enter

b. Constant is included in the model.

c. Initial -2 Log Likelihood: 160.826

d. Estimation terminated at iteration number 3 because parameter estimates changed by less than .001.

Omnibus Tests of Model Coefficients

		Chi-square	df	Sig.
Step 1	Step	4.114	1	.043
	Block	4.114	1	.043
	Model	4.114	1	.043

Model Summary

Step	-2 Log likelihood	Cox & Snell R Square	Nagelkerke R Square
1	156.712[a]	.034	.046

a. Estimation terminated at iteration number 3 because parameter estimates changed by less than .001.

Hosmer and Lemeshow Test

Step	Chi-square	df	Sig.
1	.000	0	.

Contingency Table for Hosmer and Lemeshow Test

		CS = No		CS = Yes		Total
		Observed	Expected	Observed	Expected	
Step 1	1	27	27.000	24	24.000	51
	2	23	23.000	44	44.000	67

Classification Table[a]

Observed			Predicted CS No	Predicted CS Yes	Percentage Correct
Step 1	CS	No	27	23	54.0
		Yes	24	44	64.7
	Overall Percentage				60.2

a. The cut value is .500

Variables in the Equation

		B	S.E.	Wald	df	Sig.	Exp(B)	95% C.I.for EXP(B) Lower	Upper
Step 1[a]	Sympathectomy.Level(1)	.766	.381	4.054	1	.044	2.152	1.021	4.538
	Constant	-.118	.281	.176	1	.675	.889		

a. Variable(s) entered on step 1: Sympathectomy.Level.

```
        Step number: 1

        Observed Groups and Predicted Probabilities

    80 +
                                 +
```

```
         I
                                    I
         I
                                    I
F        I
Y                                   I
R     60 +
Y                                   +
E        I
Y                                   I
Q        I                                                Y
Y                                   I
U        I                                                Y
Y                                   I
E     40 +                                                Y
Y                                   +
N        I                                                Y
Y                                   I
C        I                                                Y
Y                                   I
Y        I                                                N
N                                   I
      20 +                                                N
N                                   +
         I                                                N
N                                   I
         I                                                N
N                                   I
         I                                                N
N                                   I
Predicted ---------+---------+---------+---------+---------+---------+-----
----+---------+---------+----------
  Prob:   0        .1        .2        .3        .4        .5        .6
   .7        .8        .9        1
  Group:  NNNNNNNNNNNNNNNNNNNNNNNNNNNNNNNNNNNNNNNNNNNNNNNNNNNYYYYYYYYYYYYYY
YYYYYYYYYYYYYYYYYYYYYYYYYYYYYYYYYYY

          Predicted Probability is of Membership for Yes
          The Cut Value is .50
          Symbols: N - No
                   Y - Yes
          Each Symbol Represents 5 Cases.
```

Casewise List[a]

---

a. The casewise plot is not produced because no outliers were found.

```
LOGISTIC REGRESSION VARIABLES Compensatory.sweating
  /METHOD=ENTER Medical.issues
  /CONTRAST (Medical.issues)=Indicator(1)
  /SAVE=PRED
  /CLASSPLOT
  /CASEWISE OUTLIER(2)
  /PRINT=GOODFIT ITER(1) CI(95)
  /CRITERIA=PIN(0.05) POUT(0.10) ITERATE(20) CUT(0.5).
```

Logistic Regression

Notes

Output Created		18-APR-2018 22:01:...
Comments		
Input	Data	C:\Users\rnordin.ADMIN\Desktop\2018\PUBLICATION 2018 ETS\ETS.Data (Complete).sav 18APRIL2018.sav
	Active Dataset	DataSet1
	Filter	<none>
	Weight	<none>
	Split File	<none>
	N of Rows in Working Data File	118
Missing Value Handling	Definition of Missing	User-defined missing values are treated as missing
Syntax		LOGISTIC REGRESSION VARIABLES Compensatory.sweating /METHOD=ENTER Medical.issues /CONTRAST (Medical.issues)=Indicator(1) /SAVE=PRED /CLASSPLOT /CASEWISE OUTLIER(2) /PRINT=GOODFIT ITER (1) CI(95) /CRITERIA=PIN(0.05) POUT(0.10) ITERATE(20) CUT(0.5).
Resources	Processor Time	00:00:00.03
	Elapsed Time	00:00:00.03
Variables Created or Modified	PRE_5	Predicted probability

Case Processing Summary

Unweighted Cases[a]		N	Percent
Selected Cases	Included in Analysis	118	100.0
	Missing Cases	0	.0
	Total	118	100.0
Unselected Cases		0	.0
Total		118	100.0

a. If weight is in effect, see classification table for the total number of cases.

Dependent Variable Encoding

Original Value	Internal Value
No	0
Yes	1

Categorical Variables Codings

		Frequency	Parameter coding (1)
MedicalIssue	No	109	.000
	Yes	9	1.000

Block 0: Beginning Block

Iteration History[a,b,c]

Iteration		-2 Log likelihood	Coefficients Constant
Step 0	1	160.826	.305
	2	160.826	.307
	3	160.826	.307

a. Constant is included in the model.

b. Initial -2 Log Likelihood: 160.826

c. Estimation terminated at iteration number 3 because parameter estimates changed by less than .001.

Classification Table[a,b]

Observed			Predicted CS No	Predicted CS Yes	Percentage Correct
Step 0	CS	No	0	50	.0
		Yes	0	68	100.0
	Overall Percentage				57.6

a. Constant is included in the model.

b. The cut value is .500

Variables in the Equation

		B	S.E.	Wald	df	Sig.	Exp(B)
Step 0	Constant	.307	.186	2.724	1	.099	1.360

Variables not in the Equation

			Score	df	Sig.
Step 0	Variables	MedicalIssue(1)	1.620	1	.203
	Overall Statistics		1.620	1	.203

Block 1: Method = Enter

Iteration History[a,b,c,d]

Iteration		-2 Log likelihood	Coefficients Constant	Coefficients MedicalIssue (1)
Step 1	1	159.119	.239	.873
	2	159.087	.240	1.008
	3	159.087	.240	1.013
	4	159.087	.240	1.013

a. Method: Enter

b. Constant is included in the model.

c. Initial -2 Log Likelihood: 160.826

d. Estimation terminated at iteration number 4 because parameter estimates changed by less than .001.

Omnibus Tests of Model Coefficients

		Chi-square	df	Sig.
Step 1	Step	1.740	1	.187
	Block	1.740	1	.187
	Model	1.740	1	.187

Model Summary

Step	-2 Log likelihood	Cox & Snell R Square	Nagelkerke R Square
1	159.087[a]	.015	.020

a. Estimation terminated at iteration number 4 because parameter estimates changed by less than .001.

Hosmer and Lemeshow Test

Step	Chi-square	df	Sig.
1	.000	0	.

Contingency Table for Hosmer and Lemeshow Test

		CS = No		CS = Yes		
		Observed	Expected	Observed	Expected	Total
Step 1	1	48	48.000	61	61.000	109
	2	2	2.000	7	7.000	9

Classification Table[a]

Observed			Predicted		
			CS		Percentage Correct
			No	Yes	
Step 1	CS	No	0	50	.0
		Yes	0	68	100.0
	Overall Percentage				57.6

a. The cut value is .500

Variables in the Equation

		B	S.E.	Wald	df	Sig.	Exp(B)	95% C.I.for EXP(B)	
								Lower	Upper
Step 1[a]	MedicalIssue(1)	1.013	.825	1.509	1	.219	2.754	.547	13.866
	Constant	.240	.193	1.543	1	.214	1.271		

a. Variable(s) entered on step 1: MedicalIssue.

```
          Step number: 1

          Observed Groups and Predicted Probabilities

160 +
                              +
```

```
         I
                                    I
         I
                                    I
F        I
                                    I
R    120 +
                                    +
E        I                                                            Y
                                    I
Q        I                                                            Y
                                    I
U        I                                                            Y
                                    I
E     80 +                                                            Y
                                    +
N        I                                                            Y
                                    I
C        I                                                            Y
                                    I
Y        I                                                            N
                                    I
      40 +                                                            N
                                    +
         I                                                            N
                                    I
         I                                                            N
                                    I
         I                                                            N
            Y                       I
Predicted ---------+---------+---------+---------+---------+---------+-----
----+---------+---------+----------
  Prob:   0       .1        .2        .3        .4        .5        .6
   .7        .8        .9        1
  Group:  NNNNNNNNNNNNNNNNNNNNNNNNNNNNNNNNNNNNNNNNNNNNNNNNNYYYYYYYYYYYYYY
YYYYYYYYYYYYYYYYYYYYYYYYYYYYYYYYYYY

          Predicted Probability is of Membership for Yes
          The Cut Value is .50
          Symbols: N - No
                   Y - Yes
          Each Symbol Represents 10 Cases.
```

Casewise List[a]

a. The casewise plot is not produced because no outliers were found.

```
LOGISTIC REGRESSION VARIABLES Compensatory.sweating
 /METHOD=ENTER FollowupYN
 /SAVE=PRED
 /CLASSPLOT
 /CASEWISE OUTLIER(2)
 /PRINT=GOODFIT ITER(1) CI(95)
 /CRITERIA=PIN(0.05) POUT(0.10) ITERATE(20) CUT(0.5).
```

Logistic Regression

Notes

Output Created		18-APR-2018 22:02:...
Comments		
Input	Data	C:\Users\rnordin.ADMIN\Desktop\2018\PUBLICATION 2018 ETS\ETS.Data (Complete).sav 18APRIL2018.sav
	Active Dataset	DataSet1
	Filter	<none>
	Weight	<none>
	Split File	<none>
	N of Rows in Working Data File	118
Missing Value Handling	Definition of Missing	User-defined missing values are treated as missing
Syntax		LOGISTIC REGRESSION VARIABLES Compensatory.sweating /METHOD=ENTER FollowupYN /SAVE=PRED /CLASSPLOT /CASEWISE OUTLIER(2) /PRINT=GOODFIT ITER (1) CI(95) /CRITERIA=PIN(0.05) POUT(0.10) ITERATE(20) CUT(0.5).
Resources	Processor Time	00:00:00.00
	Elapsed Time	00:00:00.02
Variables Created or Modified	PRE_6	Predicted probability

Case Processing Summary

Unweighted Cases[a]		N	Percent
Selected Cases	Included in Analysis	115	97.5
	Missing Cases	3	2.5
	Total	118	100.0
Unselected Cases		0	.0
Total		118	100.0

a. If weight is in effect, see classification table for the total number of cases.

Dependent Variable Encoding

Original Value	Internal Value
No	0
Yes	1

Block 0: Beginning Block

Iteration History[a,b,c]

Iteration		-2 Log likelihood	Coefficients Constant
Step 0	1	156.271	.330
	2	156.270	.333
	3	156.270	.333

a. Constant is included in the model.

b. Initial -2 Log Likelihood: 156.270

c. Estimation terminated at iteration number 3 because parameter estimates changed by less than .001.

Classification Table[a,b]

	Observed		Predicted CS No	Predicted CS Yes	Predicted Percentage Correct
Step 0	CS	No	0	48	.0
		Yes	0	67	100.0
	Overall Percentage				58.3

a. Constant is included in the model.

b. The cut value is .500

Variables in the Equation

		B	S.E.	Wald	df	Sig.	Exp(B)
Step 0	Constant	.333	.189	3.110	1	.078	1.396

Variables not in the Equation

			Score	df	Sig.
Step 0	Variables	FollowupYN	22.737	1	.000
	Overall Statistics		22.737	1	.000

Block 1: Method = Enter

Iteration History[a,b,c,d]

Iteration		-2 Log likelihood	Coefficients	
			Constant	FollowupYN
Step 1	1	132.047	-.286	1.865
	2	130.777	-.288	2.330
	3	130.742	-.288	2.424
	4	130.742	-.288	2.428
	5	130.742	-.288	2.428

a. Method: Enter

b. Constant is included in the model.

c. Initial -2 Log Likelihood: 156.270

d. Estimation terminated at iteration number 5 because parameter estimates changed by less than .001.

Omnibus Tests of Model Coefficients

		Chi-square	df	Sig.
Step 1	Step	25.529	1	.000
	Block	25.529	1	.000
	Model	25.529	1	.000

Model Summary

Step	-2 Log likelihood	Cox & Snell R Square	Nagelkerke R Square
1	130.742[a]	.199	.268

a. Estimation terminated at iteration number 5 because parameter estimates changed by less than .001.

Hosmer and Lemeshow Test

Step	Chi-square	df	Sig.
1	.000	0	.

Contingency Table for Hosmer and Lemeshow Test

		CS = No		CS = Yes		
		Observed	Expected	Observed	Expected	Total
Step 1	1	44	44.000	33	33.000	77
	2	4	4.000	34	34.000	38

Classification Table[a]

Observed			Predicted CS No	Predicted CS Yes	Percentage Correct
Step 1	CS	No	44	4	91.7
		Yes	33	34	50.7
	Overall Percentage				67.8

a. The cut value is .500

Variables in the Equation

		B	S.E.	Wald	df	Sig.	Exp(B)	95% C.I.for EXP(B) Lower	95% C.I.for EXP(B) Upper
Step 1[a]	FollowupYN	2.428	.577	17.729	1	.000	11.333	3.661	35.087
	Constant	-.288	.230	1.561	1	.212	.750		

a. Variable(s) entered on step 1: FollowupYN.

```
         Step number: 1

         Observed Groups and Predicted Probabilities

      80 +
                                    +
         I                                        Y
                                    I
         I                                        Y
                                    I
F        I                                        Y
                                    I
R     60 +                                        Y
                                    +
E        I                                        Y
                                    I
```

```
Q        I                                                  Y
                                           I
U        I                                                  N
                                           I
E     40 +                                                  N
                        Y                  +
N        I                                                  N
                        Y                  I
C        I                                                  N
                        Y                  I
Y        I                                                  N
                        Y                  I
      20 +                                                  N
                        Y                  +
         I                                                  N
                        Y                  I
         I                                                  N
                        Y                  I
         I                                                  N
                        N                  I
Predicted ---------+---------+---------+---------+---------+---------+-----
----+---------+---------+----------
  Prob:   0        .1        .2        .3        .4        .5        .6
   .7        .8        .9         1
  Group:  NNNNNNNNNNNNNNNNNNNNNNNNNNNNNNNNNNNNNNNNNNNNNNNNNNNNNNNNNNYYYYYYYYYYYYYY
YYYYYYYYYYYYYYYYYYYYYYYYYYYYYYYYYYYY

          Predicted Probability is of Membership for Yes
          The Cut Value is .50
          Symbols: N - No
                   Y - Yes
          Each Symbol Represents 5 Cases.
```

Casewise List[b]

Case	Selected Status[a]	Observed CS	Predicted	Predicted Group	Temporary Variable Resid	Temporary Variable ZResid
7	S	N**	.895	Y	-.895	-2.915
8	S	N**	.895	Y	-.895	-2.915
13	S	N**	.895	Y	-.895	-2.915
24	S	N**	.895	Y	-.895	-2.915

a. S = Selected, U = Unselected cases, and ** = Misclassified cases.

b. Cases with studentized residuals greater than 2.000 are listed.

```
LOGISTIC REGRESSION VARIABLES Compensatory.sweating
 /METHOD=ENTER Age
 /CONTRAST (Age)=Indicator(1)
 /SAVE=PRED
 /CLASSPLOT
```

```
/CASEWISE OUTLIER(2)
/PRINT=GOODFIT ITER(1) CI(95)
/CRITERIA=PIN(0.05) POUT(0.10) ITERATE(20) CUT(0.5).
```

Logistic Regression

Notes

Output Created		18-APR-2018 22:03:...
Comments		
Input	Data	C:\Users\rnordin.ADMIN\Desktop\2018\PUBLICATION 2018 ETS\ETS.Data (Complete).sav 18APRIL2018.sav
	Active Dataset	DataSet1
	Filter	<none>
	Weight	<none>
	Split File	<none>
	N of Rows in Working Data File	118
Missing Value Handling	Definition of Missing	User-defined missing values are treated as missing
Syntax		LOGISTIC REGRESSION VARIABLES Compensatory.sweating /METHOD=ENTER Age /CONTRAST (Age)=Indicator(1) /SAVE=PRED /CLASSPLOT /CASEWISE OUTLIER(2) /PRINT=GOODFIT ITER(1) CI(95) /CRITERIA=PIN(0.05) POUT(0.10) ITERATE(20) CUT(0.5).
Resources	Processor Time	00:00:00.06
	Elapsed Time	00:00:00.05
Variables Created or Modified	PRE_7	Predicted probability

Case Processing Summary

Unweighted Cases[a]		N	Percent
Selected Cases	Included in Analysis	118	100.0
	Missing Cases	0	.0
	Total	118	100.0
Unselected Cases		0	.0
Total		118	100.0

a. If weight is in effect, see classification table for the total number of cases.

Dependent Variable Encoding

Original Value	Internal Value
No	0
Yes	1

Categorical Variables Codings

			Parameter coding																											
		Frequency	(1)	(2)	(3)	(4)	(5)	(6)	(7)	(8)	(9)	(10)	(11)	(12)	(13)	(14)	(15)	(16)	(17)	(18)	(19)	(20)	(21)	(22)	(23)	(24)	(25)	(26)	(27)	(28)
Age	9	1	.000	.000	.000	.000	.000	.000	.000	.000	.000	.000	.000	.000	.000	.000	.000	.000	.000	.000	.000	.000	.000	.000	.000	.000	.000	.000	.000	.000
	10	1	1.000	.000	.000	.000	.000	.000	.000	.000	.000	.000	.000	.000	.000	.000	.000	.000	.000	.000	.000	.000	.000	.000	.000	.000	.000	.000	.000	.000
	13	1	.000	1.000	.000	.000	.000	.000	.000	.000	.000	.000	.000	.000	.000	.000	.000	.000	.000	.000	.000	.000	.000	.000	.000	.000	.000	.000	.000	.000
	14	3	.000	.000	1.000	.000	.000	.000	.000	.000	.000	.000	.000	.000	.000	.000	.000	.000	.000	.000	.000	.000	.000	.000	.000	.000	.000	.000	.000	.000
	15	9	.000	.000	.000	1.000	.000	.000	.000	.000	.000	.000	.000	.000	.000	.000	.000	.000	.000	.000	.000	.000	.000	.000	.000	.000	.000	.000	.000	.000
	16	3	.000	.000	.000	.000	1.000	.000	.000	.000	.000	.000	.000	.000	.000	.000	.000	.000	.000	.000	.000	.000	.000	.000	.000	.000	.000	.000	.000	.000
	17	10	.000	.000	.000	.000	.000	1.000	.000	.000	.000	.000	.000	.000	.000	.000	.000	.000	.000	.000	.000	.000	.000	.000	.000	.000	.000	.000	.000	.000
	18	10	.000	.000	.000	.000	.000	.000	1.000	.000	.000	.000	.000	.000	.000	.000	.000	.000	.000	.000	.000	.000	.000	.000	.000	.000	.000	.000	.000	.000
	19	5	.000	.000	.000	.000	.000	.000	.000	1.000	.000	.000	.000	.000	.000	.000	.000	.000	.000	.000	.000	.000	.000	.000	.000	.000	.000	.000	.000	.000
	20	8	.000	.000	.000	.000	.000	.000	.000	.000	1.000	.000	.000	.000	.000	.000	.000	.000	.000	.000	.000	.000	.000	.000	.000	.000	.000	.000	.000	.000
	21	9	.000	.000	.000	.000	.000	.000	.000	.000	.000	1.000	.000	.000	.000	.000	.000	.000	.000	.000	.000	.000	.000	.000	.000	.000	.000	.000	.000	.000
	22	5	.000	.000	.000	.000	.000	.000	.000	.000	.000	.000	1.000	.000	.000	.000	.000	.000	.000	.000	.000	.000	.000	.000	.000	.000	.000	.000	.000	.000
	23	4	.000	.000	.000	.000	.000	.000	.000	.000	.000	.000	.000	1.000	.000	.000	.000	.000	.000	.000	.000	.000	.000	.000	.000	.000	.000	.000	.000	.000
	24	2	.000	.000	.000	.000	.000	.000	.000	.000	.000	.000	.000	.000	1.000	.000	.000	.000	.000	.000	.000	.000	.000	.000	.000	.000	.000	.000	.000	.000
	25	10	.000	.000	.000	.000	.000	.000	.000	.000	.000	.000	.000	.000	.000	1.000	.000	.000	.000	.000	.000	.000	.000	.000	.000	.000	.000	.000	.000	.000
	26	9	.000	.000	.000	.000	.000	.000	.000	.000	.000	.000	.000	.000	.000	.000	1.000	.000	.000	.000	.000	.000	.000	.000	.000	.000	.000	.000	.000	.000
	27	6	.000	.000	.000	.000	.000	.000	.000	.000	.000	.000	.000	.000	.000	.000	.000	1.000	.000	.000	.000	.000	.000	.000	.000	.000	.000	.000	.000	.000
	28	4	.000	.000	.000	.000	.000	.000	.000	.000	.000	.000	.000	.000	.000	.000	.000	.000	1.000	.000	.000	.000	.000	.000	.000	.000	.000	.000	.000	.000
	29	3	.000	.000	.000	.000	.000	.000	.000	.000	.000	.000	.000	.000	.000	.000	.000	.000	.000	1.000	.000	.000	.000	.000	.000	.000	.000	.000	.000	.000
	30	1	.000	.000	.000	.000	.000	.000	.000	.000	.000	.000	.000	.000	.000	.000	.000	.000	.000	.000	1.000	.000	.000	.000	.000	.000	.000	.000	.000	.000
	31	1	.000	.000	.000	.000	.000	.000	.000	.000	.000	.000	.000	.000	.000	.000	.000	.000	.000	.000	.000	1.000	.000	.000	.000	.000	.000	.000	.000	.000
	32	3	.000	.000	.000	.000	.000	.000	.000	.000	.000	.000	.000	.000	.000	.000	.000	.000	.000	.000	.000	.000	1.000	.000	.000	.000	.000	.000	.000	.000
	35	3	.000	.000	.000	.000	.000	.000	.000	.000	.000	.000	.000	.000	.000	.000	.000	.000	.000	.000	.000	.000	.000	1.000	.000	.000	.000	.000	.000	.000
	37	1	.000	.000	.000	.000	.000	.000	.000	.000	.000	.000	.000	.000	.000	.000	.000	.000	.000	.000	.000	.000	.000	.000	1.000	.000	.000	.000	.000	.000
	39	2	.000	.000	.000	.000	.000	.000	.000	.000	.000	.000	.000	.000	.000	.000	.000	.000	.000	.000	.000	.000	.000	.000	.000	1.000	.000	.000	.000	.000
	40	1	.000	.000	.000	.000	.000	.000	.000	.000	.000	.000	.000	.000	.000	.000	.000	.000	.000	.000	.000	.000	.000	.000	.000	.000	1.000	.000	.000	.000
	43	1	.000	.000	.000	.000	.000	.000	.000	.000	.000	.000	.000	.000	.000	.000	.000	.000	.000	.000	.000	.000	.000	.000	.000	.000	.000	1.000	.000	.000
	45	1	.000	.000	.000	.000	.000	.000	.000	.000	.000	.000	.000	.000	.000	.000	.000	.000	.000	.000	.000	.000	.000	.000	.000	.000	.000	.000	1.000	.000
	52	1	.000	.000	.000	.000	.000	.000	.000	.000	.000	.000	.000	.000	.000	.000	.000	.000	.000	.000	.000	.000	.000	.000	.000	.000	.000	.000	.000	1.000

Block 0: Beginning Block

Iteration History[a,b,c]

Iteration		-2 Log likelihood	Coefficients Constant
Step 0	1	160.826	.305
	2	160.826	.307
	3	160.826	.307

a. Constant is included in the model.

b. Initial -2 Log Likelihood: 160.826

c. Estimation terminated at iteration number 3 because parameter estimates changed by less than .001.

Classification Table[a,b]

Observed			Predicted CS No	Predicted CS Yes	Percentage Correct
Step 0	CS	No	0	50	.0
		Yes	0	68	100.0
	Overall Percentage				57.6

a. Constant is included in the model.

b. The cut value is .500

Variables in the Equation

		B	S.E.	Wald	df	Sig.	Exp(B)
Step 0	Constant	.307	.186	2.724	1	.099	1.360

Variables not in the Equation

			Score	df	Sig.
Step 0	Variables	Age	22.739	28	.746
		Age(1)	.742	1	.389
		Age(2)	1.372	1	.242
		Age(3)	.744	1	.388
		Age(4)	1.620	1	.203
		Age(5)	.744	1	.388
		Age(6)	.025	1	.874
		Age(7)	.260	1	.610
		Age(8)	.664	1	.415
		Age(9)	1.061	1	.303
		Age(10)	.326	1	.568
		Age(11)	.664	1	.415
		Age(12)	.099	1	.753
		Age(13)	2.767	1	.096
		Age(14)	.685	1	.408
		Age(15)	.017	1	.896
		Age(16)	.151	1	.698
		Age(17)	.512	1	.474
		Age(18)	.744	1	.388
		Age(19)	1.372	1	.242
		Age(20)	.742	1	.389
		Age(21)	.103	1	.748
		Age(22)	.103	1	.748
		Age(23)	1.372	1	.242
		Age(24)	1.496	1	.221

Variables not in the Equation

	Score	df	Sig.
Age(25)	.742	1	.389
Age(26)	.742	1	.389
Age(27)	1.372	1	.242
Age(28)	.742	1	.389
Overall Statistics	22.739	28	.746

Block 1: Method = Enter

Iteration History[a,b,c,d]

Iteration		-2 Log likelihood	Coefficients: Constant	Age(1)	Age(2)	Age(3)	Age(4)	Age(5)	Age(6)	Age(7)	Age(8)	Age(9)	Age(10)	Age(11)	Age(12)	Age(13)
Step 1	1	136.429	-2.000	4.000	.000	1.333	3.111	1.333	2.400	2.000	1.600	3.000	2.667	1.600	2.000	.000
	2	134.004	-3.135	6.271	.000	2.442	4.383	2.442	3.541	3.135	2.730	4.232	3.828	2.730	3.135	.000
	3	133.238	-4.179	8.358	.000	3.486	5.432	3.486	4.584	4.179	3.773	5.277	4.872	3.773	4.179	.000
	4	132.967	-5.194	10.388	.000	4.501	6.447	4.501	5.600	5.194	4.789	6.293	5.887	4.789	5.194	.000
	5	132.869	-6.200	12.399	.000	5.507	7.452	5.507	6.605	6.200	5.794	7.298	6.893	5.794	6.200	.000
	6	132.833	-7.202	14.403	.000	6.509	8.454	6.509	7.607	7.202	6.796	8.300	7.895	6.796	7.202	.000
	7	132.820	-8.202	16.405	.000	7.509	9.455	7.509	8.608	8.202	7.797	9.301	8.896	7.797	8.202	.000
	8	132.815	-9.203	18.405	.000	8.510	10.455	8.510	9.608	9.203	8.797	10.301	9.896	8.797	9.203	.000
	9	132.813	-10.203	20.406	.000	9.510	11.456	9.510	10.608	10.203	9.797	11.301	10.896	9.797	10.203	.000
	10	132.813	-11.203	22.406	.000	10.510	12.456	10.510	11.608	11.203	10.797	12.301	11.896	10.797	11.203	.000
	11	132.813	-12.203	24.406	.000	11.510	13.456	11.510	12.608	12.203	11.797	13.301	12.896	11.797	12.203	.000
	12	132.812	-13.203	26.406	.000	12.510	14.456	12.510	13.608	13.203	12.797	14.302	13.896	12.797	13.203	.000
	13	132.812	-14.203	28.406	.000	13.510	15.456	13.510	14.608	14.203	13.797	15.302	14.896	13.797	14.203	.000
	14	132.812	-15.203	30.406	.000	14.510	16.456	14.510	15.608	15.203	14.797	16.302	15.896	14.797	15.203	.000
	15	132.812	-16.203	32.406	.000	15.510	17.456	15.510	16.608	16.203	15.797	17.302	16.896	15.797	16.203	.000
	16	132.812	-17.203	34.406	.000	16.510	18.456	16.510	17.608	17.203	16.797	18.302	17.896	16.797	17.203	.000
	17	132.812	-18.203	36.406	.000	17.510	19.456	17.510	18.608	18.203	17.797	19.302	18.896	17.797	18.203	.000
	18	132.812	-19.203	38.406	.000	18.510	20.456	18.510	19.608	19.203	18.797	20.302	19.896	18.797	19.203	.000
	19	132.812	-20.203	40.406	.000	19.510	21.456	19.510	20.608	20.203	19.797	21.302	20.896	19.797	20.203	.000
	20	132.812	-21.203	42.406	.000	20.510	22.456	20.510	21.608	21.203	20.797	22.302	21.896	20.797	21.203	.000

Iteration		Age(14)	Age(15)	Age(16)	Age(17)	Age(18)	Age(19)	Age(20)	Age(21)	Age(22)	Age(23)	Age(24)	Age(25)	Age(26)	Age(27)	Age(28)
Step 1	1	2.800	2.222	2.000	3.000	1.333	.000	4.000	2.667	2.667	.000	4.000	4.000	4.000	.000	4.000
	2	3.982	3.358	3.135	4.232	2.442	.000	6.271	3.828	3.828	.000	6.271	6.271	6.271	.000	6.271
	3	5.026	4.402	4.179	5.277	3.486	.000	8.358	4.872	4.872	.000	8.358	8.358	8.358	.000	8.358
	4	6.041	5.417	5.194	6.293	4.501	.000	10.388	5.887	5.887	.000	10.388	10.388	10.388	.000	10.388
	5	7.047	6.423	6.200	7.298	5.507	.000	12.399	6.893	6.893	.000	12.399	12.399	12.399	.000	12.399
	6	8.049	7.425	7.202	8.300	6.509	.000	14.403	7.895	7.895	.000	14.403	14.403	14.403	.000	14.403
	7	9.050	8.426	8.202	9.301	7.509	.000	16.405	8.896	8.896	.000	16.405	16.405	16.405	.000	16.405
	8	10.050	9.426	9.203	10.301	8.510	.000	18.405	9.896	9.896	.000	18.405	18.405	18.405	.000	18.405
	9	11.050	10.426	10.203	11.301	9.510	.000	20.406	10.896	10.896	.000	20.406	20.406	20.406	.000	20.406
	10	12.050	11.426	11.203	12.301	10.510	.000	22.406	11.896	11.896	.000	22.406	22.406	22.406	.000	22.406
	11	13.050	12.426	12.203	13.301	11.510	.000	24.406	12.896	12.896	.000	24.406	24.406	24.406	.000	24.406
	12	14.050	13.426	13.203	14.302	12.510	.000	26.406	13.896	13.896	.000	26.406	26.406	26.406	.000	26.406
	13	15.050	14.426	14.203	15.302	13.510	.000	28.406	14.896	14.896	.000	28.406	28.406	28.406	.000	28.406
	14	16.050	15.426	15.203	16.302	14.510	.000	30.406	15.896	15.896	.000	30.406	30.406	30.406	.000	30.406
	15	17.050	16.426	16.203	17.302	15.510	.000	32.406	16.896	16.896	.000	32.406	32.406	32.406	.000	32.406
	16	18.050	17.426	17.203	18.302	16.510	.000	34.406	17.896	17.896	.000	34.406	34.406	34.406	.000	34.406
	17	19.050	18.426	18.203	19.302	17.510	.000	36.406	18.896	18.896	.000	36.406	36.406	36.406	.000	36.406
	18	20.050	19.426	19.203	20.302	18.510	.000	38.406	19.896	19.896	.000	38.406	38.406	38.406	.000	38.406
	19	21.050	20.426	20.203	21.302	19.510	.000	40.406	20.896	20.896	.000	40.406	40.406	40.406	.000	40.406
	20	22.050	21.426	21.203	22.302	20.510	.000	42.406	21.896	21.896	.000	42.406	42.406	42.406	.000	42.406

a. Method: Enter

b. Constant is included in the model.

c. Initial -2 Log Likelihood: 160.826

d. Estimation terminated at iteration number 20 because maximum iterations has been reached. Final solution cannot be found.

Omnibus Tests of Model Coefficients

		Chi-square	df	Sig.
Step 1	Step	28.014	28	.464
	Block	28.014	28	.464
	Model	28.014	28	.464

Model Summary

Step	-2 Log likelihood	Cox & Snell R Square	Nagelkerke R Square
1	132.812[a]	.211	.284

a. Estimation terminated at iteration number 20 because maximum iterations has been reached. Final solution cannot be found.

Hosmer and Lemeshow Test

Step	Chi-square	df	Sig.
1	.000	8	1.000

Contingency Table for Hosmer and Lemeshow Test

		CS = No		CS = Yes		
		Observed	Expected	Observed	Expected	Total
Step 1	1	13	13.000	3	3.000	16
	2	6	6.000	4	4.000	10
	3	10	10.000	10	10.000	20
	4	4	4.000	5	5.000	9
	5	4	4.000	6	6.000	10
	6	5	5.000	10	10.000	15
	7	3	3.000	7	7.000	10
	8	3	3.000	9	9.000	12
	9	2	2.000	7	7.000	9
	10	0	.000	7	7.000	7

Classification Table[a]

Observed			Predicted		
			CS		Percentage Correct
			No	Yes	
Step 1	CS	No	19	31	38.0
		Yes	7	61	89.7
	Overall Percentage				67.8

a. The cut value is .500

Variables in the Equation

		B	S.E.	Wald	df	Sig.	Exp(B)	95% C.I.for EXP(B)	
								Lower	Upper
Step 1[a]	Age			7.631	28	1.000			
	Age(1)	42.406	56842.191	.000	1	.999	2.610E+18	.000	.
	Age(2)	.000	56842.192	.000	1	1.000	1.000	.000	.
	Age(3)	20.510	40194.029	.000	1	1.000	807749668	.000	.
	Age(4)	22.456	40194.029	.000	1	1.000	5.654E+9	.000	.
	Age(5)	20.510	40194.029	.000	1	1.000	807749668	.000	.
	Age(6)	21.608	40194.029	.000	1	1.000	2.423E+9	.000	.
	Age(7)	21.203	40194.029	.000	1	1.000	1.615E+9	.000	.
	Age(8)	20.797	40194.029	.000	1	1.000	1.077E+9	.000	.
	Age(9)	22.302	40194.029	.000	1	1.000	4.846E+9	.000	.
	Age(10)	21.896	40194.029	.000	1	1.000	3.231E+9	.000	.
	Age(11)	20.797	40194.029	.000	1	1.000	1.077E+9	.000	.
	Age(12)	21.203	40194.029	.000	1	1.000	1.615E+9	.000	.
	Age(13)	.000	49226.998	.000	1	1.000	1.000	.000	.
	Age(14)	22.050	40194.029	.000	1	1.000	3.769E+9	.000	.
	Age(15)	21.426	40194.029	.000	1	1.000	2.019E+9	.000	.
	Age(16)	21.203	40194.029	.000	1	1.000	1.615E+9	.000	.
	Age(17)	22.302	40194.029	.000	1	1.000	4.846E+9	.000	.
	Age(18)	20.510	40194.029	.000	1	1.000	807749668	.000	.
	Age(19)	.000	56842.192	.000	1	1.000	1.000	.000	.
	Age(20)	42.406	56842.191	.000	1	.999	2.610E+18	.000	.
	Age(21)	21.896	40194.029	.000	1	1.000	3.231E+9	.000	.
	Age(22)	21.896	40194.029	.000	1	1.000	3.231E+9	.000	.
	Age(23)	.000	56842.192	.000	1	1.000	1.000	.000	.
	Age(24)	42.406	49226.998	.000	1	.999	2.610E+18	.000	.
	Age(25)	42.406	56842.191	.000	1	.999	2.610E+18	.000	.
	Age(26)	42.406	56842.191	.000	1	.999	2.610E+18	.000	.
	Age(27)	.000	56842.192	.000	1	1.000	1.000	.000	.
	Age(28)	42.406	56842.191	.000	1	.999	2.610E+18	.000	.
	Constant	-21.203	40194.029	.000	1	1.000	.000		

a. Variable(s) entered on step 1: Age.

```
Step number: 1

Observed Groups and Predicted Probabilities

    20 +                                                Y
                          +
       I                                                Y
                          I
       I                                                Y
                          I
F      I                                                Y
                          I
```

```
R      15 +                                                  Y
 Y                                  +
E        I                                                   Y
 Y                                  I
Q        I                                                   Y
 Y       Y                          I
U        I                                                   Y
 Y       Y                          I
E      10 +                                        Y         N         Y
 Y   Y   Y                          +
N        I                                  Y      Y         N    Y    Y
 Y   Y   Y  Y                       I
C        IN                                 Y      Y         N    Y    Y
 Y   Y   Y  Y                      YI
Y        IN                                 N      N         N    Y    Y
 Y   Y   Y  Y                      YI
        5 +N                                N      N         N    Y    Y
 N   Y   Y  Y                      Y+
         IN                                 N      N         N    N    N
 N   Y   Y  Y                      YI
         IN                                 N      N         N    N    N
 N   N   N  N                      YI
         IN                                 N      N         N    N    N
 N   N   N  N                      YI
Predicted ---------+---------+---------+---------+---------+---------+-----
----+---------+---------+----------
  Prob:   0       .1        .2        .3        .4        .5        .6
   .7        .8        .9         1
  Group:  NNNNNNNNNNNNNNNNNNNNNNNNNNNNNNNNNNNNNNNNNNNNNNNNNNNYYYYYYYYYYYYYYY
YYYYYYYYYYYYYYYYYYYYYYYYYYYYYYYYYYY

          Predicted Probability is of Membership for Yes
          The Cut Value is .50
          Symbols: N - No
                   Y - Yes
          Each Symbol Represents 1.25 Cases.
```

Casewise List[a]

a. The casewise plot is not produced because no outliers were found.

```
LOGISTIC REGRESSION VARIABLES Compensatory.sweating
 /METHOD=ENTER Age
 /SAVE=PRED
 /CLASSPLOT
```

```
/CASEWISE OUTLIER(2)
/PRINT=GOODFIT ITER(1) CI(95)
/CRITERIA=PIN(0.05) POUT(0.10) ITERATE(20) CUT(0.5).
```

Logistic Regression

Notes

Output Created		18-APR-2018 22:03:...
Comments		
Input	Data	C:\Users\rnordin.ADMIN\Desktop\2018\PUBLICATION 2018 ETS\ETS.Data (Complete).sav 18APRIL2018.sav
	Active Dataset	DataSet1
	Filter	<none>
	Weight	<none>
	Split File	<none>
	N of Rows in Working Data File	118
Missing Value Handling	Definition of Missing	User-defined missing values are treated as missing
Syntax		LOGISTIC REGRESSION VARIABLES Compensatory.sweating /METHOD=ENTER Age /SAVE=PRED /CLASSPLOT /CASEWISE OUTLIER(2) /PRINT=GOODFIT ITER (1) CI(95) /CRITERIA=PIN(0.05) POUT(0.10) ITERATE(20) CUT(0.5).
Resources	Processor Time	00:00:00.02
	Elapsed Time	00:00:00.01
Variables Created or Modified	PRE_8	Predicted probability

Case Processing Summary

Unweighted Cases[a]		N	Percent
Selected Cases	Included in Analysis	118	100.0
	Missing Cases	0	.0
	Total	118	100.0
Unselected Cases		0	.0
Total		118	100.0

a. If weight is in effect, see classification table for the total number of cases.

Dependent Variable Encoding

Original Value	Internal Value
No	0
Yes	1

Block 0: Beginning Block

Iteration History[a,b,c]

Iteration		-2 Log likelihood	Coefficients Constant
Step 0	1	160.826	.305
	2	160.826	.307
	3	160.826	.307

a. Constant is included in the model.

b. Initial -2 Log Likelihood: 160.826

c. Estimation terminated at iteration number 3 because parameter estimates changed by less than .001.

Classification Table[a,b]

Observed			Predicted CS No	Predicted CS Yes	Percentage Correct
Step 0	CS	No	0	50	.0
		Yes	0	68	100.0
	Overall Percentage				57.6

a. Constant is included in the model.

b. The cut value is .500

Variables in the Equation

		B	S.E.	Wald	df	Sig.	Exp(B)
Step 0	Constant	.307	.186	2.724	1	.099	1.360

Variables not in the Equation

			Score	df	Sig.
Step 0	Variables	Age	.611	1	.434
	Overall Statistics		.611	1	.434

Block 1: Method = Enter

Iteration History[a,b,c,d]

Iteration		-2 Log likelihood	Coefficients	
			Constant	Age
Step 1	1	160.209	-.145	.020
	2	160.207	-.162	.021
	3	160.207	-.162	.021

a. Method: Enter

b. Constant is included in the model.

c. Initial -2 Log Likelihood: 160.826

d. Estimation terminated at iteration number 3 because parameter estimates changed by less than .001.

Omnibus Tests of Model Coefficients

		Chi-square	df	Sig.
Step 1	Step	.619	1	.431
	Block	.619	1	.431
	Model	.619	1	.431

Model Summary

Step	-2 Log likelihood	Cox & Snell R Square	Nagelkerke R Square
1	160.207[a]	.005	.007

a. Estimation terminated at iteration number 3 because parameter estimates changed by less than .001.

Hosmer and Lemeshow Test

Step	Chi-square	df	Sig.
1	.931	7	.996

Contingency Table for Hosmer and Lemeshow Test

		CS = No		CS = Yes		
		Observed	Expected	Observed	Expected	Total
Step 1	1	6	7.033	9	7.967	15
	2	6	5.907	7	7.093	13
	3	5	4.481	5	5.519	10
	4	5	5.718	8	7.282	13
	5	6	6.035	8	7.965	14
	6	7	6.654	9	9.346	16
	7	7	6.087	8	8.913	15
	8	5	4.677	7	7.323	12
	9	3	3.409	7	6.591	10

Classification Table[a]

Observed			Predicted: CS No	Predicted: CS Yes	Percentage Correct
Step 1	CS	No	0	50	.0
		Yes	0	68	100.0
	Overall Percentage				57.6

a. The cut value is .500

Variables in the Equation

		B	S.E.	Wald	df	Sig.	Exp(B)	95% C.I.for EXP(B) Lower	Upper
Step 1[a]	Age	.021	.026	.606	1	.436	1.021	.969	1.075
	Constant	-.162	.629	.066	1	.797	.850		

a. Variable(s) entered on step 1: Age.

```
            Step number: 1

            Observed Groups and Predicted Probabilities

    20 +
                          +
       I
                          I
       I                                Y
                          I
F      I                                Y
                          I
```

```
R     15 +                                                     YY  Y
                                    +
E        I                                                     YY  Y
                                    I
Q        I                                                   YYYY YY
                                    I
U        I                                                   YYYY YY
                                    I
E     10 +                                                   YYYY YY
                                    +
N        I                                                   YYYYYYY
                                    I
C        I                                                   YYNYYYNY
                                    I
Y        I                                                   YNNYYYNY
                                    I
       5 +                                                   YNNNNNNY
                                    +
         I                                                   NNNNNNNY
                                    I
         I                                                   NNNNNNNNYYY
Y                                   I
         I                                                NYNNNNNNNNNNNNN
Y YN  Y                             I
Predicted ---------+---------+---------+---------+---------+---------+-----
----+---------+---------+----------
  Prob:   0       .1        .2        .3        .4        .5        .6
   .7        .8        .9        1
  Group:  NNNNNNNNNNNNNNNNNNNNNNNNNNNNNNNNNNNNNNNNNNNNNNNNNNNYYYYYYYYYYYYYY
YYYYYYYYYYYYYYYYYYYYYYYYYYYYYYYYYYY

          Predicted Probability is of Membership for Yes
          The Cut Value is .50
          Symbols: N - No
                   Y - Yes
          Each Symbol Represents 1.25 Cases.
```

Casewise List[a]

a. The casewise plot is not produced because no outliers were found.

```
LOGISTIC REGRESSION VARIABLES Compensatory.sweating
  /METHOD=ENTER Sex
  /CONTRAST (Sex)=Indicator(1)
  /SAVE=PRED
```

```
/CLASSPLOT
/CASEWISE OUTLIER(2)
/PRINT=GOODFIT ITER(1) CI(95)
/CRITERIA=PIN(0.05) POUT(0.10) ITERATE(20) CUT(0.5).
```

Logistic Regression

Notes

Output Created		18-APR-2018 22:04:...
Comments		
Input	Data	C:\Users\rnordin.ADMIN\Desktop\2018\PUBLICATION 2018 ETS\ETS.Data (Complete).sav 18APRIL2018.sav
	Active Dataset	DataSet1
	Filter	<none>
	Weight	<none>
	Split File	<none>
	N of Rows in Working Data File	118
Missing Value Handling	Definition of Missing	User-defined missing values are treated as missing
Syntax		LOGISTIC REGRESSION VARIABLES Compensatory.sweating /METHOD=ENTER Sex /CONTRAST (Sex)=Indicator(1) /SAVE=PRED /CLASSPLOT /CASEWISE OUTLIER(2) /PRINT=GOODFIT ITER(1) CI(95) /CRITERIA=PIN(0.05) POUT(0.10) ITERATE(20) CUT(0.5).
Resources	Processor Time	00:00:00.03
	Elapsed Time	00:00:00.03
Variables Created or Modified	PRE_9	Predicted probability

Case Processing Summary

Unweighted Cases[a]		N	Percent
Selected Cases	Included in Analysis	118	100.0
	Missing Cases	0	.0
	Total	118	100.0
Unselected Cases		0	.0
Total		118	100.0

a. If weight is in effect, see classification table for the total number of cases.

Dependent Variable Encoding

Original Value	Internal Value
No	0
Yes	1

Categorical Variables Codings

		Frequency	Parameter coding (1)
Sex	Male	50	.000
	Female	68	1.000

Block 0: Beginning Block

Iteration History[a,b,c]

Iteration		-2 Log likelihood	Coefficients Constant
Step 0	1	160.826	.305
	2	160.826	.307
	3	160.826	.307

a. Constant is included in the model.

b. Initial -2 Log Likelihood: 160.826

c. Estimation terminated at iteration number 3 because parameter estimates changed by less than .001.

Classification Table[a,b]

Observed			Predicted CS No	Predicted CS Yes	Percentage Correct
Step 0	CS	No	0	50	.0
		Yes	0	68	100.0
	Overall Percentage				57.6

a. Constant is included in the model.

b. The cut value is .500

Variables in the Equation

		B	S.E.	Wald	df	Sig.	Exp(B)
Step 0	Constant	.307	.186	2.724	1	.099	1.360

Variables not in the Equation

			Score	df	Sig.
Step 0	Variables	Sex(1)	1.125	1	.289
	Overall Statistics		1.125	1	.289

Block 1: Method = Enter

Iteration History[a,b,c,d]

Iteration		-2 Log likelihood	Coefficients Constant	Coefficients Sex(1)
Step 1	1	159.704	.080	.391
	2	159.702	.080	.400
	3	159.702	.080	.400

a. Method: Enter

b. Constant is included in the model.

c. Initial -2 Log Likelihood: 160.826

d. Estimation terminated at iteration number 3 because parameter estimates changed by less than .001.

Omnibus Tests of Model Coefficients

		Chi-square	df	Sig.
Step 1	Step	1.124	1	.289
	Block	1.124	1	.289
	Model	1.124	1	.289

Model Summary

Step	-2 Log likelihood	Cox & Snell R Square	Nagelkerke R Square
1	159.702[a]	.009	.013

a. Estimation terminated at iteration number 3 because parameter estimates changed by less than .001.

Hosmer and Lemeshow Test

Step	Chi-square	df	Sig.
1	.000	0	.

Contingency Table for Hosmer and Lemeshow Test

		CS = No		CS = Yes		
		Observed	Expected	Observed	Expected	Total
Step 1	1	24	24.000	26	26.000	50
	2	26	26.000	42	42.000	68

Classification Table[a]

Observed			Predicted CS No	Predicted CS Yes	Percentage Correct
Step 1	CS	No	0	50	.0
		Yes	0	68	100.0
	Overall Percentage				57.6

a. The cut value is .500

Variables in the Equation

		B	S.E.	Wald	df	Sig.	Exp(B)	95% C.I.for EXP(B) Lower	95% C.I.for EXP(B) Upper
Step 1[a]	Sex(1)	.400	.377	1.121	1	.290	1.491	.712	3.124
	Constant	.080	.283	.080	1	.777	1.083		

a. Variable(s) entered on step 1: Sex.

```
            Step number: 1

            Observed Groups and Predicted Probabilities

     80 +
                                       +
```

```
         I
                                    I
         I                                                          Y
                                    I
F        I                                                          Y
                                    I
R     60 +                                                          Y
                                    +
E        I                                                          Y
                                    I
Q        I                                                Y         Y
                                    I
U        I                                                Y         Y
                                    I
E     40 +                                                Y         Y
                                    +
N        I                                                Y         Y
                                    I
C        I                                                Y         Y
                                    I
Y        I                                                N         N
                                    I
      20 +                                                N         N
                                    +
         I                                                N         N
                                    I
         I                                                N         N
                                    I
         I                                                N         N
                                    I
Predicted ---------+---------+---------+---------+---------+---------+-----
----+---------+---------+----------
  Prob:   0        .1        .2        .3        .4        .5        .6
   .7        .8        .9         1
  Group:  NNNNNNNNNNNNNNNNNNNNNNNNNNNNNNNNNNNNNNNNNNNNNNNNNNYYYYYYYYYYYYYY
YYYYYYYYYYYYYYYYYYYYYYYYYYYYYYYYYYY

          Predicted Probability is of Membership for Yes
          The Cut Value is .50
          Symbols: N - No
                   Y - Yes
          Each Symbol Represents 5 Cases.
```

Casewise List[a]

a. The casewise plot is not produced because no outliers were found.

```
LOGISTIC REGRESSION VARIABLES Compensatory.sweating
 /METHOD=ENTER Race
 /CONTRAST (Race)=Indicator(1)
 /SAVE=PRED
 /CLASSPLOT
 /CASEWISE OUTLIER(2)
 /PRINT=GOODFIT ITER(1) CI(95)
 /CRITERIA=PIN(0.05) POUT(0.10) ITERATE(20) CUT(0.5).
```

Logistic Regression

Notes

Output Created		18-APR-2018 22:04:...
Comments		
Input	Data	C:\Users\rnordin.ADMIN\Desktop\2018\PUBLICATION 2018 ETS\ETS.Data (Complete).sav 18APRIL2018.sav
	Active Dataset	DataSet1
	Filter	<none>
	Weight	<none>
	Split File	<none>
	N of Rows in Working Data File	118
Missing Value Handling	Definition of Missing	User-defined missing values are treated as missing
Syntax		LOGISTIC REGRESSION VARIABLES Compensatory.sweating<br>/METHOD=ENTER Race<br>/CONTRAST (Race)=Indicator(1)<br>/SAVE=PRED<br>/CLASSPLOT<br>/CASEWISE OUTLIER(2)<br>/PRINT=GOODFIT ITER(1) CI(95)<br>/CRITERIA=PIN(0.05) POUT(0.10) ITERATE(20) CUT(0.5).
Resources	Processor Time	00:00:00.03
	Elapsed Time	00:00:00.04
Variables Created or Modified	PRE_10	Predicted probability

Case Processing Summary

Unweighted Cases[a]		N	Percent
Selected Cases	Included in Analysis	118	100.0
	Missing Cases	0	.0
	Total	118	100.0
Unselected Cases		0	.0
Total		118	100.0

a. If weight is in effect, see classification table for the total number of cases.

Dependent Variable Encoding

Original Value	Internal Value
No	0
Yes	1

Categorical Variables Codings

		Frequency	Parameter coding (1)	(2)
Race	Malay	94	.000	.000
	Chinese	16	1.000	.000
	Indian	8	.000	1.000

Block 0: Beginning Block

Iteration History[a,b,c]

Iteration		-2 Log likelihood	Coefficients Constant
Step 0	1	160.826	.305
	2	160.826	.307
	3	160.826	.307

a. Constant is included in the model.

b. Initial -2 Log Likelihood: 160.826

c. Estimation terminated at iteration number 3 because parameter estimates changed by less than .001.

Classification Table[a,b]

Observed			Predicted CS No	Predicted CS Yes	Percentage Correct
Step 0	CS	No	0	50	.0
		Yes	0	68	100.0
	Overall Percentage				57.6

a. Constant is included in the model.

b. The cut value is .500

Variables in the Equation

		B	S.E.	Wald	df	Sig.	Exp(B)
Step 0	Constant	.307	.186	2.724	1	.099	1.360

Variables not in the Equation

			Score	df	Sig.
Step 0	Variables	Race	1.061	2	.588
		Race(1)	.014	1	.905
		Race(2)	1.061	1	.303
	Overall Statistics		1.061	2	.588

Block 1: Method = Enter

Iteration History[a,b,c,d]

Iteration		-2 Log likelihood	Coefficients Constant	Coefficients Race(1)	Coefficients Race(2)
Step 1	1	159.718	.255	-.005	.745
	2	159.703	.257	-.005	.840
	3	159.703	.257	-.005	.842
	4	159.703	.257	-.005	.842

a. Method: Enter

b. Constant is included in the model.

c. Initial -2 Log Likelihood: 160.826

d. Estimation terminated at iteration number 4 because parameter estimates changed by less than .001.

Omnibus Tests of Model Coefficients

		Chi-square	df	Sig.
Step 1	Step	1.123	2	.570
	Block	1.123	2	.570
	Model	1.123	2	.570

Model Summary

Step	-2 Log likelihood	Cox & Snell R Square	Nagelkerke R Square
1	159.703[a]	.009	.013

a. Estimation terminated at iteration number 4 because parameter estimates changed by less than .001.

Hosmer and Lemeshow Test

Step	Chi-square	df	Sig.
1	.000	1	1.000

Contingency Table for Hosmer and Lemeshow Test

		CS = No		CS = Yes		
		Observed	Expected	Observed	Expected	Total
Step 1	1	7	7.000	9	9.000	16
	2	41	41.000	53	53.000	94
	3	2	2.000	6	6.000	8

Classification Table[a]

Observed			Predicted		
			CS		Percentage Correct
			No	Yes	
Step 1	CS	No	0	50	.0
		Yes	0	68	100.0
	Overall Percentage				57.6

a. The cut value is .500

Variables in the Equation

		B	S.E.	Wald	df	Sig.	Exp(B)	95% C.I.for EXP(B) Lower	Upper
Step 1[a]	Race			1.009	2	.604			
	Race(1)	-.005	.545	.000	1	.992	.995	.342	2.895
	Race(2)	.842	.843	.998	1	.318	2.321	.445	12.101
	Constant	.257	.208	1.524	1	.217	1.293		

a. Variable(s) entered on step 1: Race.

```
            Step number: 1

            Observed Groups and Predicted Probabilities

     160 +
                                     +
         I
                                     I
         I
                                     I
F        I
                                     I
R    120 +
                                     +
E        I                                                      Y
                                     I
Q        I                                                      Y
                                     I
U        I                                                      Y
                                     I
E     80 +                                                      Y
                                     +
N        I                                                      Y
                                     I
C        I                                                      Y
                                     I
Y        I                                                      N
                                     I
      40 +                                                      N
                                     +
         I                                                      N
                                     I
         I                                                      N
                                     I
         I                                                      N
         Y                           I
Predicted ---------+---------+---------+---------+---------+---------+-----
----+---------+---------+----------
```

```
  Prob:   0       .1        .2        .3        .4        .5        .6
   .7        .8        .9        1
  Group:  NNNNNNNNNNNNNNNNNNNNNNNNNNNNNNNNNNNNNNNNNNNNNNNNNNNNYYYYYYYYYYYYYYY
YYYYYYYYYYYYYYYYYYYYYYYYYYYYYYYYYYY

          Predicted Probability is of Membership for Yes
          The Cut Value is .50
          Symbols: N - No
                   Y - Yes
          Each Symbol Represents 10 Cases.
```

Casewise List[a]

a. The casewise plot is not produced because no outliers were found.

```
LOGISTIC REGRESSION VARIABLES Compensatory.sweating
  /METHOD=FSTEP(LR) Sympathectomy.Level FollowupYN
  /CONTRAST (Sympathectomy.Level)=Indicator(1)
  /CONTRAST (FollowupYN)=Indicator(1)
  /SAVE=PRED
  /CLASSPLOT
  /CASEWISE OUTLIER(2)
  /PRINT=GOODFIT ITER(1) CI(95)
  /CRITERIA=PIN(0.05) POUT(0.10) ITERATE(20) CUT(0.5).
```

Logistic Regression

Notes

Output Created		18-APR-2018 22:06:...
Comments		
Input	Data	C:\Users\rnordin.ADMIN\Desktop\2018\PUBLICATION 2018 ETS\ETS.Data (Complete).sav 18APRIL2018.sav
	Active Dataset	DataSet1
	Filter	<none>
	Weight	<none>
	Split File	<none>
	N of Rows in Working Data File	118
Missing Value Handling	Definition of Missing	User-defined missing values are treated as missing
Syntax		LOGISTIC REGRESSION VARIABLES Compensatory.sweating /METHOD=FSTEP(LR) Sympathectomy.Level FollowupYN /CONTRAST (Sympathectomy.Level)=Indicator(1) /CONTRAST (FollowupYN)=Indicator(1) /SAVE=PRED /CLASSPLOT /CASEWISE OUTLIER(2) /PRINT=GOODFIT ITER(1) CI(95) /CRITERIA=PIN(0.05) POUT(0.10) ITERATE(20) CUT(0.5).
Resources	Processor Time	00:00:00.03
	Elapsed Time	00:00:00.03
Variables Created or Modified	PRE_11	Predicted probability

Case Processing Summary

Unweighted Cases[a]		N	Percent
Selected Cases	Included in Analysis	115	97.5
	Missing Cases	3	2.5
	Total	118	100.0
Unselected Cases		0	.0
Total		118	100.0

a. If weight is in effect, see classification table for the total number of cases.

Dependent Variable Encoding

Original Value	Internal Value
No	0
Yes	1

Categorical Variables Codings

		Frequency	Parameter coding (1)
FollowupYN	One	77	.000
	More than one	38	1.000
Sympathectomy.Level	T2-T4	48	.000
	T2-T3	67	1.000

Block 0: Beginning Block

Iteration History[a,b,c]

Iteration		-2 Log likelihood	Coefficients Constant
Step 0	1	156.271	.330
	2	156.270	.333
	3	156.270	.333

a. Constant is included in the model.

b. Initial -2 Log Likelihood: 156.270

c. Estimation terminated at iteration number 3 because parameter estimates changed by less than .001.

Classification Table[a,b]

Observed			Predicted CS No	Predicted CS Yes	Percentage Correct
Step 0	CS	No	0	48	.0
		Yes	0	67	100.0
	Overall Percentage				58.3

a. Constant is included in the model.

b. The cut value is .500

Variables in the Equation

		B	S.E.	Wald	df	Sig.	Exp(B)
Step 0	Constant	.333	.189	3.110	1	.078	1.396

Variables not in the Equation

			Score	df	Sig.
Step 0	Variables	Sympathectomy.Level(1)	3.625	1	.057
		FollowupYN(1)	22.737	1	.000
	Overall Statistics		27.187	2	.000

Block 1: Method = Forward Stepwise (Likelihood Ratio)

Iteration History[a,b,c,d]

Iteration		-2 Log likelihood	Coefficients Constant	Coefficients FollowupYN(1)	Coefficients Sympathectomy.Level(1)
Step 1	1	132.047	-.286	1.865	
	2	130.777	-.288	2.330	
	3	130.742	-.288	2.424	
	4	130.742	-.288	2.428	
	5	130.742	-.288	2.428	
Step 2	1	127.007	-.756	1.900	.788
	2	125.119	-.916	2.470	1.018
	3	125.057	-.940	2.601	1.053
	4	125.057	-.941	2.607	1.054
	5	125.057	-.941	2.607	1.054

a. Method: Forward Stepwise (Likelihood Ratio)

b. Constant is included in the model.

c. Initial -2 Log Likelihood: 156.270

d. Estimation terminated at iteration number 5 because parameter estimates changed by less than .001.

Omnibus Tests of Model Coefficients

		Chi-square	df	Sig.
Step 1	Step	25.529	1	.000
	Block	25.529	1	.000
	Model	25.529	1	.000
Step 2	Step	5.685	1	.017
	Block	31.213	2	.000
	Model	31.213	2	.000

Model Summary

Step	-2 Log likelihood	Cox & Snell R Square	Nagelkerke R Square
1	130.742[a]	.199	.268
2	125.057[a]	.238	.320

a. Estimation terminated at iteration number 5 because parameter estimates changed by less than .001.

Hosmer and Lemeshow Test

Step	Chi-square	df	Sig.
1	.000	0	.
2	.134	2	.935

Contingency Table for Hosmer and Lemeshow Test

		CS = No		CS = Yes		
		Observed	Expected	Observed	Expected	Total
Step 1	1	44	44.000	33	33.000	77
	2	4	4.000	34	34.000	38
Step 2	1	22	22.298	9	8.702	31
	2	22	21.702	24	24.298	46
	3	3	2.702	14	14.298	17
	4	1	1.298	20	19.702	21

Classification Table[a]

Observed			Predicted CS No	Predicted CS Yes	Percentage Correct
Step 1	CS	No	44	4	91.7
		Yes	33	34	50.7
	Overall Percentage				67.8
Step 2	CS	No	22	26	45.8
		Yes	9	58	86.6
	Overall Percentage				69.6

a. The cut value is .500

Variables in the Equation

		B	S.E.	Wald	df	Sig.	Exp(B)	95% C.I.for EXP(B) Lower	95% C.I.for EXP(B) Upper
Step 1[a]	FollowupYN(1)	2.428	.577	17.729	1	.000	11.333	3.661	35.087
	Constant	-.288	.230	1.561	1	.212	.750		
Step 2[b]	Sympathectomy.Level(1)	1.054	.454	5.378	1	.020	2.869	1.177	6.991
	FollowupYN(1)	2.607	.601	18.813	1	.000	13.558	4.174	44.038
	Constant	-.941	.378	6.198	1	.013	.390		

a. Variable(s) entered on step 1: FollowupYN.

b. Variable(s) entered on step 2: Sympathectomy.Level.

Model if Term Removed

Variable		Model Log Likelihood	Change in -2 Log Likelihood	df	Sig. of the Change
Step 1	FollowupYN	-78.135	25.529	1	.000
Step 2	Sympathectomy.Level	-65.371	5.685	1	.017
	FollowupYN	-76.323	27.589	1	.000

Variables not in the Equation

			Score	df	Sig.
Step 1	Variables	Sympathectomy.Level(1)	5.573	1	.018
	Overall Statistics		5.573	1	.018

```
Step number: 1

Observed Groups and Predicted Probabilities

80 +
                         +
```

```
         I                                          Y
                                   I
         I                                          Y
                                   I
F        I                                          Y
                                   I
R     60 +                                          Y
                                   +
E        I                                          Y
                                   I
Q        I                                          Y
                                   I
U        I                                          N
                                   I
E     40 +                                          N
                        Y          +
N        I                                          N
                        Y          I
C        I                                          N
                        Y          I
Y        I                                          N
                        Y          I
      20 +                                          N
                        Y          +
         I                                          N
                        Y          I
         I                                          N
                        Y          I
         I                                          N
                        N          I
Predicted ---------+---------+---------+---------+---------+---------+-----
----+---------+---------+----------
  Prob:   0        .1        .2        .3        .4        .5        .6
   .7        .8        .9         1
  Group:  NNNNNNNNNNNNNNNNNNNNNNNNNNNNNNNNNNNNNNNNNNNNNNNNNNNNNNYYYYYYYYYYYYYY
YYYYYYYYYYYYYYYYYYYYYYYYYYYYYYYYYYY

          Predicted Probability is of Membership for Yes
          The Cut Value is .50
          Symbols: N - No
                   Y - Yes
          Each Symbol Represents 5 Cases.




             Step number: 2

             Observed Groups and Predicted Probabilities

      80 +
                                   +
         I
                                   I
```

```
         I
                                                  I
F        I
                                                  I
R     60 +
                                                  +
E        I
                                                  I
Q        I
                                                  I
U        I                                                   Y
                                                  I
E     40 +                                                   Y
                                                  +
N        I                                                   Y
                                                  I
C        I                                           Y                       Y
                                                  I
Y        I                                           Y                       Y
                                                  I
      20 +                                           N                       N
                                          Y       +
         I                                           N                       N
                                 Y        Y       I
         I                                           N                       N
                                 Y        Y       I
         I                                           N                       N
                                 N        Y       I
Predicted ---------+---------+---------+---------+---------+---------+-----
----+---------+---------+----------
  Prob:   0        .1        .2        .3        .4        .5        .6
   .7        .8        .9        1
  Group:  NNNNNNNNNNNNNNNNNNNNNNNNNNNNNNNNNNNNNNNNNNNNNNNNNNNNNNNYYYYYYYYYYYYYYY
YYYYYYYYYYYYYYYYYYYYYYYYYYYYYYYYYYY

          Predicted Probability is of Membership for Yes
          The Cut Value is .50
          Symbols: N - No
                   Y - Yes
          Each Symbol Represents 5 Cases.
```

Casewise List[b]

Case	Selected Status[a]	Observed CS	Predicted	Predicted Group	Temporary Variable Resid	Temporary Variable ZResid
24	S	N**	.938	Y	-.938	-3.896

a. S = Selected, U = Unselected cases, and ** = Misclassified cases.

b. Cases with studentized residuals greater than 2.000 are listed.

LOGISTIC REGRESSION VARIABLES Compensatory.sweating

```
/METHOD=ENTER Sympathectomy.LevelFollowupYN
/CONTRAST (Sympathectomy.Level)=Indicator(1)
/CONTRAST (FollowupYN)=Indicator(1)
/SAVE=PRED
/CLASSPLOT
/CASEWISE OUTLIER(2)
/PRINT=GOODFIT ITER(1) CI(95)
/CRITERIA=PIN(0.05) POUT(0.10) ITERATE(20) CUT(0.5).
```

Logistic Regression

Notes

Output Created		18-APR-2018 22:22:...
Comments		
Input	Data	C:\Users\rnordin.ADMIN\Desktop\2018\PUBLICATION 2018 ETS\ETS.Data (Complete).sav 18APRIL2018.sav
	Active Dataset	DataSet1
	Filter	<none>
	Weight	<none>
	Split File	<none>
	N of Rows in Working Data File	118
Missing Value Handling	Definition of Missing	User-defined missing values are treated as missing
Syntax		LOGISTIC REGRESSION VARIABLES Compensatory.sweating /METHOD=ENTER Sympathectomy.Level FollowupYN /CONTRAST (Sympathectomy.Level)=Indicator(1) /CONTRAST (FollowupYN)=Indicator(1) /SAVE=PRED /CLASSPLOT /CASEWISE OUTLIER(2) /PRINT=GOODFIT ITER(1) CI(95) /CRITERIA=PIN(0.05) POUT(0.10) ITERATE(20) CUT(0.5).
Resources	Processor Time	00:00:00.02
	Elapsed Time	00:00:00.01
Variables Created or Modified	PRE_12	Predicted probability

Case Processing Summary

Unweighted Cases[a]		N	Percent
Selected Cases	Included in Analysis	115	97.5
	Missing Cases	3	2.5
	Total	118	100.0
Unselected Cases		0	.0
Total		118	100.0

a. If weight is in effect, see classification table for the total number of cases.

Dependent Variable Encoding

Original Value	Internal Value
No	0
Yes	1

Categorical Variables Codings

		Frequency	Parameter coding (1)
FollowupYN	One	77	.000
	More than one	38	1.000
Sympathectomy.Level	T2-T4	48	.000
	T2-T3	67	1.000

Block 0: Beginning Block

Iteration History[a,b,c]

Iteration		-2 Log likelihood	Coefficients Constant
Step 0	1	156.271	.330
	2	156.270	.333
	3	156.270	.333

a. Constant is included in the model.

b. Initial -2 Log Likelihood: 156.270

c. Estimation terminated at iteration number 3 because parameter estimates changed by less than .001.

Classification Table[a,b]

Observed			Predicted CS No	Predicted CS Yes	Percentage Correct
Step 0	CS	No	0	48	.0
		Yes	0	67	100.0
	Overall Percentage				58.3

a. Constant is included in the model.

b. The cut value is .500

Variables in the Equation

		B	S.E.	Wald	df	Sig.	Exp(B)
Step 0	Constant	.333	.189	3.110	1	.078	1.396

Variables not in the Equation

			Score	df	Sig.
Step 0	Variables	Sympathectomy.Level(1)	3.625	1	.057
		FollowupYN(1)	22.737	1	.000
	Overall Statistics		27.187	2	.000

Block 1: Method = Enter

Iteration History[a,b,c,d]

Iteration		-2 Log likelihood	Coefficients Constant	Coefficients Sympathectomy.Level(1)	Coefficients FollowupYN(1)
Step 1	1	127.007	-.756	.788	1.900
	2	125.119	-.916	1.018	2.470
	3	125.057	-.940	1.053	2.601
	4	125.057	-.941	1.054	2.607
	5	125.057	-.941	1.054	2.607

a. Method: Enter

b. Constant is included in the model.

c. Initial -2 Log Likelihood: 156.270

d. Estimation terminated at iteration number 5 because parameter estimates changed by less than .001.

Omnibus Tests of Model Coefficients

		Chi-square	df	Sig.
Step 1	Step	31.213	2	.000
	Block	31.213	2	.000
	Model	31.213	2	.000

Model Summary

Step	-2 Log likelihood	Cox & Snell R Square	Nagelkerke R Square
1	125.057[a]	.238	.320

a. Estimation terminated at iteration number 5 because parameter estimates changed by less than .001.

Hosmer and Lemeshow Test

Step	Chi-square	df	Sig.
1	.134	2	.935

Contingency Table for Hosmer and Lemeshow Test

		CS = No		CS = Yes		
		Observed	Expected	Observed	Expected	Total
Step 1	1	22	22.298	9	8.702	31
	2	22	21.702	24	24.298	46
	3	3	2.702	14	14.298	17
	4	1	1.298	20	19.702	21

Classification Table[a]

Observed			Predicted: CS No	Predicted: CS Yes	Percentage Correct
Step 1	CS	No	22	26	45.8
		Yes	9	58	86.6
	Overall Percentage				69.6

a. The cut value is .500

Variables in the Equation

		B	S.E.	Wald	df	Sig.	Exp(B)	95% C.I.for EXP(B)	
								Lower	Upper
Step 1[a]	Sympathectomy.Level(1)	1.054	.454	5.378	1	.020	2.869	1.177	6.991
	FollowupYN(1)	2.607	.601	18.813	1	.000	13.558	4.174	44.038
	Constant	-.941	.378	6.198	1	.013	.390		

a. Variable(s) entered on step 1: Sympathectomy.Level, FollowupYN.

```
             Step number: 1

             Observed Groups and Predicted Probabilities

      80 +
                                   +
         I
                                   I
         I
                                   I
F        I
                                   I
R     60 +
                                   +
E        I
                                   I
Q        I
                                   I
U        I                                                    Y
                                   I
E     40 +                                                    Y
                                   +
N        I                                                    Y
                                   I
C        I                            Y                       Y
                                   I
Y        I                            Y                       Y
                                   I
      20 +                            N                       N
                            Y      +
         I                            N                       N
                   Y        Y      I
         I                            N                       N
                   Y        Y      I
         I                            N                       N
                   N        Y      I
Predicted ---------+---------+---------+---------+---------+---------+-----
----+---------+---------+----------
  Prob:   0       .1        .2        .3        .4        .5        .6
   .7        .8        .9         1
```

```
  Group:  NNNNNNNNNNNNNNNNNNNNNNNNNNNNNNNNNNNNNNNNNNNNNNNNNNNNNNYYYYYYYYYYYYY
YYYYYYYYYYYYYYYYYYYYYYYYYYYYYYYYYYY

          Predicted Probability is of Membership for Yes
          The Cut Value is .50
          Symbols: N - No
                   Y - Yes
          Each Symbol Represents 5 Cases.
```

Casewise List[b]

Case	Selected Status[a]	Observed CS	Predicted	Predicted Group	Temporary Variable Resid	Temporary Variable ZResid
24	S	N**	.938	Y	-.938	-3.896

a. S = Selected, U = Unselected cases, and ** = Misclassified cases.

b. Cases with studentized residuals greater than 2.000 are listed.

```
 DATASET ACTIVATE DataSet1.

SAVE OUTFILE='C:\Users\rnordin.ADMIN\Desktop\2018\PUBLICATION 2018 ETS\ETS.
Data(Complete).sav '+
    '18APRIL2018.sav'
  /COMPRESSED.



 GET
  FILE='C:\Users\rnordin.ADMIN\Desktop\2018\PUBLICATION 2018 ETS\ETS.Data(C
omplete).sav 18APRIL2018.sav'.
DATASET NAME DataSet1 WINDOW=FRONT.
SORT CASES BY BMI (A).
DATASET ACTIVATE DataSet1.

SAVE OUTFILE='C:\Users\rnordin.ADMIN\Desktop\2018\PUBLICATION 2018 ETS\ETS.
Data(Complete).sav '+
    '18APRIL2018.sav'
  /COMPRESSED.
STRING BMI.UNOO (A8).
RECODE BMI (Lowest thru 18.4='0') (18.5 thru 24='1') (25 thru 29.9='2') (30
 thru Highest='3') INTO
    BMI.UNOO.
VARIABLE LABELS  BMI.UNOO 'BMI.UNOO'.
EXECUTE.
SORT CASES BY BMI.UNOO (A).
SORT CASES BY BMI.UNOO (D).
SORT CASES BY BMI (A).
SORT CASES BY BMI.UNOO (A).
RECODE BMI (SYSMIS='99') (18.5 thru 24='1') (30 thru Highest='3') (24.1 thr
u 29.9='2') (15 thru
    18.4='0') INTO BMI.UNOO.
```

```
VARIABLE LABELS  BMI.UNOO 'BMI.UNOO'.
EXECUTE.
SORT CASES BY BMI.UNOO (A).
SORT CASES BY BMI.UNOO (D).
DATASET ACTIVATE DataSet1.

SAVE OUTFILE='C:\Users\rnordin.ADMIN\Desktop\2018\PUBLICATION 2018 ETS\ETS.
Data(Complete).sav '+
    '18APRIL2018.sav'
  /COMPRESSED.
DATASET ACTIVATE DataSet1.

SAVE OUTFILE='C:\Users\rnordin.ADMIN\Desktop\2018\PUBLICATION 2018 ETS\ETS.
Data(Complete).sav '+
    '18APRIL2018.sav'
  /COMPRESSED.
SORT CASES BY BMI.UNOO (A).
SORT CASES BY BMI.UNOO (D).
DATASET ACTIVATE DataSet1.

SAVE OUTFILE='C:\Users\rnordin.ADMIN\Desktop\2018\PUBLICATION 2018 ETS\ETS.
Data(Complete).sav '+
    '18APRIL2018.sav'
  /COMPRESSED.
DATASET ACTIVATE DataSet1.

SAVE OUTFILE='C:\Users\rnordin.ADMIN\Desktop\2018\PUBLICATION 2018 ETS\ETS.
Data(Complete).sav '+
    '18APRIL2018.sav'
  /COMPRESSED.
CODEBOOK  BMI.UNOO [n]
  /VARINFO POSITION LABEL TYPE FORMAT MEASURE ROLE VALUELABELS MISSING ATTR
IBUTES
  /OPTIONS VARORDER=VARLIST SORT=ASCENDING MAXCATS=200
  /STATISTICS COUNT PERCENT MEAN STDDEV QUARTILES.
```

Codebook

Notes

Output Created		19-APR-2018 19:23:...
Comments		
Input	Data	C:\Users\rnordin.ADMIN\Desktop\2018\PUBLICATION 2018 ETS\ETS.Data (Complete).sav 18APRIL2018.sav
	Active Dataset	DataSet1
	Filter	<none>
	Weight	<none>
	Split File	<none>
	N of Rows in Working Data File	118
Syntax		CODEBOOK BMI.UNOO [n] /VARINFO POSITION LABEL TYPE FORMAT MEASURE ROLE VALUELABELS MISSING ATTRIBUTES /OPTIONS VARORDER=VARLIST SORT=ASCENDING MAXCATS=200 /STATISTICS COUNT PERCENT MEAN STDDEV QUARTILES.
Resources	Processor Time	00:00:00.00
	Elapsed Time	00:00:00.04

[DataSet1] C:\Users\rnordin.ADMIN\Desktop\2018\PUBLICATION 2018 ETS\ETS.Data(Complete).sav 18APRIL2018.sav

BMI.UNOO

		Value	Count	Percent
Standard Attributes	Position	57		
	Label	BMI.UNOO		
	Type	String		
	Format	A8		
	Measurement	Nominal		
	Role	Input		
Valid Values	0	Underweight	11	9.3%
	1	Normal	54	45.8%
	2	Overweight	38	32.2%
	3	Obese	15	12.7%
