# Supplementary material for: A retrospective review on minimally invasive technique via endoscopic thoracic sympathectomy (ETS) in the treatment of severe primary hyperhidrosis: Experiences from the National Heart Institute, Malaysia
Source: F1000Res. 2018 May 29;7:670. [Version 1] doi: 10.12688/f1000research.14777.1 (PMC7338916; doi:10.12688/f1000research.14777.1)
Supplement: Supplementary file 3 [file f1000research-7-16079-s0002.tgz › a6348ee1-b770-4ea9-948b-a1057d466995.pdf]

ETS PROFORMA - IJN

MRN:	______________________________
Name:	______________________________
Age:	______________________________
Sex:	☐Male ☐Female
Race:	☐Malay ☐Chinese ☐Indian ☐Others:______________
Marital Status:	☐Married ☐Divorced ☐Single
Occupation:	______________________________
Address:	______________________________<br>______________________________
Contact no.:	Home:____________________<br>Mobile:_________________________
Height:	_________ (cm)
Weight:	__________(kg)
Date of Admission:	__________________(DD/MM/YYYY)
Co-morbidities:	______________________________<br>______________________________
Thyroid Function Test:	☐Normal ☐Abnormal: Hyperthyroidism/ Hypothyroidism (please circle)
Diabetes Mellitus:	☐Yes ☐No
Primary Hyperhidrosis	☐Face ☐Axilla ☐Hands ☐Feet ☐Trunk
Medical	☐No ☐Yes: _________________

Treatment

Operative Procedure	☐Bilateral ☐Unilateral: Left / Right (please circle)
Date of Operation:	________________ (DD/MM/YYYY)
Surgeon's name:	____

VATS DETAILS

Procedure:	☐Bilateral ☐Single
Patient Position:	☐Lateral ☐Supine/ Semi Upright
Number of Ports:	
Size of Ports:	Right: _____ (mm) Left: _______ (mm)
Thoroscope degree:	
Usage of CO2:	☐Yes ☐No
Level of Sympathectomy:	____
Method of Excision:	☐Thermocoagulation ☐Division ☐Others:________________
Histopathology Sent:	☐Yes ☐No
Histopathology Results:	Right HPE:____________________<br>Left HPE: _____________________<br>Right accessory nerve: _____________________<br>Left accessory nerve: ______________________
Duration of Surgery:	Right side: ___________________(mins)<br>Left side: ______________________(mins)

Total duration both sides: ____________________(mins)

Conversion to Open Surgery:	☐Yes ☐No
Local Anaesthetic Given:	☐Yes ☐No
Extubation:	☐Immediate ☐Delay
Post-Operative Inotrope:	☐Yes ☐No
Analgesia:	☐NSAIDs ☐Morphine ☐Others:_______________
ICU Stay:	☐Yes: ___________(hours) ☐No
Hospital Stay (Days):	________
Complications:	☐Yes ☐No
If Yes:	☐Pneumothorax ☐Horner's Syndrome ☐Respiratory Distress<br>☐Others: ____________________
Date of Discharge:	_________________(DD/MM/YYYY)
Follow-up:	☐Yes ☐No
Number of Follow-ups:	________
Issues:	☐Yes ☐No
If Yes:	______________________________________

Compensatory
Sweating: ☐Yes ☐No

When Noticed: ☐Immediate after surgery

☐Delayed:
State time after surgery: ______________________

Severity: ☐Very ☐Moderate ☐Mild

Location of CS: ☐Upper Back ☐Lower Back ☐Chest ☐Abdomen ☐Buttock

☐Groin ☐Back of thighs ☐Knees ☐Other:_____________

Follow-ups
Progression of CS: ☐Reduces ☐Increases ☐Status Quo

Last Follow-up: ☐Weeks after surgery:___________

☐Still On Follow-up
