# Supplementary material for: A retrospective review on minimally invasive technique via endoscopic thoracic sympathectomy (ETS) in the treatment of severe primary hyperhidrosis: Experiences from the National Heart Institute, Malaysia
Source: F1000Res. 2018 May 29;7:670. [Version 1] doi: 10.12688/f1000research.14777.1 (PMC7338916; doi:10.12688/f1000research.14777.1)
Supplement: Peer review history

# Peer review: Reviewer response for version 1

**DOI:** 10.5256/f1000research.16079.r65956

**Published:** 2020-07-06

**Authors:** Yongbing Chen

**Affiliations:** 1 Department of Thoracic Surgery, The Second Affiliated Hospital of Soochow University, Suzhou, China

The authors performed a retrospective study on the role of ETS in treating primary hyperhidrosis and found that ETS is a safe, effective and aesthetically remarkable procedure for the treatment of primary hyperhidrosis  with only half of the patients developing mild to moderate degree of compensatory sweating. However, the findings are actually not novel enough except the predictors of CS. In addition, the number of patients was too small to provide robust evidence for practice. Meanwhile, ETS procedure with two incisions in their institution seemed outdated since uniportal ETS is routinely performed in our center. Generally, the study is well-designed and the statistical methods seem sound, but the findings are not very novel. Last but not least, some grammatical errors exist. These errors should be corrected and more limitations should be acknowledged before the manuscript is acceptable.

Is the work clearly and accurately presented and does it cite the current literature?

Yes

If applicable, is the statistical analysis and its interpretation appropriate?

Yes

Are all the source data underlying the results available to ensure full reproducibility?

Yes

Is the study design appropriate and is the work technically sound?

Partly

Are the conclusions drawn adequately supported by the results?

Yes

Are sufficient details of methods and analysis provided to allow replication by others?

Yes

Reviewer Expertise:

thoracic surgery

I confirm that I have read this submission and believe that I have an appropriate level of expertise to confirm that it is of an acceptable scientific standard.

# Peer review: Reviewer response for version 1

**DOI:** 10.5256/f1000research.16079.r48307

**Published:** 2019-05-28

**Authors:** Nelson Wolosker

**Affiliations:** 1 Hospital Israelita Albert Einstein, São Paulo, Brazil

This is a review of a well-studied subject: retrospective results of assisted video sympathectomy in a single center.

 In the Introduction the authors make a general review on the subject. There is a final paragraph in the Introduction defining the aims of the study, which are written within the Material and Methods section. Remove the aims from the Methods and put them in the last paragraph of the Introduction.

 The casuistry is small, but significant. It is acceptable for a study demonstrating the results in Malaysia.

 The number of overweight and obese patients in this series is interesting. In international studies BMI greater than 25 generated greater intensity in SC.

 The results are adequate, demonstrating that the sympathectomy in the Malaysian population is similar to international. The great variation of the levels of sympathectomy is also interesting, since articles from 2005 to 2007 already demonstrated advantages of sympathectomy at T3 or T4 levels alone over other levels of sympathectomy. But they reflect the local reality, which makes this data valid.

 The conclusions are appropriate.

Is the work clearly and accurately presented and does it cite the current literature?

Yes

If applicable, is the statistical analysis and its interpretation appropriate?

Yes

Are all the source data underlying the results available to ensure full reproducibility?

Yes

Is the study design appropriate and is the work technically sound?

Yes

Are the conclusions drawn adequately supported by the results?

Yes

Are sufficient details of methods and analysis provided to allow replication by others?

Yes

Reviewer Expertise:

Hyperhidrosis.

I confirm that I have read this submission and believe that I have an appropriate level of expertise to confirm that it is of an acceptable scientific standard.
